# Turnover of sex chromosomes in the Lake Tanganyika cichlid tribe Tropheini (Teleostei: *Cichlidae*)

**DOI:** 10.1038/s41598-024-53021-3

**Published:** 2024-01-30

**Authors:** Kristen A. Behrens, Holger Zimmermann, Radim Blažek, Martin Reichard, Stephan Koblmüller, Thomas D. Kocher

**Affiliations:** 1 Department of Biology, University of Maryland, College Park, MD 20742 USA; 2 Institute of Vertebrate Biology, Czech Academy of Sciences, Květná 8, 603 00 Brno, Czech Republic; 3 Institute of Biology, University of Graz, Universitätsplatz 2, 8010 Graz, Austria; 4 Department of Ecology and Vertebrate Zoology, University of Łódź, Łódź, Poland

**Keywords:** Evolution, Genetics

## Abstract

Sex chromosome replacement is frequent in many vertebrate clades, including fish, frogs, and lizards. In order to understand the mechanisms responsible for sex chromosome turnover and the early stages of sex chromosome divergence, it is necessary to study lineages with recently evolved sex chromosomes. Here we examine sex chromosome evolution in a group of African cichlid fishes (tribe Tropheini) which began to diverge from one another less than 4 MYA. We have evidence for a previously unknown sex chromosome system, and preliminary indications of several additional systems not previously reported in this group. We find a high frequency of sex chromosome turnover and estimate a minimum of 14 turnovers in this tribe. We date the origin of the most common sex determining system in this tribe (XY-LG5/19) near the base of one of two major sub-clades of this tribe, about 3.4 MY ago. Finally, we observe variation in the size of one sex-determining region that suggests independent evolution of evolutionary strata in species with a shared sex-determination system. Our results illuminate the rapid rate of sex chromosome turnover in the tribe Tropheini and set the stage for further studies of the dynamics of sex chromosome evolution in this group.

## Introduction

Early methods for characterizing sex chromosomes used cytological approaches that were only capable of identifying highly differentiated sex chromosomes^1–4^. Sex chromosome systems were therefore originally classified by the morphology of the sex chromosome pair. The availability of inexpensive genome sequencing techniques has allowed the identification of sex chromosome pairs that do not show overt morphological differences. Often these are young sex chromosomes that have not yet diverged enough to be considered heteromorphic. The same XY or ZW terminology can be used for these homomorphic sex chromosomes. Male heterogametic systems are referred to as XY, and female heterogametic systems are referred to as ZW.

New sex determining alleles arising in a population may initiate a transition to a new sex chromosome^5–8^. The rate of sex chromosome turnover is highly variable among lineages. With only a few exceptions, such as the spiny rat^9^, there have been no turnovers of sex chromosomes in therian mammals or birds in the last ~ 100–180 million years^10,11^. In stark contrast, groups such as fishes and squamate reptiles show a high frequency of such turnovers^12–14^. Why some organisms experience high rates of turnover while others do not remains an unanswered question^15^. Hypotheses about the evolutionary mechanisms promoting sex chromosome turnover include sexually antagonistic selection^16,17^, accumulation of deleterious mutations^18,19^, and genetic drift^20^. Distinguishing these mechanisms empirically has been challenging and will require the discovery and characterization of sex chromosomes in additional species.

In many species, the chromosome region immediately adjacent to the sex-determining locus shows a reduction in recombination. This sex-linked region may expand over time through further recombination suppression, often creating ‘evolutionary strata’ exhibiting various levels of differentiation between the sex chromosomes. A predominant theory for the development of evolutionary strata is that each expansion associates advantageous sexually antagonistic variants with the sex-determining gene^8^. However, this theory has been difficult to prove empirically^21–23^ and has been challenged^24^. The mechanisms by which recombination becomes suppressed is also the subject of debate^23^. One recent model suggests that inversions expand in a stepwise manner from the sex-determining region because they shelter deleterious mutations^25^. Another theory proposes that co-evolution of cis and trans regulators of gene expression drives inversions on the Y^26^. All of these models should cause regions adjacent to the sex-determining region to cease recombination at different times, producing evolutionary strata^27–31^.

A large portion of the work on sex chromosome evolution has been conducted in mammals and birds. These two groups have ancient heteromorphic sex chromosomes with evolutionary strata that are readily apparent^10,11^. However, these systems are not ideal for studying the mechanisms of sex chromosome turnover or the early stages in the development of evolutionary strata. To understand the mechanisms of sex chromosome evolution we need to study lineages with a high rate of recent sex chromosome turnover. While highly divergent evolutionary strata have been identified in some fish, such as sticklebacks^32–34^, sex chromosomes in most fish species are homomorphic^35–37^. This homomorphy, and the high frequency at which sex chromosome turnover occurs, makes fishes ideal for studying sex chromosome evolution^36,38^. The direction of turnover, XY to ZW versus ZW to XY, is thought to be skewed in favor of the latter in fishes^12^. Instances of multiple sex chromosomes segregating within teleost species have been reviewed recently^39^, but the effects of polygenic systems on estimates of sex chromosome turnover have yet to be explored. Finally, a role for chromosome fusions in sex chromosome evolution has also been proposed in fishes^40^, and may contribute to reproductive isolation^38^.

We have focused on East African cichlid fishes (*Cichlidae*), a group known for its rapid radiation that generated species-rich lineages^41^. The relatively recent evolution of these species makes them ideal for studying evolutionary mechanisms. The high species density allows for a large number of comparisons among closely related species. Many sex chromosome turnovers have been detected in this clade, and at last count involved 12 of the 23 cichlid chromosomes^15,42,43^. The rate of sex chromosome turnover in this group has been calculated to be at least 0.186 turnovers per million years^42^.

**Figure 1:**
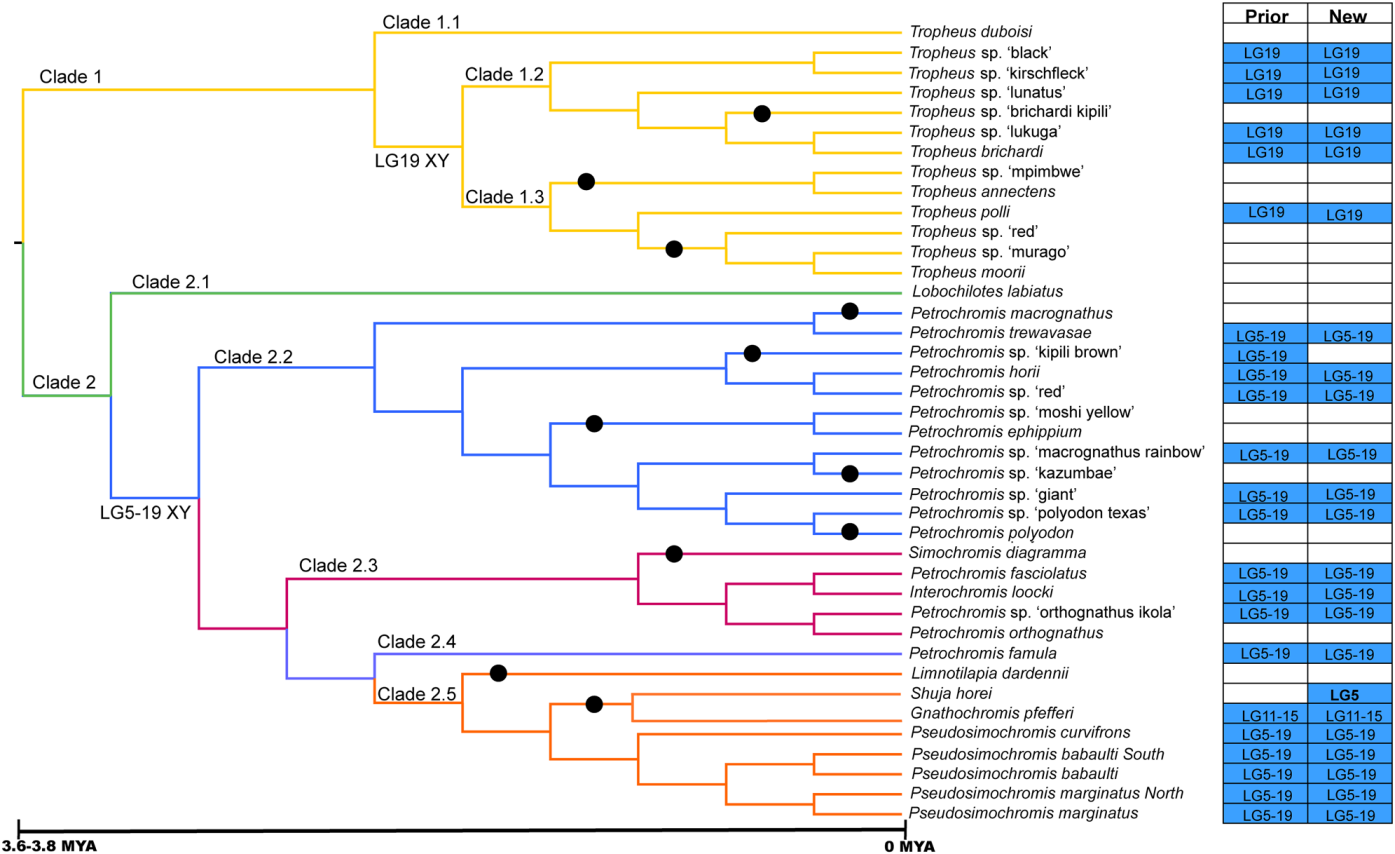
Phylogeny of Tropheini with previous sex chromosome calls and proposed sex chromosome systems. Some systems feature chromosomal fusions or multiple sex chromosomes. Cells in blue indicate an XY system, cells in orange indicate a ZW system. Bolded text indicates disagreement between previous call and current call. Black circles indicate loss of sex chromosome common to that clade. Topology adapted from Ronco et al.^44^ and approximate time scale is from Singh et al.^48^.

The Tropheini, one of the thirteen cichlid tribes in Lake Tanganyika^44^, is particularly well-suited for such studies. The Tropheini consist of ~ 40 species grouped into eight genera^45–47^ which shared a common ancestor ~ 3.6–3.8 million years ago^44,48^. Genera in the Tropheini are defined by their distinct jaw morphologies^49^, and previous phylogenetic analysis has identified several subclades within the tribe^44,48^ (Fig. 1). Clade 1 consists of 13 species of *Tropheus* divided into 3 subclades. Clade 1.1 contains a single species, *T. duboisi*, which is sister to the rest of Clade 1. Clades 1.2 and 1.3 each contain 6 species. Clade 2 contains the 27 remaining species that can be grouped into five sub-clades. Clade 2.1 consists of the single species *Lobochilotes labiatus*, which is an outgroup to the other four clades. Clade 2.2 contains 12 species of *Petrochromis*. Clade 2.3 contains *Simochromis diagramma*, *Interochromis loocki* and 3 more species of *Petrochromis.* Clade 2.4 consists of a single species, *Petrochromis famula* and Clade 2.5 includes *Limnotilapia dardennii, Shuja horei, Gnathochromis pfefferi* and five species of *Pseudosimochromis.*

**Figure 2:**
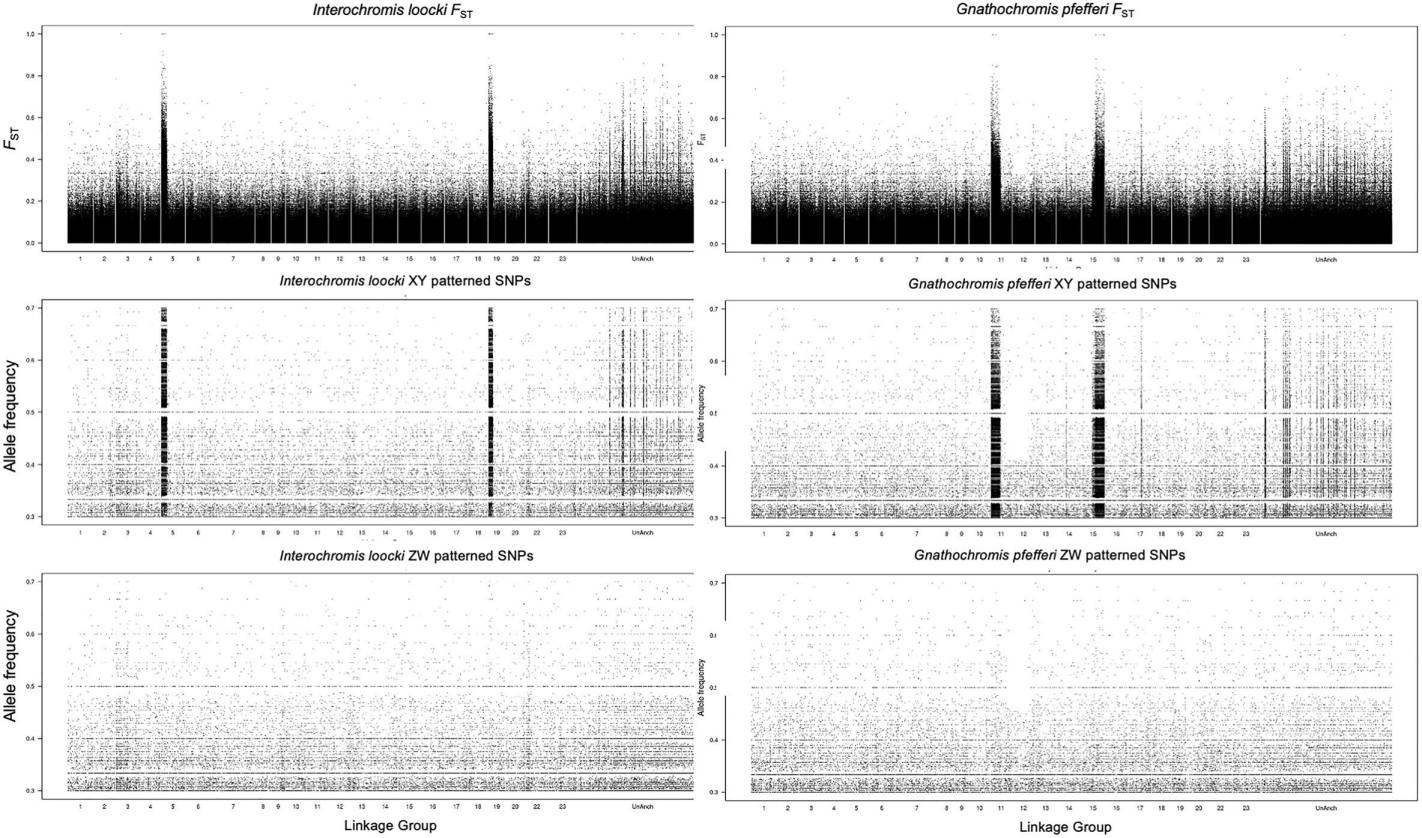
Pairwise male x female *F*_ST_ and sex specific allele frequency in pool-seq data for *Interochromis loocki* and *Gnathochromis pfefferi*. The x-axis is position on the *Metriaclima zebra* (UMD2a) reference assembly.

Our previous study identified an XY system on LG19 in *Tropheus* sp. ‘black’^43^. A more recent survey of 40 taxa within the tribe identified three distinct sex chromosome systems, two of which are shared by multiple species^42^. Species of the genus *Tropheus* (Clade 1) were found to segregate the XY system on LG19, while species of *Petrochromis* and *Pseudosimochromis* (Clade 2) segregated an XY system involving an apparent fusion of LG5 and LG19. *Gnathochromis pfefferi* (Clade 2.4) carried an XY system involving an apparent fusion of LG11 and LG15^42^ (Fig. 2).

Here we use new data from pooled sequencing of males and females in several species, together with a reanalysis of previously published sequences of single male and female individuals^42^, to identify new sex chromosome systems in this tribe. We quantify the high rate of sex chromosome turnover and also describe the independent development of evolutionary strata on LG19 among species. Our study provides new insights into the early stages of sex chromosome evolution in this group.

## Methods

Cichlid sex chromosomes are usually at an early stage of differentiation. This means the allele frequency differences between the sex chromosomes are small and often present at a relatively small number of sites. Furthermore, the characteristics of the signal changes with the age of the system. At first the main signal will be heterozygosity of a few sites in the heterogametic sex. However, with continued divergence, some sequences are lost from the Y (or W) chromosome, leading to apparently contradictory signals. For example, loss of a sequence from the Y chromosome makes males hemizygous at some sites, so that heterozygosity of these sites on the X falsely suggests a ZW system. Many signals typically quantified for older sex chromosome systems (e.g. differences in base composition or sequence coverage) are not present in the very recently evolved systems in cichlids.

Identification of these signals is further complicated by the small effective size of many cichlid populations. A small number of large haplotype blocks in these populations can lead to identification of spurious sex-associated blocks when small numbers of male and female individuals are sampled. Even when a larger number of individuals are sampled, the small Ne can lead to high background levels of allele frequency differences between the male and female samples.

Finally, there is a possibility that polygenic systems segregate in these species, arising from either the high rates of sex chromosome turnover, or the maintenance of balanced polymorphisms over hundreds of thousands of years. Sampling for sex chromosome surveys is generally not designed to identify multiple sex chromosome systems segregating in a population. With these challenges in mind, we used a variety of approaches to extract the maximum information from the limited data available.

### DNA samples and sequencing

We obtained new sequencing data from pooled males and females from four species collected in 2019 near Kalambo Lodge, Zambia near the southern end of the lake (-8.623°N, 31.200°E): 25 males and 23 females of *Pseudosimochromis babaulti,* 23 males and 25 females of *Gnathochromis pfefferi*, 24 males and 21 females of *Interochromis loocki*, and 25 males and 25 females of *Shuja horei* (*Ctenochromis horei*)^50^. We also analyzed a full-sib family of 30 males and 28 females of *Simochromis diagramma* reared at U. Graz. Finally, we reanalyzed data from 30 males and 24 females of *Tropheus* sp. “black” that were wild caught near Ikola, Tanzania^43^. Animal experiments were conducted in accordance with the Guide for Care and Use of Laboratory Animals and reported in accordance with ARRIVE guidelines^51^. All animal use was approved under Zambian research permit K-4335/18 KA/K.48/18 (S. Koblmüller) and animal care protocols R-OCT-19-48 (U. Maryland) and BMWFW-66.007/004-WF/V/3b/2016 (U. Graz). This study was carried out with the approval of the ethics committee of the University of Graz (permit number GZ. 39/115/63 ex 2022/23).

DNA was extracted from fin clips by phenol–chloroform extraction using phase-lock gel tubes (5Prime, Gaithersburg, MD). DNA concentrations were quantified by fluorescence spectroscopy using a Quant-iT PicoGreen assay (ThermoFisher, Waltham, MA). Equimolar amounts of DNA from each individual were then pooled by sex for each species. Sequencing libraries were constructed, and 150 bp paired-end DNA sequencing was performed on a NovaSeq6000 S4 (Illumina, San Diego CA) by Novogene US (Davis, CA).

Sequence data from previous studies were retrieved from the NCBI Short Read Archive and GenBank. This included whole genome sequence data from single males and females for the species in the tribe *Tropheini,* analyzed by El Taher et al.^42^. We also examined transcriptome data for 9 species, which were also included in that study (Suppl Table S1).

### Sex-specific SNP analysis

The main basis of our analyses is the identification and analysis of sex-specific SNPs. These SNPs were identified following the methods described in Behrens et al.^52^ using the pipeline developed by Gammerdinger et al.^43^. Previously reported code from that study is available (https://github.com/Gammerdinger/sex-SNP-finder). Briefly, the sequence reads were aligned with BWA version 0.7.12 using the default parameters along with read group labels^53^. We aligned all samples to the closest high-quality reference assembly, the Malawi zebra (*Maylandia zebra*—UMD2a, RefSeq GCF_000238955.4)^54^. In some cases, some of the sex-specific signal mapped to unanchored contigs of the Malawi zebra assembly, so we remapped the reads to the more contiguous assembly of Nile tilapia (*Oreochromis niloticus—*UMD_NMBU, RefSeq GCF_001858055.2)^55^. At each variable nucleotide site we calculated the *F*_ST_ statistic between the populations of male and female sequence reads. The resulting *F*_ST_ plots provide a first indication of the differentiation between male and female genomes. We further identified XY- and ZW- patterned SNPs as SNPs that were fixed (frequency less than 0.1) in one sex and polymorphic (frequency between 0.3 and 0.7) in the other sex. Separate plots of the allele frequency of XY- and ZW- patterned SNPs often allowed determination of the type of heterogametic system segregating (XY or ZW).

Next we used Bedtools^56^ *make windows* and *coverage* to calculate the density of sex-patterned SNPs in 100kbp windows across the genome. We identified the top 1% of windows (~ 78 of 7,800 anchored windows) with the highest number of sex-patterned SNPs using the methodology described in Kocher et al.^57^. The log_2_(XY:ZW) ratio of SNP density was then calculated for each window^58^. A Kruskal–Wallis (KW) test on the ranked data was conducted in R (v.2023.03.0 + 386) using kruskal.test from the *stats* package to determine if the log ratio differed among chromosomes^59^. If the differences were statistically significant, the Dunn’s test from the *rstatix* R package was conducted post-hoc to determine which chromosomes differed significantly from one another with Benjamini–Hochberg correction for multiple tests. Regions of elevated sex-specific SNP density were visualized in IGV^60^ to identify candidate sex determining genes.

### k-mer analysis

To identify sex-linked variants shared by species with the same sex chromosome system we conducted a k-mer analysis. Following our previous work, we used using Jellyfish v. 2.2.7^61^ to tabulate the frequency of k-mers of length 22 bases^52^. Code from that study is available (https://github.com/KristenBehrens/K-mer-scripts). Briefly, for each shared sex chromosome system, a core region was identified using a python script that compares k-mers from each species and generates a list of k-mers shared by all species with that system. These k-mers were then aligned to the *M. zebra* reference genome (M_zebra_UMD2a) using BLAST to determine the core region of differentiation. Samtools v. 1.10 was used for any necessary file format conversions, sorting, or indexing. This analysis was conducted for species with the XY-linkage group (LG) 5/19 system and to compare the XY-LG5 system between *Shuja horei* and *Cyprichromis pavo*.

## Results

### Identification of sex chromosomes from pooled sequence data

In the new pool-seq data for five species reported here, we confirmed three systems previously identified from individual sequence data^42^. The sex chromosomes confirmed were the XY-LG5/19 systems in *Interochromis loocki* and *Pseudosimochromis babaulti,* and the XY-LG11/15 system in *Gnathochromis pfefferi* (Fig. 2)*.*

**Figure 3:**
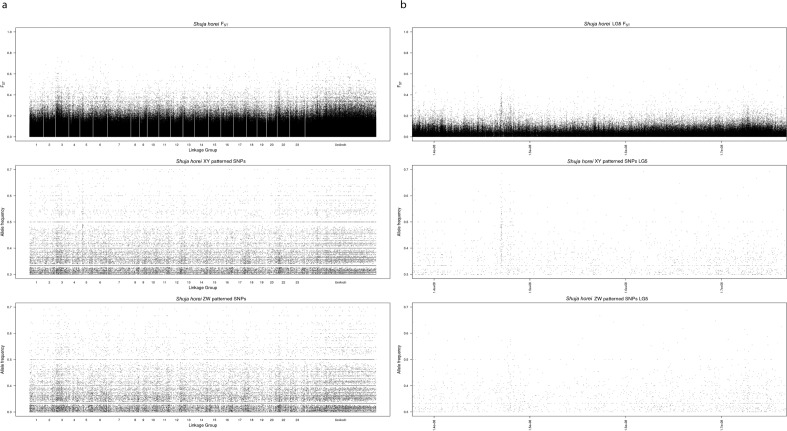
Pairwise male x female *F*_ST_ and sex specific allele frequency for *S. horei*, (**a**) whole genome, (**b**) LG5. The x-axis is position on the *Metriaclima zebra* (UMD2a) reference assembly.

The *F*_ST_ plots of the pool-seq data for *S. horei* (Clade 2.5) show no evidence of the XY-LG5/19 system common in the rest of Clade 2, but do suggest an XY system on LG5 (Fig. 3). This narrow peak (~ 1.5 Mb wide) is located at 7.8–7.9 Mbp, which is the top window of differentiation containing 80 XY-patterned SNPs. However, this narrow peak did not yield a significant Kruskal–Wallis test for heterogeneity among chromosomes for either the single individual data or the pooled sequencing data (Suppl Table S2). The peak (7.8–7.9 Mbp) encompasses two genes, *ncoa3* and *id1*, both of which might be considered candidate genes for male sex determination. *ncoa3* (*src-3*, *ab1*) is a member of the p160 steroid receptor coactivator family that regulates transcription^62,63^. This gene has been found to control cell migration in the ovary in both *Drosophila* and humans^64^. *id1* is an inhibitor of differentiation protein that acts during embryogenesis to promote cell growth^65^. This gene is mediated upstream by the TGF-beta pathway^66^ which has been implicated in sex determination in cichlids and other fishes^67–69^. However, the highest density of male-specific SNPs fell in the region between *ncoa3* and *id1*, which has no annotated gene in the *M. zebra* assembly. The differentiation on LG5 in *S. horei* is not related to the XY system on LG5/19 seen throughout Clade 2, as the peaks on LG5 are in different regions of the chromosome.

The *F*_ST_ plot for the pooled sample of *Simochromis diagramma* shows no evidence of the XY-LG5/19 system common in Clade 2. Instead, we observe strong differentiation on LG7 (Suppl Fig. S1). The top 1% windows include 45 ZW and 34 XY windows on LG7. Although the KW test for heterogeneity among chromosomes is significant, the Dunn's tests do not suggest unusual differentiation of LG7 relative to other chromosomes, but instead suggest differentiation of LG2, LG8 and LG20. In contrast, the top 1% windows in the comparison of a single male and female shows 22 XY windows on LG8 and 11 XY windows on LG16. The KW test for heterogeneity is also significant and the Dunn's test suggests unusual differentiation of LG8, LG16 and LG18. The multiple signals, and the differences between the individual samples and the pooled sequencing of the family suggest this species may be segregating multiple sex chromosome systems.

### Confirmation of sex chromosomes from individual sequence data

We also reanalyzed a published dataset consisting of whole genome sequence reads from a single male and single female for most species of *Tropheini* and were able to confirm many of the systems identified by El Taher^42^*.* These include an XY-LG19 system predominant in the *Tropheus* of Clade 1, which we confirmed in *Tropheus* sp. 'black', *T.* sp. 'kirschfleck', *T*. sp. 'lunatus', *T.* sp. 'lukuga', *T. brichardi* and *T. polli.* Each of these species showed strong statistical support for the XY-LG19 system. The KW tests for heterogeneity were significant, the Dunn's test for differences among chromosomes implicated LG19, and nearly all of the top 1% windows were located on LG19 (Suppl Table S2). Equally important, we confirmed the absence of the XY-LG19 system in many species of Clade 1. We found no evidence of XY-LG19 in *T*. sp. 'brichardi kipili', *T*. sp. 'mpimbwe', *T. annectens*, *T*. sp. 'red', *T*. sp. 'murago' or *T. moorii.* The strong XY-LG19 signals we observed in the sister taxa of each of these species allows the strong inference that the XY-LG19 system is not present in our samples of these species. We infer 3 independent losses of the XY-LG19 system in Clade 1 (Fig. 1).

We also confirmed an XY-LG5/19 system in many species from Clade 2, including *Petrochromis trewavasae*, *P. horii*, *P.* sp. 'red', *P.* sp. 'macrognathus rainbow', *P*. sp. 'giant', *Interochromis loocki*, *P.* sp. ‘orthognathus Ikola’, *P. famula*, *Pseudosimochromis curvifrons*, *P. babaulti South*, *P. babaulti*, *P. marginatus North* and *P. marginatus.* These species showed evidence for an XY-LG5/19 system across multiple tests (Suppl Table S2). We agree with the conclusion of El Taher et al. ^42^ that the LG19 system seen in the Clade 1 is different from the LG19 system in Clade 2. These two systems involve different regions of LG19, and their haplotypes do not group together phylogenetically^42^.

We found less support for the XY-LG5/19 system in several other species (Suppl Table S2). *Petrochromis* sp. ‘polyodon Texas’ shows top 1% window support for XY-LG5/19, and KW heterogeneity among chromosomes. The Dunn's tests support differentiation of LG5 and LG19, but also LG8 and LG18. In the absence of top 1% window support for LG8 and LG18, we conclude this species is segregating the XY-LG5/19 system. *Petrochromis fasciolatus* shows KW heterogeneity and top1% support for 5/19 but the Dunn's results are scattered. Again, we conclude this species segregates the XY-LG5/19, and the scattered Dunn's test results arise from artifacts of the small sample. Finally, we were unable to confirm the claim of an XY-LG5/19 system in *Petrochromis* sp. ‘kipili brown’^42^. Our analysis shows KW heterogeneity, but the top 1% windows suggest XY signal for LG4, LG11, LG20, LG22. We are unable to identify a sex chromosome system in this species because there are many significant Dunn's tests, again suggesting artifacts of small sample size or identity by descent in a small population. For the remaining 9 species in Clade 2 we could find no support for an XY-LG5/19 system, possibly indicating that the XY-LG5/19 system has been lost and replaced by a new sex chromosome. Such replacements may have occurred as many as 8 times during the evolution of Clade 2 (Fig. 1).

We were also able to confirm the XY-LG11/15 system in *G. pfefferi*, which is the second instance of sex chromosome fusion in this tribe. The X and Y chromosomes are strongly differentiated, as seen in the *F*_ST_ plot (Fig. 2), and all of the top 1% windows are XY windows falling on LG11 and LG15 (Suppl Table S2). This high level of differentiation suggests that this sex chromosome is similar in age to the XY-LG5/19 system in Clade 2.

### Possible new systems identified in single individual data

We found provisional support for several additional systems in Clade 1. We mention them so that they may be examined with additional data in the future. None of these species showed evidence of the LG19 system found in other species of Clade 1. *Tropheus duboisi* has no obvious signal in the *F*_ST_ plot. The KW test for heterogeneity is significant, and Dunn's tests identify LG9 as distinct. There are 4 top 1% windows that suggest an XY system on LG9, but the overall ratio of XY to ZW SNPs on LG9 is strongly biased toward ZW. *Tropheus* sp. 'brichardi kipili' shows no obvious signals in the *F*_ST_ plot, and the KW test is not significant. We are unable to identify a sex chromosome for this species. *Tropheus* sp. 'mpimbwe' show no obvious signal in the *F*_ST_ plot. The KW test is moderately significant, and the Dunn's tests identify LG8 as distinct. However, there are no top 1% windows on LG8. *Tropheus annectens* shows a narrow peak of *F*_ST_ on LG8 suggestive of a ZW system. The KW test is significant, but the Dunn's tests suggest that LG14 and LG23 are distinct from the other chromosomes. The top 1% windows include one ZW window on LG8, two ZW windows on LG14, and one XY and six ZW windows on LG23. The *F*_ST_ plot for *Tropheus* sp. 'red' is unremarkable, and the KW test is not significant. The *F*_ST_ plot for *Tropheus* sp. 'murago' suggests a possible XY system on LG6, but the KW test is insignificant and there is no support in the top 1% windows. The *F*_ST_ plot for *Tropheus moorii* is unremarkable, the KW test is barely significant, and there is no obvious system indicated in the top 1% windows.

We also found provisional support for additional systems in Clade 2. None of these species showed evidence of the XY-LG5/19 system found in other species of Clade 2. The *F*_ST_ plots for *Petrochromis macrognathus* do not show obvious sex signals, and the KW and Dunn's tests are only moderately significant. *Petrochromis* sp. 'moshi yellow' shows some possible *F*_ST_ peaks, and the KW test is significant. However, the Dunn's results are weak and scattered, and the top 1% windows do not support any particular chromosome. The *F*_ST_ plot for *Petrochromis ephippium* is unremarkable, but the KW test is significant and the Dunn's tests highlight LG3 and LG4. The top 1% windows suggest a ZW system on LG4. The *F*_ST_ plot for *Petrochromis* sp. 'kazumbae' is also unremarkable, but the KW test is significant, and the Dunn's tests highlight LG23. *Petrochromis polyodon* shows no obvious *F*_ST_ peaks, and the KW test is moderately significant. The Dunn's tests do not highlight any chromosome. The evidence for multiple sex systems segregating in *Simochromis diagramma* was discussed in detail in the section on pooled sequencing results above. The *F*_ST_ plot for *Petrochromis orthognathus* is unremarkable, but the KW is significant, and the Dunn's tests suggest a system on LG13. There is a single top 1% window on LG13 that contains 18 ZW-patterned SNPS. The *F*_ST_ plot for *Limnotilapia dardennii* is unremarkable, the KW test is moderately significant but only a handful of the Dunn's test comparisons show weak significance. The evidence for an XY-LG5 system in *Shuja horei* was discussed in detail in the section on pooled sequencing results above.

Discovery of sex chromosomes using the single individual data was impeded by high levels of background noise in the sex-specific SNP analyses (e.g. *Petrochromis* sp. 'giant' in Suppl Fig. S2). Single individual data is not ideal for identifying sex chromosomes regardless of analytical methodology. We hope that calling attention to these possible sex chromosome signals will motivate further research.

### Origins of the sex chromosomes in Clades 1 and 2

Clades 1 and 2 both include species with an XY system that involves LG19. In order to determine whether these species share elements of an ancestral sex determination system, we compared male-specific k-mers between Clade 1 and Clade 2.2. We found only 10 shared k-mers that aligned to the reference assembly. Of these, eight k-mers representing 2 SNPs aligned to LG19, one aligned to LG2, and the last aligned to LG6. Of the k-mers associated with LG19, all fell in the same region (~ 10.1 Mbp) in the gene *kiaa1109*, well within the sex-linked region in Clade 1, but just outside the right-most boundary of the sex-linked region in Clade 2.2. Thus, it is likely not a candidate sex determining gene, and it is unlikely that this handful of shared k-mers, representing just 2 SNPs, are evidence for a shared origin of LG19.

The species in Clade 1 with an XY-LG19 system share 320,209 k-mers. Of these, 245,240 (76%) are Y-specific k-mers on LG19 representing approximately 10,000 SNPs. The large number of shared SNPs on LG19 are indicative of an old sex chromosome system with a relatively long period of shared ancestry before the current species diverged. The SNPs are distributed relatively evenly over a large portion of LG19, and so do not help to localize a candidate sex determination gene.

We also quantified shared k-mers for species with the XY-LG5/19 system in Clade 2. The four subclades **(**2.2–2.5) of Clade 2 share only 20 Y-specific k-mers, of which 18 aligned to LG5. The majority of these k-mers (14) correspond to a single SNP that maps to ~ 5.127 Mbp within an intron of the gene *bin2b*, which has no obvious function in sex determination. The other four k-mers on LG5 represent a single SNP mapped to ~ 17.57 Mbp in a region that contained no annotations. The absence of a large number of SNPs shared across clades suggests that the radiation of species occurred very quickly after the emergence of the new sex determiner. Therefore, most of the sequence differentiation in the sex-determining region has occurred independently in each lineage.

The distantly related tribe Cyprichromini also has an XY system on LG5^52^. We counted k-mers shared between *Cyprichromis pavo* and *P. trewavasae* (XY-LG5/19). These two species shared slightly more k-mers on LG5 and LG19 than on the other chromosomes (Suppl Table S3). However, given the phylogenetic distance between these clades, these similarities are unlikely to represent shared origin of the sex chromosome systems in these two tribes. A comparison between *Cyprichromis pavo* and the recently evolved XY-LG5 system in *S. horei* did not show an excess of shared k-mers on LG5.

### Evolutionary strata

Sex chromosome systems shared by multiple species off the opportunity to study the early divergence of sex-linked regions into evolutionary strata. We characterized male-specific SNP density per 100 kb window and found interspecific variation in the extent and levels of sequence differentiation of the sex determining regions that suggests the presence of multiple evolutionary strata.

Six species in Clade 1 share the XY-LG19 system. Although the number of XY-patterned SNPs varies among species, there are no obvious differences in the pattern or chromosomal extent of the differentiation (Suppl Fig. S3). The variation in the number of sex-patterned SNPs likely reflect differences in *N*_e_ among species.

Sixteen species in Clade 2 share the XY-LG5/19 system. The first 9 Mb of LG5 shows differentiation in all species (Suppl Fig. S4). The species in Clades 2.2, 2.3 and 2.4 show no further expansion from the core 9 Mb. In Clade 2.5, *P. babaulti South* does not appear to have expanded beyond the 9 Mb core, but the remaining 3 species share a stratum that extends and additional ~ 4 Mb. *Pseudosimochromis babaulti* appears to have an additional stratum that extends and additional ~ 4 Mb beyond the core.

**Figure 4:**
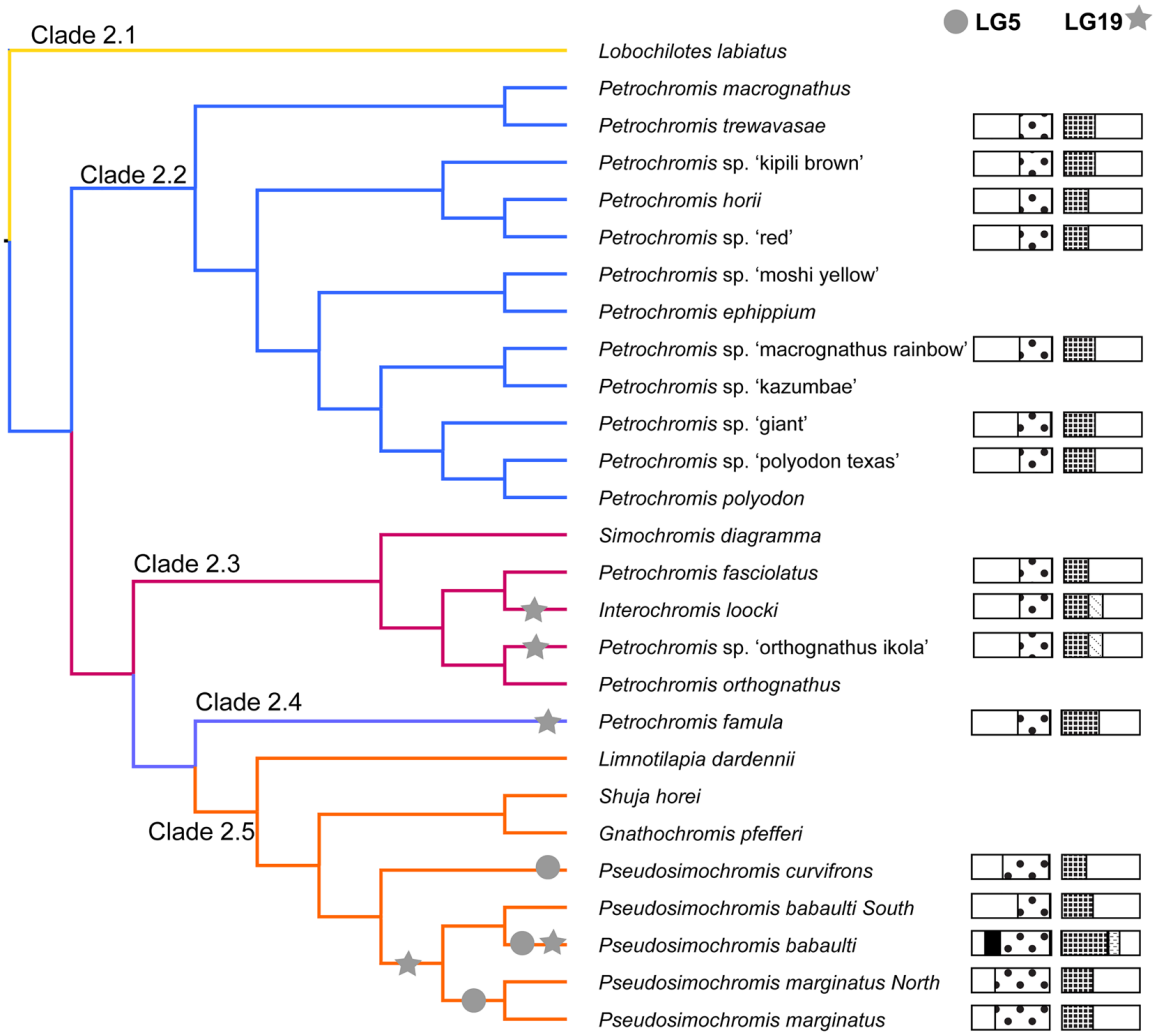
Expansion of the XY-LG5/19 sex chromosome on the phylogeny of Clade 2 as estimated from sex-specific SNP density data. Circles indicate expansion of LG5, stars indicate expansion of LG19. For LG5 the dotted pattern indicates what is presumably the original region, as does the plaid pattern for LG19, with any further additions in different patterns representing potential evolutionary strata. Phylogeny adapted from Ronco et al.^44^.

The expansion of evolutionary strata on LG19 appears to have occurred independently in each clade (Suppl Fig. S5). The species in Clade 2.2 share a stratum from 0 to 5 Mb. The species in Clade 2.3 share a stratum from 0 to 6 Mb, while in Clade 2.4 (*P. famula*) it extends approximately 0–8 Mb. In Clade 2.5 the differentiated region in *P. curvifrons* extends 0–5 Mb, but in *P. babaulti South*, *P. marginatus* and *P. marginatus North* it extends 0–7 Mb, and in *P. babaulti* it extends all the way from 0 to 9 Mb. The expansion of these two regions in each species is summarized in Fig. 4.

## Discussion

### Rates and patterns of sex chromosome turnover

Cichlids are an ideal system in which to examine the rates and patterns of sex chromosome turnover due to their high species diversity and relatively recent divergence. Our analysis of the tribe Tropheini further contributes to the narrative that East African cichlid fishes have some of the highest rates of sex chromosome turnover in vertebrates.

The ancestral sex chromosome state for the tribe Tropheini is unknown. We were unable to identify sex chromosome systems in the deepest lineage of Clade 1 (*T. duboisi*) or Clade 2 (*L. labiatus*). Given the high rate of sex chromosome turnover in the group, and the large phylogenetic distance to neighboring tribes, we are unable to reconstruct the ancestral state for the tribe with any confidence. A previous study found significant probabilities for 5 different ancestral states for the common ancestor of the tribe^42^.

Although we were unable to identify a sex chromosome system in the deepest lineage of Clade 1 (*T. duboisi*), the ancestral state for the rest of Clade 1 appears to have been XY-LG19. The signal for LG19 in several species in Clade 1.2 and in *T. polli* (Clade 1.3) is strong and encompasses at least 60% of the chromosome. This signal should be detectable in other species in Clade 1 with our current methodology even in samples of single males and females. The absence of such signals in six species of Clades 1.2 and 1.3 is thus evidence for at least 3 additional sex chromosome turnovers.

We were also unable to identify the sex chromosome system in the deepest lineage of Clade 2 (*L. labiatus*). The ancestral state for the remainder of Clade 2 appears to have been XY-LG5/19, which is found in each of the major subclades (2.2, 2.3, and 2.5). This system also shows high levels of differentiation between the X and Y chromosomes over roughly 30% of both LG5 and LG19. This signal should also have been detectable in samples of single individuals. The absence of this signal in 12 species of Clade 2 suggests at least 10 additional sex chromosome turnovers have occurred.

A turnover to XY-LG11/15 in *G. pfefferi* is strongly supported by the pool-seq data. The sex-differentiated regions in *G. pfefferi* encompass roughly half of each chromosome and show differentiation similar in magnitude to the XY-LG5/19 system and is thus relatively old. Our data also suggest a recent turnover to an XY-LG5 system in *S. horei*. The region of differentiation is only about 100 kb, but already shows 82 XY-patterned SNPs.

The other 9 species lacking the XY-LG5/19 system represent at least 7 independent losses, presumably accompanied by the rise of different system of genetic sex determination. In total, from the root of the tribe Tropheini there have been at least 12 sex chromosome turnovers, leaving aside the unknown sex systems in *T. duboisi* and *L. labiatus* (Fig. 3). Assuming 12 turnovers and the branch lengths of the time-calibrated phylogeny from Singh et al.^48^ which suggests that the tribe Tropheini is 3.6–3.8 million years old, we estimate the rate of sex chromosome turnover for this tribe is at least 0.259 turnovers per million years.

### Polygenic sex determination

There is disagreement on the stability of polygenic sex chromosome systems, as some argue they should be should be unstable intermediates or the result of recent hybridization^70,71^. However evidence in house flies (*Musca domestica*)^72,73^ and in cichlids, where multiple sex chromosome systems can persist within species for periods of up to a million years, suggests otherwise. This is evident from work on Lake Malawi cichlids, where sex loci associated with both the orange-blotch (OB) inversion^74,75^ and B chromosomes^57,76,77^ are broadly shared among species. Further support comes from the polymorphism of sex chromosomes in *Astatotilapia burtoni*^57,74,75,77,78^. Finally, long-term sex chromosome polymorphism has been described in the Lake Tanganyika tribe *Cyprichromini*^52^. Similar polymorphisms may have been overlooked in less species-rich clades, especially because most sampling strategies are not designed to detect multiple sex chromosome systems segregating in a population.

We discovered some evidence for sex chromosome polymorphism in *S. diagramma*. The pool-seq analysis revealed strong differentiation on LG7, but the analysis of single individuals from another sample suggested XY systems on LG8 and 16. The lack of sex chromosome signal in the other species which do not show either the XY-LG19 or XY-LG5/19 system can most likely be attributed to the recent invasion of a new, relatively undifferentiated sex chromosome that is not detectable using our GWAS approaches. But it might also indicate the segregation of multiple sex chromosome systems that would tend to obscure the signal in pool-seq analyses.

### Chromosome fusions

The relationship between the evolution of karyotypes, sex chromosomes and speciation has been the subject of considerable research^79,80^. Sex chromosomes can drive overall karyotype evolution via inversions, deletions, and fusions^40^. Chromosome fusions have been proposed to be involved in sex chromosome turnover^38^, which may further result in speciation as has been proposed in stickleback^34^. Supernumerary B chromosomes have become sex-determining in some Lake Malawi cichlids^81^, and we have suggested that a fusion between a B chromosome and a sex chromosome generated the giant LG3 chromosome found only in the tilapia lineage^76^.

The typical cichlid diploid chromosomal number is (2n = 44)^82^. Karyotypic data is not available for most species of the Tropheini*,* but we predict at least two fusions in this clade. The first is predicted by the strong sex signals of an XY-LG5/19 system in Clade 2, We predict that karyotypes of these species will display 2n = 42. The second is the XY-LG11/15 system in *G. pfefferi*, which we predict will display 2n = 40. In both cases, the fusions likely preceded the development of sex chromosome characteristics. The differentiated regions are found at the end of these chromosomes, where cichlid recombination rates are typically low^54^. These regions of reduced recombination may have facilitated the development of sex chromosomes in these species.

### Evolutionary strata

The multiple versions of the Y chromosomes in these clades provide an opportunity to study the dynamics of the formation of evolutionary strata on the sex chromosomes. We summarize our findings in Fig. 4. *Pseudosimochromis babaulti* in Clade 2.5 appears to have the largest region of differentiation on both LG5 and LG19. The other species in Clades 2.4 and 2.5 have experienced some expansion as well, but not to the same extent as *P. babaulti*. The overall pattern suggests that the initial stratum was established over the ~ 5 Mb terminal regions of each chromosome, which were telomeric regions prior to chromosome fusion. Low recombination rates seem to persist in these formerly telomeric regions for millions of years after chromosome fusion (e.g. LG23)^54^. These regions of inherently low recombination may represent a favorable environment for the evolution of new sex chromosomes.

The definition of very young evolutionary strata remains a challenging problem. Because the levels of differentiation are low, and the differences between strata even smaller, it is difficult to delineate the boundaries of individual strata from measures of sequence differentiation alone. Stringent definition of these strata may require phased assemblies of the sex chromosomes for each species.

## Conclusion

With each new study it becomes evident that the variability of the sex chromosomes in East African cichlids is greater than previously anticipated. This is an important insight because when we underestimate the number of sex chromosome systems, we undercount the number of turnovers and may infer a different pattern of sex chromosome replacement. We also undercount the number of autosomes that have become sex chromosomes, which skews our understanding of which chromosomes and genes can become involved in sex determination. Most of what we understand about sex chromosome evolution comes from distantly related organisms with old and very diverged sex chromosomes. Only in groups like cichlids, with recent, species-rich clades, can we begin to understand the early stages of sex chromosome evolution.

### Supplementary Information

Supplementary Information 1.

Supplementary Information 2.

## Data Availability

The new sequence data for *Pseudosimochromis babaulti, Shuja horei* (previously called *Ctenochromis horei*)*, Gnathochromis pfefferi, Simochromis diagramma,* and *Interochromis loocki* are available on NCBI under BioProject: PRJNA802233. Previously published data are available under their respective BioProjects on NCBI: Gammerdinger et al. 2018 BioProject: PRJNA400462 and El Taher et al. 2021 BioProject: PRJNA552202.
