# Supplementary material for: Turnover of sex chromosomes in the Lake Tanganyika cichlid tribe Tropheini (Teleostei: Cichlidae)
Source: Sci Rep. 2024 Jan 30;14:2471. doi: 10.1038/s41598-024-53021-3 (PMC10828463; doi:10.1038/s41598-024-53021-3)
Supplement: Supplementary file 1 — Supplementary Information 1. [file 41598_2024_53021_MOESM1_ESM.pdf]

Supplementary Tables and Figures

Turnover of Sex Chromosomes in the Lake Tanganyika cichlid tribe Tropheini (Teleostei: *Cichlidae*)

Kristen A. Behrens [1], Holger Zimmermann[2,3], Radim Blažek[2], Martin Reichard[2,4], Stephan Koblmüller [3], Thomas D. Kocher [1]

[1] Department of Biology, University of Maryland, College Park MD 20742 USA

[2] Czech Academy of Sciences, Institute of Vertebrate Biology, Květná 8, 603 00 Brno, Czech Republic

[3] Institute of Biology, University of Graz, Universitätsplatz 2, 8010 Graz, Austria

[4] Department of Ecology and Vertebrate Zoology, University of Łódź, Łódź, Poland

Tables

Supplemental Table 1. Samples from the tribe *Tropheini*

**Species**	**Sex**	**Number of Individuals**	**Mean Coverage**	**Collection Location**	**Project Number**
Shuja horei	M	1	7.91	Toby's Place	PRJNA550295
Shuja horei	F	1	8.26	Toby's Place	PRJNA550295
*Shuja horei**	M	25	41.76	Kalambo Lodge, Zambia	PRJNA802233
*Shuja horei**	F	25	49.82	Kalambo Lodge, Zambia	PRJNA802233
Gnathochromis pfefferi	M	1	7.49	Toby's Place	PRJNA550295
Gnathochromis pfefferi	F	1	7.24	Toby's Place	PRJNA550295
*Gnathochromis pfefferi**	M	23	44.27	Kalambo Lodge, Zambia	PRJNA802233
*Gnathochromis pfefferi**	F	25	45.94	Kalambo Lodge, Zambia	PRJNA802233
Interochromis loocki	M	1	7.22	Toby's Place	PRJNA550295
Interochromis loocki	F	1	9.08	Toby's Place	PRJNA550295
Interochromis loocki[+]	M	3	NA	Toby's Place	PRJNA552202
Interochromis loocki[+]	F	3	NA	Toby's Place	PRJNA552202
*Interochromis loocki**	M	24	41.76	Kalambo Lodge, Zambia	PRJNA802233
*Interochromis loocki**	F	21	41.33	Kalambo Lodge, Zambia	PRJNA802233
Limnotilapia dardennii	M	1	7.99	Toby's Place	PRJNA550295
Limnotilapia dardennii	F	1	7.84	Toby's Place	PRJNA550295
Lobochilotes labiatus	M	1	7.57	Toby's Place	PRJNA550295
Lobochilotes labiatus	F	1	6.55	Toby's Place	PRJNA550295
Lobochilotes labiatus[+]	M	3	NA	Toby's Place	PRJNA552202
Lobochilotes labiatus[+]	F	3	NA	Toby's Place	PRJNA552202
Petrochromis famula	M	1	8.21	Toby's Place	PRJNA550295
Petrochromis famula	F	1	5.92	Toby's Place	PRJNA550295
Petrochromis famula[+]	M	3	NA	Toby's Place	PRJNA552202
Petrochromis famula[+]	F	3	NA	Toby's Place	PRJNA552202
Petrochromis fasciolatus	M	1	8.00	Toby's Place	PRJNA550295
Petrochromis fasciolatus	F	1	7.93	Toby's Place	PRJNA550295
Petrochromis fasciolatus[+]	M	3	NA	Toby's Place	PRJNA552202
Petrochromis fasciolatus[+]	F	3	NA	Toby's Place	PRJNA552202
Petrochromis horii	M	1	7.38	Katete 3	PRJNA550295
Petrochromis horii	F	1	9.71	Katete 3	PRJNA550295
Petrochromis macrognathus	M	1	7.11	Chituta	PRJNA550295
Petrochromis macrognathus	F	1	6.33	Chituta	PRJNA550295

Petrochromis macrognathus[+]	M	3	NA	Chituta	PRJNA552202
Petrochromis macrognathus[+]	F	3	NA	Chituta	PRJNA552202
Petrochromis orthognathus	M	1	12.62	Nyaruhongoka 2	PRJNA550295
Petrochromis orthognathus	F	1	4.17	Nyaruhongoka 2	PRJNA550295
Petrochromis polyodon	M	1	7.76	Toby's Place	PRJNA550295
Petrochromis polyodon	F	1	7.83	Toby's Place	PRJNA550295
Petrochromis polyodon[+]	M	3	NA	Toby's Place	PRJNA552202
Petrochromis polyodon[+]	F	3	NA	Toby's Place	PRJNA552202
Petrochromis sp. giant	M	1	8.22	Fulwe	PRJNA550295
Petrochromis sp. giant	F	1	7.60	Fulwe	PRJNA550295
Petrochromis sp. kazumbae	M	1	8.63	Kaku	PRJNA550295
Petrochromis sp. kazumbae	F	1	8.39	Kaku	PRJNA550295
Petrochromis sp. kipili brown	M	1	6.50	Fulwe	PRJNA550295
Petrochromis sp. kipili brown	F	1	7.46	Fulwe	PRJNA550295
Petrochromis sp. macrognathus rainbow	M	1	7.50	Nkondwe	PRJNA550295
Petrochromis sp. macrognathus rainbow	F	1	7.58	Nkondwe	PRJNA550295
Petrochromis sp. moshi yellow	M	1	7.81	Nganja	PRJNA550295
Petrochromis sp. moshi yellow	F	1	7.27	Nganja	PRJNA550295
Petrochromis sp. orthognathus Ikola	M	1	7.40	Korongwe	PRJNA550295
Petrochromis sp. orthognathus Ikola	F	1	7.69	Korongwe	PRJNA550295
Petrochromis sp. polyodon Texas	M	1	8.12	Twiyu	PRJNA550295
Petrochromis sp. polyodon Texas	F	1	9.99	Twiyu	PRJNA550295
Petrochromis sp. red	M	1	6.50	Kalila Nkwasi	PRJNA550295
Petrochromis sp. red	F	1	6.53	Kalila Nkwasi	PRJNA550295
Petrochromis trewavasae	M	1	14.43	Katete 2	PRJNA550295
Petrochromis trewavasae	F	1	8.34	Katete 2	PRJNA550295
Petrochromis trewavasae ephippium	M	1	8.21	Toby's Place	PRJNA550295
Petrochromis trewavasae ephippium	F	1	7.44	Toby's Place	PRJNA550295
Petrochromis trewavasae ephippium[+]	M	3	NA	Toby's Place	PRJNA552202

Petrochromis trewavasae ephippium+	F	3	NA	Toby's Place	PRJNA552202
Pseudosimochromis babaulti	M	1	8.28	Kitaza south	PRJNA550295
Pseudosimochromis babaulti	F	1	12.68	Kitaza south	PRJNA550295
*Pseudosimochromis babaulti**	M	25	46.21	Kalambo Lodge, Zambia	PRJNA802233
*Pseudosimochromis babaulti**	F	23	41.16	Kalambo Lodge, Zambia	PRJNA802233
Pseudosimochromis babaulti South	M	1	9.64	Toby's Place	PRJNA550295
Pseudosimochromis babaulti South	F	1	9.62	Toby's Place	PRJNA550295
Pseudosimochromis curvifrons	M	1	9.90	Toby's Place	PRJNA550295
Pseudosimochromis curvifrons	F	1	9.07	Toby's Place	PRJNA550295
Pseudosimochromis marginatus	M	1	9.90	Nyanza Lac	PRJNA550295
Pseudosimochromis marginatus	F	1	15.20	Nyanza Lac	PRJNA550295
Pseudosimochromis marginatus North	M	1	7.26	Tembo Rock	PRJNA550295
Pseudosimochromis marginatus North	F	1	7.66	Tembo Rock	PRJNA550295
Simochromis diagramma	M	1	9.59	Toby's Place	PRJNA550295
Simochromis diagramma	F	1	10.21	Toby's Place	PRJNA550295
Simochromis diagramma+	M	3	NA	Toby's Place	PRJNA552202
Simochromis diagramma+	F	3	NA	Toby's Place	PRJNA552202
*Simochromis diagramma**	M	20	47.08	Captive full-sib	PRJNA802233
*Simochromis diagramma**	F	28	38.93	Captive full-sib	PRJNA802233
Tropheus annectens	M	1	14.12	Mukamba	PRJNA550295
Tropheus annectens	F	1	7.94	Mukamba	PRJNA550295
Tropheus brichardi	M	1	8.73	Nyanza Lac	PRJNA550295
Tropheus brichardi	F	1	8.69	Nyanza Lac	PRJNA550295
Tropheus duboisi	M	1	7.66	Kaku	PRJNA550295
Tropheus duboisi	F	1	8.19	Kaku	PRJNA550295
Tropheus moorii	M	1	6.83	Mbita Island W	PRJNA550295
Tropheus moorii	F	1	7.86	Mbita Island W	PRJNA550295
Tropheus moorii+	M	3	NA	Mbita Island W	PRJNA552202
Tropheus moorii+	F	3	NA	Mbita Island W	PRJNA552202
Tropheus polli	M	1	11.96	Storo 1	PRJNA550295
Tropheus polli	F	1	11.86	Storo 1	PRJNA550295
Tropheus sp. black	M	1	8.52	Nyaruhongoka	PRJNA550295
Tropheus sp. black	F	1	10.56	Nyaruhongoka	PRJNA550295

Tropheus sp. black*	M	30	12.03	Ikola, Tanzania	PRJNA400462
Tropheus sp. black*	F	24	12.88	Ikola, Tanzania	PRJNA400462
Tropheus sp. brichardi kipili	M	1	7.41	Nkondwe	PRJNA550295
Tropheus sp. brichardi kipili	F	1	7.97	Nkondwe	PRJNA550295
Tropheus sp. kirschfleck	M	1	7.38	Nganja	PRJNA550295
Tropheus sp. kirschfleck	F	1	6.73	Nganja	PRJNA550295
Tropheus sp. lukuga	M	1	11.91	Katumbi	PRJNA550295
Tropheus sp. lukuga	F	1	7.40	Katumbi	PRJNA550295
Tropheus sp. lunatus	M	1	7.51	Cape Kabogo	PRJNA550295
Tropheus sp. lunatus	F	1	7.99	Cape Kabogo	PRJNA550295
Tropheus sp. mpimbwe	M	1	7.17	Korongwe	PRJNA550295
Tropheus sp. mpimbwe	F	1	6.42	Korongwe	PRJNA550295
Tropheus sp. murago	M	1	7.74	Wapembwe	PRJNA550295
Tropheus sp. murago	F	1	7.45	Wapembwe	PRJNA550295
Tropheus sp. red	M	1	9.13	Chimba	PRJNA550295
Tropheus sp. red	F	1	7.69	Chimba	PRJNA550295

* pooled, + transcriptome

Supplemental Table 2. Compilation of all statistical results by species. Each species table contains all results obtained for that species and is divided into sub-categories. The sex-specific SNP test section designates calls made by visually inspecting the F_{ST} and allele frequency plots and was the first stage of our analysis. Blue cell fill indicates an XY signal, pink indicates a ZW signal, and purple indicates the presence of both XY and ZW signal. If transcriptome and/or pooled sequencing data for a species was available, the cells in these rows will not be blacked out. Following the sex-specific SNP test, the Kruskal-Wallis test was conducted to determine if the $\log_2$(XY:ZW) was significantly different between chromosomes, with a p-value cutoff of 0.05. If that test was significant, a post-hoc Dunn's test with correction for multiple testing was then conducted (ns = no significance, * = 0.05, ** = 0.01, *** = 0.001, **** = 0.00001). After this, the Top 1% (# of windows per chromosome) was calculated, taking the top ~78 of 7800 windows, and the table contains the number of XY and ZW windows per chromosome that meet that criteria. Finally, mean per chromosome $\log_2$(XY:ZW) was calculated.

Tropheus duboisi		LG01	LG02	LG03	LG04	LG05	LG06	LG07	LG08	LG09	LG10	LG11	LG12	LG13	LG14	LG15	LG16	LG17	LG18	LG19	LG20	LG22	LG23
**Sex-specific SNP inspection**																							
Individual	1M, 1F																						
Transcriptome		[redacted]																					
Pooled		[redacted]																					
**Kruskal-Wallis for heterogeneity among chromosomes, df = 21**		[redacted]																					
	1M, 1F	[redacted]																					
	chi-squared = 157.65, p-value < 2.2e-16	[redacted]																					
**Top 1% (# windows per chromosome)**																							
	1M, 1F																						
**XY**		0	1	20	1	0	1	2	1	4	1	0	0	2	1	0	2	0	0	2	0	7	2
**ZW**		0	2	10	2	0	0	0	0	0	4	0	0	0	0	1	0	0	3	1	0	7	1
**mean per chromosome log2(XY:ZW) ratio of SNP density**																							
	1M, 1F	-0.13043767	-0.02098196	0.04189163	-0.20170328	-0.18490863	-0.20131224	0.03655278	0.00635136	-1.30473234	-0.39428705	-0.02148442	-0.21377555	-0.45555536	0.06076278	-0.11539525	0.10216061	-0.06455976	0.00950557	-0.12422104	-2.01090796	0.05821307	-0.31040839
**Dunn's test to identifiy outlier chromosomes**	1M, 1F																						
	LG01																						
	LG02	ns																					
	LG03	ns	ns																				
	LG04	ns	ns	ns																			
	LG05	ns	ns	ns	ns																		
	LG06	ns	ns	ns	ns	ns																	
	LG07	ns	ns	ns	ns	ns	ns																
	LG08	ns	ns	ns	ns	ns	ns	ns															
	LG09	****	****	****	****	****	****	****	****														
	LG10	ns	*	**	ns	ns	ns	***	*	****													
	LG11	ns	ns	ns	ns	ns	ns	ns	ns	****	ns												
	LG12	ns	ns	ns	ns	ns	ns	ns	ns	****	ns	ns											
	LG13	ns	*	**	ns	ns	ns	***	*	****	ns	*	ns										
	LG14	ns	ns	ns	ns	ns	ns	ns	ns	****	**	ns	ns	**									
	LG15	ns	ns	ns	ns	ns	ns	ns	ns	****	ns	ns	ns	ns	ns								
	LG16	ns	ns	ns	ns	ns	*	ns	ns	****	***	ns	ns	***	ns	ns							
	LG17	ns	ns	ns	ns	ns	ns	ns	ns	****	*	ns	ns	*	ns	ns	ns						
	LG18	ns	ns	ns	ns	ns	ns	ns	ns	****	*	ns	ns	*	ns	ns	ns	ns					
	LG19	ns	ns	ns	ns	ns	ns	ns	ns	****	ns	ns	ns	ns	ns	ns	ns	ns	ns				
	LG20	ns	ns	ns	ns	ns	ns	ns	ns	****	ns	ns	ns	ns	ns	ns	ns	ns	ns	ns			
	LG22	ns	ns	ns	ns	ns	ns	ns	ns	****	**	ns	ns	**	ns	ns	ns	ns	ns	ns	ns		
	LG23	ns	ns	*	ns	ns	ns	**	ns	****	ns	ns	ns	ns	*	ns	**	ns	ns	ns	ns	*	

Tropheus sp. 'black'		LG01	LG02	LG03	LG04	LG05	LG06	LG07	LG08	LG09	LG10	LG11	LG12	LG13	LG14	LG15	LG16	LG17	LG18	LG19	LG20	LG22	LG23
**Sex-specific SNP inspection**																							
Individual	1M, 1F																						
Transcriptome																							
Pooled	20M, 24F																						
**Kruskal-Wallis for heterogeneity among chromosomes, df = 21**																							
	1M, 1F																						
	chi-squared = 429.45, p-value < 2.2e-16																						
	20M, 24F																						
	chi-squared = 439.16, p-value < 2.2e-16																						
**(# windows per chromosome)**																							
	1M, 1F																						
XY		0	0	0	0	0	0	0	0	0	0	0	0	0	0	0	0	0	0	77	0	0	0
ZW		0	0	0	0	0	0	0	0	0	0	0	0	0	0	0	0	0	0	0	0	0	0
	20M, 24F																						
XY		0	0	0	0	0	0	0	0	0	0	0	0	0	0	0	0	0	0	78	0	0	0
ZW		0	0	0	0	0	0	0	0	0	0	0	0	0	0	0	0	0	0	0	0	0	0
**mean per chromosome log2(XY:ZW) ratio of SNP density**																							
	1M, 1F	-0.08554637	0.17234594	0.04418953	0.25320214	-0.16731544	-0.09357618	0.16057239	-0.32166867	-0.07831161	0.02813864	0.22868056	-0.07860554	-0.09772872	-0.16054374	-0.1771118	-0.08682522	-0.05911453	-0.5471318	4.30140868	-0.05447771	0.09889549	0.12710707
	20M, 24F	-0.02992809	-0.01284525	-0.15409282	0.0771982	-0.02536538	-0.00094101	0.0014581	0.0203157	-0.06343691	-0.0080497	0.05016465	-0.15639729	-0.03779538	-0.04243594	-0.01090799	-0.00940034	0.05682711	-0.14350464	4.57360258	-0.02330661	-0.00485796	-0.04942742
**Dunn's test to identifiy outlier chromosomes**	1M, 1F																						
	LG01																						
	LG02	ns																					
	LG03	ns	ns																				
	LG04	ns	ns	ns																			
	LG05	ns	*	ns	**																		
	LG06	ns	ns	ns	*	ns																	
	LG07	ns	ns	ns	ns	*	ns																
	LG08	ns	**	ns	**	ns	ns	**															
	LG09	ns	ns	ns	ns	ns	ns	ns	ns														
	LG10	ns	ns	ns	ns	ns	ns	ns	ns	ns													
	LG11	ns	ns	ns	ns	**	*	ns	**	ns	ns												
	LG12	ns	ns	ns	ns	ns	ns	ns	ns	ns	ns	ns											
	LG13	ns	ns	ns	ns	ns	ns	ns	ns	ns	ns	ns	ns										
	LG14	ns	*	ns	*	ns	ns	*	ns	ns	ns	*	ns	ns									
	LG15	ns	*	ns	**	ns	ns	*	ns	ns	ns	**	ns	ns	ns								
	LG16	ns	ns	ns	*	ns	ns	ns	ns	ns	ns	*	ns	ns	ns	ns							
	LG17	ns	ns	ns	ns	ns	ns	ns	ns	ns	ns	ns	ns	ns	ns	ns	ns						
	LG18	*	****	**	****	ns	*	****	ns	*	**	****	*	*	ns	ns	*	*					
	LG19	****	****	****	****	****	****	****	****	****	****	****	****	****	****	****	****	****	****				
	LG20	ns	ns	ns	ns	**	*	ns	**	ns	ns	ns	ns	ns	*	**	*	ns	****	****			
	LG22	ns	ns	ns	*	ns	ns	ns	ns	ns	ns	*	ns	ns	ns	ns	ns	ns	*	****	*		
	LG23	ns	ns	ns	ns	ns	ns	ns	*	ns	ns	ns	ns	ns	ns	ns	ns	ns	***	****	ns	ns	
**Dunn's test to identifiy outlier chromosomes**	20M, 24F																						
	LG01																						
	LG02	ns																					
	LG03	ns	ns																				
	LG04	ns	ns	ns																			
	LG05	ns	ns	ns	ns																		
	LG06	ns	ns	ns	ns	ns																	
	LG07	ns	ns	ns	ns	ns	ns																
	LG08	ns	ns	ns	ns	ns	ns	ns															
	LG09	ns	ns	ns	ns	ns	ns	ns	ns														
	LG10	ns	ns	ns	ns	ns	ns	ns	ns	ns													
	LG11	ns	ns	ns	ns	ns	ns	ns	ns	ns	ns												
	LG12	ns	ns	ns	ns	ns	ns	ns	ns	ns	ns	ns											
	LG13	ns	ns	ns	ns	ns	ns	ns	ns	ns	ns	ns	ns										
	LG14	ns	ns	ns	ns	ns	ns	ns	ns	ns	ns	ns	ns	ns									
	LG15	ns	ns	ns	ns	ns	ns	ns	ns	ns	ns	ns	ns	ns	ns								
	LG16	ns	ns	ns	ns	ns	ns	ns	ns	ns	ns	ns	ns	ns	ns	ns							
	LG17	ns	ns	ns	ns	ns	ns	ns	ns	ns	ns	ns	ns	ns	ns	ns	ns						
	LG18	ns	ns	ns	ns	ns	ns	ns	ns	ns	ns	ns	ns	ns	ns	ns	ns	ns					
	LG19	****	****	****	****	****	****	****	****	****	****	****	****	****	****	****	****	****	****				
	LG20	ns	ns	ns	ns	ns	ns	ns	ns	ns	ns	ns	ns	ns	ns	ns	ns	ns	ns	****			
	LG22	ns	ns	ns	ns	ns	ns	ns	ns	ns	ns	ns	ns	ns	ns	ns	ns	ns	ns	****	ns		
	LG23	ns	ns	ns	ns	ns	ns	ns	ns	ns	ns	ns	ns	ns	ns	ns	ns	ns	ns	****	ns	ns	

Tropheus sp. 'kirschfleck'		LG01	LG02	LG03	LG04	LG05	LG06	LG07	LG08	LG09	LG10	LG11	LG12	LG13	LG14	LG15	LG16	LG17	LG18	LG19	LG20	LG22	LG23
**Sex-specific SNP inspection**																							
Individual	1M, 1F																						
Transcriptome		[redacted]																					
Pooled																							
**Kruskal-Wallis for heterogeneity among chromosomes, df = 21**																							
	1M, 1F																						
	chi-squared = 542.35, p-value < 2.2e-16																						
**Top 1% (# windows per chromosome)**																							
	1M, 1F																						
XY		0	0	0	0	0	1	0	0	0	0	0	0	0	0	0	0	0	0	79	0	0	0
ZW		0	0	0	0	0	0	0	0	0	0	0	0	0	0	0	0	0	0	0	0	0	1
**mean per chromosome log2(XY:ZW) ratio of SNP density**																							
	1M, 1F	-0.36236962	0.02216785	-0.05554885	-0.00831248	0.01167358	-0.05548058	-0.03469078	-0.25245795	-0.06675745	-0.21441717	0.00088941	-0.25125035	0.14640852	-0.05505821	0.08947661	0.16187534	-0.08489218	-0.0170177	3.27606044	-0.55829555	-0.51259759	0.01761948
**Dunn's test to identifiy outlier chromosomes**	1M, 1F																						
	LG01																						
	LG02	**																					
	LG03	**	ns																				
	LG04	**	ns	ns																			
	LG05	**	ns	ns	ns																		
	LG06	*	ns	ns	ns	ns																	
	LG07	**	ns	ns	ns	ns	ns																
	LG08	ns	ns	ns	ns	ns	ns	ns															
	LG09	ns	ns	ns	ns	ns	ns	ns	ns														
	LG10	ns	ns	ns	ns	ns	ns	ns	ns	ns													
	LG11	**	ns	ns	ns	ns	ns	ns	ns	ns	ns												
	LG12	ns	ns	ns	ns	ns	ns	ns	ns	ns	ns	ns											
	LG13	****	ns	ns	ns	ns	ns	ns	**	ns	**	ns	**										
	LG14	**	ns	ns	ns	ns	ns	ns	ns	ns	ns	ns	ns	ns									
	LG15	***	ns	ns	ns	ns	ns	ns	ns	ns	*	ns	*	ns	ns								
	LG16	****	ns	ns	ns	ns	ns	ns	**	ns	**	ns	**	ns	ns	ns							
	LG17	*	ns	ns	ns	ns	ns	ns	ns	ns	ns	ns	ns	ns	ns	ns	ns						
	LG18	**	ns	ns	ns	ns	ns	ns	ns	ns	ns	ns	ns	ns	ns	ns	ns	ns					
	LG19	****	****	****	****	****	****	****	****	****	****	****	****	****	****	****	****	****	****				
	LG20	****	****	****	****	****	****	****	****	****	****	****	****	****	****	****	****	****	****	****			
	LG22	ns	ns	ns	ns	ns	ns	ns	ns	ns	ns	ns	ns	ns	ns	ns	*	ns	ns	****	****		
	LG23	***	ns	ns	ns	ns	ns	ns	ns	ns	*	ns	*	ns	ns	ns	ns	ns	ns	****	****	ns	

Tropheus sp. 'lunatus'		LG01	LG02	LG03	LG04	LG05	LG06	LG07	LG08	LG09	LG10	LG11	LG12	LG13	LG14	LG15	LG16	LG17	LG18	LG19	LG20	LG22	LG23
**Sex-specific SNP inspection**																							
Individual	1M, 1F																						
Transcriptome		[redacted]																					
Pooled																							
**Kruskal-Wallis for heterogeneity among chromosomes, df = 21**																							
	1M, 1F																						
	chi-squared = 411.59, p-value < 2.2e-16																						
**Top 1% (# windows per chromosome)**																							
	1M, 1F																						
XY		0	0	0	0	0	0	0	0	0	0	0	0	0	0	0	0	0	0	76	0	0	0
ZW		0	0	1	0	0	0	0	0	0	0	0	0	0	0	0	0	0	0	0	0	0	0
**mean per chromosome log2(XY:ZW) ratio of SNP density**																							
	1M, 1F	-0.11760924	-0.00849748	0.071281766	-0.09513584	-0.08573585	-0.07282748	-0.05208581	-0.09626601	0.028103627	-0.16018053	-0.08939633	-0.02076291	-0.09972503	-0.08895636	-0.19202782	-0.03751748	-0.06586512	-0.10185343	3.298098983	0.006038715	-0.03297755	-0.11605454
**Dunn's test to identifiy outlier chromosomes**	1M, 1F																						
	LG01																						
	LG02	ns																					
	LG03	ns	ns																				
	LG04	ns	ns	ns																			
	LG05	ns	ns	ns	ns																		
	LG06	ns	ns	ns	ns	ns																	
	LG07	ns	ns	ns	ns	ns	ns																
	LG08	ns	ns	ns	ns	ns	ns	ns															
	LG09	ns	ns	ns	ns	ns	ns	ns	ns														
	LG10	ns	ns	ns	ns	ns	ns	ns	ns	ns													
	LG11	ns	ns	ns	ns	ns	ns	ns	ns	ns	ns												
	LG12	ns	ns	ns	ns	ns	ns	ns	ns	ns	ns	ns											
	LG13	ns	ns	ns	ns	ns	ns	ns	ns	ns	ns	ns	ns										
	LG14	ns	ns	ns	ns	ns	ns	ns	ns	ns	ns	ns	ns	ns									
	LG15	ns	ns	ns	ns	ns	ns	ns	ns	ns	ns	ns	ns	ns	ns								
	LG16	ns	ns	ns	ns	ns	ns	ns	ns	ns	ns	ns	ns	ns	ns	ns							
	LG17	ns	ns	ns	ns	ns	ns	ns	ns	ns	ns	ns	ns	ns	ns	ns	ns						
	LG18	ns	ns	ns	ns	ns	ns	ns	ns	ns	ns	ns	ns	ns	ns	ns	ns	ns					
	LG19	****	****	****	****	****	****	****	****	****	****	****	****	****	****	****	****	****	****				
	LG20	ns	ns	ns	ns	ns	ns	ns	ns	ns	ns	ns	ns	ns	ns	ns	ns	ns	ns	****			
	LG22	ns	ns	ns	ns	ns	ns	ns	ns	ns	ns	ns	ns	ns	ns	ns	ns	ns	ns	****	ns		
	LG23	ns	ns	ns	ns	ns	ns	ns	ns	ns	ns	ns	ns	ns	ns	ns	ns	ns	ns	****	ns	ns	

Tropheus sp. 'brichardi kipili'		**LG01**	**LG02**	**LG03**	**LG04**	**LG05**	**LG06**	**LG07**	**LG08**	**LG09**	**LG10**	**LG11**	**LG12**	**LG13**	**LG14**	**LG15**	**LG16**	**LG17**	**LG18**	**LG19**	**LG20**	**LG22**	**LG23**
**Sex-specific SNP inspection**																							
Individual	1M, 1F																						
Transcriptome																							
Pooled																							
**Kruskal-Wallis for heterogeneity among chromosomes, df = 21**																							
	1M, 1F																						
	chi-squared = 26.572, p-value < 0.1855																						
**Top 1% (# windows per chromosome)**																							
	1M, 1F																						
XY		0	2	12	2	1	1	1	0	1	1	2	1	1	0	4	1	0	1	1	0	6	4
ZW		0	3	10	2	1	0	0	0	0	0	1	0	1	0	2	1	0	4	1	0	4	1
**mean per chromosome log2(XY:ZW) ratio of SNP density**																							
	1M, 1F	0.04494021	-0.08239958	-0.18545075	0.00929338	0.12157545	0.0608576	-0.00219919	-0.05833592	0.12669884	-0.01107006	0.02021002	0.09715911	0.05263295	0.12646693	-0.10292322	-0.02895293	0.05160238	0.01865813	0.03698563	0.42677466	-0.04702307	-0.05983891

Tropheus sp. 'lukuga'		LG01	LG02	LG03	LG04	LG05	LG06	LG07	LG08	LG09	LG10	LG11	LG12	LG13	LG14	LG15	LG16	LG17	LG18	LG19	LG20	LG22	LG23
**Sex-specific SNP inspection**																							
Individual	1M, 1F																						
Transcriptome		[redacted]																					
Pooled																							
**Kruskal-Wallis for heterogeneity among chromosomes, df = 21**																							
	1M, 1F																						
	chi-squared = 413.56, p-value < 2.2e-16																						
**Top 1% (# windows per chromosome)**																							
	1M, 1F																						
XY		0	0	0	0	0	0	0	0	0	0	0	0	0	0	0	0	0	0	77	0	0	0
ZW		0	0	0	0	0	0	0	0	0	0	0	0	0	0	0	0	0	0	0	0	0	0
**mean per chromosome log2(XY:ZW) ratio of SNP density**																							
	1M, 1F	-0.2143055	0.12839056	-0.04183341	-0.29444731	-0.13972654	0.11880139	-0.16763066	0.13471252	-0.1665919	0.06463222	-0.10280812	0.18244299	0.03713607	0.09476374	-0.08301923	0.28809133	0.37658382	-0.00837791	3.51481511	0.02531723	0.06968185	-0.10784541
**Dunn's test to identifiy outlier chromosomes**	1M, 1F																						
	LG01																						
	LG02	ns																					
	LG03	ns	ns																				
	LG04	ns	**	ns																			
	LG05	ns	ns	ns	ns																		
	LG06	ns	ns	ns	*	ns																	
	LG07	ns	ns	ns	ns	ns	ns																
	LG08	ns	ns	ns	*	ns	ns	ns															
	LG09	ns	ns	ns	ns	ns	ns	ns	ns														
	LG10	ns	ns	ns	ns	ns	ns	ns	ns	ns													
	LG11	ns	ns	ns	ns	ns	ns	ns	ns	ns	ns												
	LG12	*	ns	ns	**	*	ns	*	ns	ns	ns	ns											
	LG13	ns	ns	ns	*	ns	ns	ns	ns	ns	ns	ns	ns										
	LG14	ns	ns	ns	*	ns	ns	ns	ns	ns	ns	ns	ns	ns									
	LG15	ns	ns	ns	ns	ns	ns	ns	ns	ns	ns	ns	ns	ns	ns								
	LG16	*	ns	ns	***	*	ns	*	ns	ns	ns	*	ns	ns	ns	ns							
	LG17	**	ns	*	****	**	ns	***	ns	*	ns	**	ns	ns	ns	*	ns						
	LG18	ns	ns	ns	ns	ns	ns	ns	ns	ns	ns	ns	ns	ns	ns	ns	ns	*					
	LG19	****	****	****	****	****	****	****	****	****	****	****	****	****	****	****	****	****	****				
	LG20	*	ns	ns	**	ns	ns	*	ns	ns	ns	ns	ns	ns	ns	ns	ns	ns	ns	****			
	LG22	ns	ns	ns	ns	ns	ns	ns	ns	ns	ns	ns	ns	ns	ns	ns	ns	*	ns	****	ns		
	LG23	ns	ns	ns	ns	ns	ns	ns	ns	ns	ns	ns	ns	ns	ns	ns	ns	*	ns	****	ns	ns	

Tropheus brichardi		LG01	LG02	LG03	LG04	LG05	LG06	LG07	LG08	LG09	LG10	LG11	LG12	LG13	LG14	LG15	LG16	LG17	LG18	LG19	LG20	LG22	LG23
**Sex-specific SNP inspection**																							
Individual	1M, 1F																						
Transcriptome		[redacted]																					
Pooled																							
**Kruskal-Wallis for heterogeneity among chromosomes, df = 21**																							
	1M, 1F																						
	chi-squared = 412.4, p-value < 2.2e-16																						
**Top 1% (# windows per chromosome)**																							
	1M, 1F																						
XY		0	0	0	0	0	0	0	0	0	0	0	0	0	0	0	0	0	0	77	0	0	0
ZW		0	0	0	0	0	0	0	0	0	0	0	0	0	0	0	0	0	0	0	0	0	0
**mean per chromosome log2(XY:ZW) ratio of SNP density**																							
	1M, 1F	-0.04681607	-0.03537008	0.17096649	-0.10384815	0.05271512	-0.05216068	-0.17397936	0.02285354	X3046:X3256	0.16264172	0.02873602	-0.05661152	0.16491341	0.10286431	-0.21710568	-0.10420314	0.08498337	-0.06587749	4.18038301	1.04422684	0.03730409	0.01772586
**Dunn's test to identifiy outlier chromosomes**	1M, 1F																						
	LG01																						
	LG02	ns																					
	LG03	ns	ns																				
	LG04	ns	ns	ns																			
	LG05	ns	ns	ns	ns																		
	LG06	ns	ns	ns	ns	ns																	
	LG07	ns	ns	ns	ns	ns	ns																
	LG08	ns	ns	ns	ns	ns	ns	ns															
	LG09	ns	ns	ns	ns	ns	ns	ns	ns														
	LG10	ns	ns	ns	ns	ns	ns	*	ns	ns													
	LG11	ns	ns	ns	ns	ns	ns	ns	ns	ns	ns												
	LG12	ns	ns	ns	ns	ns	ns	ns	ns	ns	ns	ns											
	LG13	ns	ns	ns	ns	ns	ns	ns	ns	ns	ns	ns	ns										
	LG14	ns	ns	ns	ns	ns	ns	ns	ns	ns	ns	ns	ns	ns									
	LG15	ns	ns	ns	ns	ns	ns	ns	ns	ns	ns	ns	ns	ns	ns								
	LG16	ns	ns	ns	ns	ns	ns	ns	ns	ns	ns	ns	ns	ns	ns	ns							
	LG17	ns	ns	ns	ns	ns	ns	ns	ns	ns	ns	ns	ns	ns	ns	ns	ns						
	LG18	ns	ns	ns	ns	ns	ns	ns	ns	ns	ns	ns	ns	ns	ns	ns	ns	ns					
	LG19	****	****	****	****	****	****	****	****	****	****	****	****	****	****	****	****	****	****				
	LG20	ns	ns	ns	ns	ns	ns	ns	ns	ns	ns	ns	ns	ns	ns	ns	ns	ns	ns	****			
	LG22	ns	ns	ns	ns	ns	ns	*	ns	ns	ns	ns	ns	ns	ns	ns	ns	ns	ns	****	ns		
	LG23	ns	ns	ns	ns	ns	ns	ns	ns	ns	ns	ns	ns	ns	ns	ns	ns	ns	ns	****	ns	ns	

Tropheus sp. 'mpimbwe'		**LG01**	**LG02**	**LG03**	**LG04**	**LG05**	**LG06**	**LG07**	**LG08**	**LG09**	**LG10**	**LG11**	**LG12**	**LG13**	**LG14**	**LG15**	**LG16**	**LG17**	**LG18**	**LG19**	**LG20**	**LG22**	**LG23**
**Sex-specific SNP inspection**																							
Individual	1M, 1F																						
Transcriptome		[redacted]																					
Pooled																							
**Kruskal-Wallis for heterogeneity among chromosomes, df = 21**																							
	1M, 1F																						
	chi-squared = 56.554, p-value < 4.183e-05																						
**Top 1% (# windows per chromosome)**																							
	1M, 1F																						
XY		0	3	14	1	2	1	0	0	5	1	1	0	2	0	0	0	0	4	0	0	7	2
ZW		0	1	8	1	1	1	1	0	4	1	2	0	1	0	1	2	0	1	2	2	5	2
**mean per chromosome log2(XY:ZW) ratio of SNP density**																							
	1M, 1F	0.02344801	0.1373192	0.07163484	0.04317876	0.00855214	-0.06335241	-0.02990895	0.50912958	0.15468129	-0.19771176	-0.03518426	-0.03516045	0.10064381	-0.07162539	-0.01429424	-0.05072719	-0.00670325	0.14858622	0.1681207	-0.9163028	-0.13906688	-0.09576647
**Dunn's test to identifiy outlier chromosomes**	1M, 1F																						
	LG01																						
	LG02	ns																					
	LG03	ns	ns																				
	LG04	ns	ns	ns																			
	LG05	ns	ns	ns	ns																		
	LG06	ns	ns	ns	ns	ns																	
	LG07	ns	ns	ns	ns	ns	ns																
	LG08	*	ns	*	*	**	***	***															
	LG09	ns	ns	ns	ns	ns	ns	ns	ns														
	LG10	ns	ns	ns	ns	ns	ns	ns	****	ns													
	LG11	ns	ns	ns	ns	ns	ns	ns	**	ns	ns												
	LG12	ns	ns	ns	ns	ns	ns	ns	***	ns	ns	ns											
	LG13	ns	ns	ns	ns	ns	ns	ns	ns	ns	ns	ns	ns										
	LG14	ns	ns	ns	ns	ns	ns	ns	***	ns	ns	ns	ns	ns									
	LG15	ns	ns	ns	ns	ns	ns	ns	**	ns	ns	ns	ns	ns	ns								
	LG16	ns	ns	ns	ns	ns	ns	ns	***	ns	ns	ns	ns	ns	ns	ns							
	LG17	ns	ns	ns	ns	ns	ns	ns	**	ns	ns	ns	ns	ns	ns	ns	ns						
	LG18	ns	ns	ns	ns	ns	ns	ns	ns	ns	*	ns	ns	ns	ns	ns	ns	ns					
	LG19	ns	ns	ns	ns	ns	ns	ns	ns	ns	ns	ns	ns	ns	ns	ns	ns	ns	ns				
	LG20	ns	*	ns	ns	ns	ns	ns	****	ns	ns	ns	ns	ns	ns	ns	ns	ns	*	*			
	LG22	ns	ns	ns	ns	ns	ns	ns	***	ns	ns	ns	ns	ns	ns	ns	ns	ns	ns	ns	ns		
	LG23	ns	ns	ns	ns	ns	ns	ns	***	ns	ns	ns	ns	ns	ns	ns	ns	ns	ns	ns	ns	ns	

Tropheus annectens		LG01	LG02	LG03	LG04	LG05	LG06	LG07	LG08	LG09	LG10	LG11	LG12	LG13	LG14	LG15	LG16	LG17	LG18	LG19	LG20	LG22	LG23
**Sex-specific SNP inspection**																							
Individual	1M, 1F																						
Transcriptome		[redacted]																					
Pooled																							
**Kruskal-Wallis for heterogeneity among chromosomes, df = 21**																							
	1M, 1F																						
	chi-squared = 75.528, p-value < 4.449e-08																						
**Top 1% (# windows per chromosome)**																							
	1M, 1F																						
XY		0	4	15	1	0	1	0	0	1	2	0	0	3	0	0	0	0	1	0	0	4	1
ZW		0	2	12	1	0	2	0	1	3	1	2	2	1	2	0	1	0	0	0	2	7	6
**mean per chromosome log2(XY:ZW) ratio of SNP density**																							
	1M, 1F	0.02835635	-0.04977938	0.18539596	0.01330093	0.08219535	-0.04740918	0.0450097	0.01610488	-0.15552425	0.1606326	0.01165631	0.0066625	-0.03036763	-0.45302413	0.0644143	0.09194617	-0.06953547	0.05450422	0.11270231	-0.17890307	0.0194157	-0.49637699
**Dunn's test to identifiy outlier chromosomes**	1M, 1F																						
	LG01																						
	LG02	ns																					
	LG03	ns	ns																				
	LG04	ns	ns	ns																			
	LG05	ns	ns	ns	ns																		
	LG06	ns	ns	ns	ns	ns																	
	LG07	ns	ns	ns	ns	ns	ns																
	LG08	ns	ns	ns	ns	ns	ns	ns															
	LG09	ns	ns	*	ns	ns	ns	ns	ns														
	LG10	ns	ns	ns	ns	ns	ns	ns	ns	*													
	LG11	ns	ns	ns	ns	ns	ns	ns	ns	ns	ns												
	LG12	ns	ns	ns	ns	ns	ns	ns	ns	ns	ns	ns											
	LG13	ns	ns	ns	ns	ns	ns	ns	ns	ns	ns	ns	ns										
	LG14	**	ns	***	*	**	**	**	*	ns	***	*	**	**									
	LG15	ns	ns	ns	ns	ns	ns	ns	ns	ns	ns	ns	ns	ns	**								
	LG16	ns	ns	ns	ns	ns	ns	ns	ns	*	ns	ns	ns	ns	***	ns							
	LG17	ns	ns	ns	ns	ns	ns	ns	ns	ns	*	ns	ns	ns	ns	ns	ns						
	LG18	ns	ns	ns	ns	ns	ns	ns	ns	ns	ns	ns	ns	ns	**	ns	ns	ns					
	LG19	ns	ns	ns	ns	ns	ns	ns	ns	*	ns	ns	ns	ns	***	ns	ns	ns	ns				
	LG20	ns	ns	ns	ns	ns	ns	ns	ns	ns	ns	ns	ns	ns	*	ns	ns	ns	ns	ns			
	LG22	ns	ns	ns	ns	ns	ns	ns	ns	ns	ns	ns	ns	ns	*	ns	ns	ns	ns	ns	ns		
	LG23	***	*	****	**	***	**	***	**	ns	****	**	**	**	ns	***	***	*	***	***	**	**	

Tropheus polli		LG01	LG02	LG03	LG04	LG05	LG06	LG07	LG08	LG09	LG10	LG11	LG12	LG13	LG14	LG15	LG16	LG17	LG18	LG19	LG20	LG22	LG23
**Sex-specific SNP inspection**																							
Individual	1M, 1F																						
Transcriptome		[redacted]																					
Pooled																							
**Kruskal-Wallis for heterogeneity among chromosomes, df = 21**																							
	1M, 1F																						
	chi-squared = 586.57, p-value < 2.2e-16																						
**Top 1% (# windows per chromosome)**																							
	1M, 1F																						
XY		0	0	0	0	0	0	0	0	0	0	0	0	0	0	0	0	0	0	77	0	0	0
ZW		0	0	0	0	0	0	0	0	0	0	0	0	0	0	0	0	0	0	0	0	0	0
**mean per chromosome log2(XY:ZW) ratio of SNP density**																							
	1M, 1F	-0.38086296	0.34774249	-0.05130694	0.24548356	0.20572539	-0.2955881	-0.24803038	-0.05951587	-0.06599645	0.89656961	-0.0871825	0.14372845	-0.2943628	0.18996139	0.15418227	-0.01397598	-0.31858517	-0.39090596	4.78300631	0.78708452	0.01034192	0.01244775
**Dunn's test to identifiy outlier chromosomes**	1M, 1F																						
	LG01																						
	LG02	****																					
	LG03	*	ns																				
	LG04	****	ns	ns																			
	LG05	***	ns	ns	ns																		
	LG06	ns	***	ns	**	**																	
	LG07	ns	****	ns	***	**	ns																
	LG08	ns	*	ns	ns	ns	ns	ns															
	LG09	ns	*	ns	ns	ns	ns	ns	ns														
	LG10	****	*	****	*	**	****	****	****	****													
	LG11	ns	*	ns	*	ns	ns	ns	ns	ns	****												
	LG12	**	ns	ns	ns	ns	*	*	ns	ns	***	ns											
	LG13	ns	***	ns	**	**	ns	ns	ns	ns	****	ns	*										
	LG14	***	ns	ns	ns	ns	**	**	ns	ns	**	ns	ns	**									
	LG15	**	ns	ns	ns	ns	*	**	ns	ns	***	ns	ns	*	ns								
	LG16	*	*	ns	ns	ns	ns	ns	ns	ns	****	ns	ns	ns	ns	ns							
	LG17	ns	****	ns	***	**	ns	ns	ns	ns	****	ns	*	ns	***	**	ns						
	LG18	ns	****	*	***	**	ns	ns	ns	ns	****	ns	*	ns	***	**	ns	ns					
	LG19	****	****	****	****	****	****	****	****	****	****	****	****	****	****	****	****	****	****				
	LG20	ns	*	ns	ns	ns	ns	ns	ns	ns	****	ns	ns	ns	ns	ns	ns	ns	ns	****			
	LG22	**	ns	ns	ns	ns	*	**	ns	ns	***	ns	ns	*	ns	ns	ns	**	**	****	ns		
	LG23	*	ns	ns	ns	ns	ns	ns	ns	ns	****	ns	ns	ns	ns	ns	ns	*	*	****	ns	ns	

***Tropheus* sp. 'red'**		**LG01**	**LG02**	**LG03**	**LG04**	**LG05**	**LG06**	**LG07**	**LG08**	**LG09**	**LG10**	**LG11**	**LG12**	**LG13**	**LG14**	**LG15**	**LG16**	**LG17**	**LG18**	**LG19**	**LG20**	**LG22**	**LG23**
**Sex-specific SNP inspection**																							
Individual	1M, 1F																						
Transcriptome		[redacted]																					
Pooled																							
**Kruskal-Wallis for heterogeneity among chromosomes, df = 21**																							
	1M, 1F																						
	chi-squared = 29.825, p-value < 0.09556																						
**Top 1% (# windows per chromosome)**																							
	1M, 1F																						
XY		0	0	17	2	1	0	0	0	2	0	0	0	2	0	2	1	0	2	2	0	4	2
ZW		0	5	9	1	1	1	0	0	3	0	2	0	1	0	0	0	0	3	1	2	7	4
**mean per chromosome log2(XY:ZW) ratio of SNP density**																							
	1M, 1F	-0.00636948	-0.12564886	0.07918274	0.03011987	0.0288336	0.09595159	-0.03329331	0.02885024	-0.1156553	0.0899754	0.07079385	0.12299181	0.02014075	-0.02629346	0.06940708	0.14705282	-0.153233	-0.01640881	0.10896778	-0.71414048	0.0117198	-0.03002561

Tropheus sp. 'murago'		LG01	LG02	LG03	LG04	LG05	LG06	LG07	LG08	LG09	LG10	LG11	LG12	LG13	LG14	LG15	LG16	LG17	LG18	LG19	LG20	LG22	LG23
**Sex-specific SNP inspection**																							
Individual	1M, 1F																						
Transcriptome		[redacted]																					
Pooled																							
**Kruskal-Wallis for heterogeneity among chromosomes, df = 21**																							
	1M, 1F																						
	chi-squared = 26.287, p-value < 0.1957																						
**Top 1% (# windows per chromosome)**																							
	1M, 1F																						
XY		0	2	11	2	1	2	0	2	0	0	1	0	0	0	0	0	0	4	2	0	6	3
ZW		0	4	15	2	0	2	0	0	4	2	1	0	1	0	0	0	0	3	1	0	5	1
**mean per chromosome log2(XY:ZW) ratio of SNP density**																							
	1M, 1F	-0.04402759	-0.00862437	-0.13033366	0.03686251	0.0525695	0.07084778	0.00193155	0.00812197	0.00726261	0.05629503	0.06855637	0.24331918	0.04616279	-0.01223413	0.00155131	-0.07611394	0.04974705	0.20818476	0.04065562	0.38867777	0.08405907	-0.01657738

Tropheus moorii		LG01	LG02	LG03	LG04	LG05	LG06	LG07	LG08	LG09	LG10	LG11	LG12	LG13	LG14	LG15	LG16	LG17	LG18	LG19	LG20	LG22	LG23
**Sex-specific SNP inspection**																							
Individual	1M, 1F																						
Transcriptome	3M, 3F																						
Pooled																							
**Kruskal-Wallis for heterogeneity among chromosomes, df = 21**																							
	1M, 1F																						
	chi-squared = 37.011, p-value < 0.01679																						
**Top 1% (# windows per chromosome)**																							
	1M, 1F																						
XY		0	3	14	2	1	1	0	0	1	0	4	0	2	0	1	1	0	4	0	0	4	0
ZW		2	3	15	2	0	1	0	0	2	2	1	0	0	0	3	1	0	4	1	0	2	2
**mean per chromosome log2(XY:ZW) ratio of SNP density**																							
	1M, 1F	0.010211	0.23718476	0.03999953	-0.00629004	0.034174	-0.08098581	-0.03821988	0.05955687	-0.0966644	-0.14296136	0.03050964	-0.00078159	0.05345899	0.0226005	0.00626798	-0.04596585	-0.02445741	0.05763005	0.24484306	0.24618686	0.05854193	0.04392538
**Dunn's test to identifiy outlier chromosomes**	1M, 1F																						
	LG01																						
	LG02	ns																					
	LG03	ns	ns																				
	LG04	ns	ns	ns																			
	LG05	ns	ns	ns	ns																		
	LG06	ns	*	ns	ns	ns																	
	LG07	ns	*	ns	ns	ns	ns																
	LG08	ns	ns	ns	ns	ns	ns	ns															
	LG09	ns	ns	ns	ns	ns	ns	ns	ns														
	LG10	ns	**	ns	ns	ns	ns	ns	ns	ns													
	LG11	ns	ns	ns	ns	ns	ns	ns	ns	ns	ns												
	LG12	ns	ns	ns	ns	ns	ns	ns	ns	ns	ns	ns											
	LG13	ns	ns	ns	ns	ns	ns	ns	ns	ns	ns	ns	ns										
	LG14	ns	ns	ns	ns	ns	ns	ns	ns	ns	ns	ns	ns	ns									
	LG15	ns	ns	ns	ns	ns	ns	ns	ns	ns	ns	ns	ns	ns	ns								
	LG16	ns	*	ns	ns	ns	ns	ns	ns	ns	ns	ns	ns	ns	ns	ns							
	LG17	ns	ns	ns	ns	ns	ns	ns	ns	ns	ns	ns	ns	ns	ns	ns	ns						
	LG18	ns	ns	ns	ns	ns	ns	ns	ns	ns	ns	ns	ns	ns	ns	ns	ns	ns					
	LG19	ns	ns	ns	ns	ns	ns	ns	ns	ns	*	ns	ns	ns	ns	ns	ns	ns	ns				
	LG20	ns	ns	ns	ns	ns	ns	ns	ns	ns	ns	ns	ns	ns	ns	ns	ns	ns	ns	ns			
	LG22	ns	ns	ns	ns	ns	ns	ns	ns	ns	ns	ns	ns	ns	ns	ns	ns	ns	ns	ns	ns		
	LG23	ns	ns	ns	ns	ns	ns	ns	ns	ns	ns	ns	ns	ns	ns	ns	ns	ns	ns	ns	ns	ns	

Lobochilotes labiatus		LG01	LG02	LG03	LG04	LG05	LG06	LG07	LG08	LG09	LG10	LG11	LG12	LG13	LG14	LG15	LG16	LG17	LG18	LG19	LG20	LG22	LG23
**Sex-specific SNP inspection**																							
Individual	1M, 1F																						
Transcriptome	3M, 3F																						
Pooled		[redacted]																					
**Kruskal-Wallis for heterogeneity among chromosomes, df = 21**																							
	1M, 1F																						
	chi-squared = 83.671, p-value < 1.948e-09																						
**Top 1% (# windows per chromosome)**																							
	1M, 1F																						
XY		0	3	6	1	0	1	0	1	0	1	1	0	1	2	1	1	0	3	0	0	13	4
ZW		0	2	9	1	2	1	1	4	3	0	0	0	1	1	0	1	0	3	0	0	11	5
**mean per chromosome log2(XY:ZW) ratio of SNP density**																							
	1M, 1F	0.33716243	-0.10266981	-0.34981353	-0.04396188	0.06934207	-0.01488092	0.12288298	-0.30210001	-0.22192777	-0.02035397	-0.02863847	-0.17012823	-0.09123992	0.16338983	0.15582176	0.0558152	-0.06583842	0.04811598	-0.05925791	-0.26258058	0.01624466	0.20275207
**Dunn's test to identifiy outlier chromosomes**	1M, 1F																						
	LG01																						
	LG02	**																					
	LG03	****	ns																				
	LG04	**	ns	ns																			
	LG05	ns	ns	***	ns																		
	LG06	*	ns	**	ns	ns																	
	LG07	ns	ns	****	ns	ns	ns																
	LG08	****	ns	ns	ns	*	ns	*															
	LG09	**	ns	ns	ns	ns	ns	ns	ns														
	LG10	**	ns	*	ns	ns	ns	ns	ns	ns													
	LG11	**	ns	*	ns	ns	ns	ns	ns	ns	ns												
	LG12	***	ns	ns	ns	ns	ns	*	ns	ns	ns	ns											
	LG13	***	ns	ns	ns	ns	ns	ns	ns	ns	ns	ns	ns										
	LG14	ns	ns	***	ns	ns	ns	ns	*	ns	ns	ns	ns	ns									
	LG15	ns	ns	***	ns	ns	ns	ns	*	ns	ns	ns	ns	ns	ns								
	LG16	*	ns	**	ns	ns	ns	ns	ns	ns	ns	ns	ns	ns	ns	ns							
	LG17	**	ns	*	ns	ns	ns	ns	ns	ns	ns	ns	ns	ns	ns	ns	ns						
	LG18	ns	ns	**	ns	ns	ns	ns	ns	ns	ns	ns	ns	ns	ns	ns	ns	ns					
	LG19	*	ns	*	ns	ns	ns	ns	ns	ns	ns	ns	ns	ns	ns	ns	ns	ns	ns				
	LG20	**	ns	*	ns	ns	ns	ns	ns	ns	ns	ns	ns	ns	ns	ns	ns	ns	ns	ns			
	LG22	**	ns	*	ns	ns	ns	ns	ns	ns	ns	ns	ns	ns	ns	ns	ns	ns	ns	ns	ns		
	LG23	ns	*	****	*	ns	ns	ns	**	*	ns	ns	**	*	ns	ns	ns	*	ns	ns	ns	ns	

Petrochromis macrognathus		LG01	LG02	LG03	LG04	LG05	LG06	LG07	LG08	LG09	LG10	LG11	LG12	LG13	LG14	LG15	LG16	LG17	LG18	LG19	LG20	LG22	LG23
**Sex-specific SNP inspection**																							
Individual	1M, 1F																						
Transcriptome	3M, 3F																						
Pooled																							
**Kruskal-Wallis for heterogeneity among chromosomes, df = 21**																							
	1M, 1F																						
	chi-squared = 59.046, p-value < 1.779e-05																						
**Top 1% (# windows per chromosome)**																							
	1M, 1F																						
XY		0	0	18	1	0	1	1	0	1	0	6	1	0	0	0	0	0	0	0	0	8	1
ZW		0	1	17	1	0	3	0	0	4	0	9	1	0	1	0	1	1	0	0	1	6	0
**mean per chromosome log2(XY:ZW) ratio of SNP density**																							
	1M, 1F	-0.00117892	0.04784386	-0.31837818	-0.02615105	-0.02911445	-0.15078907	-0.08213356	0.06781083	0.01173948	-0.19455339	-0.29374759	-0.14533114	-0.05374122	-0.19205822	-0.20147545	-0.02804946	-0.17019403	-0.10658804	0.09398481	-0.21797456	0.00055363	-0.11926196
**Dunn's test to identifiy outlier chromosomes**	1M, 1F																						
	LG01																						
	LG02	ns																					
	LG03	*	**																				
	LG04	ns	ns	*																			
	LG05	ns	ns	*	ns																		
	LG06	ns	ns	ns	ns	ns																	
	LG07	ns	ns	*	ns	ns	ns																
	LG08	ns	ns	**	ns	ns	ns	ns															
	LG09	ns	ns	*	ns	ns	ns	ns	ns														
	LG10	ns	ns	ns	ns	ns	ns	ns	ns	ns													
	LG11	*	*	ns	ns	ns	ns	ns	*	ns	ns												
	LG12	ns	ns	ns	ns	ns	ns	ns	ns	ns	ns	ns											
	LG13	ns	ns	ns	ns	ns	ns	ns	ns	ns	ns	ns	ns										
	LG14	ns	ns	ns	ns	ns	ns	ns	ns	ns	ns	ns	ns	ns									
	LG15	ns	ns	ns	ns	ns	ns	ns	ns	ns	ns	ns	ns	ns	ns								
	LG16	ns	ns	*	ns	ns	ns	ns	ns	ns	ns	ns	ns	ns	ns	ns							
	LG17	ns	*	ns	ns	ns	ns	ns	ns	ns	ns	ns	ns	ns	ns	ns	ns						
	LG18	ns	ns	ns	ns	ns	ns	ns	ns	ns	ns	ns	ns	ns	ns	ns	ns	ns					
	LG19	ns	ns	**	ns	ns	ns	ns	ns	ns	*	*	ns	ns	*	*	ns	*	ns				
	LG20	ns	ns	ns	ns	ns	ns	ns	ns	ns	ns	ns	ns	ns	ns	ns	ns	ns	ns	ns			
	LG22	ns	ns	**	ns	ns	ns	ns	ns	ns	ns	*	ns	ns	*	*	ns	*	ns	ns	ns		
	LG23	ns	ns	ns	ns	ns	ns	ns	ns	ns	ns	ns	ns	ns	ns	ns	ns	ns	ns	ns	ns	ns	

Petrochromis trewavasae		LG01	LG02	LG03	LG04	LG05	LG06	LG07	LG08	LG09	LG10	LG11	LG12	LG13	LG14	LG15	LG16	LG17	LG18	LG19	LG20	LG22	LG23
**Sex-specific SNP inspection**																							
Individual	1M, 1F																						
Transcriptome		[redacted]																					
Pooled																							
**Kruskal-Wallis for heterogeneity among chromosomes, df = 21**																							
	1M, 1F																						
	chi-squared = 500.74, p-value < 2.2e-16																						
**Top 1% (# windows per chromosome)**																							
	1M, 1F																						
XY		0	1	3	0	30	0	0	0	0	0	0	0	0	0	0	0	0	0	39	0	1	0
ZW		0	0	0	1	0	0	0	0	0	0	0	0	0	0	0	0	0	0	0	0	0	2
**mean per chromosome log2(XY:ZW) ratio of SNP density**																							
	1M, 1F	-0.23506118	0.41100065	0.99139641	1.52889066	1.21630123	-0.35273927	0.02506788	-0.37445388	-0.16858851	-0.25094589	-0.13985383	-0.02682793	0.16719942	0.36532195	0.14048726	-0.12604281	-0.24767316	0.00695823	1.2478654	0.1833018	-0.063788	-0.34794287
**Dunn's test to identifiy outlier chromosomes**	1M, 1F																						
	LG01																						
	LG02	****																					
	LG03	****	***																				
	LG04	****	****	***																			
	LG05	****	**	ns	****																		
	LG06	ns	****	****	****	****																	
	LG07	ns	**	****	****	****	**																
	LG08	ns	****	****	****	****	ns	**															
	LG09	ns	**	****	****	****	ns	ns	ns														
	LG10	ns	****	****	****	****	ns	ns	ns	ns													
	LG11	ns	***	****	****	****	ns	ns	ns	ns	ns												
	LG12	ns	**	****	****	****	ns	ns	ns	ns	ns	ns											
	LG13	*	ns	****	****	****	***	ns	**	ns	**	ns	ns										
	LG14	****	ns	****	****	***	****	**	****	**	****	***	**	ns									
	LG15	**	ns	****	****	****	***	ns	***	ns	**	*	ns	ns	ns								
	LG16	ns	***	****	****	****	ns	ns	ns	ns	ns	ns	ns	*	***	*							
	LG17	ns	****	****	****	****	ns	ns	ns	ns	ns	ns	ns	**	****	**	ns						
	LG18	ns	*	****	****	****	*	ns	*	ns	ns	ns	ns	ns	*	ns	ns	ns					
	LG19	****	**	ns	***	ns	****	****	****	****	****	****	****	****	**	****	****	****	****				
	LG20	ns	*	****	****	****	**	ns	*	ns	ns	ns	ns	ns	*	ns	ns	ns	ns	****			
	LG22	ns	**	****	****	****	ns	ns	ns	ns	ns	ns	ns	ns	**	ns	ns	ns	ns	****	ns		
	LG23	ns	****	****	****	****	ns	*	ns	ns	ns	ns	ns	**	****	***	ns	ns	*	****	*	ns	

Petrochromis sp. 'kipili brown'		LG01	LG02	LG03	LG04	LG05	LG06	LG07	LG08	LG09	LG10	LG11	LG12	LG13	LG14	LG15	LG16	LG17	LG18	LG19	LG20	LG22	LG23
**Sex-specific SNP inspection**																							
Individual	1M, 1F																						
Transcriptome		[redacted]																					
Pooled		[redacted]																					
**Kruskal-Wallis for heterogeneity among chromosomes, df = 21**		[redacted]																					
	1M, 1F	[redacted]																					
	chi-squared = 1211, p-value < 2.2e-16	[redacted]																					
**Top 1% (# windows per chromosome)**																							
	1M, 1F																						
XY		1	1	4	14	0	0	0	0	1	0	9	0	1	0	0	0	1	2	4	12	17	0
ZW		0	0	2	0	0	1	0	0	1	1	1	0	0	1	2	0	0	1	0	0	2	1
**mean per chromosome log2(XY:ZW) ratio of SNP density**																							
	1M, 1F	1.64327574	0.04847143	0.36192776	1.04760426	0.68155563	-0.25694648	-0.02967323	-0.02478319	0.07373965	-0.028202	1.57115523	0.03760709	0.24705906	0.03647726	0.06283384	-0.14255226	0.80202554	0.0669544	0.62960159	0.35283762	1.70634441	-0.04001991
**Dunn's test to identifiy outlier chromosomes**	1M, 1F																						
	LG01																						
	LG02	****																					
	LG03	****	*																				
	LG04	****	****	****																			
	LG05	****	****	*	***																		
	LG06	****	*	****	****	****																	
	LG07	****	ns	***	****	****	ns																
	LG08	****	ns	**	****	****	ns	ns															
	LG09	****	ns	ns	****	***	*	ns	ns														
	LG10	****	ns	**	****	****	ns	ns	ns	ns													
	LG11	ns	****	****	***	****	****	****	****	****	****												
	LG12	****	ns	**	****	****	*	ns	ns	ns	ns	****											
	LG13	****	ns	ns	****	**	****	**	*	ns	*	****	*										
	LG14	****	ns	*	****	****	*	ns	ns	ns	ns	****	ns	ns									
	LG15	****	ns	*	****	****	*	ns	ns	ns	ns	****	ns	ns	ns								
	LG16	****	ns	****	****	****	ns	ns	ns	ns	ns	****	ns	***	ns	ns							
	LG17	****	****	**	*	ns	****	****	****	****	****	****	****	***	****	****	****						
	LG18	****	ns	*	****	****	*	ns	ns	ns	ns	****	ns	ns	ns	ns	ns	****					
	LG19	****	**	ns	****	ns	****	****	***	**	****	****	***	ns	***	**	****	ns	**				
	LG20	****	****	****	ns	*	****	****	****	****	****	****	****	****	****	****	****	ns	****	**			
	LG22	****	****	****	****	****	****	****	****	****	****	****	****	****	****	****	****	****	****	****	****		
	LG23	****	ns	***	****	****	ns	ns	ns	ns	ns	****	ns	*	ns	ns	ns	****	ns	****	****	****	

Petrochromis horii		LG01	LG02	LG03	LG04	LG05	LG06	LG07	LG08	LG09	LG10	LG11	LG12	LG13	LG14	LG15	LG16	LG17	LG18	LG19	LG20	LG22	LG23
**Sex-specific SNP inspection**																							
Individual	1M, 1F																						
Transcriptome		[redacted]																					
Pooled																							
**Kruskal-Wallis for heterogeneity among chromosomes, df = 21**																							
	1M, 1F																						
	chi-squared = 579.25, p-value < 2.2e-16																						
**Top 1% (# windows per chromosome)**																							
	1M, 1F																						
XY		0	1	7	0	23	2	0	0	0	0	0	0	0	0	0	0	0	0	20	1	5	5
ZW		0	0	6	1	1	1	0	0	0	0	0	0	0	0	0	0	0	0	1	0	3	1
**mean per chromosome log2(XY:ZW) ratio of SNP density**																							
	1M, 1F	-0.09905749	0.48543652	0.22859461	0.10937844	0.7656366	0.21892497	-0.12299044	0.16360203	-1.18052557	-0.64172876	-0.01643547	-0.11672864	0.7234985	-0.20209618	0.41363452	-0.23071611	0.48845998	-0.84189946	0.48771449	0.12983373	0.16304591	1.5860364
**Dunn's test to identifiy outlier chromosomes**	1M, 1F																						
	LG01																						
	LG02	***																					
	LG03	ns	ns																				
	LG04	ns	*	ns																			
	LG05	****	ns	**	**																		
	LG06	*	ns	ns	ns	*																	
	LG07	ns	****	*	ns	****	**																
	LG08	ns	ns	ns	ns	*	ns	*															
	LG09	****	****	****	****	****	****	****	****														
	LG10	***	****	****	****	****	****	***	****	*													
	LG11	ns	***	ns	ns	****	ns	ns	ns	****	***												
	LG12	ns	****	*	ns	****	**	ns	ns	****	**	ns											
	LG13	****	ns	**	***	ns	**	****	**	****	****	****	****										
	LG14	ns	****	**	ns	****	**	ns	*	****	**	ns	ns	****									
	LG15	***	ns	ns	ns	ns	ns	****	ns	****	****	**	***	ns	****								
	LG16	ns	****	*	ns	****	**	ns	*	****	**	ns	ns	****	ns	****							
	LG17	***	ns	ns	*	ns	ns	****	ns	****	****	***	****	ns	****	ns	****						
	LG18	****	****	****	****	****	****	****	****	ns	ns	****	***	****	***	****	***	****					
	LG19	**	ns	ns	ns	ns	ns	**	ns	****	****	*	**	ns	***	ns	***	ns	****				
	LG20	ns	****	*	ns	****	*	ns	ns	****	**	ns	ns	****	ns	***	ns	****	***	**			
	LG22	**	ns	ns	ns	ns	ns	***	ns	****	****	**	***	ns	****	ns	***	ns	****	ns	***		
	LG23	****	****	****	****	****	****	****	****	****	****	****	****	****	****	****	****	****	****	****	****	****	

Petrochromis sp. 'red'		**LG01**	**LG02**	**LG03**	**LG04**	**LG05**	**LG06**	**LG07**	**LG08**	**LG09**	**LG10**	**LG11**	**LG12**	**LG13**	**LG14**	**LG15**	**LG16**	**LG17**	**LG18**	**LG19**	**LG20**	**LG22**	**LG23**
**Sex-specific SNP inspection**																							
Individual	1M, 1F																						
Transcriptome		[redacted]																					
Pooled																							
**Kruskal-Wallis for heterogeneity among chromosomes, df = 21**																							
	1M, 1F																						
	chi-squared = 79.617, p-value < 9.355e-09																						
**Top 1% (# windows per chromosome)**																							
	1M, 1F																						
XY		0	3	5	0	8	1	1	0	2	2	1	1	0	0	0	0	0	4	12	0	5	1
ZW		0	4	11	1	0	0	0	1	3	0	0	0	2	0	0	2	0	4	0	0	5	2
**mean per chromosome log2(XY:ZW) ratio of SNP density**																							
	1M, 1F	0.13210226	0.07896166	-0.09093339	-0.10601577	0.50632301	-0.07863083	0.03183419	-0.12223235	0.10952519	0.05748468	0.0421005	-0.15478177	0.11931875	-0.03380464	-0.17761156	0.03214181	-0.02494644	0.01897743	0.62085397	0.23104047	0.03373921	-0.01996731
**Dunn's test to identifiy outlier chromosomes**	1M, 1F																						
	LG01																						
	LG02	ns																					
	LG03	ns	ns																				
	LG04	ns	ns	ns																			
	LG05	ns	*	****	****																		
	LG06	ns	ns	ns	ns	****																	
	LG07	ns	ns	ns	ns	**	ns																
	LG08	ns	ns	ns	ns	****	ns	ns															
	LG09	ns	ns	ns	ns	ns	ns	ns	ns														
	LG10	ns	ns	ns	ns	**	ns	ns	ns	ns													
	LG11	ns	ns	ns	ns	**	ns	ns	ns	ns	ns												
	LG12	ns	ns	ns	ns	****	ns	ns	ns	ns	ns	ns											
	LG13	ns	ns	ns	ns	ns	ns	ns	ns	ns	ns	ns	ns										
	LG14	ns	ns	ns	ns	***	ns	ns	ns	ns	ns	ns	ns	ns									
	LG15	*	ns	ns	ns	****	ns	ns	ns	ns	ns	ns	ns	*	ns								
	LG16	ns	ns	ns	ns	**	ns	ns	ns	ns	ns	ns	ns	ns	ns	ns							
	LG17	ns	ns	ns	ns	**	ns	ns	ns	ns	ns	ns	ns	ns	ns	ns	ns						
	LG18	ns	ns	ns	ns	**	ns	ns	ns	ns	ns	ns	ns	ns	ns	ns	ns	ns					
	LG19	*	**	****	****	ns	****	***	****	*	**	**	****	*	***	****	**	***	**				
	LG20	ns	ns	ns	ns	**	ns	ns	ns	ns	ns	ns	ns	ns	ns	ns	ns	ns	ns	**			
	LG22	ns	ns	ns	ns	*	ns	ns	ns	ns	ns	ns	ns	ns	ns	ns	ns	ns	ns	*	ns		
	LG23	ns	ns	ns	ns	***	ns	ns	ns	ns	ns	ns	ns	ns	ns	ns	ns	ns	ns	***	ns	ns	

Petrochromis sp. 'moshi yellow'		LG01	LG02	LG03	LG04	LG05	LG06	LG07	LG08	LG09	LG10	LG11	LG12	LG13	LG14	LG15	LG16	LG17	LG18	LG19	LG20	LG22	LG23
**Sex-specific SNP inspection**																							
Individual	1M, 1F																						
Transcriptome		[redacted]																					
Pooled																							
**Kruskal-Wallis for heterogeneity among chromosomes, df = 21**	1M, 1F																						
	chi-squared = 74.824, p-value < 5.805e-08																						
**Top 1% (# windows per chromosome)**																							
	1M, 1F																						
XY		0	3	7	5	0	0	0	0	7	0	0	0	3	0	2	0	0	2	0	1	5	1
ZW		0	2	12	3	1	1	0	0	1	0	3	0	1	0	1	2	0	4	0	1	4	5
**mean per chromosome log2(XY:ZW) ratio of SNP density**																							
	1M, 1F	-0.00770554	0.21961709	-0.06305203	0.19460564	-0.06830082	-0.21578304	-0.05297901	-0.32771574	0.01202627	0.13776607	0.15538016	-0.21917803	0.11135061	-0.16184737	-0.20820721	-0.16593881	-0.06513884	0.15875508	-0.00942874	0.04119061	0.14042337	-0.10717652
**Dunn's test to identifiy outlier chromosomes**	1M, 1F																						
	LG01																						
	LG02	ns																					
	LG03	ns	*																				
	LG04	ns	ns	ns																			
	LG05	ns	ns	ns	ns																		
	LG06	ns	**	ns	**	ns																	
	LG07	ns	*	ns	ns	ns	ns																
	LG08	*	***	ns	**	*	ns	*															
	LG09	ns	ns	ns	ns	ns	ns	ns	ns														
	LG10	ns	ns	ns	ns	ns	*	ns	**	ns													
	LG11	ns	ns	ns	ns	ns	*	ns	**	ns	ns												
	LG12	ns	**	ns	**	ns	ns	ns	ns	ns	*	*											
	LG13	ns	ns	ns	ns	ns	*	ns	**	ns	ns	ns	*										
	LG14	ns	**	ns	*	ns	ns	ns	ns	ns	*	*	ns	ns									
	LG15	ns	**	ns	**	ns	ns	ns	ns	ns	*	*	ns	*	ns								
	LG16	ns	*	ns	*	ns	ns	ns	ns	ns	ns	ns	ns	ns	ns	ns							
	LG17	ns	*	ns	ns	ns	ns	ns	ns	ns	ns	ns	ns	ns	ns	ns	ns						
	LG18	ns	ns	ns	ns	ns	*	ns	**	ns	ns	ns	*	ns	*	*	ns	ns					
	LG19	ns	ns	ns	ns	ns	ns	ns	*	ns	ns	ns	ns	ns	ns	ns	ns	ns	ns				
	LG20	ns	ns	ns	ns	ns	ns	ns	*	ns	ns	ns	ns	ns	ns	ns	ns	ns	ns	ns			
	LG22	ns	ns	ns	ns	ns	*	ns	**	ns	ns	ns	**	ns	*	*	*	ns	ns	ns	ns		
	LG23	ns	*	ns	ns	ns	ns	ns	ns	ns	ns	ns	ns	ns	ns	ns	ns	ns	ns	ns	ns	ns	

Petrochromis trewavasae ephippium		LG01	LG02	LG03	LG04	LG05	LG06	LG07	LG08	LG09	LG10	LG11	LG12	LG13	LG14	LG15	LG16	LG17	LG18	LG19	LG20	LG22	LG23
**Sex-specific SNP inspection**																							
Individual	1M, 1F																						
Transcriptome	3M, 3F																						
Pooled		[redacted]																					
**Kruskal-Wallis for heterogeneity among chromosomes, df = 21**																							
	1M, 1F																						
	chi-squared = 140.9, p-value < 2.2e-16																						
**Top 1% (# windows per chromosome)**																							
	1M, 1F																						
XY		0	2	10	1	0	0	0	1	1	0	2	0	0	2	1	0	2	5	0	1	6	0
ZW		1	2	12	3	0	1	1	1	3	0	1	0	1	1	3	0	2	2	0	1	5	3
**mean per chromosome log2(XY:ZW) ratio of SNP density**																							
	1M, 1F	-0.14424	-0.02646187	0.68126278	0.53014223	0.12194627	-0.0860386	0.1131773	-0.00362487	-0.12002966	0.00848971	-0.03470317	0.41187814	-0.09246498	-0.05579368	0.03251298	0.02843343	0.27296271	-0.09870019	0.13315099	0.21018631	0.0487506	-0.00852446
**Dunn's test to identifiy outlier chromosomes**	1M, 1F																						
	LG01																						
	LG02	ns																					
	LG03	****	****																				
	LG04	****	****	ns																			
	LG05	ns	ns	****	**																		
	LG06	ns	ns	****	****	ns																	
	LG07	*	ns	****	***	ns	ns																
	LG08	ns	ns	****	***	ns	ns	ns															
	LG09	ns	ns	****	****	ns	ns	ns	ns														
	LG10	ns	ns	****	****	ns	ns	ns	ns	ns													
	LG11	ns	ns	****	****	ns	ns	ns	ns	ns	ns												
	LG12	***	**	*	ns	ns	**	ns	*	**	*	**											
	LG13	ns	ns	****	****	ns	ns	ns	ns	ns	ns	ns	***										
	LG14	ns	ns	****	****	ns	ns	ns	ns	ns	ns	ns	**	ns									
	LG15	ns	ns	****	***	ns	ns	ns	ns	ns	ns	ns	*	ns	ns								
	LG16	ns	ns	****	***	ns	ns	ns	ns	ns	ns	ns	*	ns	ns	ns							
	LG17	***	*	**	ns	ns	**	ns	ns	**	*	**	ns	***	**	ns	ns						
	LG18	ns	ns	****	****	ns	ns	ns	ns	ns	ns	ns	**	ns	ns	ns	ns	**					
	LG19	*	ns	***	*	ns	ns	ns	ns	ns	ns	ns	ns	ns	ns	ns	ns	ns	ns				
	LG20	ns	ns	****	**	ns	ns	ns	ns	ns	ns	ns	ns	ns	ns	ns	ns	ns	ns	ns			
	LG22	ns	ns	****	***	ns	ns	ns	ns	ns	ns	ns	ns	ns	ns	ns	ns	ns	ns	ns	ns		
	LG23	ns	ns	****	****	ns	ns	ns	ns	ns	ns	ns	*	ns	ns	ns	ns	*	ns	ns	ns	ns	

Petrochromis sp. 'macrognathus rainbow'		LG01	LG02	LG03	LG04	LG05	LG06	LG07	LG08	LG09	LG10	LG11	LG12	LG13	LG14	LG15	LG16	LG17	LG18	LG19	LG20	LG22	LG23
**Sex-specific SNP inspection**																							
Individual	1M, 1F																						
Transcriptome		[redacted]																					
Pooled																							
**Kruskal-Wallis for heterogeneity among chromosomes, df = 21**																							
	1M, 1F																						
	chi-squared = 198.85, p-value < 2.2e-16																						
**Top 1% (# windows per chromosome)**																							
	1M, 1F																						
XY		1	0	8	0	8	2	1	1	1	0	0	0	1	0	2	0	0	0	20	0	5	2
ZW		0	1	7	1	1	2	0	0	0	0	2	0	0	0	0	1	0	1	1	0	6	3
**mean per chromosome log2(XY:ZW) ratio of SNP density**																							
	1M, 1F	-0.08535219	-0.10566172	0.06119606	0.05290293	0.90453784	0.19823853	0.07285151	-0.21124195	0.07751588	-0.00905218	-0.13694066	0.30009651	0.23577348	-0.22024983	0.25629923	0.00142031	0.17781878	-0.1794376	1.3335875	-0.01050435	-0.03813594	-0.16898071
**Dunn's test to identifiy outlier chromosomes**	1M, 1F																						
	LG01																						
	LG02	ns																					
	LG03	ns	ns																				
	LG04	ns	ns	ns																			
	LG05	****	****	****	****																		
	LG06	*	*	ns	ns	**																	
	LG07	ns	ns	ns	ns	****	ns																
	LG08	ns	ns	ns	ns	****	*	ns															
	LG09	ns	ns	ns	ns	****	ns	ns	ns														
	LG10	ns	ns	ns	ns	****	ns	ns	ns	ns													
	LG11	ns	ns	ns	ns	****	ns	ns	ns	ns	ns												
	LG12	*	*	ns	ns	**	ns	ns	*	ns	ns	*											
	LG13	ns	ns	ns	ns	**	ns	ns	ns	ns	ns	ns	ns										
	LG14	ns	ns	*	ns	****	**	*	ns	ns	ns	ns	***	**									
	LG15	*	*	ns	ns	**	ns	ns	*	ns	ns	*	ns	ns	***								
	LG16	ns	ns	ns	ns	****	ns	ns	ns	ns	ns	ns	ns	ns	ns	ns							
	LG17	ns	ns	ns	ns	***	ns	ns	ns	ns	ns	ns	ns	ns	*	ns	ns						
	LG18	ns	ns	ns	ns	****	*	ns	ns	ns	ns	ns	**	*	ns	**	ns	ns					
	LG19	****	****	****	****	*	****	****	****	****	****	****	****	****	****	****	****	****	****				
	LG20	ns	ns	ns	ns	****	ns	ns	ns	ns	ns	ns	*	ns	ns	*	ns	ns	ns	****			
	LG22	ns	ns	ns	ns	****	ns	ns	ns	ns	ns	ns	ns	ns	ns	ns	ns	ns	ns	****	ns		
	LG23	ns	ns	*	ns	****	**	*	ns	ns	ns	ns	**	*	ns	**	ns	*	ns	****	ns	ns	

Petrochromis sp. 'kazumbae'		LG01	LG02	LG03	LG04	LG05	LG06	LG07	LG08	LG09	LG10	LG11	LG12	LG13	LG14	LG15	LG16	LG17	LG18	LG19	LG20	LG22	LG23
**Sex-specific SNP inspection**																							
Individual	1M, 1F																						
Transcriptome		[redacted]																					
Pooled																							
**Kruskal-Wallis for heterogeneity among chromosomes, df = 21**																							
	1M, 1F																						
	chi-squared = 197.7, p-value < 2.2e-16																						
**Top 1% (# windows per chromosome)**																							
	1M, 1F																						
XY		0	2	11	0	1	0	0	2	2	1	1	4	0	0	2	0	0	5	0	1	10	1
ZW		0	4	7	1	0	3	2	0	0	0	2	4	0	0	2	0	1	4	0	1	4	2
**mean per chromosome log2(XY:ZW) ratio of SNP density**																							
	1M, 1F	0.03901373	-0.17659115	-0.22654403	0.05628171	0.47945724	-0.37728597	-0.20198757	0.13291679	-0.25581334	-0.16984905	0.26963092	-0.43823672	0.18952782	-0.04413833	0.03136433	-0.07610719	-0.07485879	0.19905442	-0.00231563	-0.09816852	0.18330099	-0.76472345
**Dunn's test to identifiy outlier chromosomes**	1M, 1F																						
	LG01																						
	LG02	ns																					
	LG03	ns	ns																				
	LG04	ns	ns	ns																			
	LG05	**	****	****	**																		
	LG06	**	ns	ns	*	****																	
	LG07	ns	ns	ns	ns	****	ns																
	LG08	ns	**	**	ns	ns	***	**															
	LG09	ns	ns	ns	ns	****	ns	ns	**														
	LG10	ns	ns	ns	ns	***	*	ns	ns	ns													
	LG11	ns	**	***	ns	ns	****	***	ns	**	ns												
	LG12	***	ns	ns	**	****	ns	*	****	ns	**	****											
	LG13	ns	**	***	ns	ns	****	***	ns	**	ns	ns	****										
	LG14	ns	ns	ns	ns	***	*	ns	ns	ns	ns	ns	**	ns									
	LG15	ns	ns	ns	ns	**	**	ns	ns	ns	ns	ns	***	ns	ns								
	LG16	ns	ns	ns	ns	***	*	ns	ns	ns	ns	ns	**	ns	ns	ns							
	LG17	ns	ns	ns	ns	***	*	ns	ns	ns	ns	ns	**	ns	ns	ns	ns						
	LG18	ns	***	***	ns	ns	****	***	ns	**	*	ns	****	ns	ns	ns	*	*					
	LG19	ns	ns	ns	ns	**	*	ns	ns	ns	ns	ns	**	ns	ns	ns	ns	ns	ns				
	LG20	ns	ns	ns	ns	**	*	ns	ns	ns	ns	ns	**	ns	ns	ns	ns	ns	ns	ns			
	LG22	*	****	****	*	ns	****	****	ns	***	**	ns	****	ns	*	*	**	**	ns	*	*		
	LG23	****	**	**	****	****	ns	***	****	*	****	****	ns	****	****	****	****	****	****	****	****	****	

Petrochromis sp. 'giant'		LG01	LG02	LG03	LG04	LG05	LG06	LG07	LG08	LG09	LG10	LG11	LG12	LG13	LG14	LG15	LG16	LG17	LG18	LG19	LG20	LG22	LG23
**Sex-specific SNP inspection**																							
Individual	1M, 1F																						
Transcriptome		[redacted]																					
Pooled																							
**Kruskal-Wallis for heterogeneity among chromosomes, df = 21**																							
	1M, 1F																						
	chi-squared = 134.2, p-value < 2.2e-16																						
**Top 1% (# windows per chromosome)**																							
	1M, 1F																						
XY		0	0	9	2	9	1	0	2	1	1	2	3	0	1	0	0	0	0	21	0	4	0
ZW		0	3	4	1	0	1	1	0	2	0	1	1	1	0	3	0	1	1	0	0	7	1
**mean per chromosome log2(XY:ZW) ratio of SNP density**																							
	1M, 1F	0.02783935	-0.02667604	0.12613925	-0.13460299	0.44600415	-0.10139665	-0.0596522	0.17964453	0.13470348	0.47059289	-0.13510548	-0.09718878	-0.3151799	-0.3862231	-0.14721957	0.06821946	0.09578963	-0.17552269	0.84730308	-0.22299081	0.00177184	-0.00273586
**Dunn's test to identifiy outlier chromosomes**	1M, 1F																						
	LG01																						
	LG02	ns																					
	LG03	ns	ns																				
	LG04	ns	ns	ns																			
	LG05	*	*	ns	**																		
	LG06	ns	ns	ns	ns	**																	
	LG07	ns	ns	ns	ns	***	ns																
	LG08	ns	ns	ns	ns	ns	ns	ns															
	LG09	ns	ns	ns	ns	ns	ns	ns	ns														
	LG10	**	**	*	***	ns	***	****	ns	ns													
	LG11	ns	ns	ns	ns	**	ns	ns	ns	ns	****												
	LG12	ns	ns	ns	ns	**	ns	ns	ns	ns	***	ns											
	LG13	ns	ns	*	ns	****	ns	ns	*	*	****	ns	ns										
	LG14	**	*	***	ns	****	*	*	**	**	****	ns	*	ns									
	LG15	ns	ns	ns	ns	***	ns	ns	ns	*	****	ns	ns	ns	ns								
	LG16	ns	ns	ns	ns	*	ns	ns	ns	ns	**	ns	ns	ns	**	ns							
	LG17	ns	ns	ns	ns	ns	ns	ns	ns	ns	*	ns	ns	ns	**	ns	ns						
	LG18	ns	ns	ns	ns	***	ns	ns	ns	ns	****	ns	ns	ns	ns	ns	ns	ns					
	LG19	****	****	****	****	*	****	****	***	**	ns	****	****	****	****	****	****	****	****				
	LG20	ns	ns	ns	ns	*	ns	ns	ns	ns	**	ns	ns	ns	*	ns	ns	ns	ns	****			
	LG22	ns	ns	ns	ns	**	ns	ns	ns	ns	***	ns	ns	ns	*	ns	ns	ns	ns	****	ns		
	LG23	ns	ns	ns	ns	*	ns	ns	ns	ns	***	ns	ns	ns	**	ns	ns	ns	ns	****	ns	ns	

Petrochromis sp. 'polyodon texas'		LG01	LG02	LG03	LG04	LG05	LG06	LG07	LG08	LG09	LG10	LG11	LG12	LG13	LG14	LG15	LG16	LG17	LG18	LG19	LG20	LG22	LG23
**Sex-specific SNP inspection**																							
Individual	1M, 1F																						
Transcriptome		[redacted]																					
Pooled																							
**Kruskal-Wallis for heterogeneity among chromosomes, df = 21**																							
	1M, 1F																						
	chi-squared = 151.3, p-value < 2.2e-16																						
**Top 1% (# windows per chromosome)**																							
	1M, 1F																						
XY		0	3	7	0	11	0	0	1	0	0	3	1	0	0	1	0	0	0	24	0	6	2
ZW		0	0	4	0	0	0	0	0	2	0	2	0	1	0	1	0	1	1	1	0	4	2
**mean per chromosome log2(XY:ZW) ratio of SNP density**																							
	1M, 1F	0.01457643	0.09038043	0.02987238	0.08784035	0.84839091	-0.19025996	0.15243038	0.61671327	-0.10293674	-0.13295307	0.11946747	0.12295223	0.11412453	-0.06079103	-0.1169741	-0.22551188	-0.20534525	-0.6401377	0.68739642	-0.29424691	-0.08111438	-0.15148338
**Dunn's test to identifiy outlier chromosomes**	1M, 1F																						
	LG01																						
	LG02	ns																					
	LG03	ns	ns																				
	LG04	ns	ns	ns																			
	LG05	****	****	****	***																		
	LG06	ns	ns	ns	ns	****																	
	LG07	ns	ns	ns	ns	****	*																
	LG08	**	**	**	*	ns	****	*															
	LG09	ns	ns	ns	ns	****	ns	ns	***														
	LG10	ns	ns	ns	ns	****	ns	*	****	ns													
	LG11	ns	ns	ns	ns	***	ns	ns	*	ns	ns												
	LG12	ns	ns	ns	ns	***	*	ns	ns	ns	ns	ns											
	LG13	ns	ns	ns	ns	****	ns	ns	*	ns	ns	ns	ns										
	LG14	ns	ns	ns	ns	****	ns	ns	***	ns	ns	ns	ns	ns									
	LG15	ns	ns	ns	ns	****	ns	*	***	ns	ns	ns	ns	ns	ns								
	LG16	ns	ns	ns	ns	****	ns	*	****	ns	ns	ns	ns	ns	ns	ns							
	LG17	ns	ns	ns	ns	****	ns	**	****	ns	ns	*	*	ns	ns	ns	ns						
	LG18	***	***	***	***	****	*	****	****	ns	ns	****	****	***	*	*	*	ns					
	LG19	*	ns	*	ns	ns	***	ns	ns	**	***	ns	ns	ns	**	**	***	***	****				
	LG20	ns	ns	ns	ns	****	ns	ns	***	ns	ns	ns	ns	ns	ns	ns	ns	ns	*	**			
	LG22	ns	ns	ns	ns	****	ns	ns	***	ns	ns	ns	ns	ns	ns	ns	ns	ns	*	**	ns		
	LG23	ns	ns	ns	ns	****	ns	*	****	ns	ns	ns	*	ns	ns	ns	ns	ns	*	***	ns	ns	

Petrochromis polyodon		LG01	LG02	LG03	LG04	LG05	LG06	LG07	LG08	LG09	LG10	LG11	LG12	LG13	LG14	LG15	LG16	LG17	LG18	LG19	LG20	LG22	LG23
**Sex-specific SNP inspection**																							
Individual	1M, 1F																						
Transcriptome	3M, 3F																						
Pooled		[redacted]																					
**Kruskal-Wallis for heterogeneity among chromosomes, df = 21**																							
	1M, 1F																						
	chi-squared = 53.476, p-value < 0.0001175																						
**Top 1% (# windows per chromosome)**																							
	1M, 1F																						
XY		0	0	7	1	1	0	0	2	3	1	5	1	0	1	2	2	1	2	3	0	2	3
ZW		0	3	12	0	0	2	0	2	1	0	2	3	1	0	3	0	0	1	0	1	3	5
**mean per chromosome log2(XY:ZW) ratio of SNP density**																							
	1M, 1F	-0.15334861	-0.07646526	-0.00993119	-0.11848671	0.16951522	-0.22586256	-0.07853516	-0.14648504	-0.38961637	0.01305264	0.06585737	-0.03444309	0.0492916	0.07139378	0.11241007	-0.1407037	0.33065771	-0.05205793	0.27022938	-0.1917905	0.00430829	-0.05442338
**Dunn's test to identifiy outlier chromosomes**	1M, 1F																						
	LG01																						
	LG02	ns																					
	LG03	ns	ns																				
	LG04	ns	ns	ns																			
	LG05	ns	ns	ns	ns																		
	LG06	ns	ns	ns	ns	*																	
	LG07	ns	ns	ns	ns	ns	ns																
	LG08	ns	ns	ns	ns	ns	ns	ns															
	LG09	ns	ns	ns	ns	*	ns	ns	ns														
	LG10	ns	ns	ns	ns	ns	ns	ns	ns	ns													
	LG11	ns	ns	ns	ns	ns	ns	ns	ns	ns	ns												
	LG12	ns	ns	ns	ns	ns	ns	ns	ns	ns	ns	ns											
	LG13	ns	ns	ns	ns	ns	ns	ns	ns	ns	ns	ns	ns										
	LG14	ns	ns	ns	ns	ns	ns	ns	ns	*	ns	ns	ns	ns									
	LG15	ns	ns	ns	ns	ns	*	ns	ns	*	ns	ns	ns	ns	ns								
	LG16	ns	ns	ns	ns	ns	ns	ns	ns	ns	ns	ns	ns	ns	ns	ns							
	LG17	*	*	ns	*	ns	**	*	*	**	ns	ns	*	ns	ns	ns	*						
	LG18	ns	ns	ns	ns	ns	ns	ns	ns	ns	ns	ns	ns	ns	ns	ns	ns	*					
	LG19	ns	ns	ns	ns	ns	*	ns	ns	*	ns	ns	ns	ns	ns	ns	ns	ns	ns				
	LG20	ns	ns	ns	ns	ns	ns	ns	ns	ns	ns	ns	ns	ns	ns	ns	ns	ns	ns	ns			
	LG22	ns	ns	ns	ns	ns	ns	ns	ns	ns	ns	ns	ns	ns	ns	ns	ns	ns	ns	ns	ns		
	LG23	ns	ns	ns	ns	ns	ns	ns	ns	ns	ns	ns	ns	ns	ns	ns	ns	*	ns	ns	ns	ns	

Simochromis diagramma		LG01	LG02	LG03	LG04	LG05	LG06	LG07	LG08	LG09	LG10	LG11	LG12	LG13	LG14	LG15	LG16	LG17	LG18	LG19	LG20	LG22	LG23
**Sex-specific SNP inspection**																							
Individual	1M, 1F																						
Transcriptome	3M, 3F																						
Pooled	20M, 28F																						
**Kruskal-Wallis for heterogeneity among chromosomes, df = 21**																							
	1M, 1F																						
	chi-squared = 717.56, p-value < 2.2e-16																						
	20M, 28F																						
	chi-squared = 284.98, p-value < 2.2e-16																						
**Top 1% (# windows per chromosome)**																							
	1M, 1F																						
XY		0	0	7	0	0	0	0	22	3	0	2	2	0	0	0	11	0	1	1	1	4	0
ZW		0	1	4	0	0	2	1	0	3	1	1	5	1	0	0	0	0	1	0	0	4	0
	20M, 28F																						
XY		0	0	0	0	0	0	34	0	0	0	0	0	0	0	0	0	0	0	0	0	0	0
ZW		0	0	0	0	0	0	45	0	0	0	0	0	0	0	0	0	0	0	0	0	0	0
**mean per chromosome log2(XY:ZW) ratio of SNP density**																							
	1M, 1F	-0.06202434	-0.05146128	0.17245137	0.06367982	0.02491249	0.03593434	0.03715847	4.65825815	-0.07666338	-0.03273184	-0.0660748	-0.02039759	-0.42920001	-0.49792116	-0.10354835	1.28973704	-0.03007606	1.17746478	-0.01343392	-0.1597537	0.00660171	-0.2565977
	20M, 28F	-0.05943348	-0.57248094	0.02258679	-0.26951338	8.3028E-05	0.23674378	0.07892509	-0.43631055	-0.01392336	-0.00213504	-0.09527895	-0.19838009	0.0739121	-0.02431009	0.05023995	0.17521673	-0.02478864	0.09305022	-0.32026913	0.61537222	-0.07267939	-0.21359688
**Dunn's test to identifiy outlier chromosomes**	1M, 1F																						
	LG01																						
	LG02	ns																					
	LG03	*	ns																				
	LG04	ns	ns	ns																			
	LG05	ns	ns	ns	ns																		
	LG06	ns	ns	ns	ns	ns																	
	LG07	ns	ns	ns	ns	ns	ns																
	LG08	****	****	****	****	****	****	****															
	LG09	ns	ns	ns	ns	ns	ns	ns	****														
	LG10	ns	ns	ns	ns	ns	ns	ns	****	ns													
	LG11	ns	ns	ns	ns	ns	ns	ns	****	ns	ns												
	LG12	ns	ns	ns	ns	ns	ns	ns	****	ns	ns	ns											
	LG13	ns	ns	**	ns	ns	ns	*	****	ns	ns	ns	ns										
	LG14	ns	ns	**	ns	ns	*	**	****	ns	ns	ns	ns	ns									
	LG15	ns	ns	*	ns	ns	ns	ns	****	ns	ns	ns	ns	ns	ns								
	LG16	****	****	****	****	****	****	****	****	****	****	****	****	****	****	****							
	LG17	ns	ns	ns	ns	ns	ns	ns	****	ns	ns	ns	ns	ns	ns	ns	****						
	LG18	****	****	*	***	***	***	***	****	****	****	****	****	****	****	****	ns	****					
	LG19	ns	ns	ns	ns	ns	ns	ns	****	ns	ns	ns	ns	ns	*	ns	****	ns	**				
	LG20	ns	ns	ns	ns	ns	ns	ns	****	ns	ns	ns	ns	ns	ns	ns	****	ns	****	ns			
	LG22	ns	ns	ns	ns	ns	ns	ns	****	ns	ns	ns	ns	ns	*	ns	****	ns	***	ns	ns		
	LG23	ns	ns	**	ns	ns	ns	*	****	ns	ns	ns	ns	ns	ns	ns	****	ns	****	ns	ns	ns	
**Dunn's test to identifiy outlier chromosomes**	20M, 28F																						
	LG01																						
	LG02	****																					
	LG03	ns	****																				
	LG04	*	**	**																			
	LG05	ns	****	ns	**																		
	LG06	***	****	**	****	**																	
	LG07	ns	****	ns	***	ns	*																
	LG08	***	ns	****	ns	****	****	****															
	LG09	ns	****	ns	*	ns	*	ns	****														
	LG10	ns	****	ns	*	ns	**	ns	****	ns													
	LG11	ns	****	ns	ns	ns	***	ns	***	ns	ns												
	LG12	ns	***	*	ns	*	****	**	*	*	*	ns											
	LG13	ns	****	ns	***	ns	ns	ns	****	ns	ns	ns	**										
	LG14	ns	****	ns	*	ns	**	ns	****	ns	ns	ns	*	ns									
	LG15	ns	****	ns	**	ns	*	ns	****	ns	ns	ns	**	ns	ns								
	LG16	*	****	ns	****	ns	ns	ns	****	ns	ns	*	****	ns	ns	ns							
	LG17	ns	****	ns	*	ns	**	ns	****	ns	ns	ns	ns	ns	ns	ns	ns						
	LG18	ns	****	ns	***	ns	ns	ns	****	ns	ns	*	***	ns	ns	ns	ns	ns					
	LG19	**	*	**	ns	**	****	****	ns	**	**	*	ns	****	**	***	****	**	****				
	LG20	****	****	****	****	****	**	****	****	****	****	****	****	****	****	****	****	****	****	****			
	LG22	ns	****	ns	ns	ns	***	ns	***	ns	ns	ns	ns	ns	ns	ns	**	ns	*	*	****		
	LG23	ns	****	*	ns	*	****	**	*	*	*	ns	ns	**	*	**	****	ns	***	ns	****	ns	

Petrochromis fasciolatus		LG01	LG02	LG03	LG04	LG05	LG06	LG07	LG08	LG09	LG10	LG11	LG12	LG13	LG14	LG15	LG16	LG17	LG18	LG19	LG20	LG22	LG23
**Sex-specific SNP inspection**																							
Individual	1M, 1F																						
Transcriptome	3M, 3F																						
Pooled		[redacted]																					
**Kruskal-Wallis for heterogeneity among chromosomes, df = 21**																							
	1M, 1F																						
	chi-squared = 144.57, p-value < 2.2e-16																						
**Top 1% (# windows per chromosome)**																							
	1M, 1F																						
XY		0	4	8	0	15	0	0	1	3	0	1	0	1	1	0	0	0	0	20	0	4	0
ZW		0	3	6	0	2	0	0	0	1	1	1	0	1	0	0	0	0	0	1	0	5	1
**mean per chromosome log2(XY:ZW) ratio of SNP density**																							
	1M, 1F	0.14859253	0.05941275	0.17528643	-0.00151164	0.61921987	-0.14019926	0.21877336	0.42153676	0.1723648	0.13837747	0.01622329	0.23212898	0.01507574	0.25902115	-0.19250303	0.31607258	-0.06501997	-0.1022818	0.71413213	-0.00299202	0.33396132	-0.01289001
**Dunn's test to identifiy outlier chromosomes**	1M, 1F																						
	LG01																						
	LG02	ns																					
	LG03	ns	ns																				
	LG04	ns	ns	ns																			
	LG05	ns	*	ns	**																		
	LG06	**	*	**	ns	****																	
	LG07	ns	ns	ns	*	ns	***																
	LG08	*	**	*	***	ns	****	ns															
	LG09	ns	ns	ns	ns	ns	**	ns	ns														
	LG10	ns	ns	ns	ns	ns	**	ns	ns	ns													
	LG11	ns	ns	ns	ns	*	ns	ns	**	ns	ns												
	LG12	ns	ns	ns	*	ns	***	ns	ns	ns	ns	ns											
	LG13	ns	ns	ns	ns	**	ns	ns	**	ns	ns	ns	ns										
	LG14	ns	ns	ns	**	ns	****	ns	ns	ns	ns	*	ns	*									
	LG15	**	*	**	ns	****	ns	***	****	*	**	ns	***	ns	****								
	LG16	ns	ns	ns	**	ns	****	ns	ns	ns	ns	*	ns	*	ns	****							
	LG17	ns	ns	ns	ns	***	ns	**	****	ns	*	ns	**	ns	**	ns	**						
	LG18	**	ns	**	ns	****	ns	***	****	*	**	ns	***	ns	****	ns	****	ns					
	LG19	*	**	*	***	ns	****	ns	ns	ns	ns	**	ns	**	ns	****	ns	****	****				
	LG20	ns	ns	ns	ns	ns	*	ns	*	ns	ns	ns	ns	ns	ns	*	ns	ns	*	*			
	LG22	**	**	**	****	ns	****	*	ns	*	*	***	ns	***	ns	****	ns	****	****	ns	**		
	LG23	ns	ns	ns	ns	**	ns	ns	***	ns	ns	ns	ns	ns	*	ns	*	ns	ns	**	ns	****	

Interochromis loocki		LG01	LG02	LG03	LG04	LG05	LG06	LG07	LG08	LG09	LG10	LG11	LG12	LG13	LG14	LG15	LG16	LG17	LG18	LG19	LG20	LG22	LG23
**Sex-specific SNP inspection**																							
Individual	1M, 1F																						
Transcriptome	3M, 3F																						
Pooled	24M, 21F																						
**Kruskal-Wallis for heterogeneity among chromosomes, df = 21**		[redacted]																					
	1M, 1F																						
	chi-squared = 163.54, p-value < 2.2e-16																						
	24M, 21F																						
	chi-squared = 174.38, p-value < 2.2e-16																						
**Top 1% (# windows per chromosome)**																							
	1M, 1F																						
XY		0	0	4	0	21	0	0	0	1	0	0	1	0	0	0	1	0	0	36	0	3	1
ZW		0	1	5	0	0	0	0	0	0	0	2	0	0	0	0	0	0	0	1	0	2	2
	24M, 21F																						
XY		0	0	0	0	32	0	0	0	0	0	0	0	0	0	0	0	0	0	45	0	0	0
ZW		0	0	0	0	0	0	0	0	0	0	0	0	0	0	0	0	0	0	0	0	0	0
**mean per chromosome log2(XY:ZW) ratio of SNP density**																							
	1M, 1F	-0.04195496	-0.06814053	0.06437995	0.39799201	1.00131469	0.10741215	-0.01724129	-0.05567423	0.11136079	-0.03352408	-0.03725456	0.06818592	-0.09757349	0.01328749	0.08673455	0.17512696	0.11439296	0.14139143	1.26500171	0.11605541	0.12013612	-0.01587815
	24M, 21F	-0.08125056	-0.07607142	-0.0415534	-0.04535452	1.65666905	-0.02731458	-0.05350434	0.1731192	0.04147099	-0.13278817	-0.05274897	0.0034545	-0.03545695	0.01262268	0.05757074	-0.02342532	0.02695636	-0.06601661	1.77003794	-0.11074785	0.05348336	0.0694788
**Dunn's test to identifiy outlier chromosomes**	1M, 1F																						
	LG01																						
	LG02	ns																					
	LG03	ns	ns																				
	LG04	****	****	***																			
	LG05	****	****	****	ns																		
	LG06	ns	ns	ns	*	****																	
	LG07	ns	ns	ns	****	****	ns																
	LG08	ns	ns	ns	****	****	ns	ns															
	LG09	ns	ns	ns	*	***	ns	ns	ns														
	LG10	ns	ns	ns	****	****	ns	ns	ns	ns													
	LG11	ns	ns	ns	****	****	ns	ns	ns	ns	ns												
	LG12	ns	ns	ns	**	****	ns	ns	ns	ns	ns	ns											
	LG13	ns	ns	ns	****	****	ns	ns	ns	ns	ns	ns	ns										
	LG14	ns	ns	ns	***	****	ns	ns	ns	ns	ns	ns	ns	ns									
	LG15	ns	ns	ns	**	****	ns	ns	ns	ns	ns	ns	ns	ns	ns								
	LG16	ns	ns	ns	*	****	ns	ns	ns	ns	ns	ns	ns	ns	ns	ns							
	LG17	ns	ns	ns	**	****	ns	ns	ns	ns	ns	ns	ns	ns	ns	ns	ns						
	LG18	ns	ns	ns	*	***	ns	ns	ns	ns	ns	ns	ns	ns	ns	ns	ns	ns					
	LG19	****	****	****	*	ns	****	****	****	****	****	****	****	****	****	****	****	****	****				
	LG20	ns	ns	ns	***	****	ns	ns	ns	ns	ns	ns	ns	ns	ns	ns	ns	ns	ns	****			
	LG22	*	*	ns	ns	**	ns	ns	*	ns	ns	*	ns	*	ns	ns	ns	ns	ns	****	ns		
	LG23	ns	ns	ns	***	****	ns	ns	ns	ns	ns	ns	ns	ns	ns	ns	ns	ns	ns	****	ns	ns	
**Dunn's test to identifiy outlier chromosomes**	24M, 21F																						
	LG01																						
	LG02	ns																					
	LG03	ns	ns																				
	LG04	ns	ns	ns																			
	LG05	****	****	****	****																		
	LG06	ns	ns	ns	ns	****																	
	LG07	ns	ns	ns	ns	****	ns																
	LG08	ns	ns	ns	ns	****	ns	ns															
	LG09	ns	ns	ns	ns	****	ns	ns	ns														
	LG10	ns	ns	ns	ns	****	ns	ns	*	ns													
	LG11	ns	ns	ns	ns	****	ns	ns	ns	ns	ns												
	LG12	ns	ns	ns	ns	****	ns	ns	ns	ns	ns	ns											
	LG13	ns	ns	ns	ns	****	ns	ns	ns	ns	ns	ns	ns										
	LG14	ns	ns	ns	ns	****	ns	ns	ns	ns	ns	ns	ns	ns									
	LG15	ns	ns	ns	ns	****	ns	ns	ns	ns	ns	ns	ns	ns	ns								
	LG16	ns	ns	ns	ns	****	ns	ns	ns	ns	ns	ns	ns	ns	ns	ns							
	LG17	ns	ns	ns	ns	****	ns	ns	ns	ns	ns	ns	ns	ns	ns	ns	ns						
	LG18	ns	ns	ns	ns	****	ns	ns	ns	ns	ns	ns	ns	ns	ns	ns	ns	ns					
	LG19	****	****	****	****	ns	****	****	****	****	****	****	****	****	****	****	****	****	****				
	LG20	ns	ns	ns	ns	****	ns	ns	*	ns	ns	ns	ns	ns	ns	ns	ns	ns	ns	****			
	LG22	ns	ns	ns	ns	****	ns	ns	ns	ns	ns	ns	ns	ns	ns	ns	ns	ns	ns	****	ns		
	LG23	ns	ns	ns	ns	****	ns	ns	ns	ns	ns	ns	ns	ns	ns	ns	ns	ns	ns	****	ns	ns	

Petrochromis sp. 'orthognathus ikola'		**LG01**	**LG02**	**LG03**	**LG04**	**LG05**	**LG06**	**LG07**	**LG08**	**LG09**	**LG10**	**LG11**	**LG12**	**LG13**	**LG14**	**LG15**	**LG16**	**LG17**	**LG18**	**LG19**	**LG20**	**LG22**	**LG23**
**Sex-specific SNP inspection**																							
Individual	1M, 1F																						
Transcriptome		[redacted]																					
Pooled		[redacted]																					
**Kruskal-Wallis for heterogeneity among chromosomes, df = 21**		[redacted]																					
	1M, 1F	[redacted]																					
	chi-squared = 139.37, p-value < 2.2e-16	[redacted]																					
**Top 1% (# windows per chromosome)**																							
	1M, 1F																						
XY		0	0	3	0	19	1	0	0	0	0	1	1	0	0	0	0	0	0	36	0	3	0
ZW		0	3	5	1	2	0	0	0	1	0	0	0	0	0	1	0	0	0	0	0	1	1
**mean per chromosome log2(XY:ZW) ratio of SNP density**																							
	1M, 1F	-0.08787126	0.26465304	-0.00828909	-0.23012879	0.74276756	0.06548865	0.10832845	0.01900148	-0.23023339	-0.25249314	0.05189691	0.15815483	0.03630001	0.06786996	-0.02061089	0.1940123	-0.05722613	0.26200926	0.97016535	0.2608027	0.00677714	-0.37425132
**Dunn's test to identifiy outlier chromosomes**	1M, 1F																						
	LG01																						
	LG02	**																					
	LG03	ns	ns																				
	LG04	ns	***	ns																			
	LG05	****	ns	***	****																		
	LG06	ns	ns	ns	*	**																	
	LG07	ns	ns	ns	*	**	ns																
	LG08	ns	ns	ns	ns	**	ns	ns															
	LG09	ns	**	ns	ns	****	ns	ns	ns														
	LG10	ns	***	ns	ns	****	*	*	ns	ns													
	LG11	ns	ns	ns	ns	**	ns	ns	ns	ns	ns												
	LG12	ns	ns	ns	*	*	ns	ns	ns	*	*	ns											
	LG13	ns	ns	ns	ns	**	ns	ns	ns	ns	ns	ns	ns										
	LG14	ns	ns	ns	ns	**	ns	ns	ns	ns	ns	ns	ns	ns									
	LG15	ns	ns	ns	ns	***	ns	ns	ns	ns	ns	ns	ns	ns	ns								
	LG16	*	ns	ns	**	ns	ns	ns	ns	*	**	ns	ns	ns	ns	ns							
	LG17	ns	*	ns	ns	***	ns	ns	ns	ns	ns	ns	ns	ns	ns	ns	ns						
	LG18	**	ns	*	***	ns	ns	ns	ns	**	***	ns	ns	ns	ns	*	ns	**					
	LG19	****	**	****	****	ns	****	****	****	****	****	****	***	****	****	****	**	****	*				
	LG20	ns	ns	ns	ns	**	ns	ns	ns	ns	ns	ns	ns	ns	ns	ns	ns	ns	*	****			
	LG22	ns	ns	ns	ns	**	ns	ns	ns	ns	ns	ns	ns	ns	ns	ns	ns	ns	*	****	ns		
	LG23	ns	****	**	ns	****	***	****	*	ns	ns	**	***	**	**	*	****	*	****	****	*	**	

Petrochromis orthognathus		LG01	LG02	LG03	LG04	LG05	LG06	LG07	LG08	LG09	LG10	LG11	LG12	LG13	LG14	LG15	LG16	LG17	LG18	LG19	LG20	LG22	LG23
**Sex-specific SNP inspection**																							
Individual	1M, 1F																						
Transcriptome		[redacted]																					
Pooled																							
**Kruskal-Wallis for heterogeneity among chromosomes, df = 21**																							
	1M, 1F																						
	chi-squared = 104.07, p-value < 5.459e-13																						
**Top 1% (# windows per chromosome)**																							
	1M, 1F																						
XY		1	0	6	1	2	1	3	0	1	2	5	0	0	1	0	0	0	0	0	0	6	2
ZW		2	1	8	2	0	3	0	1	1	3	8	1	1	2	1	1	1	2	0	2	6	2
**mean per chromosome log2(XY:ZW) ratio of SNP density**																							
	1M, 1F	-0.13987556	-0.1343172	-0.09269267	0.19397163	-0.19232837	-0.01857782	-0.18745473	-0.14208092	-0.12914689	-0.12521432	-0.16823865	0.05773962	0.36613384	-0.02135379	-0.08638872	-0.11962645	0.07006026	-0.11191857	-0.05234581	0.11321161	-0.17629457	-0.2533085
**Dunn's test to identifiy outlier chromosomes**	1M, 1F																						
	LG01																						
	LG02	ns																					
	LG03	ns	ns																				
	LG04	**	*	*																			
	LG05	ns	ns	ns	***																		
	LG06	ns	ns	ns	ns	ns																	
	LG07	ns	ns	ns	***	ns	ns																
	LG08	ns	ns	ns	*	ns	ns	ns															
	LG09	ns	ns	ns	ns	ns	ns	ns	ns														
	LG10	ns	ns	ns	*	ns	ns	ns	ns	ns													
	LG11	ns	ns	ns	**	ns	ns	ns	ns	ns	ns												
	LG12	ns	ns	ns	ns	*	ns	*	ns	ns	ns	ns											
	LG13	****	****	****	ns	****	***	****	****	***	****	****	*										
	LG14	ns	ns	ns	ns	ns	ns	ns	ns	ns	ns	ns	ns	***									
	LG15	ns	ns	ns	*	ns	ns	ns	ns	ns	ns	ns	ns	****	ns								
	LG16	ns	ns	ns	*	ns	ns	ns	ns	ns	ns	ns	ns	****	ns	ns							
	LG17	ns	ns	ns	ns	*	ns	*	ns	ns	ns	ns	ns	**	ns	ns	ns						
	LG18	ns	ns	ns	*	ns	ns	ns	ns	ns	ns	ns	ns	****	ns	ns	ns	ns					
	LG19	ns	ns	ns	ns	ns	ns	ns	ns	ns	ns	ns	ns	**	ns	ns	ns	ns	ns				
	LG20	ns	ns	ns	ns	*	ns	*	ns	ns	ns	ns	ns	*	ns	ns	ns	ns	ns	ns			
	LG22	ns	ns	ns	****	ns	**	ns	ns	ns	ns	ns	***	****	**	ns	ns	***	ns	*	**		
	LG23	ns	ns	ns	****	ns	*	ns	ns	ns	ns	ns	**	****	*	ns	ns	**	ns	ns	*	ns	

Petrochromis famula		LG01	LG02	LG03	LG04	LG05	LG06	LG07	LG08	LG09	LG10	LG11	LG12	LG13	LG14	LG15	LG16	LG17	LG18	LG19	LG20	LG22	LG23
**Sex-specific SNP inspection**																							
Individual	1M, 1F																						
Transcriptome	3M, 3F																						
Pooled		[redacted]																					
**Kruskal-Wallis for heterogeneity among chromosomes, df = 21**																							
	1M, 1F																						
	chi-squared = 135.37, p-value < 2.2e-16																						
**Top 1% (# windows per chromosome)**																							
	1M, 1F																						
XY		0	2	8	2	8	3	0	0	0	0	1	1	0	0	0	0	0	4	19	0	4	1
ZW		0	0	12	0	0	3	1	0	2	1	2	1	0	0	0	0	1	0	0	0	4	0
**mean per chromosome log2(XY:ZW) ratio of SNP density**																							
	1M, 1F	-0.1612323	0.05263815	-0.04578219	-0.17115066	0.52094129	-0.23838237	-0.18654624	-0.3254551	0.04263553	-0.04189948	-0.10934039	0.0535841	-0.07758475	0.01759822	0.06388001	0.14361023	-0.20726597	0.05901171	0.92705933	0.75498898	-0.24265895	0.00969703
**Dunn's test to identifiy outlier chromosomes**	1M, 1F																						
	LG01																						
	LG02	ns																					
	LG03	ns	ns																				
	LG04	ns	ns	ns																			
	LG05	****	*	*	***																		
	LG06	ns	*	*	ns	****																	
	LG07	ns	ns	ns	ns	****	ns																
	LG08	ns	*	*	ns	****	ns	ns															
	LG09	ns	ns	ns	ns	ns	*	ns	*														
	LG10	ns	ns	ns	ns	*	ns	ns	ns	ns													
	LG11	ns	ns	ns	ns	**	ns	ns	ns	ns	ns												
	LG12	ns	ns	ns	ns	ns	*	ns	*	ns	ns	ns											
	LG13	ns	ns	ns	ns	**	ns	ns	ns	ns	ns	ns	ns										
	LG14	ns	ns	ns	ns	*	*	ns	*	ns	ns	ns	ns	ns									
	LG15	*	ns	ns	ns	ns	**	*	**	ns	ns	ns	ns	ns	ns								
	LG16	*	ns	ns	*	ns	**	*	**	ns	ns	ns	ns	ns	ns	ns							
	LG17	ns	ns	ns	ns	****	ns	ns	ns	ns	ns	ns	ns	ns	ns	*	*						
	LG18	ns	ns	ns	ns	ns	*	ns	*	ns	ns	ns	ns	ns	ns	ns	ns	ns					
	LG19	****	****	****	****	*	****	****	****	****	****	****	****	****	****	****	****	****	****				
	LG20	ns	*	*	ns	****	ns	ns	ns	*	ns	ns	*	ns	*	**	**	ns	*	****			
	LG22	ns	ns	ns	ns	***	ns	ns	ns	ns	ns	ns	ns	ns	ns	ns	ns	ns	ns	****	ns		
	LG23	ns	ns	ns	ns	*	*	ns	*	ns	ns	ns	ns	ns	ns	ns	ns	ns	ns	****	*	ns	

Limnotilapia dardennii		LG01	LG02	LG03	LG04	LG05	LG06	LG07	LG08	LG09	LG10	LG11	LG12	LG13	LG14	LG15	LG16	LG17	LG18	LG19	LG20	LG22	LG23
**Sex-specific SNP inspection**																							
Individual	1M, 1F																						
Transcriptome																							
Pooled																							
**Kruskal-Wallis for heterogeneity among chromosomes, df = 21**																							
	1M, 1F																						
	chi-squared = 48.865, p-value < 0.0005236																						
**Top 1% (# windows per chromosome)**																							
	1M, 1F																						
XY		0	3	8	4	0	0	0	0	4	5	3	8	1	0	0	0	1	3	0	0	8	0
ZW		0	1	5	1	1	1	0	1	1	0	2	9	1	1	1	0	0	0	0	0	3	2
**mean per chromosome log2(XY:ZW) ratio of SNP density**																							
	1M, 1F	0.004417448	-0.13131389	0.016058798	0.088969594	-0.08391728	0.008843956	0.212611533	-0.01811804	-0.06047111	-0.06705849	-0.00891112	0.008677657	0.036077294	0.045031463	-0.08696407	-0.01265924	0.007918917	-0.00983521	-0.04537222	-0.21024984	-0.08942634	0.045769173
**Dunn's test to identifiy outlier chromosomes**	1M, 1F																						
	LG01																						
	LG02	ns																					
	LG03	ns	ns																				
	LG04	ns	ns	ns																			
	LG05	ns	ns	ns	ns																		
	LG06	ns	ns	ns	ns	ns																	
	LG07	ns	**	ns	ns	**	ns																
	LG08	ns	ns	ns	ns	ns	ns	ns															
	LG09	ns	ns	ns	ns	ns	ns	*	ns														
	LG10	ns	ns	ns	ns	ns	ns	ns	ns	ns													
	LG11	ns	ns	ns	ns	ns	ns	ns	ns	ns	ns												
	LG12	ns	ns	ns	ns	ns	ns	ns	ns	ns	ns	ns											
	LG13	ns	ns	ns	ns	ns	ns	ns	ns	ns	ns	ns	ns										
	LG14	ns	ns	ns	ns	ns	ns	ns	ns	ns	ns	ns	ns	ns									
	LG15	ns	ns	ns	ns	ns	ns	*	ns	ns	ns	ns	ns	ns	ns								
	LG16	ns	ns	ns	ns	ns	ns	*	ns	ns	ns	ns	ns	ns	ns	ns							
	LG17	ns	ns	ns	ns	ns	ns	ns	ns	ns	ns	ns	ns	ns	ns	ns	ns						
	LG18	ns	ns	ns	ns	ns	ns	ns	ns	ns	ns	ns	ns	ns	ns	ns	ns	ns					
	LG19	ns	ns	ns	ns	ns	ns	ns	ns	ns	ns	ns	ns	ns	ns	ns	ns	ns	ns				
	LG20	ns	ns	ns	ns	ns	ns	*	ns	ns	ns	ns	ns	ns	ns	ns	ns	ns	ns	ns			
	LG22	ns	ns	ns	*	ns	ns	**	ns	ns	ns	ns	ns	ns	ns	ns	ns	ns	ns	ns	ns		
	LG23	ns	ns	ns	ns	ns	ns	ns	ns	ns	ns	ns	ns	ns	ns	ns	ns	ns	ns	ns	ns	ns	

Shuja horei		LG01	LG02	LG03	LG04	LG05	LG06	LG07	LG08	LG09	LG10	LG11	LG12	LG13	LG14	LG15	LG16	LG17	LG18	LG19	LG20	LG22	LG23
**Sex-specific SNP inspection**																							
Individual	1M, 1F																						
Transcriptome																							
Pooled	25M, 25F																						
**Kruskal-Wallis for heterogeneity among chromosomes, df = 21**																							
	1M, 1F																						
	chi-squared = 28.699, p-value < 0.1214																						
	25M, 25F																						
	chi-squared = 16.897, p-value = 0.7173																						
**Top 1% (# windows per chromosome)**																							
	1M, 1F																						
XY		1	1	8	0	0	3	0	1	1	0	2	0	1	0	1	0	0	5	1	0	3	1
ZW		1	5	12	6	0	0	0	1	1	3	5	2	2	1	1	0	0	1	1	0	4	3
	25M, 25F																						
XY		0	0	19	1	1	1	0	0	1	1	2	3	0	0	0	1	0	1	0	2	5	2
ZW		0	0	14	3	0	2	1	0	0	1	4	0	1	0	1	2	0	0	0	1	5	2
**mean per chromosome log2(XY:ZW) ratio of SNP density**																							
	1M, 1F	0.02686779	-0.05266776	0.07176064	0.00682543	0.14007964	-0.08366033	-0.20083164	-0.04816732	-0.09344734	-0.08089556	-0.03243535	-0.09478838	0.066749	0.09012635	-0.06767505	0.00493287	-0.05229694	0.02386329	0.08230792	-0.08985437	-0.12351962	-0.12694663
	25M, 25F	0.04727413	0.08849315	0.11372084	0.06468349	0.02296303	-0.02519691	0.12457987	-0.04915565	0.10038783	0.04552293	-0.01915384	0.1125893	0.02057856	0.06504678	0.03318058	0.09266498	0.07207394	0.14113329	0.11162104	0.09536895	0.06742899	0.0243562

Gnathochromis pfefferi		LG01	LG02	LG03	LG04	LG05	LG06	LG07	LG08	LG09	LG10	LG11	LG12	LG13	LG14	LG15	LG16	LG17	LG18	LG19	LG20	LG22	LG23
**Sex-specific SNP inspection**																							
Individual	1M, 1F																						
Transcriptome																							
Pooled	23M, 25F																						
**Kruskal-Wallis for heterogeneity among chromosomes, df = 21**																							
	1M, 1F																						
	chi-squared = 435.15, p-value < 2.2e-16																						
	23M, 25F																						
	chi-squared = 472.63, p-value < 2.2e-16																						
**Top 1% (# windows per chromosome)**																							
	1M, 1F																						
XY		0	1	1	0	0	0	0	0	0	0	42	0	0	0	30	0	0	0	0	0	0	0
ZW		0	1	1	0	0	0	0	0	0	0	0	0	0	0	1	0	0	0	0	0	0	0
	23M, 25F																						
XY		0	0	0	0	0	0	0	0	0	0	43	0	0	0	34	0	0	0	0	0	0	0
ZW		0	0	0	0	0	0	0	0	0	0	0	0	0	0	0	0	0	0	0	0	0	0
**mean per chromosome log2(XY:ZW) ratio of SNP density**																							
	1M, 1F	-0.0040927	-0.19198	-0.0572042	0.1553244	-0.11228	0.0671466	-0.01349	-0.044949	0.052005	0.230076	1.667838	-0.22307	-0.103063	0.155791	1.7238181	-0.00989	0.0814072	-0.02233	-0.08399	0.16687	-0.0035	-0.059354
	23M, 25F	0.1249186	0.102177	-0.0539422	0.0574772	-0.03849	0.096668	-0.007671	-0.044747	0.07653	-0.01653	2.822629	0.090778	0.04102	0.111605	2.9428656	0.0571178	0.0953469	-0.07069	-0.10096	0.05036	0.05332	0.1819789
**Dunn's test to identifiy outlier chromosomes**	1M, 1F																						
	LG01																						
	LG02	ns																					
	LG03	ns	ns																				
	LG04	ns	**	ns																			
	LG05	ns	ns	ns	*																		
	LG06	ns	*	ns	ns	ns																	
	LG07	ns	ns	ns	ns	ns	ns																
	LG08	ns	ns	ns	ns	ns	ns	ns															
	LG09	ns	*	ns	ns	ns	ns	ns	ns														
	LG10	*	****	**	ns	**	ns	**	*	ns													
	LG11	****	****	****	****	****	****	****	****	****	****												
	LG12	ns	ns	ns	**	ns	ns	ns	ns	*	***	****											
	LG13	ns	ns	ns	*	ns	ns	ns	ns	ns	**	****	ns										
	LG14	ns	**	ns	ns	*	ns	ns	ns	ns	ns	****	**	*									
	LG15	****	****	****	****	****	****	****	****	****	****	ns	****	****	****								
	LG16	ns	ns	ns	ns	ns	ns	ns	ns	ns	ns	****	ns	ns	ns	****							
	LG17	ns	ns	ns	ns	ns	ns	ns	ns	ns	ns	****	ns	ns	ns	****	ns						
	LG18	ns	ns	ns	ns	ns	ns	ns	ns	ns	ns	****	ns	ns	ns	****	ns	ns					
	LG19	ns	ns	ns	ns	ns	ns	ns	ns	ns	*	****	ns	ns	ns	****	ns	ns	ns				
	LG20	ns	*	ns	ns	ns	ns	ns	ns	ns	ns	****	ns	ns	ns	****	ns	ns	ns	ns			
	LG22	ns	ns	ns	ns	ns	ns	ns	ns	ns	*	****	ns	ns	ns	****	ns	ns	ns	ns	ns		
	LG23	ns	ns	ns	ns	ns	ns	ns	ns	ns	*	****	ns	ns	ns	****	ns	ns	ns	ns	ns	ns	
**Dunn's test to identifiy outlier chromosomes**	23M, 25F																						
	LG01																						
	LG02	ns																					
	LG03	ns	ns																				
	LG04	ns	ns	ns																			
	LG05	ns	ns	ns	ns																		
	LG06	ns	ns	ns	ns	ns																	
	LG07	ns	ns	ns	ns	ns	ns																
	LG08	ns	ns	ns	ns	ns	ns	ns															
	LG09	ns	ns	ns	ns	ns	ns	ns	ns														
	LG10	ns	ns	ns	ns	ns	ns	ns	ns	ns													
	LG11	****	****	****	****	****	****	****	****	****	****												
	LG12	ns	ns	ns	ns	ns	ns	ns	ns	ns	ns	****											
	LG13	ns	ns	ns	ns	ns	ns	ns	ns	ns	ns	****	ns										
	LG14	ns	ns	ns	ns	ns	ns	ns	ns	ns	ns	****	ns	ns									
	LG15	****	****	****	****	****	****	****	****	****	****	ns	****	****	****								
	LG16	ns	ns	ns	ns	ns	ns	ns	ns	ns	ns	****	ns	ns	ns	****							
	LG17	ns	ns	ns	ns	ns	ns	ns	ns	ns	ns	****	ns	ns	ns	****	ns						
	LG18	ns	ns	ns	ns	ns	ns	ns	ns	ns	ns	****	ns	ns	ns	****	ns	ns					
	LG19	ns	ns	ns	ns	ns	ns	ns	ns	ns	ns	****	ns	ns	ns	****	ns	ns	ns				
	LG20	ns	ns	ns	ns	ns	ns	ns	ns	ns	ns	****	ns	ns	ns	****	ns	ns	ns	ns			
	LG22	ns	ns	ns	ns	ns	ns	ns	ns	ns	ns	****	ns	ns	ns	****	ns	ns	ns	ns	ns		
	LG23	ns	ns	*	ns	ns	ns	ns	ns	ns	ns	****	ns	ns	ns	****	ns	ns	*	ns	ns	ns	

Pseudosimochromis curvifrons		LG01	LG02	LG03	LG04	LG05	LG06	LG07	LG08	LG09	LG10	LG11	LG12	LG13	LG14	LG15	LG16	LG17	LG18	LG19	LG20	LG22	LG23
**Sex-specific SNP inspection**																							
Individual	1M, 1F																						
Transcriptome		[redacted]																					
Pooled		[redacted]																					
**Kruskal-Wallis for heterogeneity among chromosomes, df = 21**		[redacted]																					
	1M, 1F	[redacted]																					
	chi-squared = 276.9, p-value < 2.2e-16	[redacted]																					
**Top 1% (# windows per chromosome)**																							
	1M, 1F																						
XY		0	0	1	0	48	0	0	0	0	0	0	0	0	0	0	0	0	0	28	0	0	0
ZW		0	0	1	0	0	0	0	0	0	0	0	0	0	0	0	0	0	0	0	0	0	0
**mean per chromosome log2(XY:ZW) ratio of SNP density**																							
	1M, 1F	0.18950388	-0.21277469	-0.06991321	-0.02688755	1.58773486	-0.05998271	0.13379068	0.02639306	0.08321766	0.10981476	-0.06010733	0.29724562	0.03506898	-0.0168158	0.18648654	-0.19874658	0.22358311	0.06703867	1.29347429	0.76990484	-0.1401228	-0.37801588
**Dunn's test to identifiy outlier chromosomes**	1M, 1F																						
	LG01																						
	LG02	*																					
	LG03	ns	ns																				
	LG04	ns	ns	ns																			
	LG05	****	****	****	****																		
	LG06	ns	ns	ns	ns	****																	
	LG07	ns	**	ns	ns	****	*																
	LG08	ns	ns	ns	ns	****	ns	ns															
	LG09	ns	ns	ns	ns	****	ns	ns	ns														
	LG10	ns	*	ns	ns	****	ns	ns	ns	ns													
	LG11	ns	ns	ns	ns	****	ns	ns	ns	ns	ns												
	LG12	ns	****	**	*	****	***	ns	*	ns	ns	**											
	LG13	ns	ns	ns	ns	****	ns	ns	ns	ns	ns	ns	ns										
	LG14	ns	ns	ns	ns	****	ns	ns	ns	ns	ns	ns	**	ns									
	LG15	ns	**	ns	ns	****	*	ns	ns	ns	ns	ns	ns	ns	ns								
	LG16	*	ns	ns	ns	****	ns	**	ns	ns	*	ns	****	ns	ns	**							
	LG17	ns	**	ns	ns	****	ns	ns	ns	ns	ns	ns	ns	ns	ns	ns	**						
	LG18	ns	*	ns	ns	****	ns	ns	ns	ns	ns	ns	ns	ns	ns	ns	*	ns					
	LG19	****	****	****	****	ns	****	****	****	****	****	****	**	****	****	****	****	****	****				
	LG20	ns	ns	ns	ns	****	ns	*	ns	ns	ns	ns	**	ns	ns	ns	ns	ns	ns	****			
	LG22	ns	ns	ns	ns	****	ns	*	ns	ns	ns	ns	***	ns	ns	*	ns	ns	ns	****	ns		
	LG23	***	ns	*	**	****	*	****	*	**	***	*	****	**	*	****	ns	****	***	****	ns	ns	

Pseudosimochromis babaulti		LG01	LG02	LG03	LG04	LG05	LG06	LG07	LG08	LG09	LG10	LG11	LG12	LG13	LG14	LG15	LG16	LG17	LG18	LG19	LG20	LG22	LG23
**Sex-specific SNP inspection**																							
Individual	1M, 1F																						
Transcriptome																							
Pooled	25M, 23F																						
**Kruskal-Wallis for heterogeneity among chromosomes, df = 21**																							
	1M, 1F																						
	chi-squared = 367.5, p-value < 2.2e-16																						
	25M, 23F																						
	chi-squared = 169.35, p-value < 2.2e-16																						
**Top 1% (# windows per chromosome)**																							
	1M, 1F																						
XY		0	0	1	1	35	0	0	0	0	0	0	0	0	0	0	0	0	0	28	0	1	0
ZW		0	0	1	0	0	0	0	0	0	0	0	0	0	0	0	0	0	0	0	0	0	0
	25M, 23F																						
XY		0	0	0	0	44	0	0	0	0	0	0	0	0	0	0	0	0	0	33	0	0	0
ZW		0	0	0	0	0	0	0	0	0	0	0	0	0	0	0	0	0	0	0	0	0	0
**mean per chromosome log2(XY:ZW) ratio of SNP density**																							
	1M, 1F	-0.106219	0.171779	0.19382113	0.42088545	1.92204172	0.48918712	0.07579476	0.182021274	0.13078764	-0.03572006	0.2736785	0.2373441	0.05818899	-0.02277383	0.13301504	0.0824552	0.04421562	0.19470589	1.58195742	0.22035585	0.00835024	0.04842894
	25M, 23F	0.06734	0.08587777	0.06727454	-0.02141293	1.48787329	0.00253123	-0.00171122	-3.37324E-05	0.08673893	0.23092569	0.27281274	-0.05537536	-0.12461508	0.0763778	0.09962029	0.22925104	-0.0042409	0.19868684	2.06791767	0.08668005	0.03229258	0.15162995
**Dunn's test to identifiy outlier chromosomes**	1M, 1F																						
	LG01																						
	LG02	**																					
	LG03	***	ns																				
	LG04	****	ns	ns																			
	LG05	****	****	****	****																		
	LG06	****	**	*	ns	****																	
	LG07	*	ns	ns	***	****	****																
	LG08	**	ns	ns	ns	****	**	ns															
	LG09	**	ns	ns	ns	****	*	ns	ns														
	LG10	ns	*	**	****	****	****	ns	*	*													
	LG11	****	ns	ns	ns	****	ns	**	ns	ns	****												
	LG12	****	ns	ns	ns	****	ns	*	ns	ns	***	ns											
	LG13	ns	ns	ns	***	****	****	ns	ns	ns	ns	**	*										
	LG14	ns	ns	*	****	****	****	ns	ns	ns	ns	***	**	ns									
	LG15	*	ns	ns	*	****	***	ns	ns	ns	ns	*	ns	ns	ns								
	LG16	ns	ns	ns	***	****	****	ns	ns	ns	ns	**	*	ns	ns	ns							
	LG17	ns	ns	ns	***	****	****	ns	ns	ns	ns	**	*	ns	ns	ns	ns						
	LG18	***	ns	ns	ns	****	*	ns	ns	ns	**	ns	ns	ns	*	ns	ns	ns					
	LG19	****	****	****	*	***	ns	****	****	***	****	**	***	****	****	****	****	****	***				
	LG20	*	ns	ns	*	****	***	ns	ns	ns	ns	*	ns	ns	ns	ns	ns	ns	ns	****			
	LG22	ns	ns	*	****	****	****	ns	ns	ns	ns	***	**	ns	ns	ns	ns	ns	*	****	ns		
	LG23	**	ns	ns	*	****	***	ns	ns	ns	ns	*	ns	ns	ns	ns	ns	ns	ns	****	ns	ns	
**Dunn's test to identifiy outlier chromosomes**	25M, 23F																						
	LG01																						
	LG02	ns																					
	LG03	ns	ns																				
	LG04	ns	ns	ns																			
	LG05	***	***	**	****																		
	LG06	ns	ns	ns	ns	****																	
	LG07	ns	ns	ns	ns	****	ns																
	LG08	ns	ns	ns	ns	***	ns	ns															
	LG09	ns	ns	ns	ns	*	ns	ns	ns														
	LG10	ns	ns	ns	ns	ns	ns	ns	ns	ns													
	LG11	ns	ns	ns	**	ns	**	**	*	ns	ns												
	LG12	ns	ns	ns	ns	****	ns	ns	ns	ns	ns	**											
	LG13	ns	ns	ns	ns	****	ns	ns	ns	ns	*	***	ns										
	LG14	ns	ns	ns	ns	**	ns	ns	ns	ns	ns	ns	ns	ns									
	LG15	ns	ns	ns	ns	**	ns	ns	ns	ns	ns	ns	ns	ns	ns								
	LG16	ns	ns	ns	*	ns	*	*	ns	ns	ns	ns	*	**	ns	ns							
	LG17	ns	ns	ns	ns	****	ns	ns	ns	ns	ns	*	ns	ns	ns	ns	ns						
	LG18	ns	ns	ns	ns	ns	ns	ns	ns	ns	ns	ns	ns	*	ns	ns	ns	ns					
	LG19	****	****	****	****	****	****	****	****	****	****	****	****	****	****	****	****	****	****				
	LG20	ns	ns	ns	ns	**	ns	ns	ns	ns	ns	ns	ns	ns	ns	ns	ns	ns	ns	****			
	LG22	ns	ns	ns	ns	***	ns	ns	ns	ns	ns	ns	ns	ns	ns	ns	ns	ns	ns	****	ns		
	LG23	ns	ns	ns	ns	**	ns	ns	ns	ns	ns	ns	ns	*	ns	ns	ns	ns	ns	****	ns	ns	

Pseudosimochromis babaulti South		LG01	LG02	LG03	LG04	LG05	LG06	LG07	LG08	LG09	LG10	LG11	LG12	LG13	LG14	LG15	LG16	LG17	LG18	LG19	LG20	LG22	LG23
**Sex-specific SNP inspection**																							
Individual	1M, 1F																						
Transcriptome		[redacted]																					
Pooled		[redacted]																					
**Kruskal-Wallis for heterogeneity among chromosomes, df = 21**		[redacted]																					
	1M, 1F	[redacted]																					
	chi-squared = 383.47, p-value < 2.2e-16	[redacted]																					
**Top 1% (# windows per chromosome)**																							
	1M, 1F																						
XY		0	1	1	0	43	0	0	0	0	0	0	0	0	0	0	0	0	0	30	0	0	0
ZW		0	1	1	0	0	0	0	0	0	0	0	0	0	0	0	0	0	0	0	0	0	0
**mean per chromosome log2(XY:ZW) ratio of SNP density**																							
	1M, 1F	-0.17369321	0.32417247	-0.22982893	0.26328448	1.60868792	0.20940354	0.26496221	0.26017514	0.50353662	-0.08464942	-0.24109965	-0.06861907	-0.47910512	0.76490283	-0.01779517	0.10165867	0.17003146	-0.61221632	1.64026959	0.03446665	-0.09978773	-0.40013208
**Dunn's test to identifiy outlier chromosomes**	1M, 1F																						
	LG01																						
	LG02	*																					
	LG03	ns	**																				
	LG04	*	ns	**																			
	LG05	****	****	****	****																		
	LG06	ns	ns	**	ns	****																	
	LG07	*	ns	***	ns	****	ns																
	LG08	*	ns	**	ns	****	ns	ns															
	LG09	***	ns	****	ns	**	ns	ns	ns														
	LG10	ns	*	ns	ns	****	ns	*	ns	**													
	LG11	ns	**	ns	**	****	*	**	**	****	ns												
	LG12	ns	*	ns	*	****	*	**	*	***	ns	ns											
	LG13	*	****	ns	****	****	****	****	***	****	*	ns	ns										
	LG14	****	*	****	*	**	**	**	ns	ns	****	****	****	****									
	LG15	ns	ns	ns	ns	****	ns	ns	ns	**	ns	ns	ns	*	****								
	LG16	ns	ns	*	ns	****	ns	ns	ns	*	ns	*	ns	***	**	ns							
	LG17	ns	ns	**	ns	****	ns	ns	ns	ns	ns	*	ns	***	**	ns	ns						
	LG18	**	****	ns	****	****	****	****	****	****	**	*	*	ns	****	**	****	****					
	LG19	****	****	****	****	ns	****	****	****	****	****	****	****	****	****	****	****	****	****				
	LG20	**	ns	***	ns	****	ns	ns	ns	ns	*	**	**	****	ns	*	ns	ns	****	****			
	LG22	*	****	ns	****	****	****	****	****	****	*	ns	*	ns	****	**	****	****	ns	****	****		
	LG23	ns	****	ns	****	****	****	****	***	****	ns	ns	ns	ns	****	*	***	***	ns	****	****	ns	

Pseudosimochromis marginatus North		LG01	LG02	LG03	LG04	LG05	LG06	LG07	LG08	LG09	LG10	LG11	LG12	LG13	LG14	LG15	LG16	LG17	LG18	LG19	LG20	LG22	LG23
**Sex-specific SNP inspection**																							
Individual	1M, 1F																						
Transcriptome		[redacted]																					
Pooled																							
**Kruskal-Wallis for heterogeneity among chromosomes, df = 21**																							
	1M, 1F																						
	chi-squared = 421.7, p-value < 2.2e-16																						
**Top 1% (# windows per chromosome)**	1M, 1F																						
	1M, 1F																						
XY		0	0	0	0	51	0	0	0	0	0	0	0	0	0	0	0	0	0	24	0	0	0
ZW		0	1	0	0	0	0	0	0	0	0	0	0	0	0	0	0	0	0	0	0	1	0
**mean per chromosome log2(XY:ZW) ratio of SNP density**																							
	1M, 1F	0.25898844	-0.09598054	-0.18202102	-0.10162414	1.72964773	-0.02926145	-0.11422259	-0.24339736	-0.23786677	-0.39474895	0.21143064	0.21844086	-0.1881643	-0.38267826	-0.15930101	-0.24768322	0.16248891	0.32108833	1.40513384	-0.18188418	0.4568237	-0.87955265
**Dunn's test to identifiy outlier chromosomes**	1M, 1F																						
	LG01																						
	LG02	**																					
	LG03	**	ns																				
	LG04	*	ns	ns																			
	LG05	****	****	****	****																		
	LG06	*	ns	ns	ns	****																	
	LG07	**	ns	ns	ns	****	ns																
	LG08	***	ns	ns	ns	****	ns	ns															
	LG09	**	ns	ns	ns	****	ns	ns	ns														
	LG10	****	ns	ns	ns	****	*	ns	ns	ns													
	LG11	ns	*	*	ns	****	ns	*	**	**	***												
	LG12	ns	ns	*	ns	****	ns	*	**	*	***	ns											
	LG13	**	ns	ns	ns	****	ns	ns	ns	ns	ns	*	*										
	LG14	****	ns	ns	*	****	*	ns	ns	ns	ns	***	***	ns									
	LG15	**	ns	ns	ns	****	ns	ns	ns	ns	ns	*	*	ns	ns								
	LG16	***	ns	ns	ns	****	ns	ns	ns	ns	ns	**	**	ns	ns	ns							
	LG17	ns	ns	*	ns	****	ns	*	**	*	***	ns	ns	*	***	*	**						
	LG18	ns	**	**	*	****	*	**	***	**	****	ns	ns	**	****	**	***	ns					
	LG19	****	****	****	****	ns	****	****	****	****	****	****	****	****	****	****	****	****	****				
	LG20	ns	*	**	ns	****	ns	**	**	**	***	ns	ns	*	****	**	**	ns	ns	****			
	LG22	ns	****	****	***	***	****	****	****	****	****	*	*	****	****	****	****	*	ns	**	ns		
	LG23	****	****	****	****	****	****	****	***	***	***	****	****	****	***	****	****	****	****	****	****	****	

Pseudosimochromis marginatus		LG01	LG02	LG03	LG04	LG05	LG06	LG07	LG08	LG09	LG10	LG11	LG12	LG13	LG14	LG15	LG16	LG17	LG18	LG19	LG20	LG22	LG23
**Sex-specific SNP inspection**																							
Individual	1M, 1F																						
Transcriptome		[redacted]																					
Pooled																							
**Kruskal-Wallis for heterogeneity among chromosomes, df = 21**																							
	1M, 1F																						
	chi-squared = 1120.6, p-value < 2.2e-16																						
**Top 1% (# windows per chromosome)**																							
	1M, 1F																						
XY		0	0	0	0	54	0	0	0	0	0	0	0	0	0	0	0	0	0	23	0	0	0
ZW		0	0	0	0	0	0	0	0	0	0	0	0	0	0	0	0	0	0	0	0	0	0
**mean per chromosome log2(XY:ZW) ratio of SNP density**																							
	1M, 1F	-0.67782264	0.39314539	0.03811528	0.25624364	2.92089183	0.01324327	-0.46109332	-1.30899625	0.2933773	-0.2062103	-0.36384241	0.42988837	-1.70999748	-0.69107109	1.77973295	1.52358561	0.83946424	0.1168918	2.96194797	-0.03065856	0.24871353	1.76652652
**Dunn's test to identifiy outlier chromosomes**	1M, 1F																						
	LG01																						
	LG02	****																					
	LG03	****	ns																				
	LG04	****	ns	ns																			
	LG05	****	****	****	****																		
	LG06	***	ns	ns	ns	****																	
	LG07	ns	****	**	****	****	**																
	LG08	*	****	****	****	****	****	***															
	LG09	****	ns	ns	ns	****	ns	***	****														
	LG10	**	**	ns	*	****	ns	ns	****	*													
	LG11	ns	***	*	**	****	ns	ns	***	**	ns												
	LG12	****	ns	*	ns	****	*	****	****	ns	**	****											
	LG13	***	****	****	****	****	****	****	ns	****	****	****	****										
	LG14	ns	****	***	****	****	***	ns	*	****	*	ns	****	***									
	LG15	****	****	****	****	ns	****	****	****	****	****	****	****	****	****								
	LG16	****	****	****	****	**	****	****	****	****	****	****	****	****	****	ns							
	LG17	****	ns	***	*	****	***	****	****	ns	****	****	ns	****	****	****	***						
	LG18	****	ns	ns	ns	****	ns	**	****	ns	ns	*	ns	****	***	****	****	**					
	LG19	****	****	****	****	ns	****	****	****	****	****	****	****	****	****	**	****	****	****				
	LG20	**	*	ns	ns	****	ns	ns	****	ns	ns	ns	**	****	**	****	****	****	ns	****			
	LG22	****	ns	*	ns	****	**	****	****	ns	***	****	ns	****	****	****	****	ns	*	****	**		
	LG23	****	****	****	****	ns	****	****	****	****	****	****	****	****	****	ns	ns	****	****	**	****	****	

Supplemental Table 3. Representative sample of shared k-mers between *Cyprichromis pavo* and *Petrochromis trewavasae*. One million k-mers were sampled randomly and blasted to determine positional information according to the methods in Behrens et al. (2022).

**LG**	**K-mers per chromosome**	**Chromosome length**	**Normalized**
LG1	32,810	38,676,823	0.00084831
LG2	25,074	32,660,920	0.00076771
LG3	26,547	37,314,939	0.00071143
LG4	28,415	30,518,969	0.00093106
LG5	43,834	36,170,306	0.00121188
LG6	33,753	39,774,503	0.00084861
LG7	55,815	64,916,660	0.00085979
LG8	21,311	23,971,387	0.00088902
LG9	18,485	21,023,328	0.00087926
LG10	27,341	32,356,376	0.00084500
LG11	30,804	32,446,080	0.00094939
LG12	32,062	34,086,040	0.00094062
LG13	30,712	32,072,427	0.00095758
LG14	31,630	37,870,038	0.00083522
LG15	29,644	34,548,429	0.00085804
LG16	28,441	34,742,138	0.00081863
LG17	30,754	35,776,103	0.00085962
LG18	27,084	29,505,383	0.00091793
LG19	26,186	25,963,618	0.00100857
LG20	29,234	29,789,237	0.00098136
LG22	30,631	34,725,690	0.00088208
LG23	35,805	42,088,218	0.00085071
		**Mean**	0.00089327
		**STD**	0.00009853

Figures

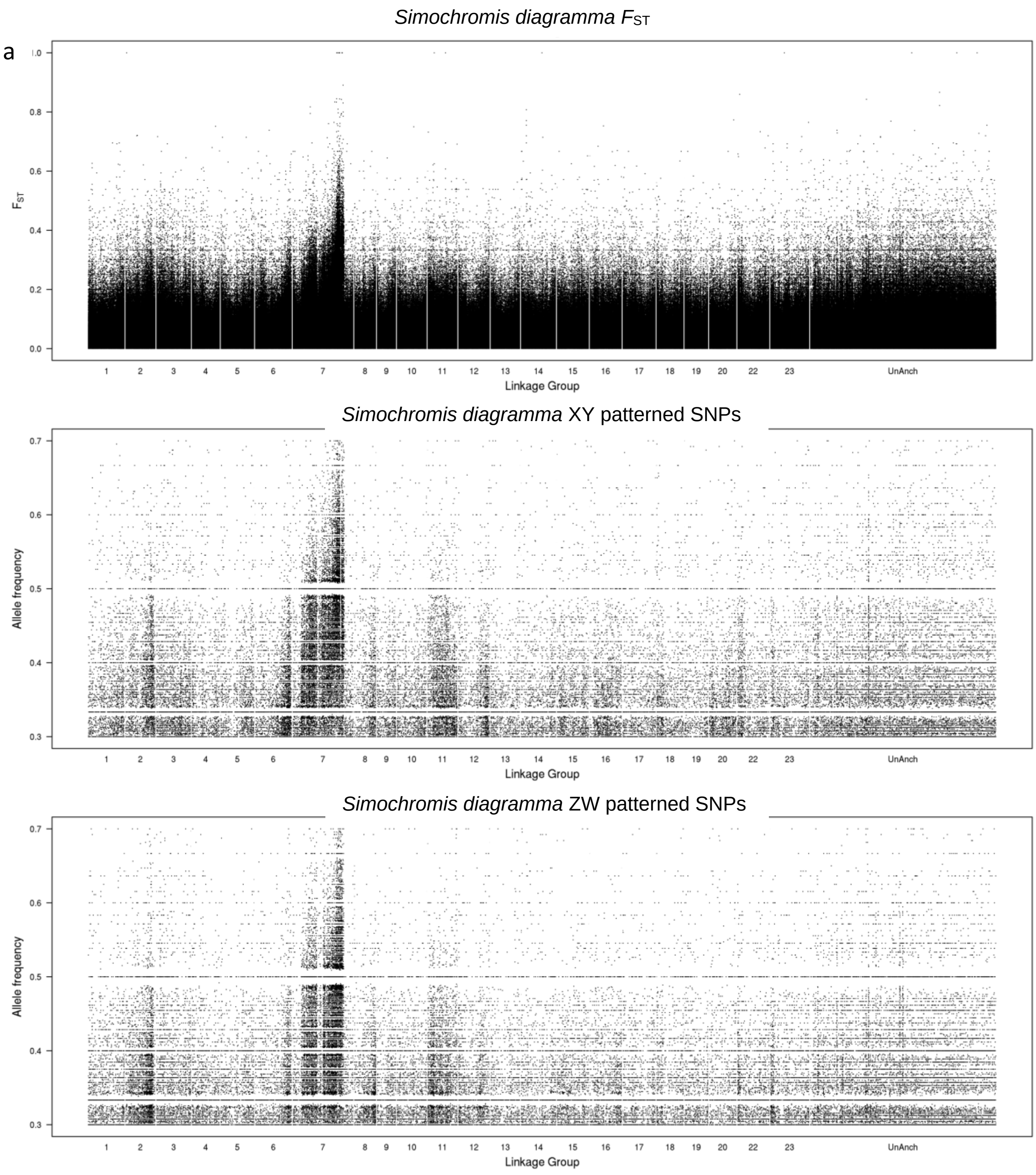

Simochromis diagramma FST
a
FST
1.0
0.8
0.6
0.4
0.2
0.0
1 2 3 4 5 6 7 8 9 10 11 12 13 14 15 16 17 18 19 20 22 23 UnAnch
Linkage Group
Simochromis diagramma XY patterned SNPs
Allele frequency
0.7
0.6
0.5
0.4
0.3
1 2 3 4 5 6 7 8 9 10 11 12 13 14 15 16 17 18 19 20 22 23 UnAnch
Linkage Group
Simochromis diagramma ZW patterned SNPs
Allele frequency
0.7
0.6
0.5
0.4
0.3
1 2 3 4 5 6 7 8 9 10 11 12 13 14 15 16 17 18 19 20 22 23 UnAnch
Linkage Group

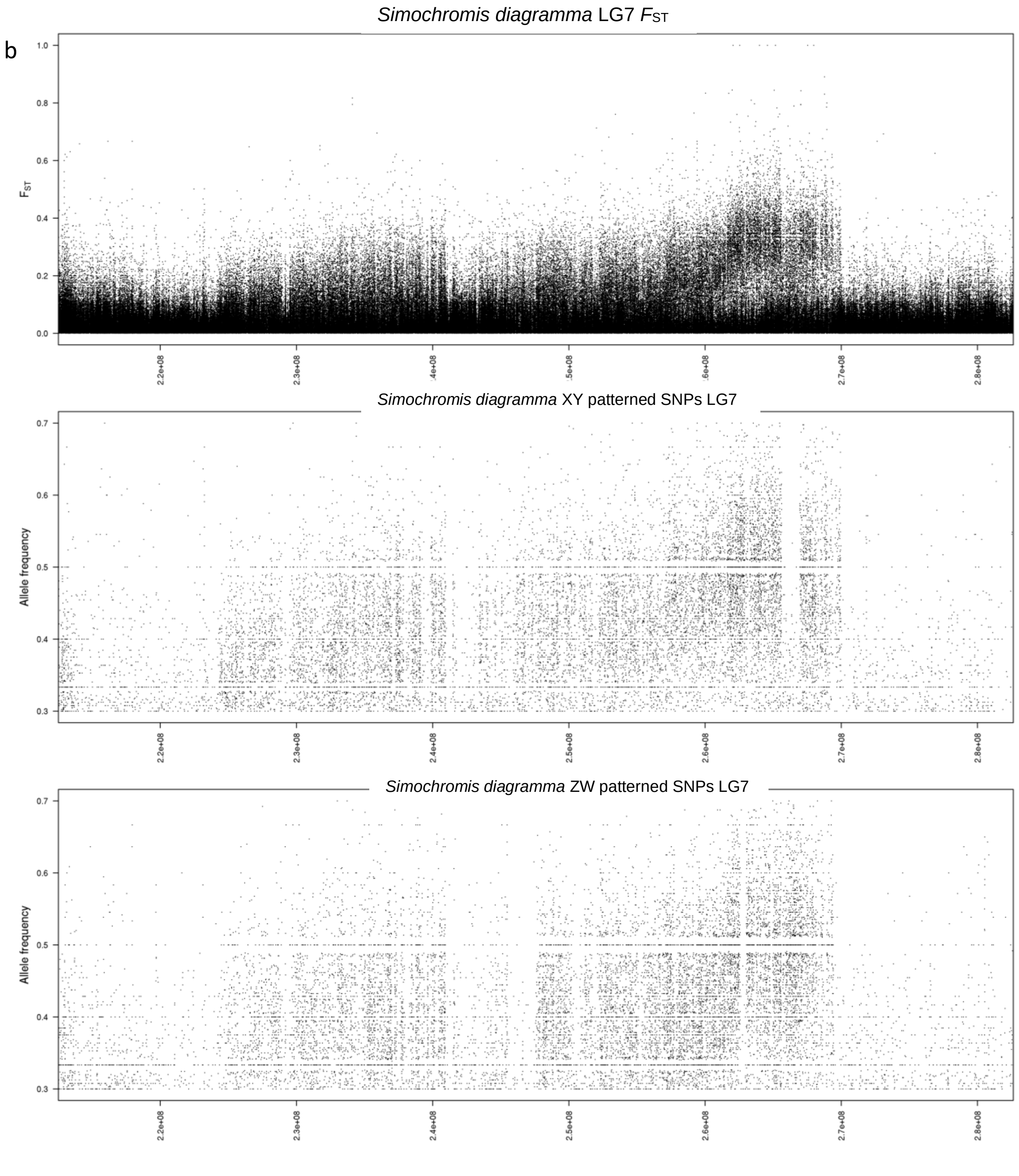

Supplemental Figure 1. Pairwise male x female F_{ST} and sex specific allele frequency for *S. diagramma* a) whole genome plot, b) LG7 plot.

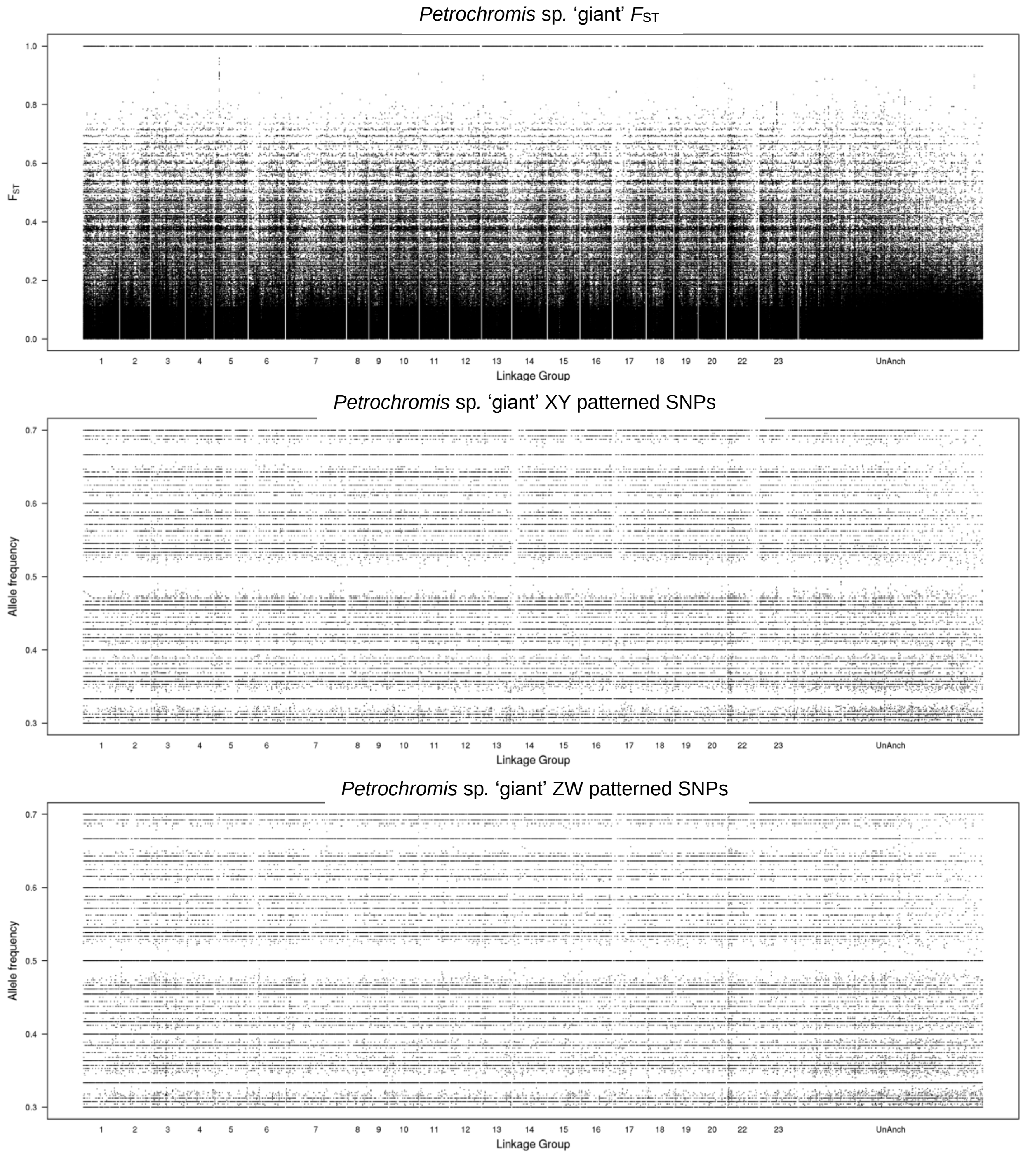

Supplemental Figure 2. Pairwise male x female F_{ST} and sex specific allele frequency for *P.* sp. 'giant' depicting the elevated heterozygosity seen in some species.

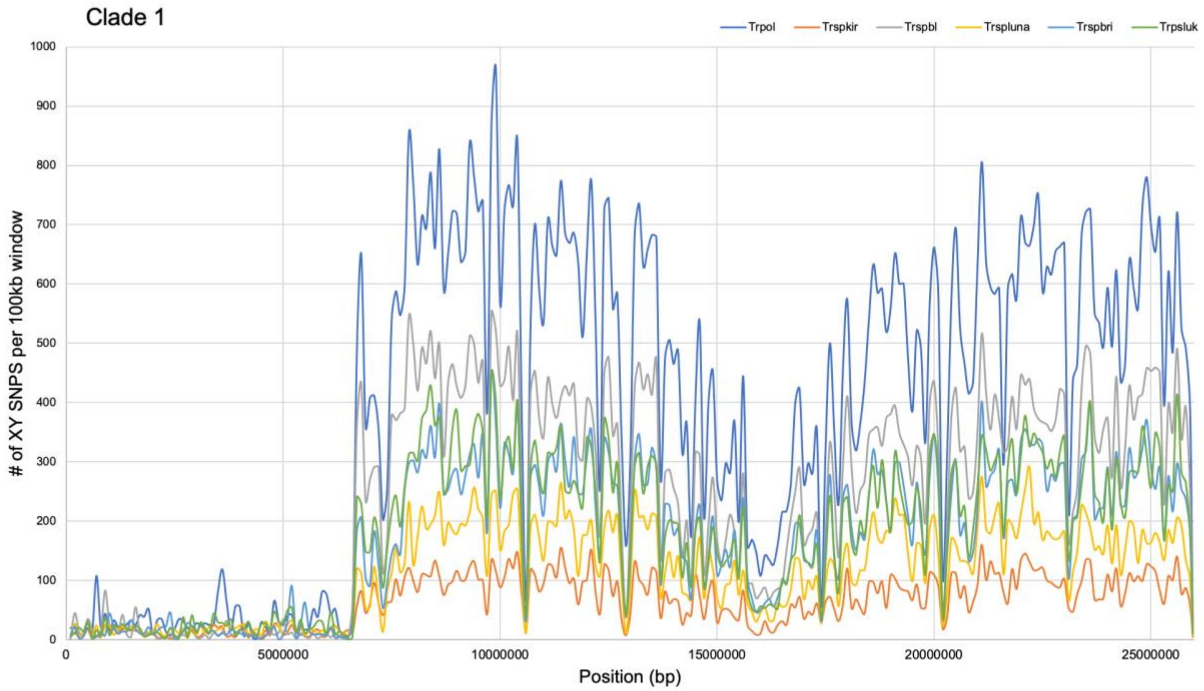

Supplemental Figure 3. Plot of XY SNP density by 100kb window for all Clade 1 species for LG19. For all species the sex-determining region occupies the same region of the chromosome and features no further expansion. The variation in the number of sex-patterned SNPs likely reflect differences in N_e among species.

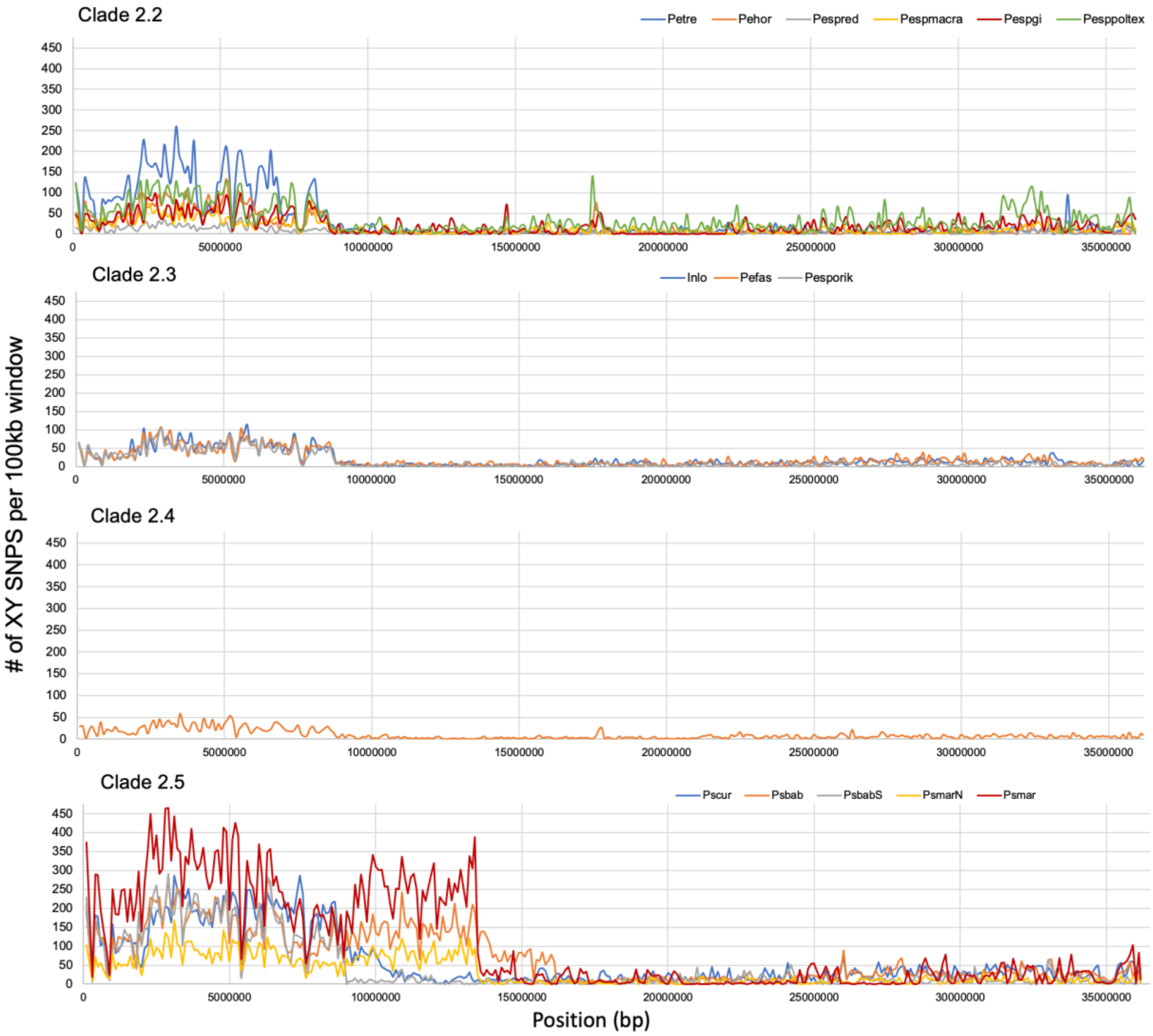

Supplemental Figure 4. Plot of XY SNP density by 100kb window for all Clade 2 species for LG5, demonstrating varying spatial extent of differentiation in the several sub-lineages of Clade 2. Species name abbreviations are Petre = *Petrochromis trewavasae*, Pehor = *Petrochromis horii*, Pespred = *Petrochromis* sp. 'red', Pespmacra = *Petrochromis* sp. 'macrognathus rainbow', Pespgi = *Petrochromis* sp. 'giant', Pesppoltex = *Petrochromis* sp. 'polyodon texas', Inlo = *Interochromis loocki*, Pefas = *Petrochromis fasciolatus*, Pesporik = *Petrochromis* sp. 'orthognathus ikola', Clade 2.4 is *Petrochromis famula*, Pscur = *Pseudosimochromis curvifrons*, Psbab = *Pseudosimochromis babaulti*, PsbabS = *Pseudosimochromis babaulti South*, PsmarN = *Pseudosimochromis marginatus North*, Psmar = *Pseudosimochromis marginatus*.

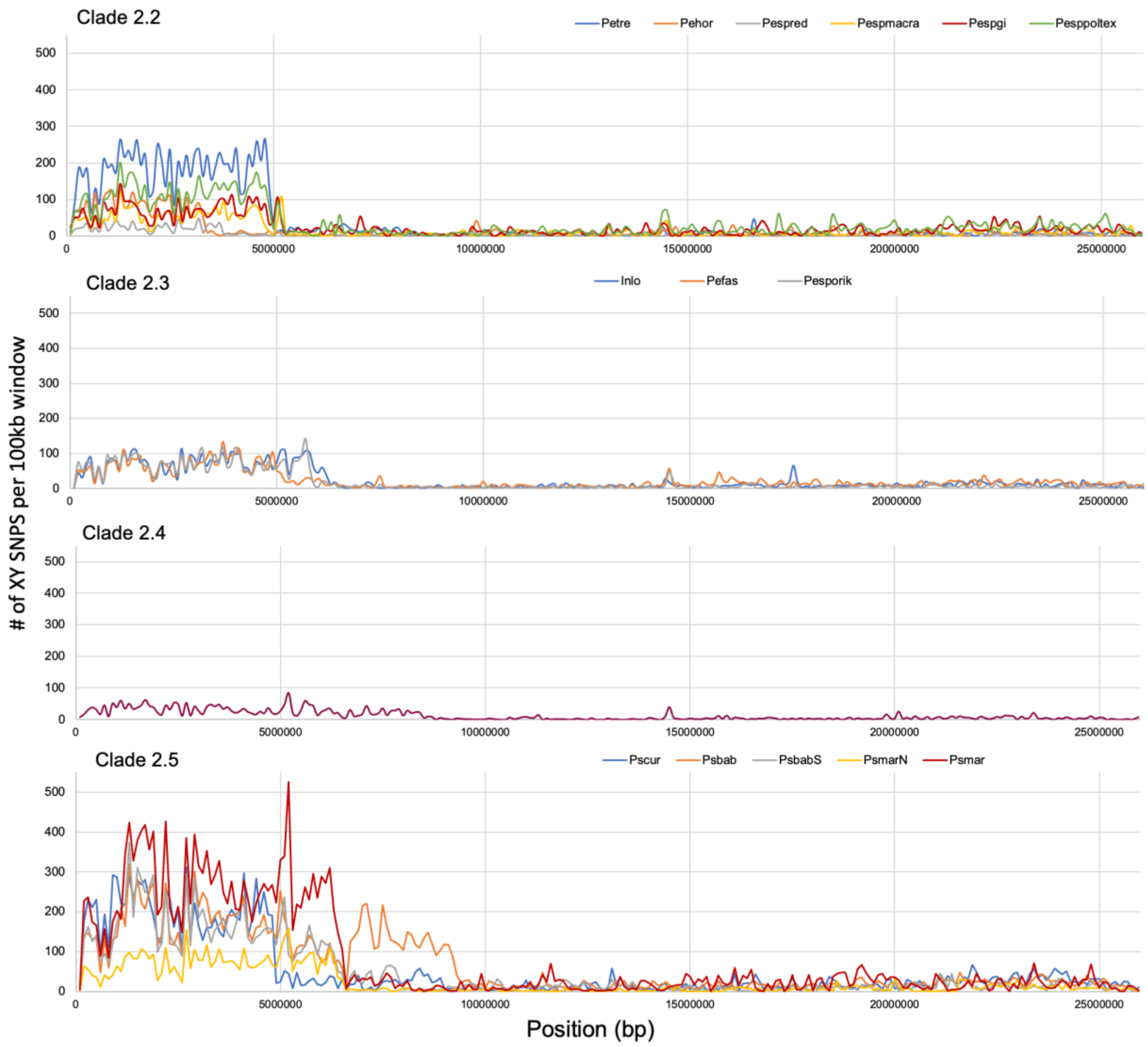

Supplemental Figure 5. Plot of XY SNP density by 100kb window for all Clade 2 species for LG19, demonstrating varying spatial extent of differentiation in the several sub-lineages of Clade 2. Species name abbreviations are Petre = *Petrochromis trewavasae*, Pehor = *Petrochromis horii*, Pespred = *Petrochromis* sp. 'red', Pespmacra = *Petrochromis* sp. 'macrognathus rainbow', Pespgi = *Petrochromis* sp. 'giant', Pesppoltex = *Petrochromis* sp. 'polyodon texas', Inlo = *Interochromis loocki*, Pefas = *Petrochromis fasciolatus*, Pesporik = *Petrochromis* sp. 'orthognathus ikola', Clade 2.4 is *Petrochromis famula*, Pscur = *Pseudosimochromis curvifrons*, Psbab = *Pseudosimochromis babaulti*, PsbabS = *Pseudosimochromis babaulti South*, PsmarN = *Pseudosimochromis marginatus North*, Psmar = *Pseudosimochromis marginatus*.
