# Supplementary material for: Turnover of sex chromosomes in the Lake Tanganyika cichlid tribe Tropheini (Teleostei: Cichlidae)
Source: Sci Rep. 2024 Jan 30;14:2471. doi: 10.1038/s41598-024-53021-3 (PMC10828463; doi:10.1038/s41598-024-53021-3)
Supplement: Supplementary file 2 — Supplementary Information 2. [file 41598_2024_53021_MOESM2_ESM.pdf]

Extended Supplementary Figures

Turnover of Sex Chromosomes in the Lake Tanganyika cichlid tribe Tropheini (Teleostei: *Cichlidae*)

Kristen A. Behrens [1], Holger Zimmermann[2,3], Radim Blažek[2], Martin Reichard[2,4], Stephan Koblmüller [3], Thomas D. Kocher [1]

[1] Department of Biology, University of Maryland, College Park MD 20742 USA

[2] Czech Academy of Sciences, Institute of Vertebrate Biology, Květná 8, 603 00 Brno, Czech Republic

[3] Institute of Biology, University of Graz, Universitätsplatz 2, 8010 Graz, Austria

[4] Department of Ecology and Vertebrate Zoology, University of Łódź, Łódź, Poland

Extended Figures

Pairwise male vs. female F_{ST} and sex specific allele frequency plots for all species that are not featured in the main manuscript or main supplement. All plots are single individual vs. single individual unless headers in plot indicate transcriptome or pool-seq data. All plots use the *M. zebra* UMD2a reference assembly GCF_000238955.4 (Conte 2019).

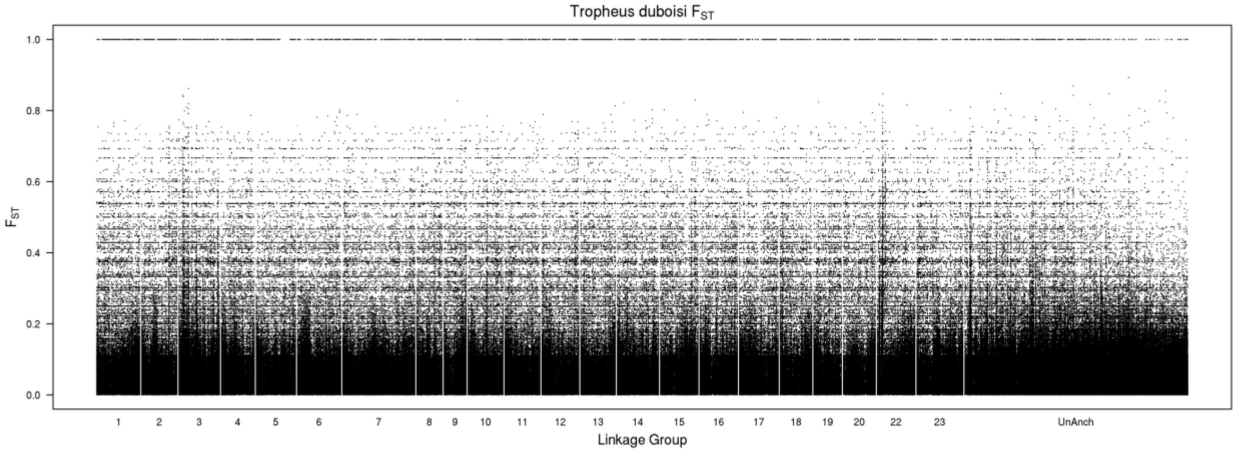

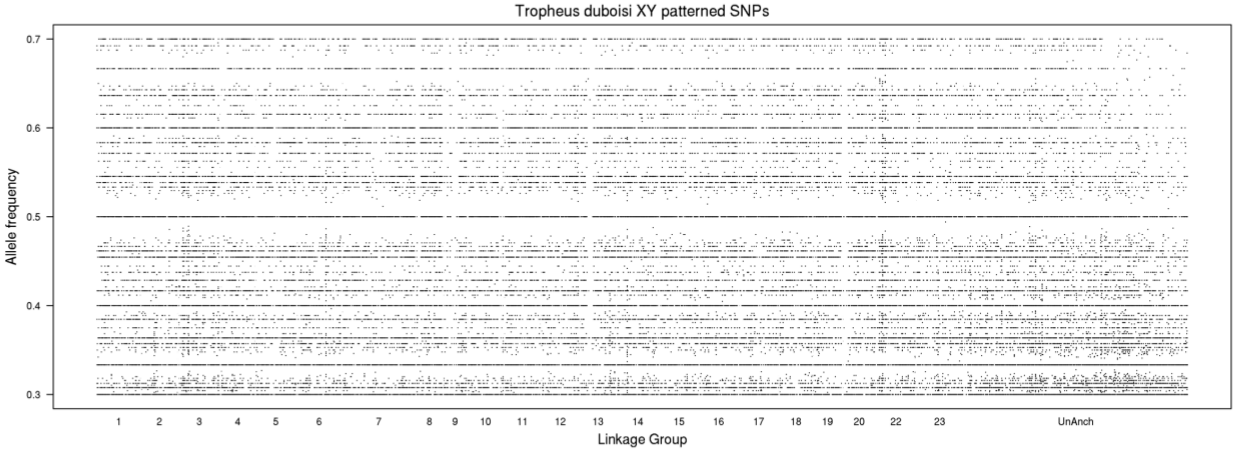

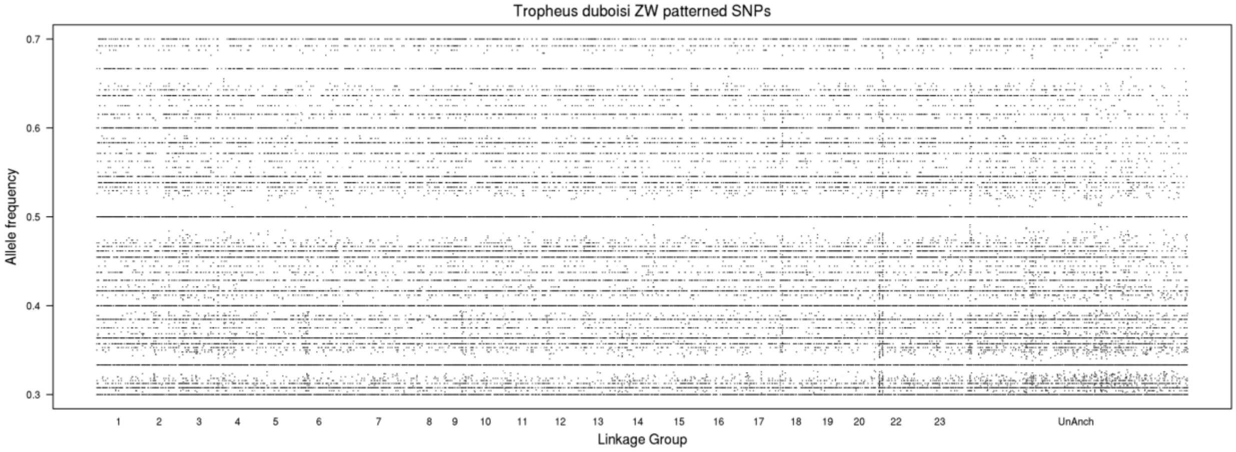

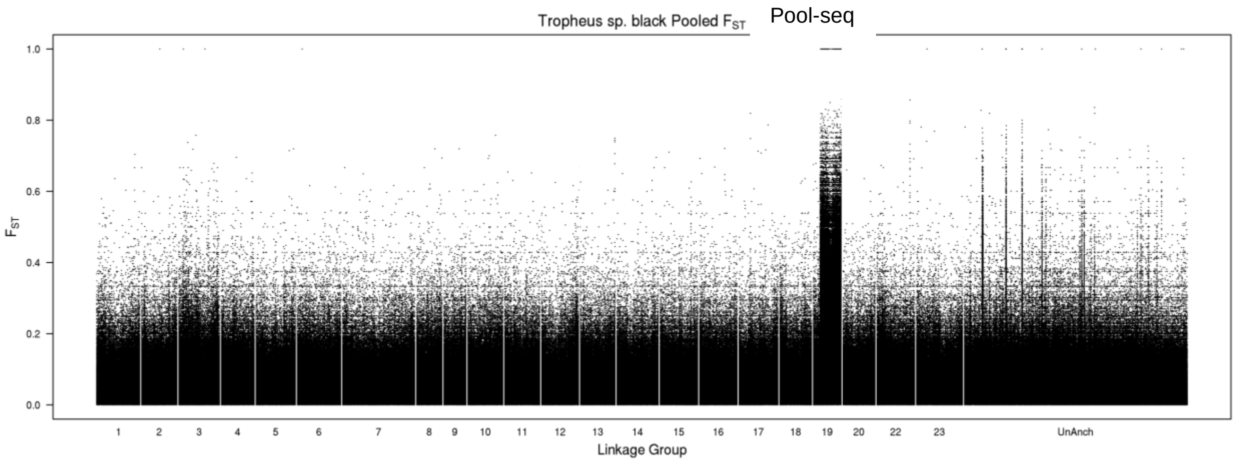
Tropheus sp. black Pooled F_{ST}
Pool-seq
F_{ST}
1.0
0.8
0.6
0.4
0.2
0.0
1 2 3 4 5 6 7 8 9 10 11 12 13 14 15 16 17 18 19 20 22 23 UnAnch
Linkage Group

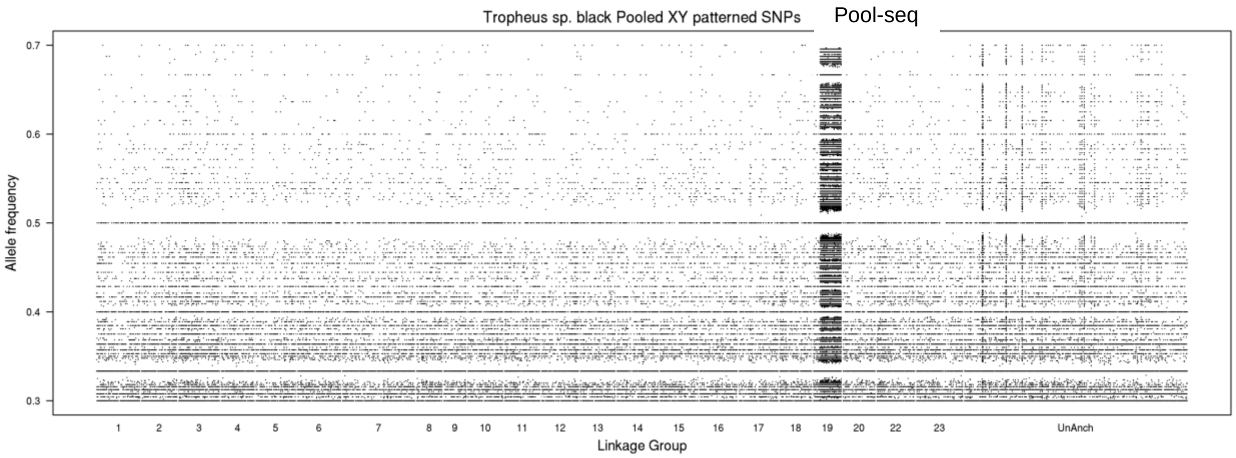
Tropheus sp. black Pooled XY patterned SNPs
Pool-seq
Allele frequency
0.7
0.6
0.5
0.4
0.3
1 2 3 4 5 6 7 8 9 10 11 12 13 14 15 16 17 18 19 20 22 23 UnAnch
Linkage Group

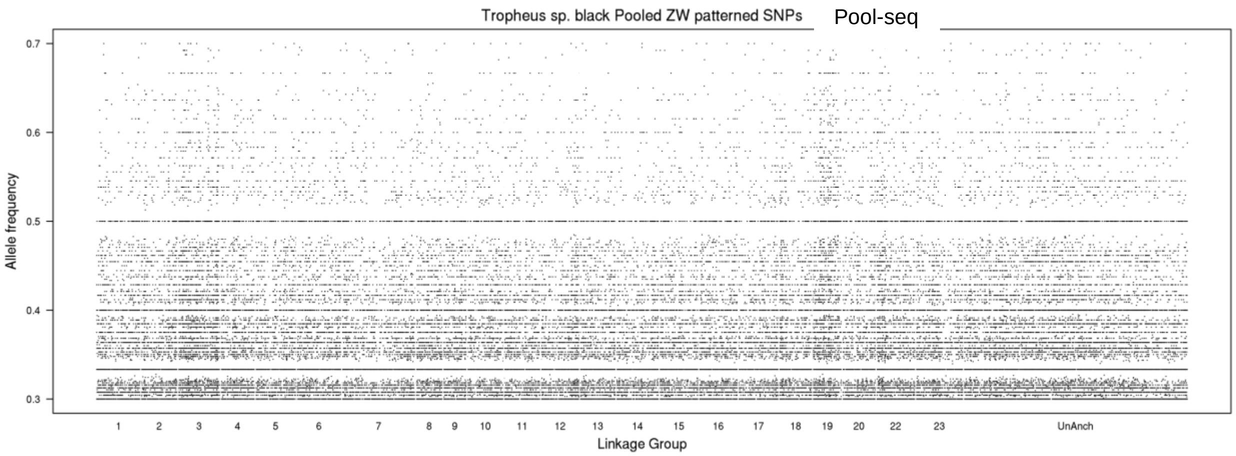
Tropheus sp. black Pooled ZW patterned SNPs
Pool-seq
Allele frequency
0.7
0.6
0.5
0.4
0.3
1 2 3 4 5 6 7 8 9 10 11 12 13 14 15 16 17 18 19 20 22 23 UnAnch
Linkage Group

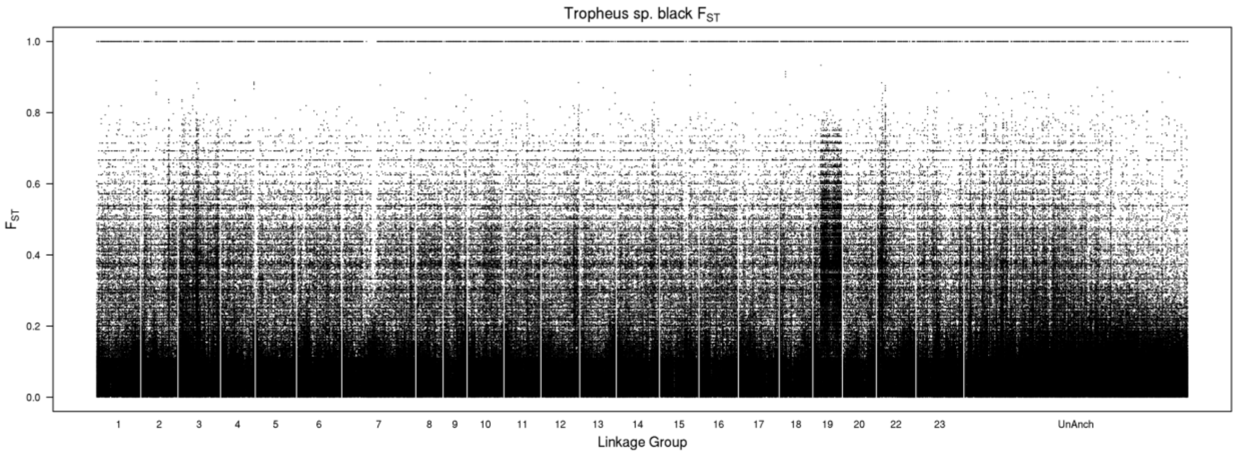
Tropheus sp. black F_{ST}
F_{ST}
1.0
0.8
0.6
0.4
0.2
0.0
1 2 3 4 5 6 7 8 9 10 11 12 13 14 15 16 17 18 19 20 22 23 UnAnch
Linkage Group

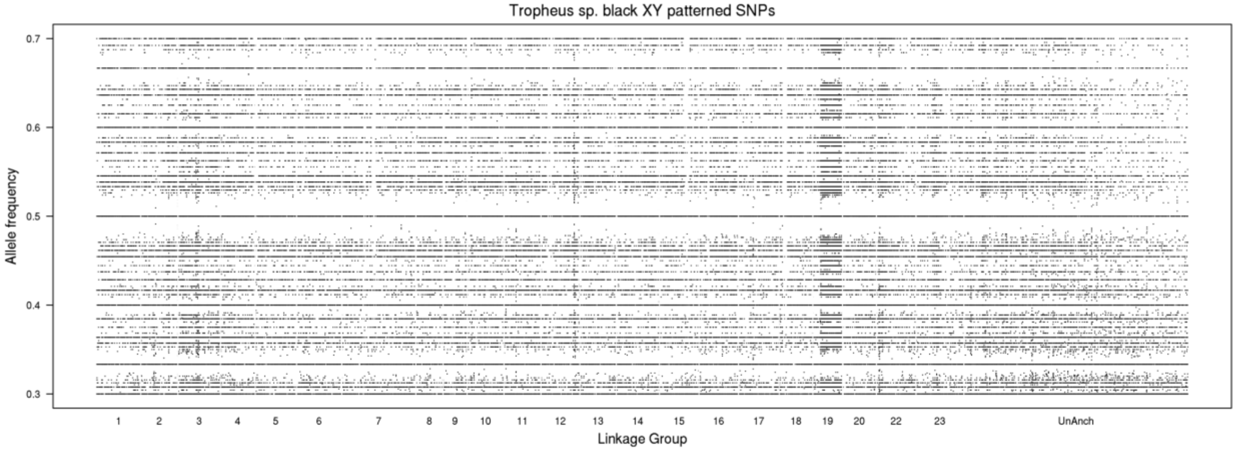
Tropheus sp. black XY patterned SNPs
Allele frequency
0.7
0.6
0.5
0.4
0.3
1 2 3 4 5 6 7 8 9 10 11 12 13 14 15 16 17 18 19 20 22 23 UnAnch
Linkage Group

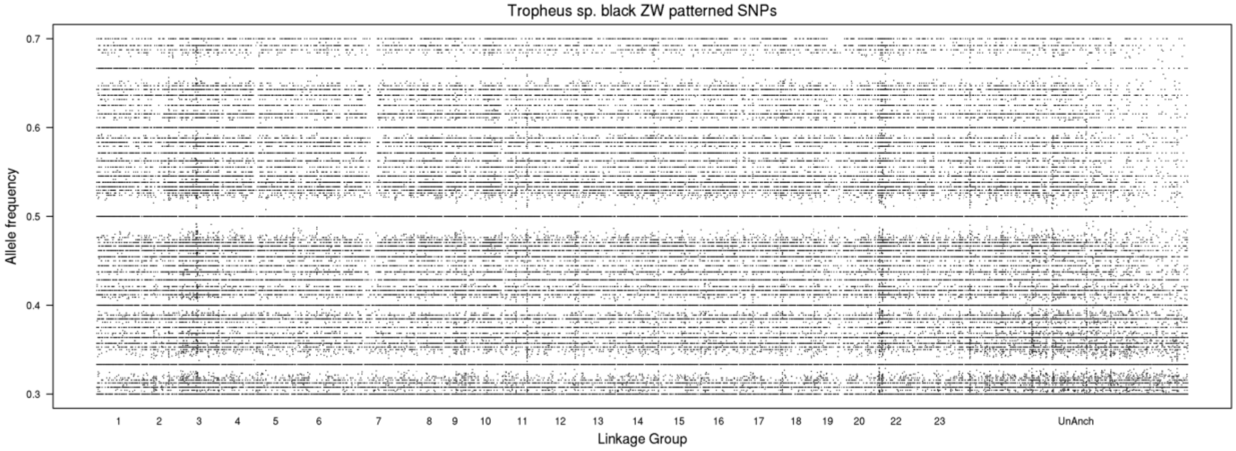
Tropheus sp. black ZW patterned SNPs
Allele frequency
0.7
0.6
0.5
0.4
0.3
1 2 3 4 5 6 7 8 9 10 11 12 13 14 15 16 17 18 19 20 21 22 23 UnAnch
Linkage Group

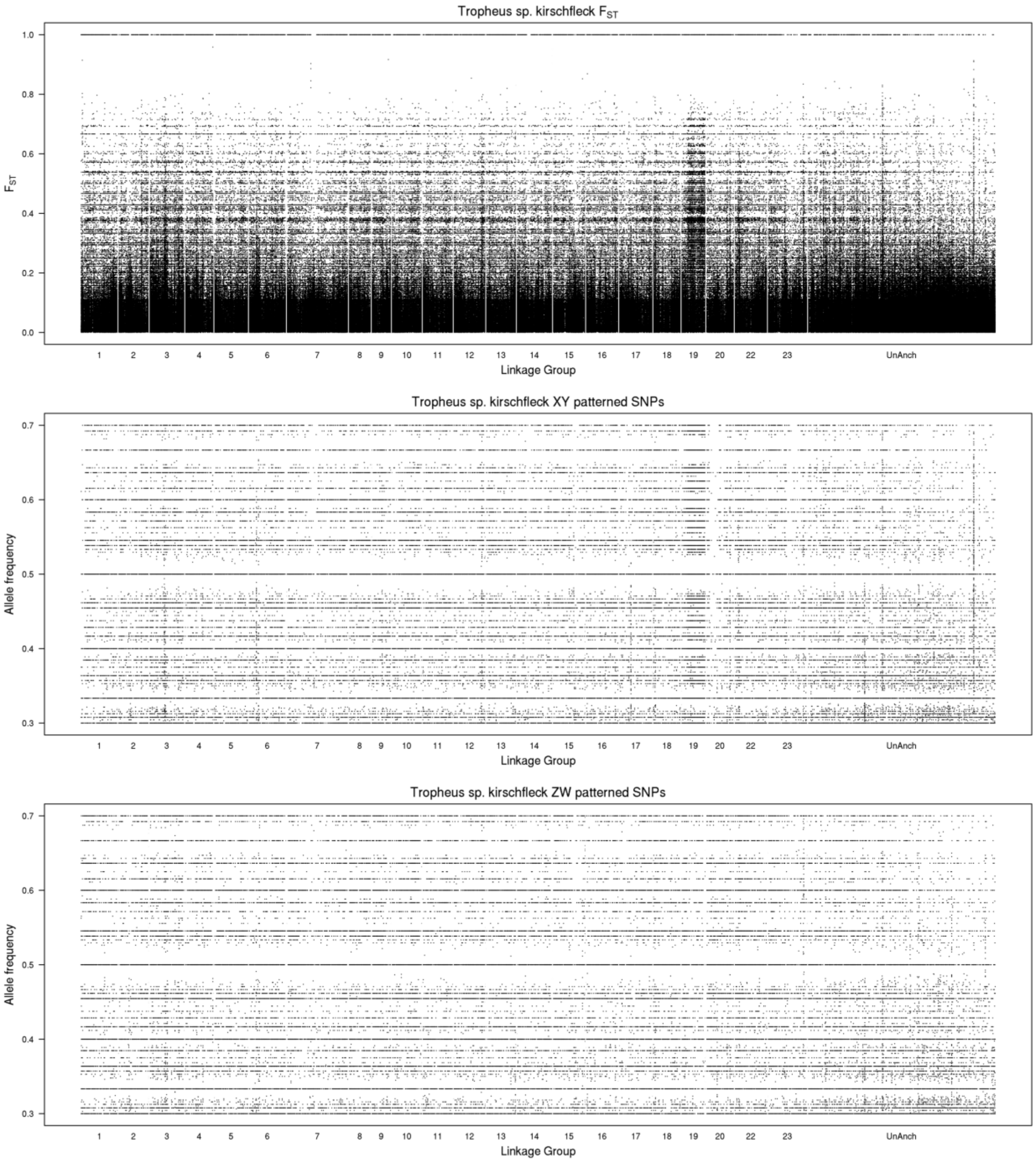
Tropheus sp. kirschfleck F_{ST}
F_{ST}
1.0
0.8
0.6
0.4
0.2
0.0
1 2 3 4 5 6 7 8 9 10 11 12 13 14 15 16 17 18 19 20 22 23 UnAnch
Linkage Group
Tropheus sp. kirschfleck XY patterned SNPs
Allele frequency
0.7
0.6
0.5
0.4
0.3
1 2 3 4 5 6 7 8 9 10 11 12 13 14 15 16 17 18 19 20 22 23 UnAnch
Linkage Group
Tropheus sp. kirschfleck ZW patterned SNPs
Allele frequency
0.7
0.6
0.5
0.4
0.3
1 2 3 4 5 6 7 8 9 10 11 12 13 14 15 16 17 18 19 20 22 23 UnAnch
Linkage Group

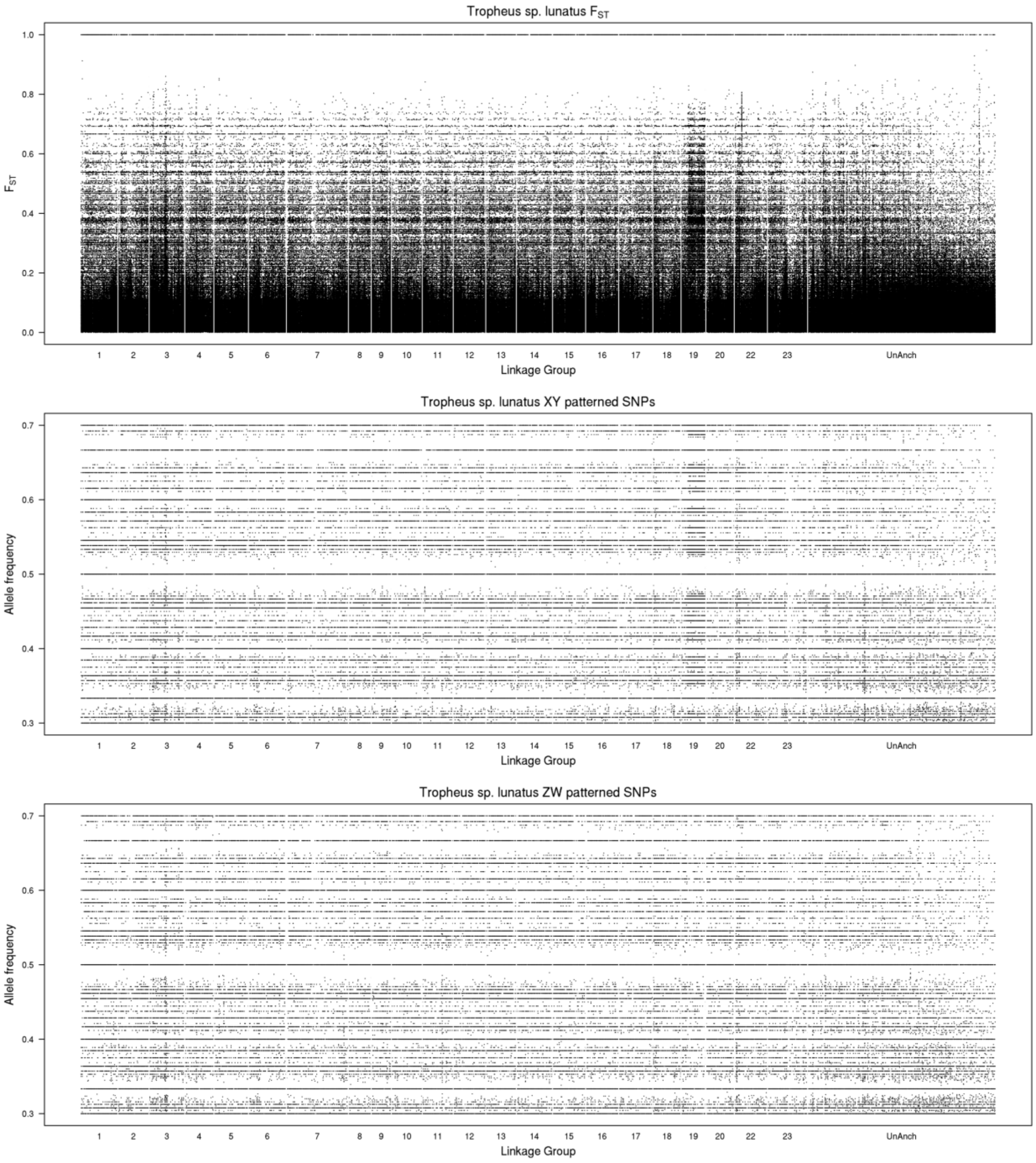

Tropheus sp. lunatus F_{ST}
1.0
0.8
0.6
0.4
0.2
0.0
F_{ST}
1 2 3 4 5 6 7 8 9 10 11 12 13 14 15 16 17 18 19 20 22 23 UnAnch
Linkage Group
Tropheus sp. lunatus XY patterned SNPs
0.7
0.6
0.5
0.4
0.3
Allele frequency
1 2 3 4 5 6 7 8 9 10 11 12 13 14 15 16 17 18 19 20 22 23 UnAnch
Linkage Group
Tropheus sp. lunatus ZW patterned SNPs
0.7
0.6
0.5
0.4
0.3
Allele frequency
1 2 3 4 5 6 7 8 9 10 11 12 13 14 15 16 17 18 19 20 22 23 UnAnch
Linkage Group

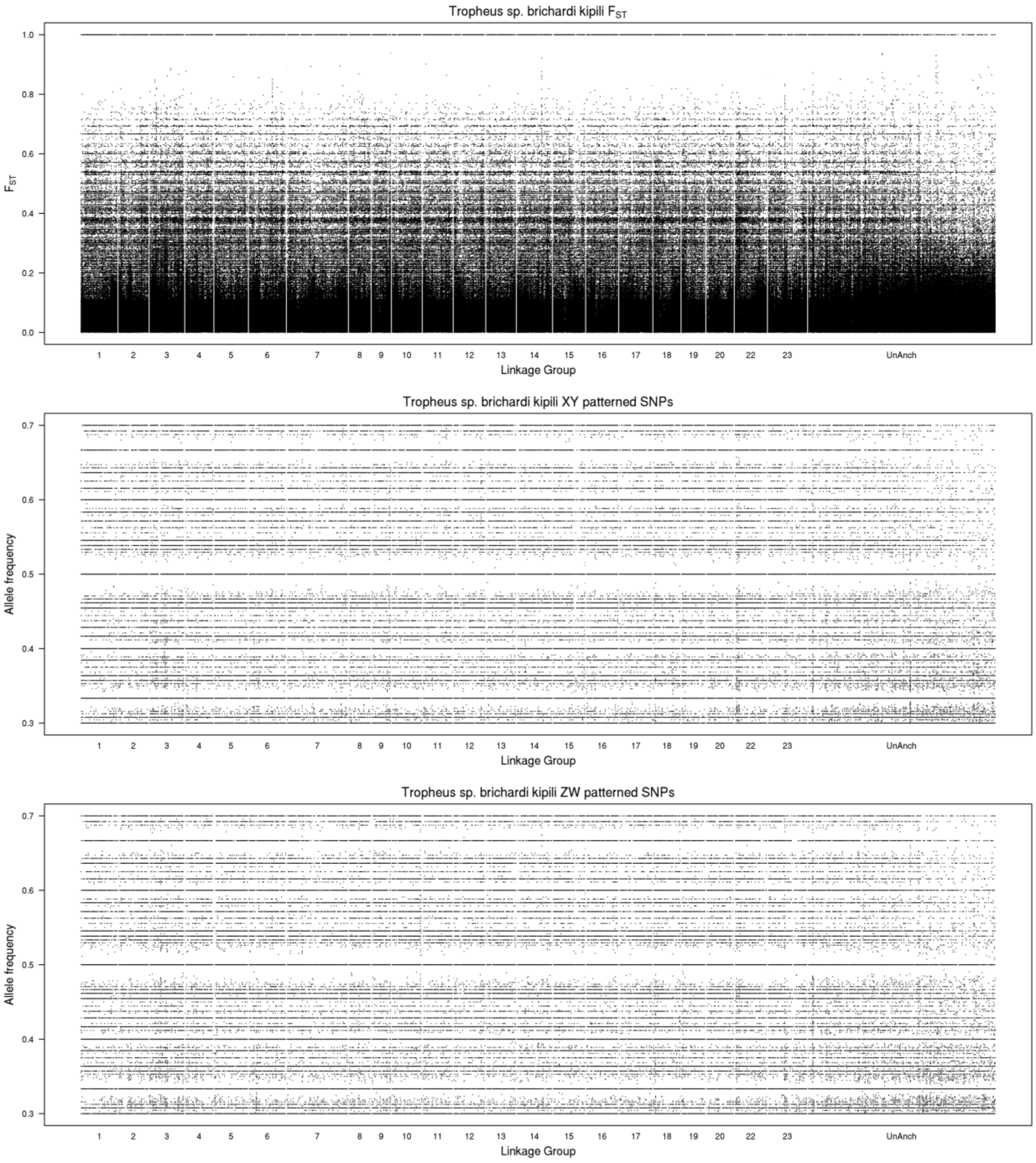
Tropheus sp. brichardi kipili F_{ST}
F_{ST}
1.0
0.8
0.6
0.4
0.2
0.0
1 2 3 4 5 6 7 8 9 10 11 12 13 14 15 16 17 18 19 20 22 23 UnAnch
Linkage Group
Tropheus sp. brichardi kipili XY patterned SNPs
Allele frequency
0.7
0.6
0.5
0.4
0.3
1 2 3 4 5 6 7 8 9 10 11 12 13 14 15 16 17 18 19 20 22 23 UnAnch
Linkage Group
Tropheus sp. brichardi kipili ZW patterned SNPs
Allele frequency
0.7
0.6
0.5
0.4
0.3
1 2 3 4 5 6 7 8 9 10 11 12 13 14 15 16 17 18 19 20 22 23 UnAnch
Linkage Group

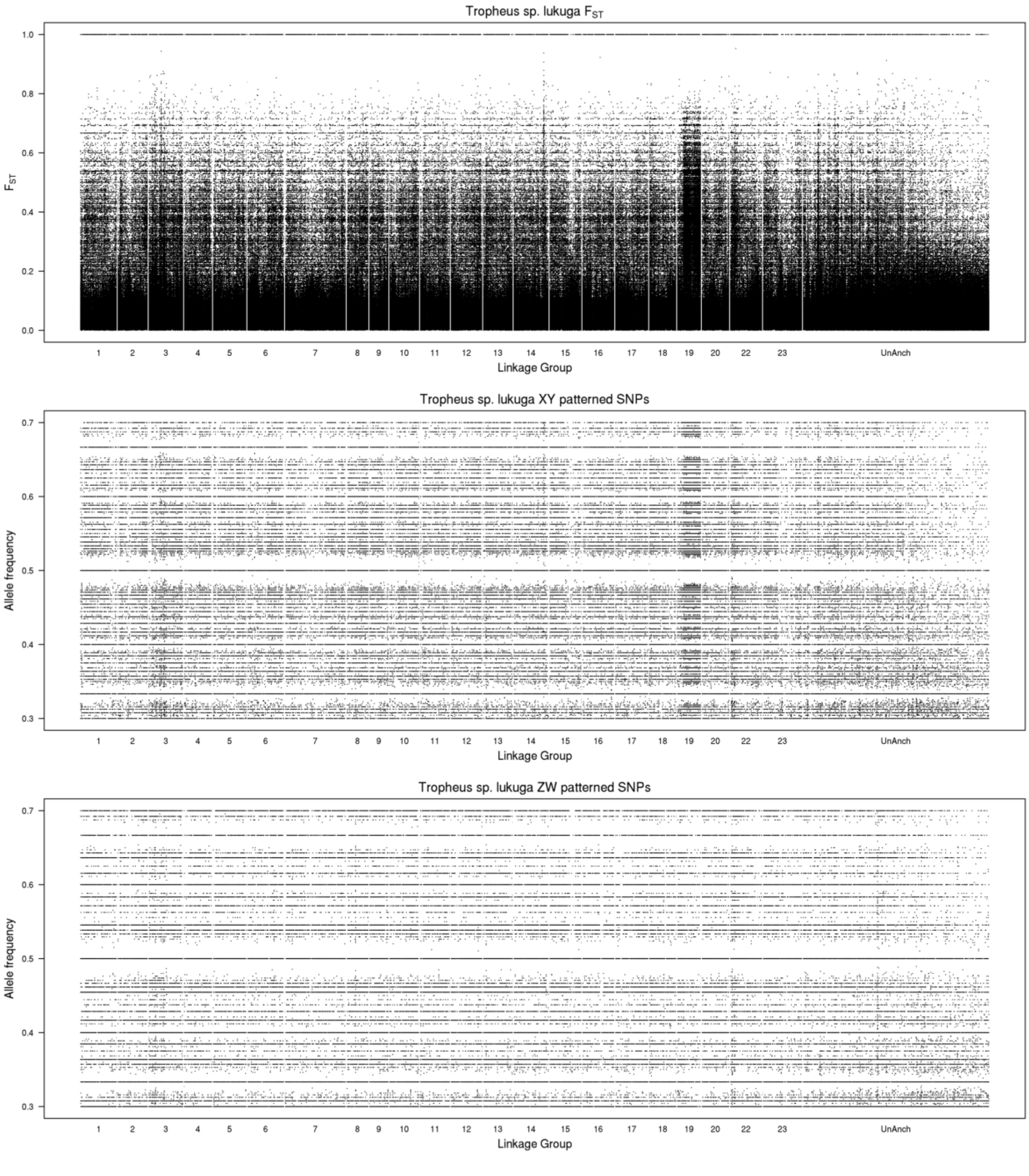

Tropheus sp. lukuga F_{ST}
F_{ST}
1.0
0.8
0.6
0.4
0.2
0.0
1 2 3 4 5 6 7 8 9 10 11 12 13 14 15 16 17 18 19 20 22 23 UnAnch
Linkage Group
Tropheus sp. lukuga XY patterned SNPs
Allele frequency
0.7
0.6
0.5
0.4
0.3
1 2 3 4 5 6 7 8 9 10 11 12 13 14 15 16 17 18 19 20 22 23 UnAnch
Linkage Group
Tropheus sp. lukuga ZW patterned SNPs
Allele frequency
0.7
0.6
0.5
0.4
0.3
1 2 3 4 5 6 7 8 9 10 11 12 13 14 15 16 17 18 19 20 22 23 UnAnch
Linkage Group

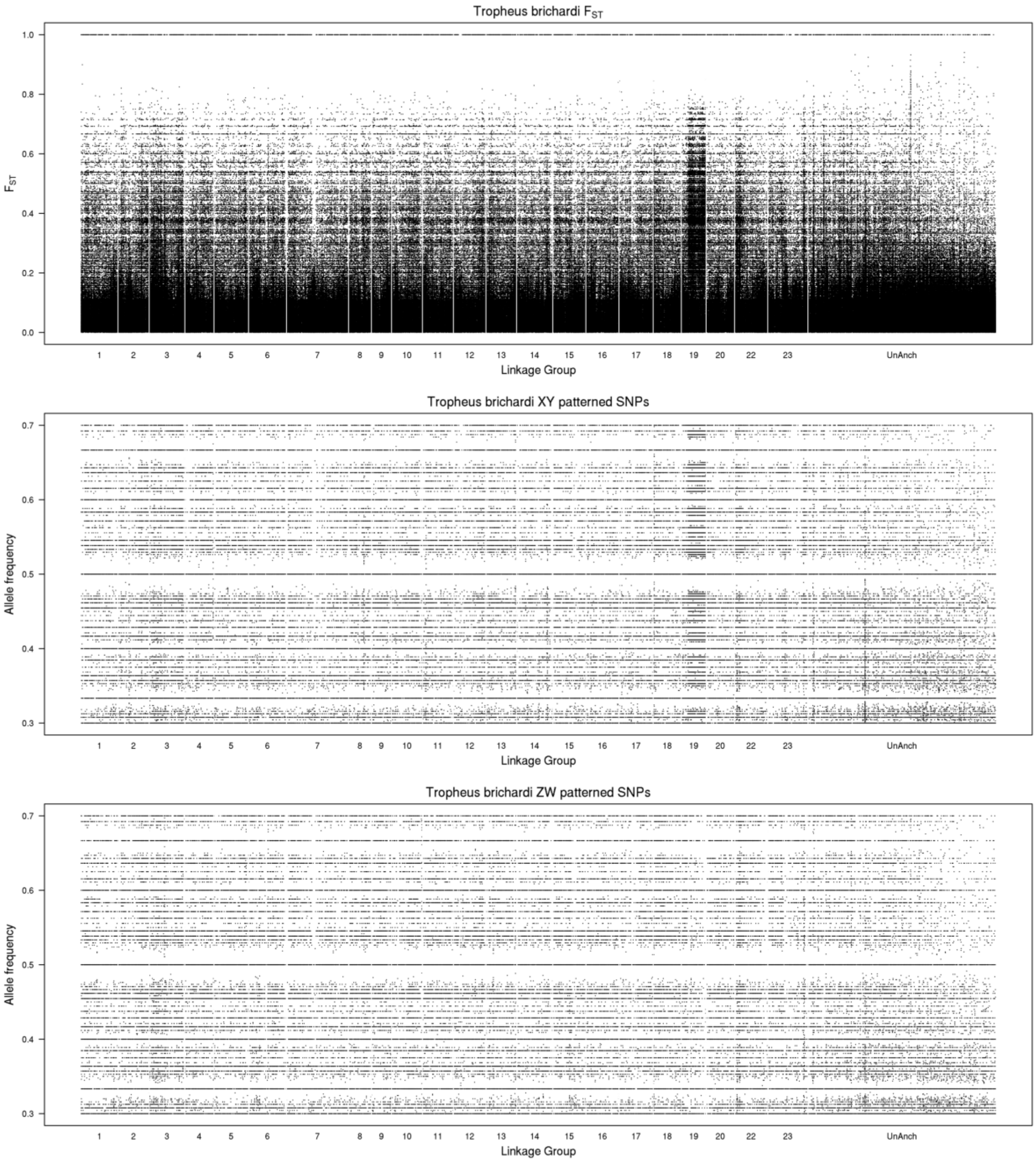
Tropheus brichardi F_{ST}
F_{ST}
1.0
0.8
0.6
0.4
0.2
0.0
1 2 3 4 5 6 7 8 9 10 11 12 13 14 15 16 17 18 19 20 22 23 UnAnch
Linkage Group
Tropheus brichardi XY patterned SNPs
Allele frequency
0.7
0.6
0.5
0.4
0.3
1 2 3 4 5 6 7 8 9 10 11 12 13 14 15 16 17 18 19 20 22 23 UnAnch
Linkage Group
Tropheus brichardi ZW patterned SNPs
Allele frequency
0.7
0.6
0.5
0.4
0.3
1 2 3 4 5 6 7 8 9 10 11 12 13 14 15 16 17 18 19 20 22 23 UnAnch
Linkage Group

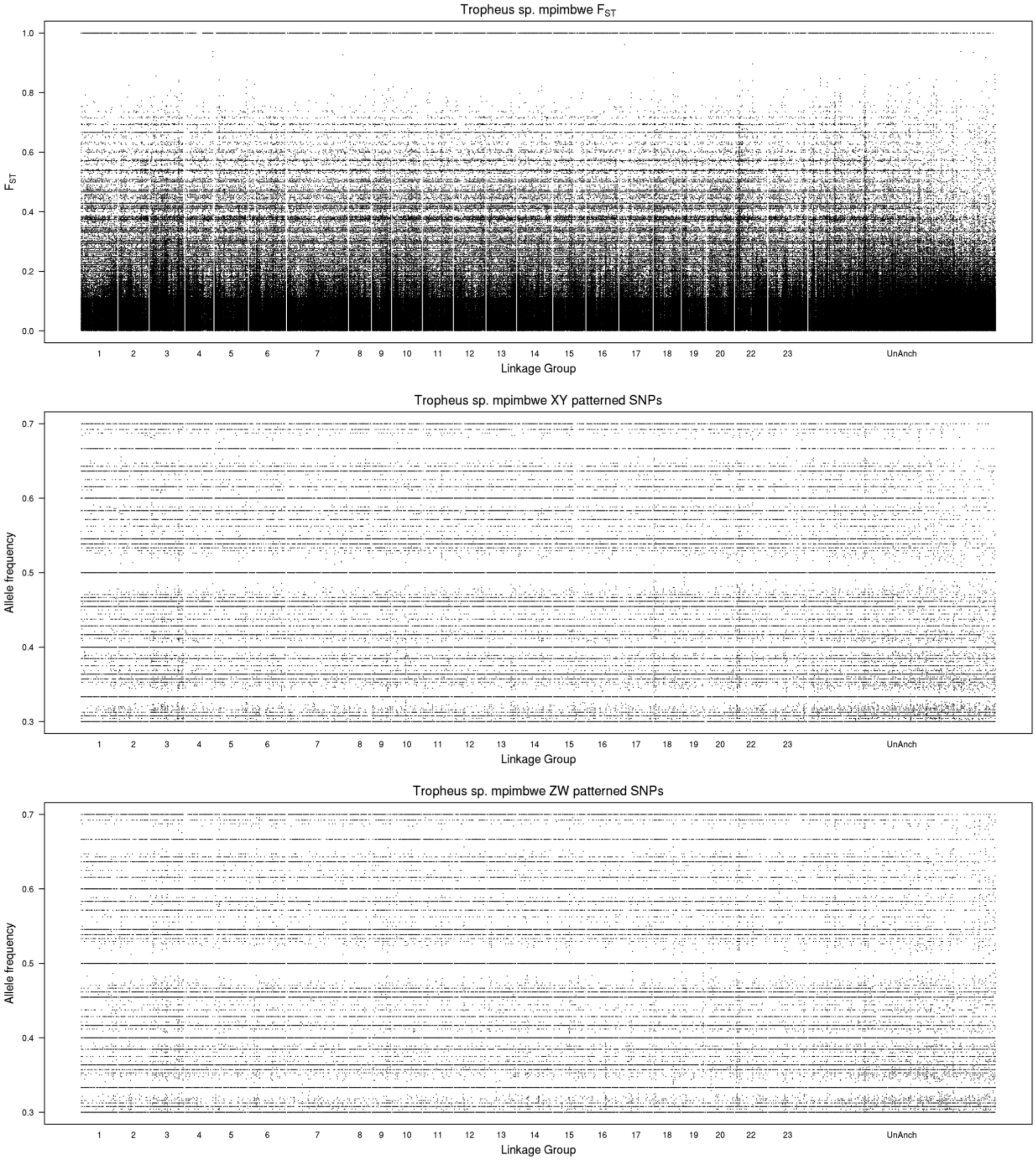

Tropheus sp. mpimbwe F_{ST}
F_{ST}
1.0
0.8
0.6
0.4
0.2
0.0
1 2 3 4 5 6 7 8 9 10 11 12 13 14 15 16 17 18 19 20 22 23 UnAnch
Linkage Group
Tropheus sp. mpimbwe XY patterned SNPs
Allele frequency
0.7
0.6
0.5
0.4
0.3
1 2 3 4 5 6 7 8 9 10 11 12 13 14 15 16 17 18 19 20 22 23 UnAnch
Linkage Group
Tropheus sp. mpimbwe ZW patterned SNPs
Allele frequency
0.7
0.6
0.5
0.4
0.3
1 2 3 4 5 6 7 8 9 10 11 12 13 14 15 16 17 18 19 20 22 23 UnAnch
Linkage Group

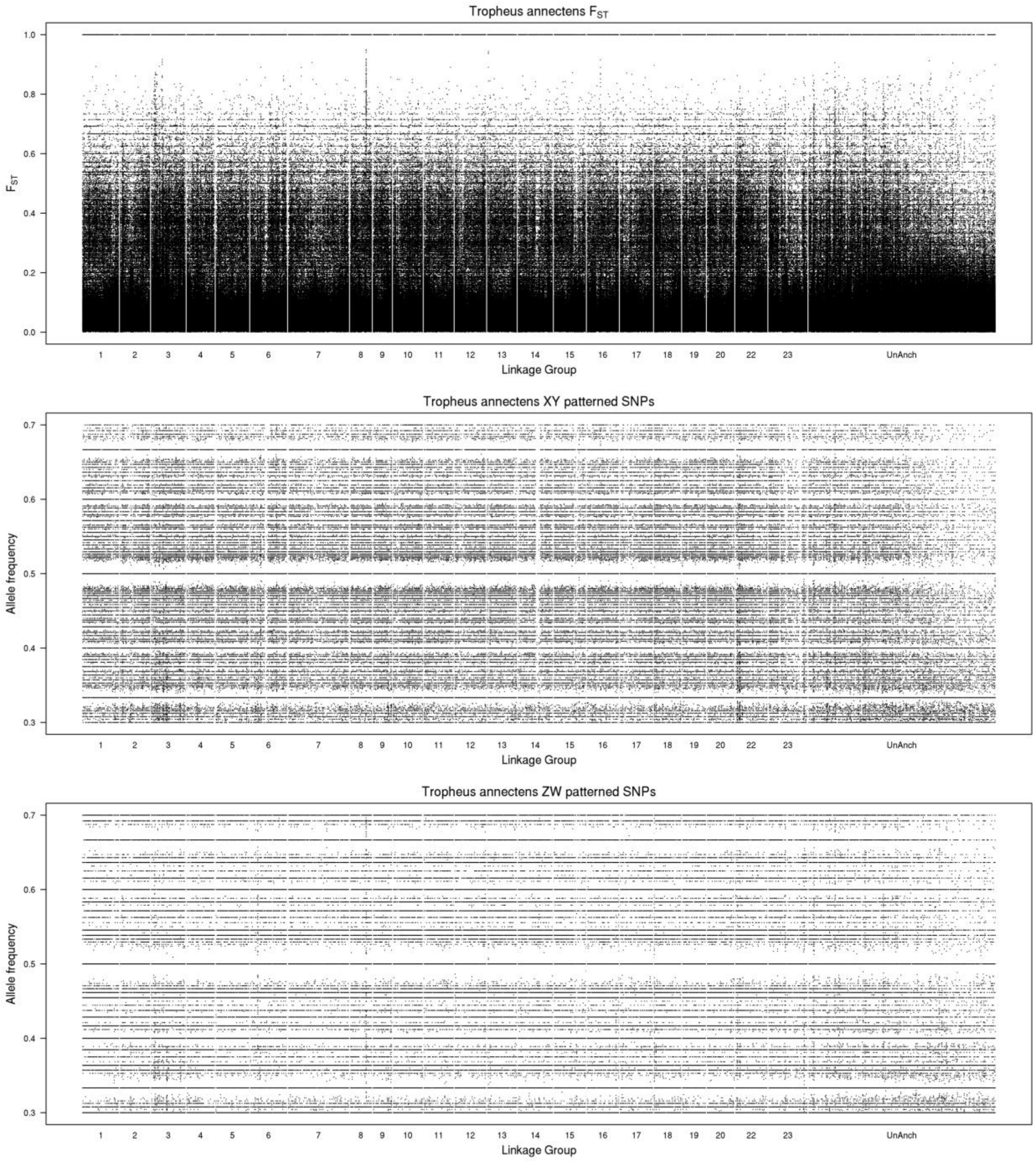
Tropheus annectens F_{ST}
F_{ST}
1.0
0.8
0.6
0.4
0.2
0.0
1 2 3 4 5 6 7 8 9 10 11 12 13 14 15 16 17 18 19 20 22 23 UnAnch
Linkage Group
Tropheus annectens XY patterned SNPs
Allele frequency
0.7
0.6
0.5
0.4
0.3
1 2 3 4 5 6 7 8 9 10 11 12 13 14 15 16 17 18 19 20 22 23 UnAnch
Linkage Group
Tropheus annectens ZW patterned SNPs
Allele frequency
0.7
0.6
0.5
0.4
0.3
1 2 3 4 5 6 7 8 9 10 11 12 13 14 15 16 17 18 19 20 22 23 UnAnch
Linkage Group

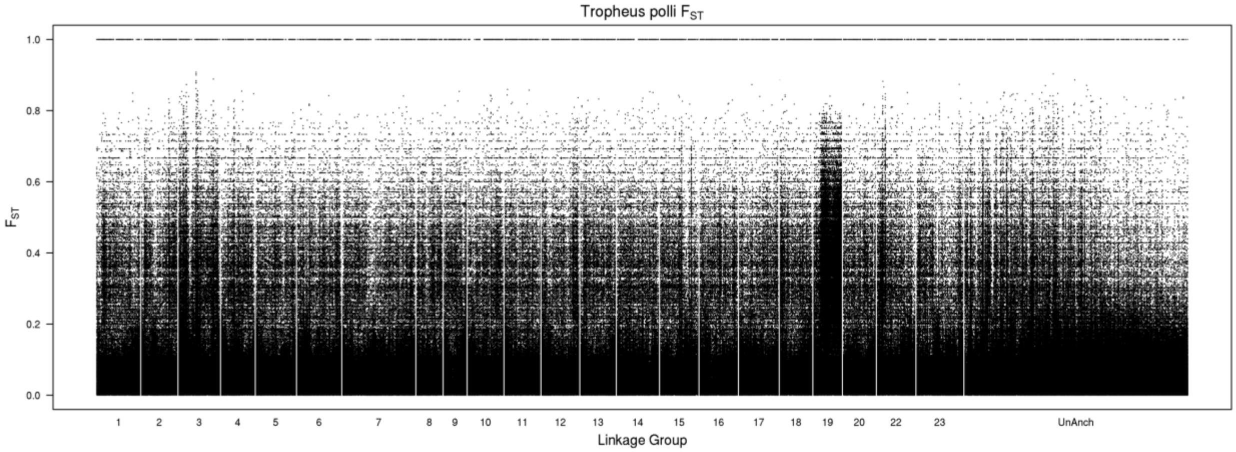
Tropheus polli F_{ST}
F_{ST}
1.0
0.8
0.6
0.4
0.2
0.0
1 2 3 4 5 6 7 8 9 10 11 12 13 14 15 16 17 18 19 20 22 23 UnAnch
Linkage Group

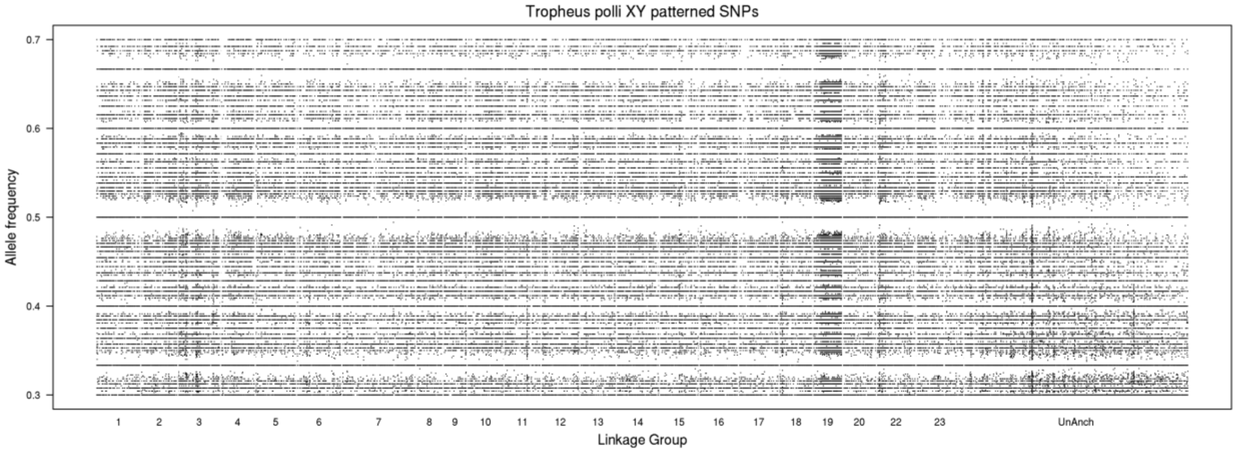
Tropheus polli XY patterned SNPs
Allele frequency
0.7
0.6
0.5
0.4
0.3
1 2 3 4 5 6 7 8 9 10 11 12 13 14 15 16 17 18 19 20 22 23 UnAnch
Linkage Group

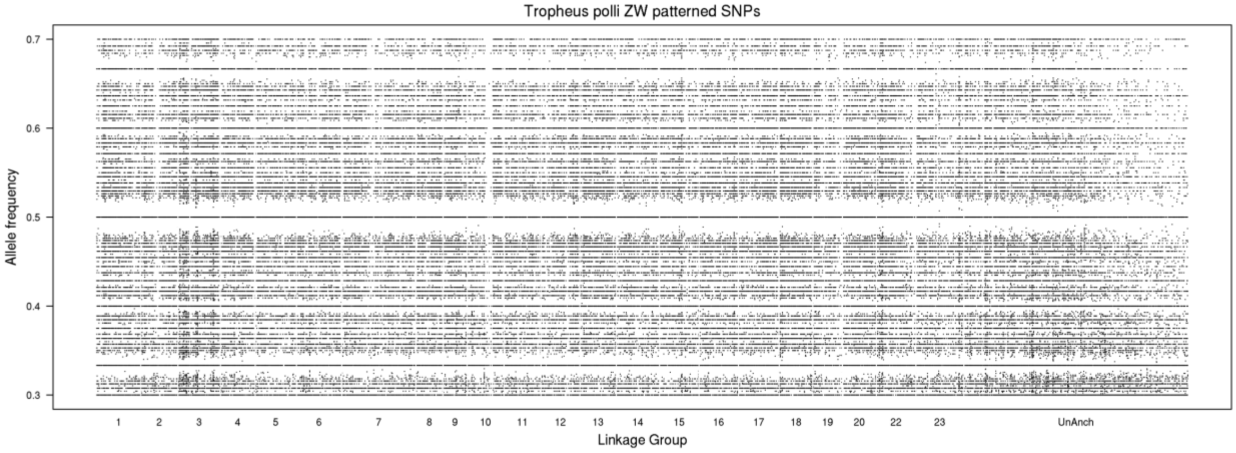
Tropheus polli ZW patterned SNPs
Allele frequency
0.7
0.6
0.5
0.4
0.3
1 2 3 4 5 6 7 8 9 10 11 12 13 14 15 16 17 18 19 20 22 23 UnAnch
Linkage Group

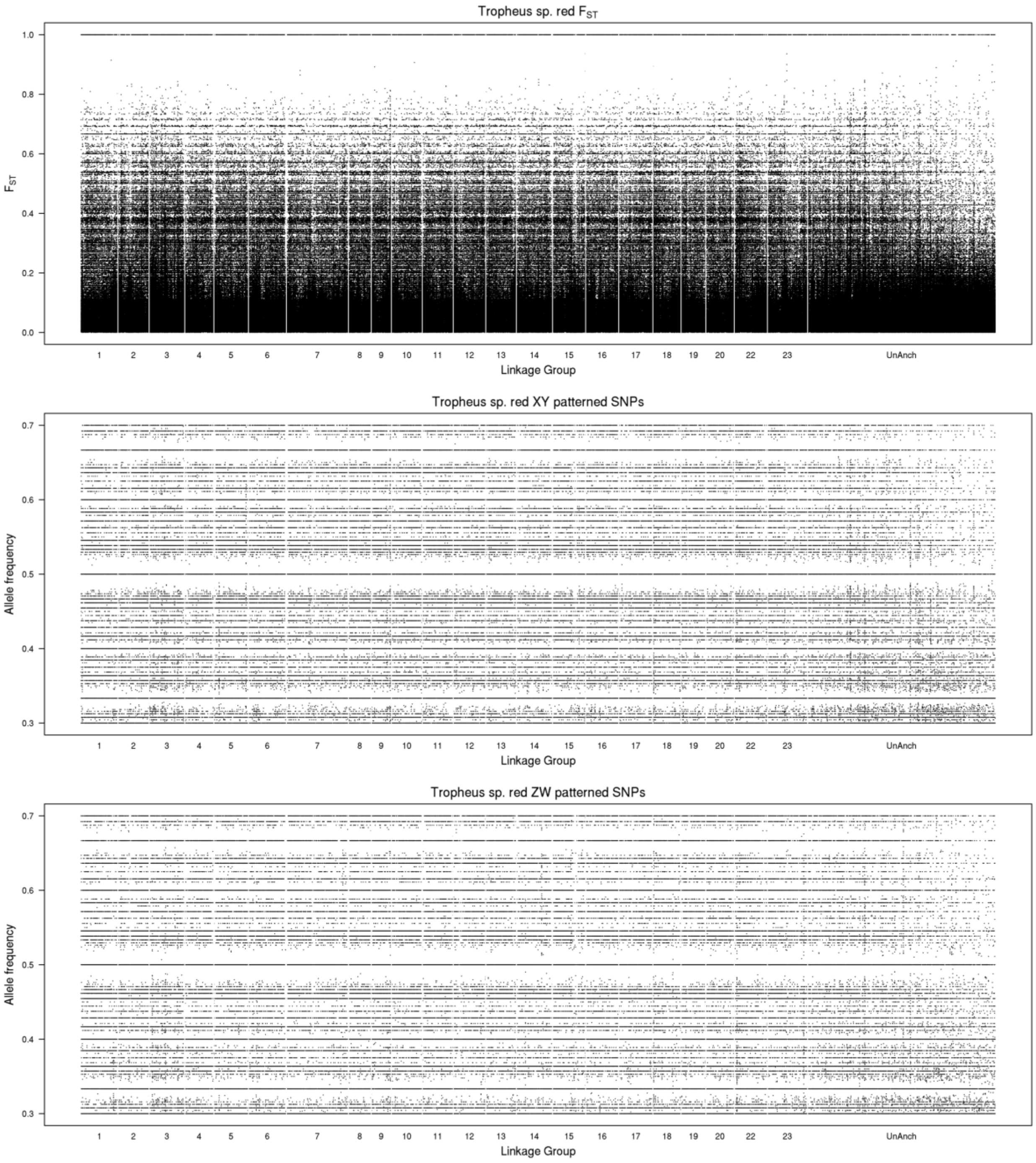

Tropheus sp. red F_{ST}
F_{ST}
1.0
0.8
0.6
0.4
0.2
0.0
1 2 3 4 5 6 7 8 9 10 11 12 13 14 15 16 17 18 19 20 22 23 UnAnch
Linkage Group
Tropheus sp. red XY patterned SNPs
Allele frequency
0.7
0.6
0.5
0.4
0.3
1 2 3 4 5 6 7 8 9 10 11 12 13 14 15 16 17 18 19 20 22 23 UnAnch
Linkage Group
Tropheus sp. red ZW patterned SNPs
Allele frequency
0.7
0.6
0.5
0.4
0.3
1 2 3 4 5 6 7 8 9 10 11 12 13 14 15 16 17 18 19 20 22 23 UnAnch
Linkage Group

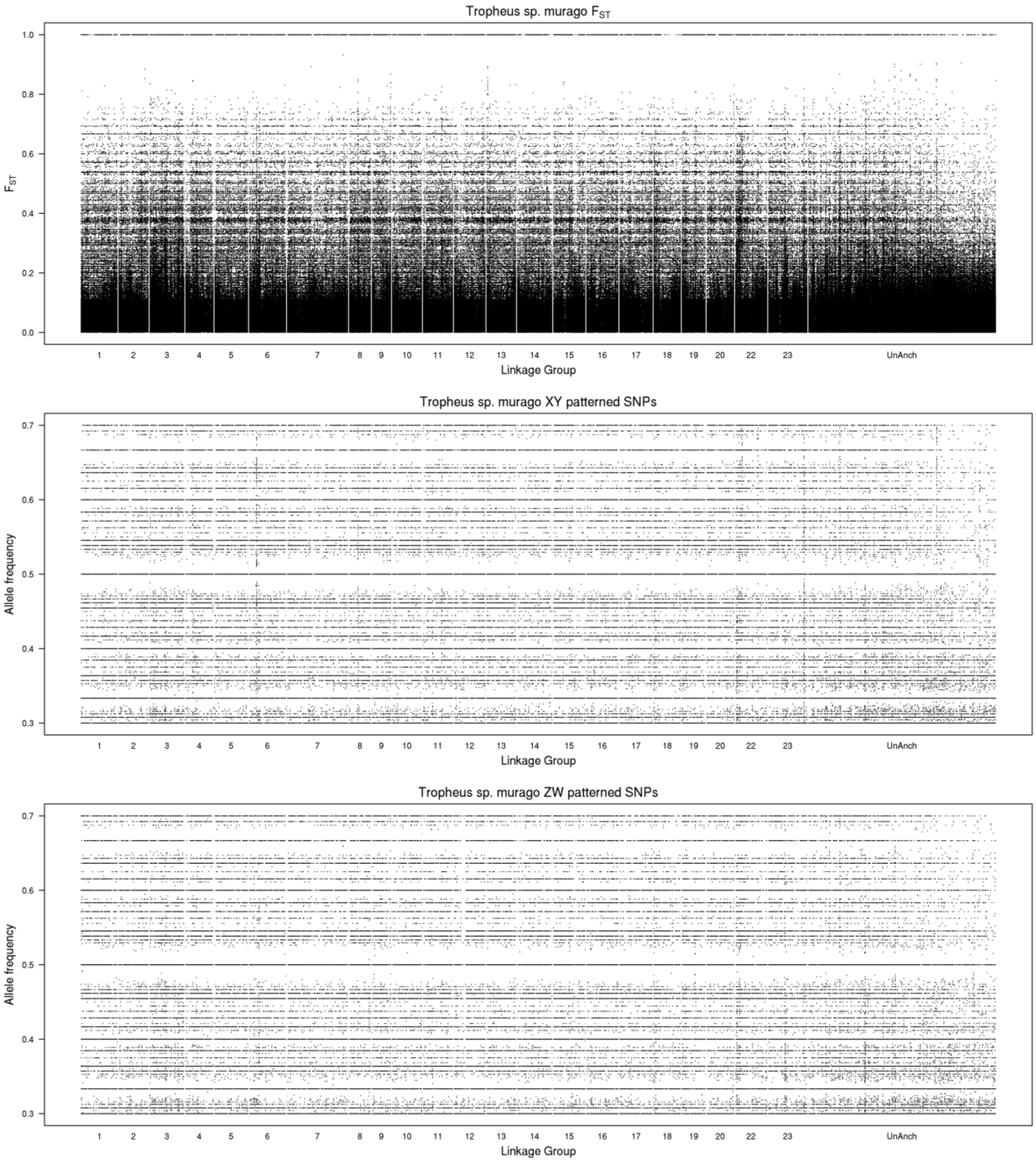
Tropheus sp. murago F_{ST}
F_{ST}
1.0
0.8
0.6
0.4
0.2
0.0
1 2 3 4 5 6 7 8 9 10 11 12 13 14 15 16 17 18 19 20 22 23 UnAnch
Linkage Group
Tropheus sp. murago XY patterned SNPs
Allele frequency
0.7
0.6
0.5
0.4
0.3
1 2 3 4 5 6 7 8 9 10 11 12 13 14 15 16 17 18 19 20 22 23 UnAnch
Linkage Group
Tropheus sp. murago ZW patterned SNPs
Allele frequency
0.7
0.6
0.5
0.4
0.3
1 2 3 4 5 6 7 8 9 10 11 12 13 14 15 16 17 18 19 20 22 23 UnAnch
Linkage Group

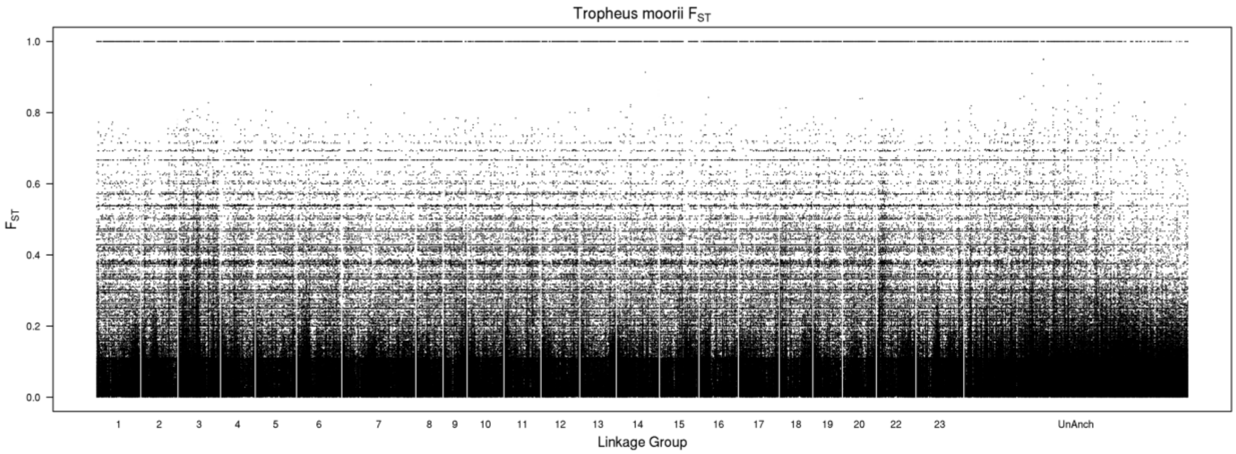
Tropheus moorii F_{ST}
1.0
0.8
0.6
0.4
0.2
0.0
F_{ST}
1 2 3 4 5 6 7 8 9 10 11 12 13 14 15 16 17 18 19 20 22 23 UnAnch
Linkage Group

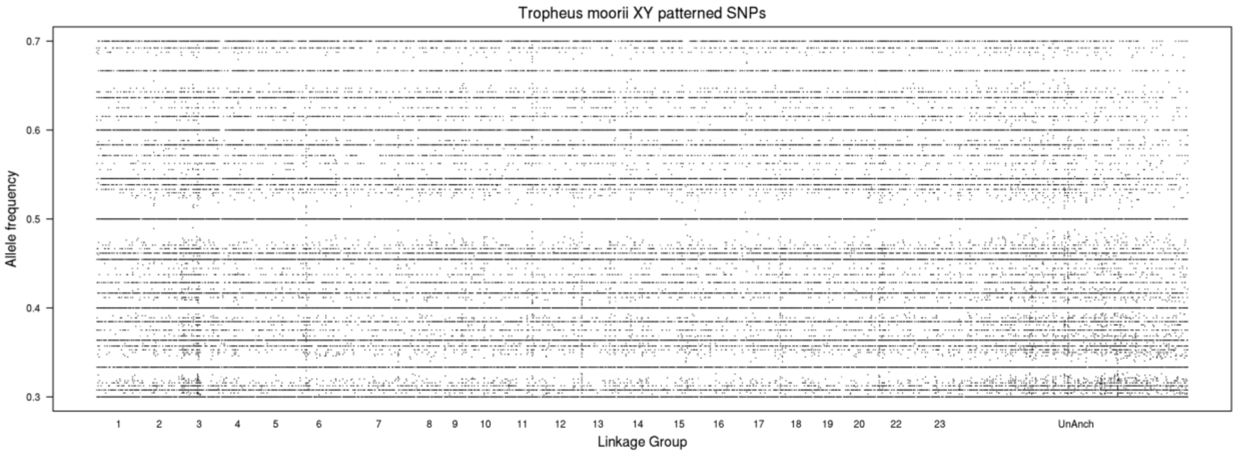
Tropheus moorii XY patterned SNPs
0.7
0.6
0.5
0.4
0.3
Allele frequency
1 2 3 4 5 6 7 8 9 10 11 12 13 14 15 16 17 18 19 20 22 23 UnAnch
Linkage Group

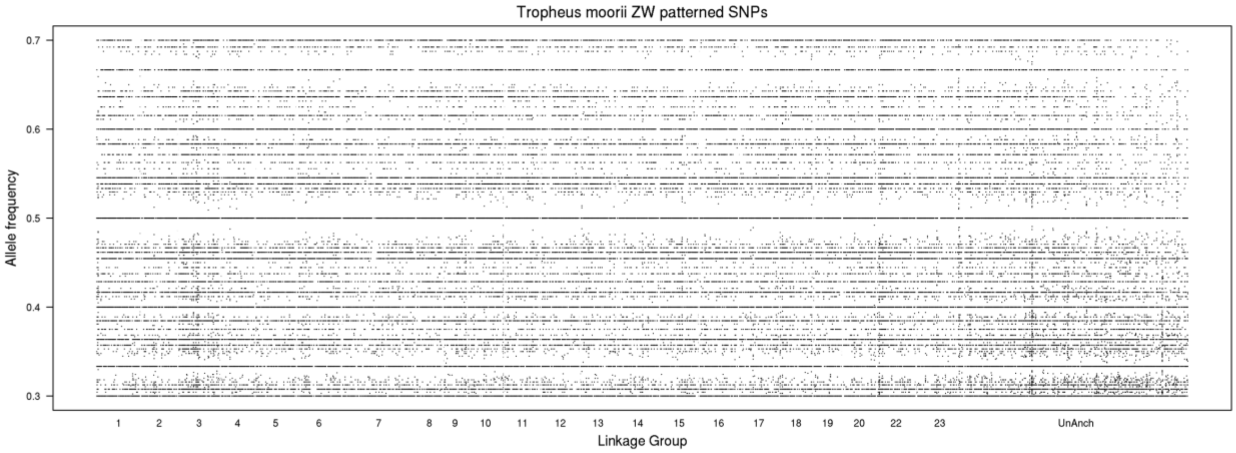
Tropheus moorii ZW patterned SNPs
0.7
0.6
0.5
0.4
0.3
Allele frequency
1 2 3 4 5 6 7 8 9 10 11 12 13 14 15 16 17 18 19 20 22 23 UnAnch
Linkage Group

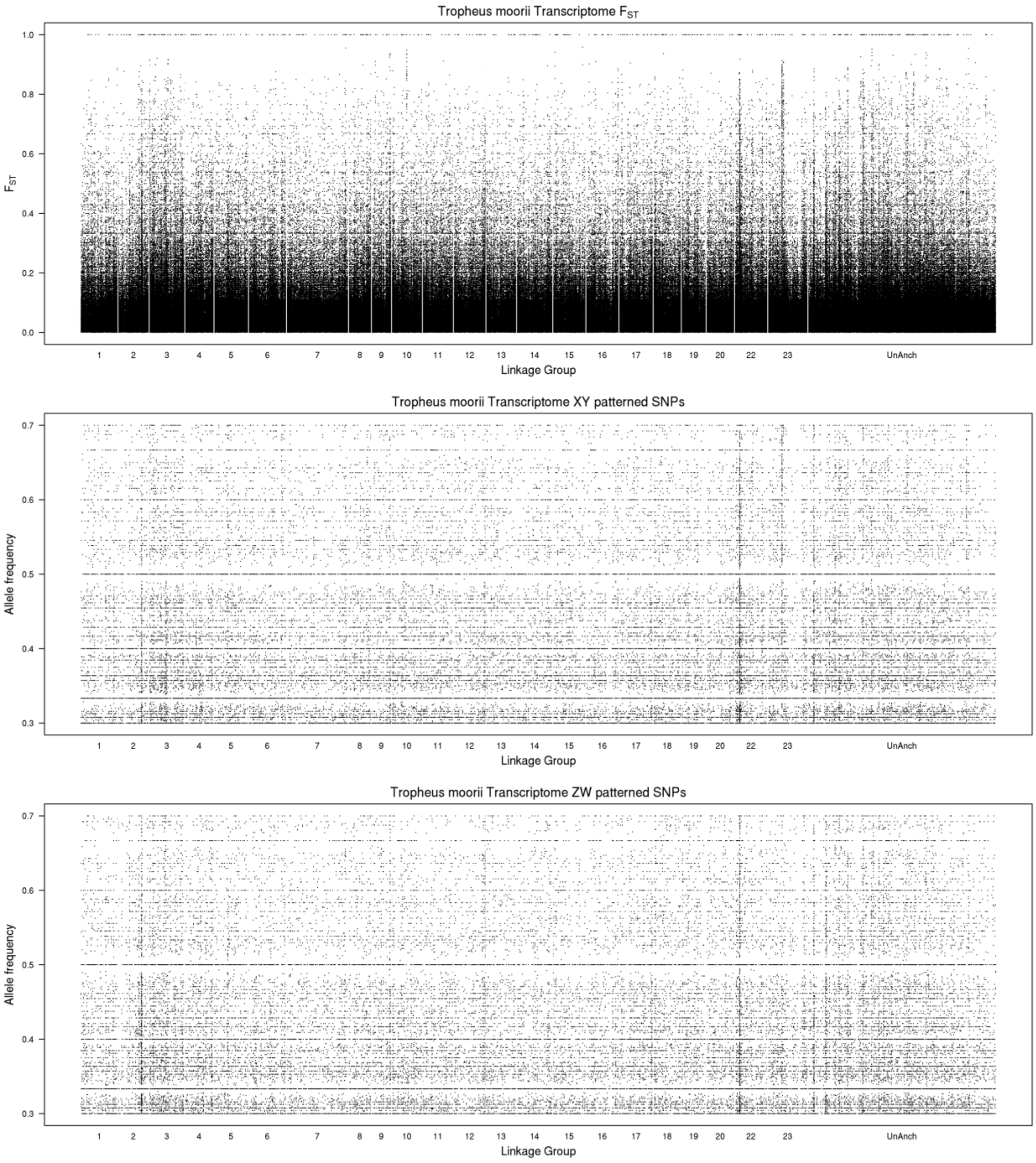
Tropheus moorii Transcriptome F_{ST}
F_{ST}
0.0
0.2
0.4
0.6
0.8
1.0
1 2 3 4 5 6 7 8 9 10 11 12 13 14 15 16 17 18 19 20 22 23 UnAnch
Linkage Group
Tropheus moorii Transcriptome XY patterned SNPs
Allele frequency
0.3
0.4
0.5
0.6
0.7
1 2 3 4 5 6 7 8 9 10 11 12 13 14 15 16 17 18 19 20 22 23 UnAnch
Linkage Group
Tropheus moorii Transcriptome ZW patterned SNPs
Allele frequency
0.3
0.4
0.5
0.6
0.7
1 2 3 4 5 6 7 8 9 10 11 12 13 14 15 16 17 18 19 20 22 23 UnAnch
Linkage Group

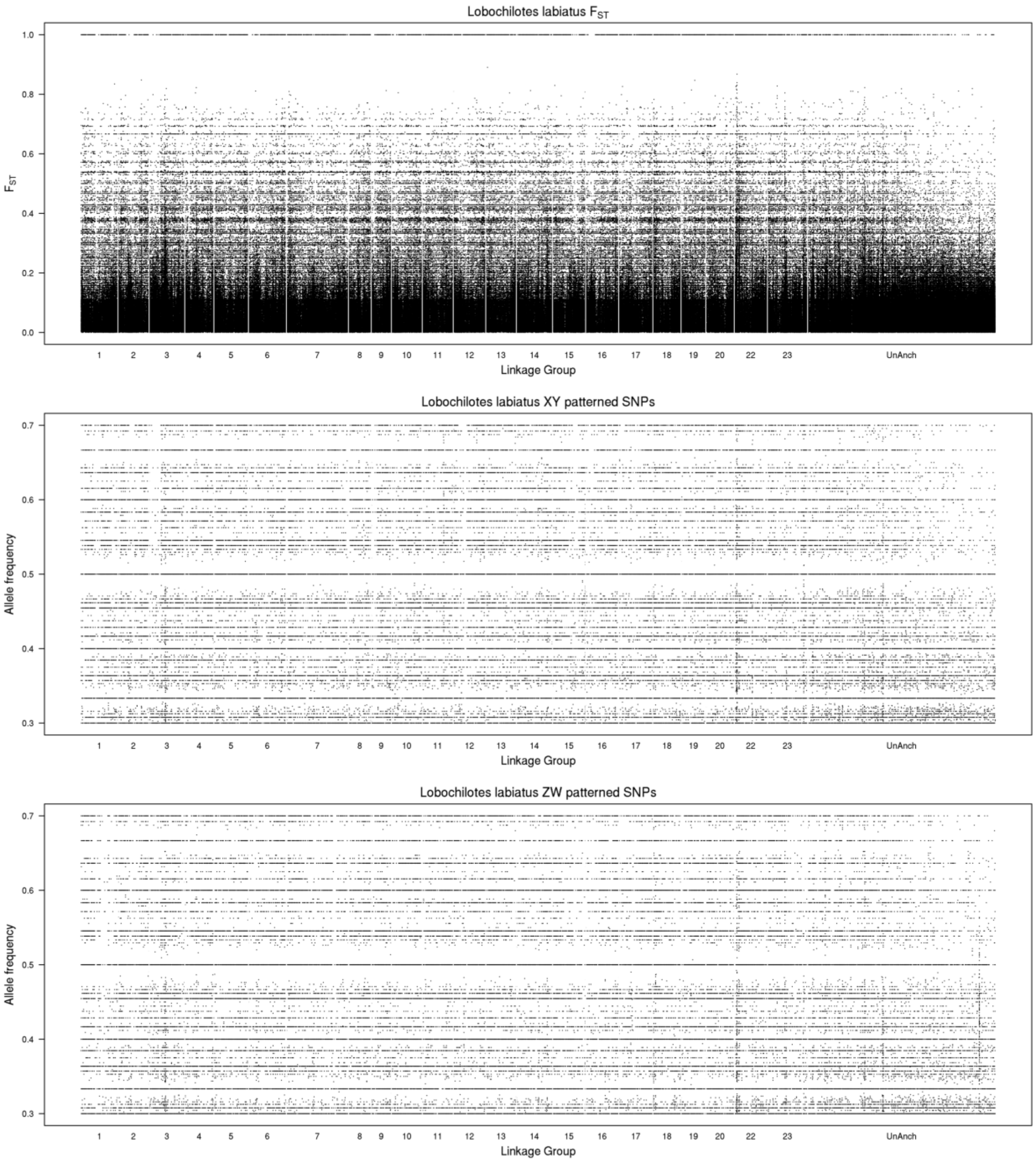
Lobochilotes labiatus F_{ST}
F_{ST}
1.0
0.8
0.6
0.4
0.2
0.0
1 2 3 4 5 6 7 8 9 10 11 12 13 14 15 16 17 18 19 20 22 23 UnAnch
Linkage Group
Lobochilotes labiatus XY patterned SNPs
Allele frequency
0.7
0.6
0.5
0.4
0.3
1 2 3 4 5 6 7 8 9 10 11 12 13 14 15 16 17 18 19 20 22 23 UnAnch
Linkage Group
Lobochilotes labiatus ZW patterned SNPs
Allele frequency
0.7
0.6
0.5
0.4
0.3
1 2 3 4 5 6 7 8 9 10 11 12 13 14 15 16 17 18 19 20 22 23 UnAnch
Linkage Group

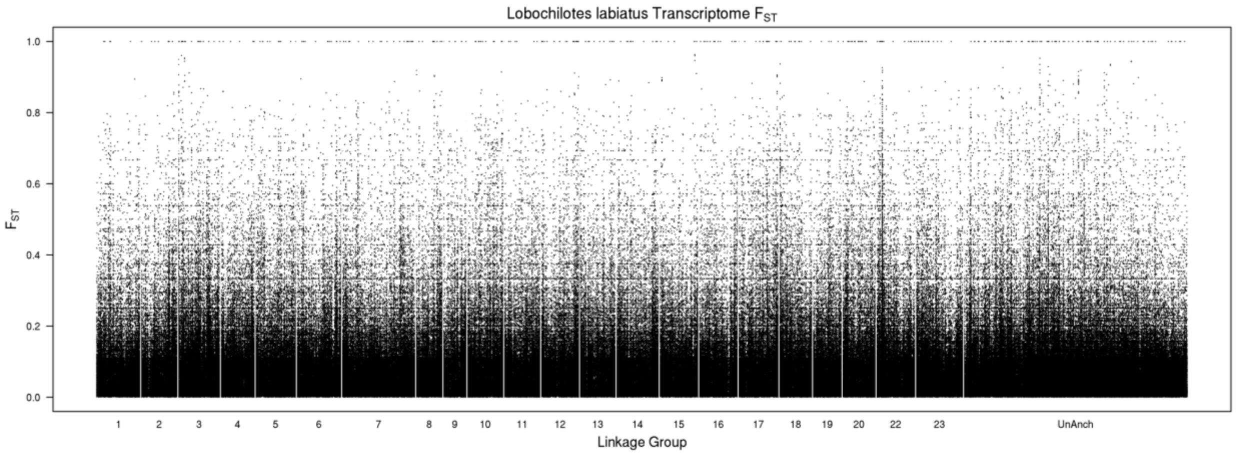
Lobochilotes labiatus Transcriptome F_{ST}
F_{ST}
1.0
0.8
0.6
0.4
0.2
0.0
1 2 3 4 5 6 7 8 9 10 11 12 13 14 15 16 17 18 19 20 22 23 UnAnch
Linkage Group

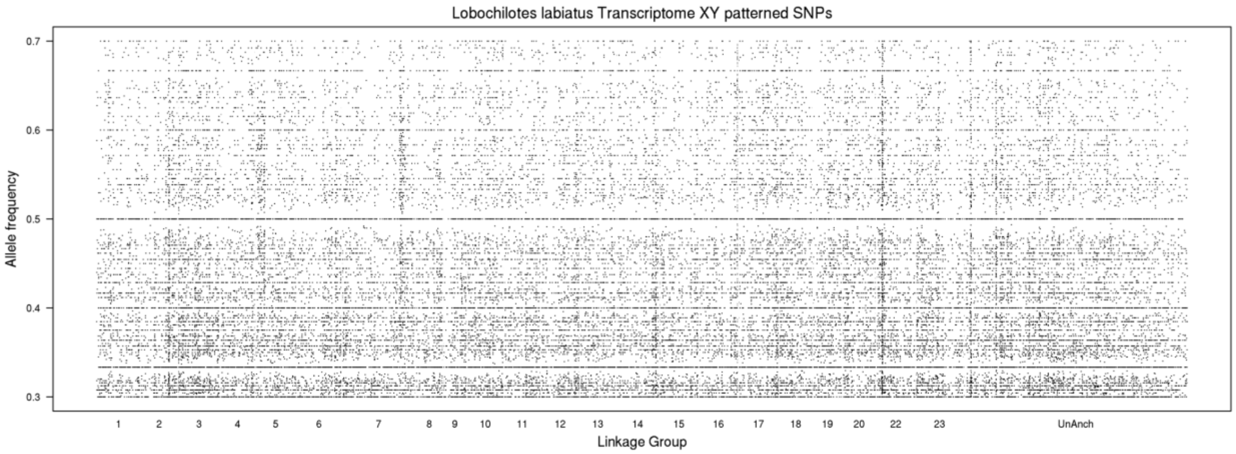
Lobochilotes labiatus Transcriptome XY patterned SNPs
Allele frequency
0.7
0.6
0.5
0.4
0.3
1 2 3 4 5 6 7 8 9 10 11 12 13 14 15 16 17 18 19 20 22 23 UnAnch
Linkage Group

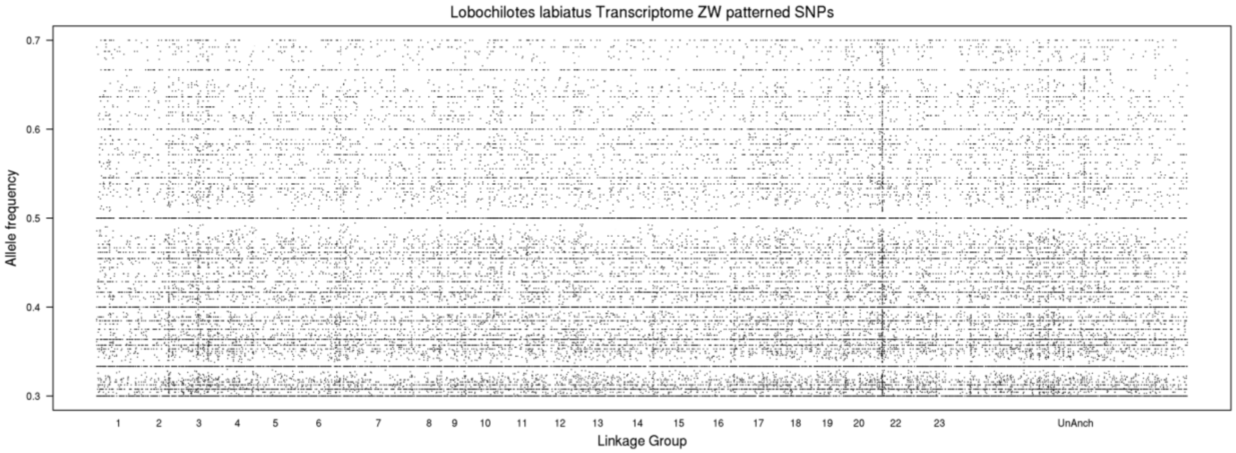
Lobochilotes labiatus Transcriptome ZW patterned SNPs
Allele frequency
0.7
0.6
0.5
0.4
0.3
1 2 3 4 5 6 7 8 9 10 11 12 13 14 15 16 17 18 19 20 22 23 UnAnch
Linkage Group

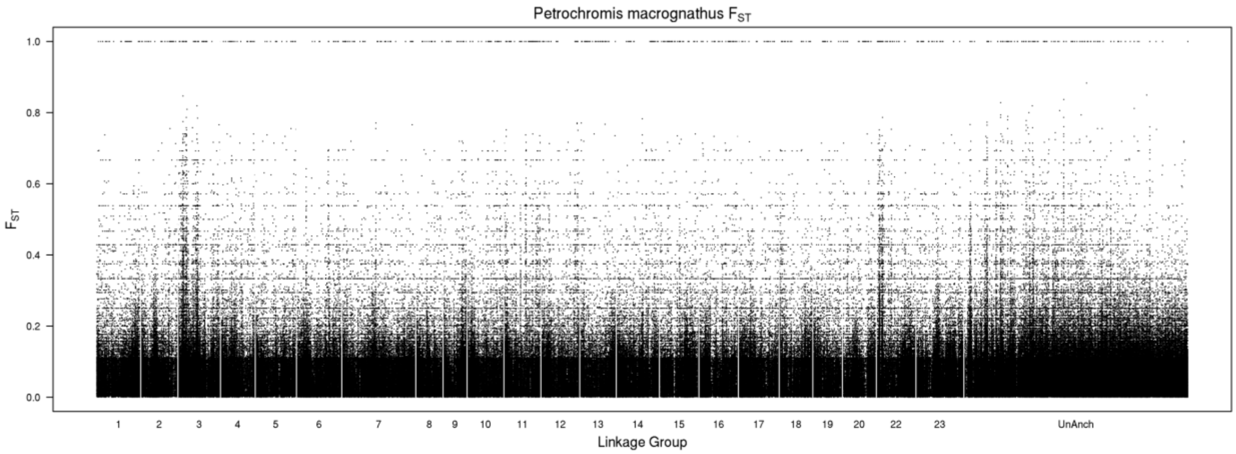
Petrochromis macrognathus F_{ST}
F_{ST}
1.0
0.8
0.6
0.4
0.2
0.0
1 2 3 4 5 6 7 8 9 10 11 12 13 14 15 16 17 18 19 20 22 23 UnAnch
Linkage Group

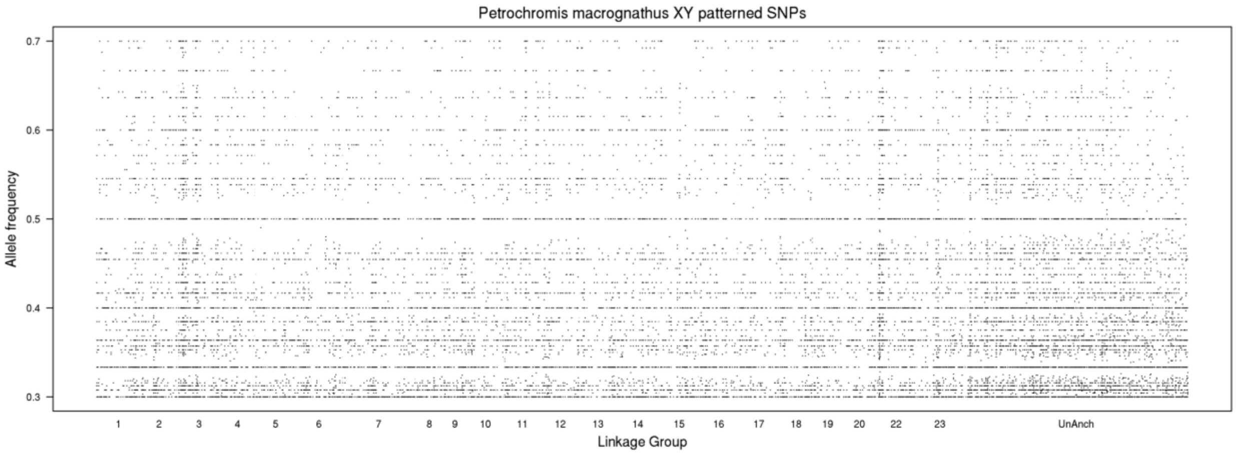
Petrochromis macrognathus XY patterned SNPs
Allele frequency
0.7
0.6
0.5
0.4
0.3
1 2 3 4 5 6 7 8 9 10 11 12 13 14 15 16 17 18 19 20 22 23 UnAnch
Linkage Group

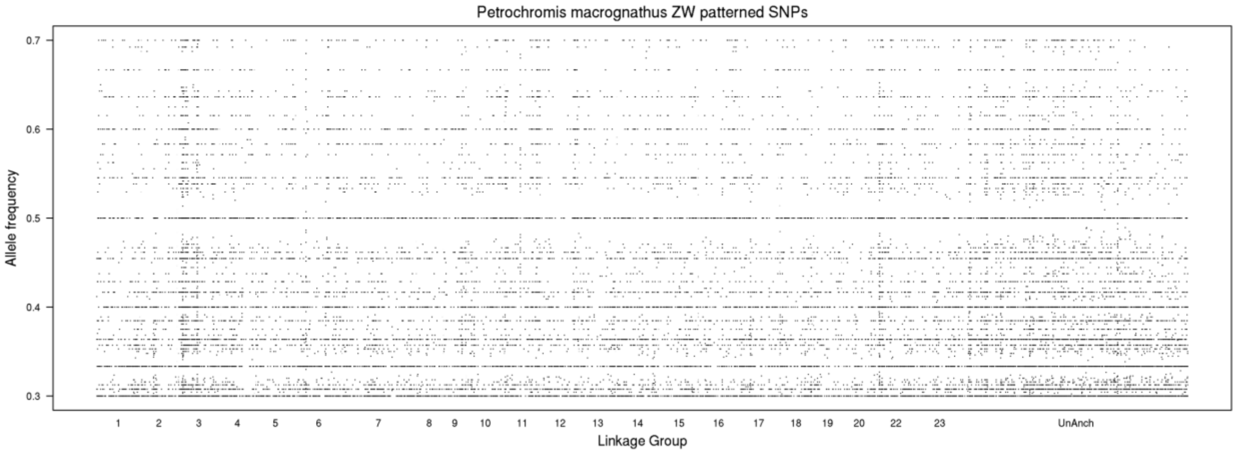
Petrochromis macrognathus ZW patterned SNPs
Allele frequency
0.7
0.6
0.5
0.4
0.3
1 2 3 4 5 6 7 8 9 10 11 12 13 14 15 16 17 18 19 20 22 23 UnAnch
Linkage Group

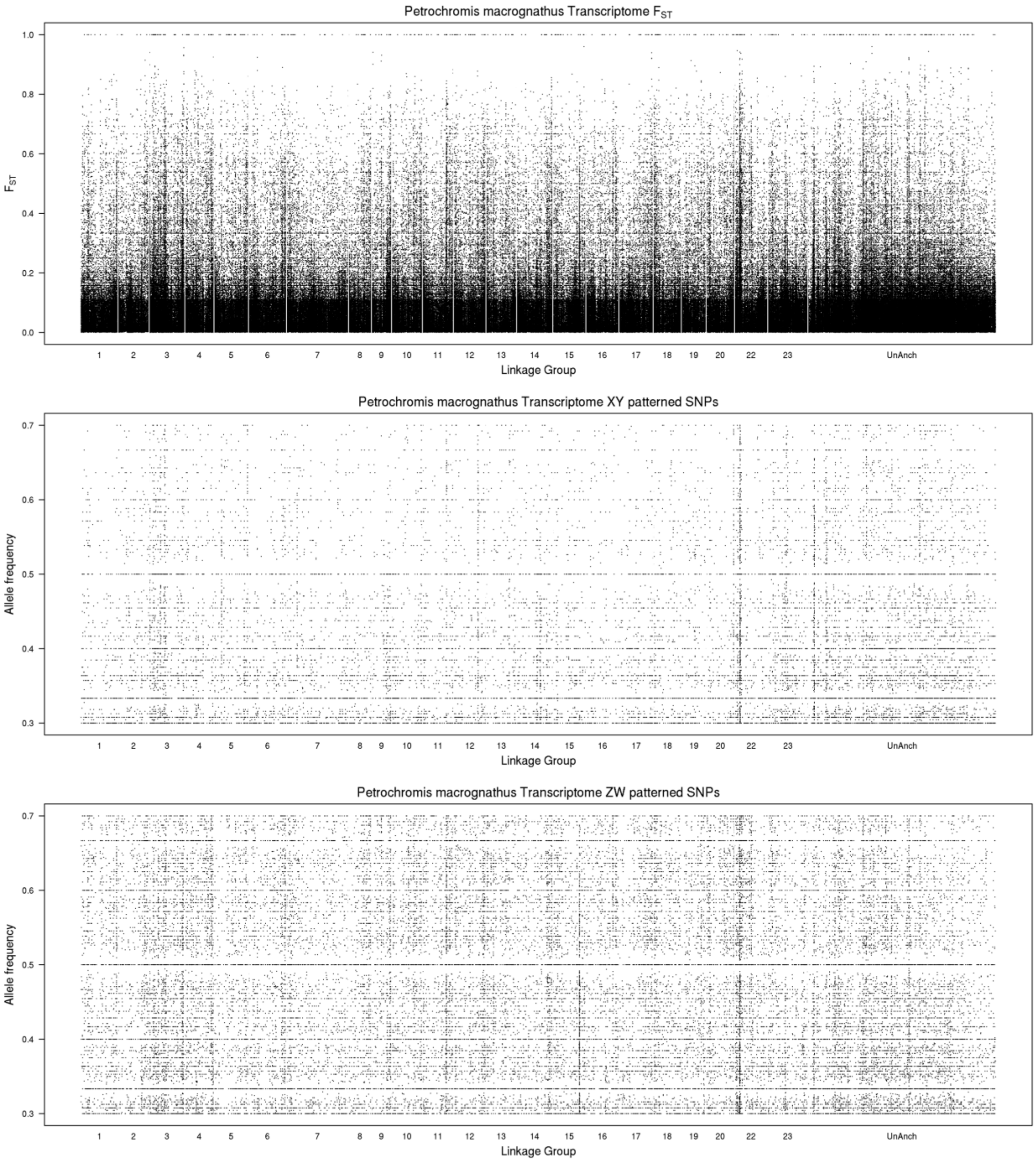

Petrochromis macrognathus Transcriptome F_{ST}
F_{ST}
1.0
0.8
0.6
0.4
0.2
0.0
1 2 3 4 5 6 7 8 9 10 11 12 13 14 15 16 17 18 19 20 22 23 UnAnch
Linkage Group
Petrochromis macrognathus Transcriptome XY patterned SNPs
Allele frequency
0.7
0.6
0.5
0.4
0.3
1 2 3 4 5 6 7 8 9 10 11 12 13 14 15 16 17 18 19 20 22 23 UnAnch
Linkage Group
Petrochromis macrognathus Transcriptome ZW patterned SNPs
Allele frequency
0.7
0.6
0.5
0.4
0.3
1 2 3 4 5 6 7 8 9 10 11 12 13 14 15 16 17 18 19 20 22 23 UnAnch
Linkage Group

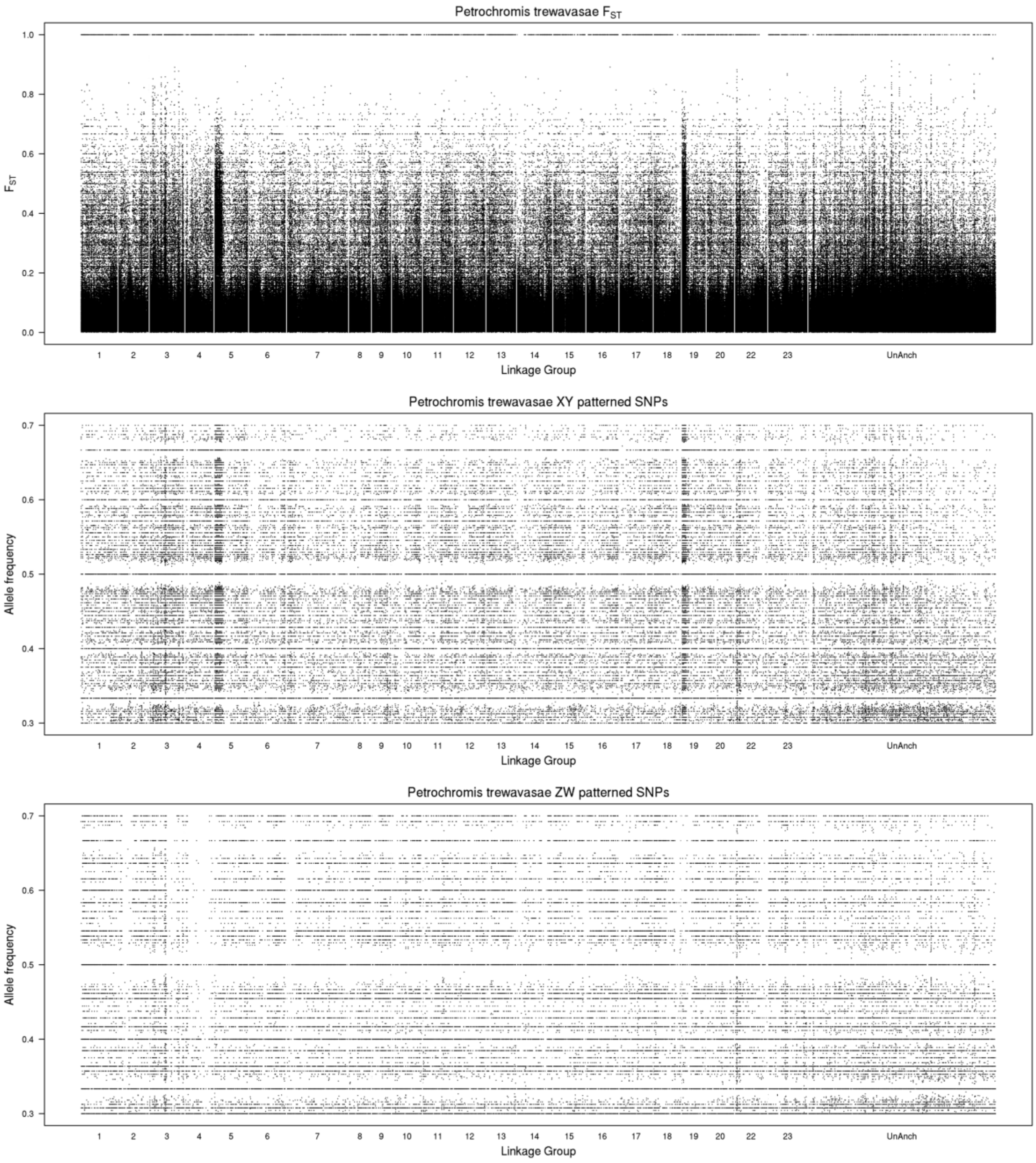

Petrochromis trewavasae F_{ST}
F_{ST}
1.0
0.8
0.6
0.4
0.2
0.0
1 2 3 4 5 6 7 8 9 10 11 12 13 14 15 16 17 18 19 20 22 23 UnAnch
Linkage Group
Petrochromis trewavasae XY patterned SNPs
Allele frequency
0.7
0.6
0.5
0.4
0.3
1 2 3 4 5 6 7 8 9 10 11 12 13 14 15 16 17 18 19 20 22 23 UnAnch
Linkage Group
Petrochromis trewavasae ZW patterned SNPs
Allele frequency
0.7
0.6
0.5
0.4
0.3
1 2 3 4 5 6 7 8 9 10 11 12 13 14 15 16 17 18 19 20 22 23 UnAnch
Linkage Group

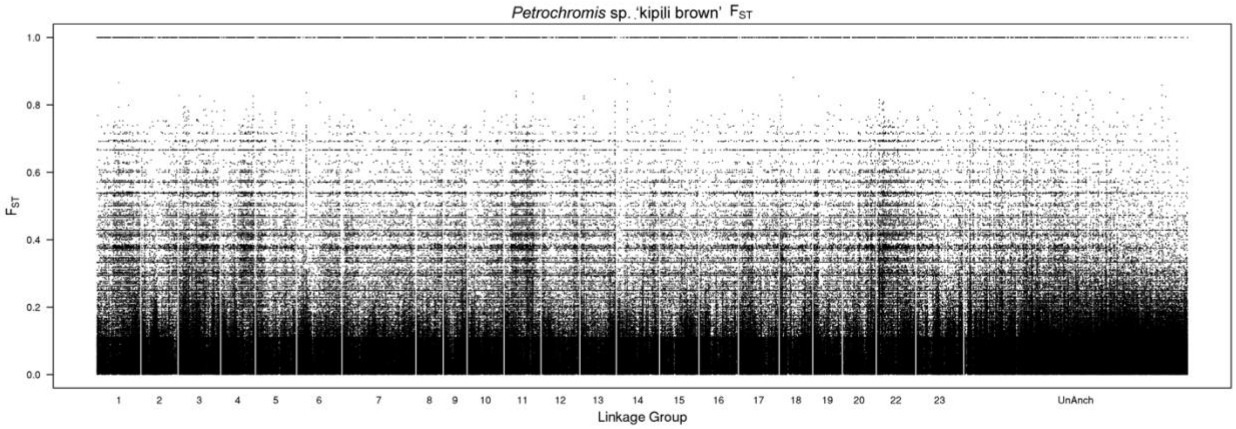
Petrochromis sp. 'kipili brown' F_{ST}
F_{ST}
0.0
0.2
0.4
0.6
0.8
1.0
1 2 3 4 5 6 7 8 9 10 11 12 13 14 15 16 17 18 19 20 22 23 UnAnch
Linkage Group

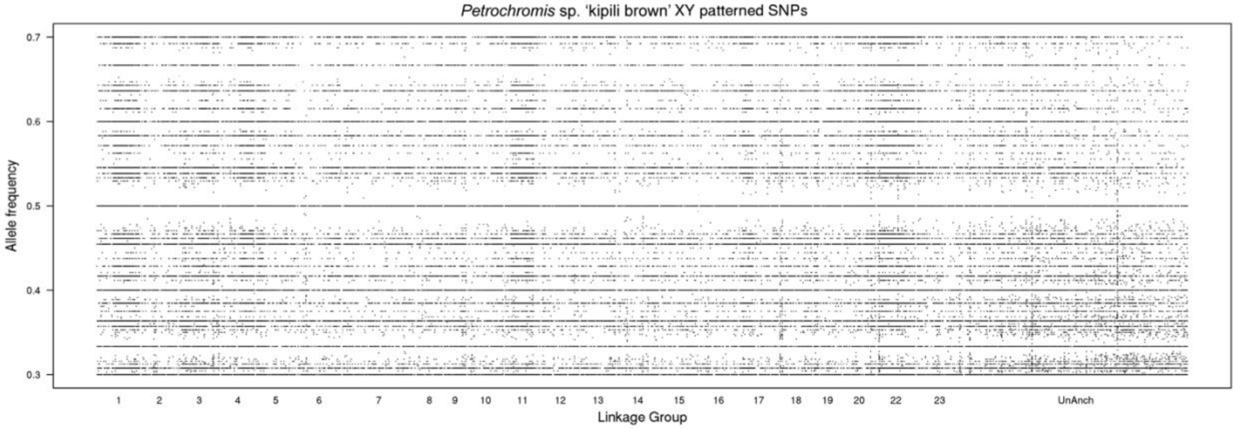
Petrochromis sp. 'kipili brown' XY patterned SNPs
Allele frequency
0.3
0.4
0.5
0.6
0.7
1 2 3 4 5 6 7 8 9 10 11 12 13 14 15 16 17 18 19 20 22 23 UnAnch
Linkage Group

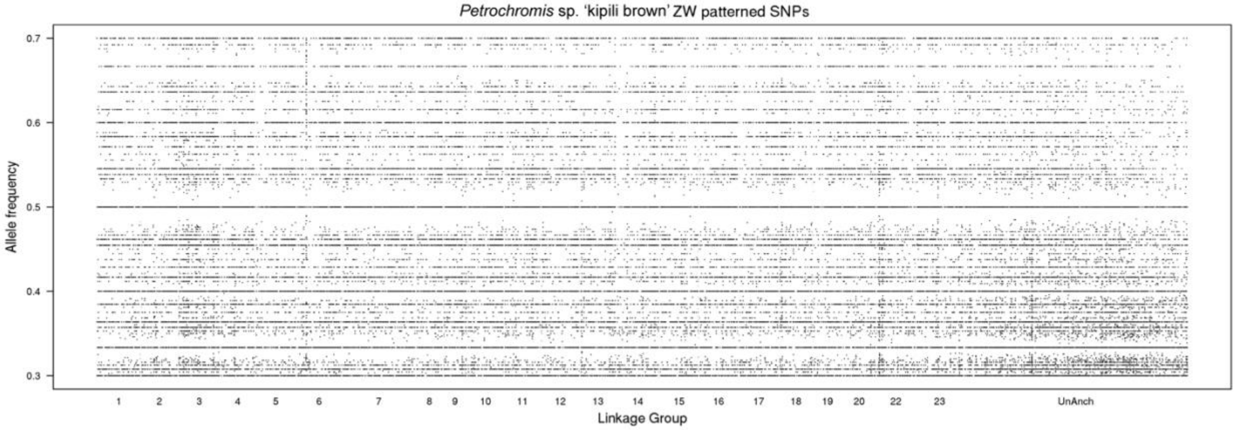
Petrochromis sp. 'kipili brown' ZW patterned SNPs
Allele frequency
0.3
0.4
0.5
0.6
0.7
1 2 3 4 5 6 7 8 9 10 11 12 13 14 15 16 17 18 19 20 22 23 UnAnch
Linkage Group

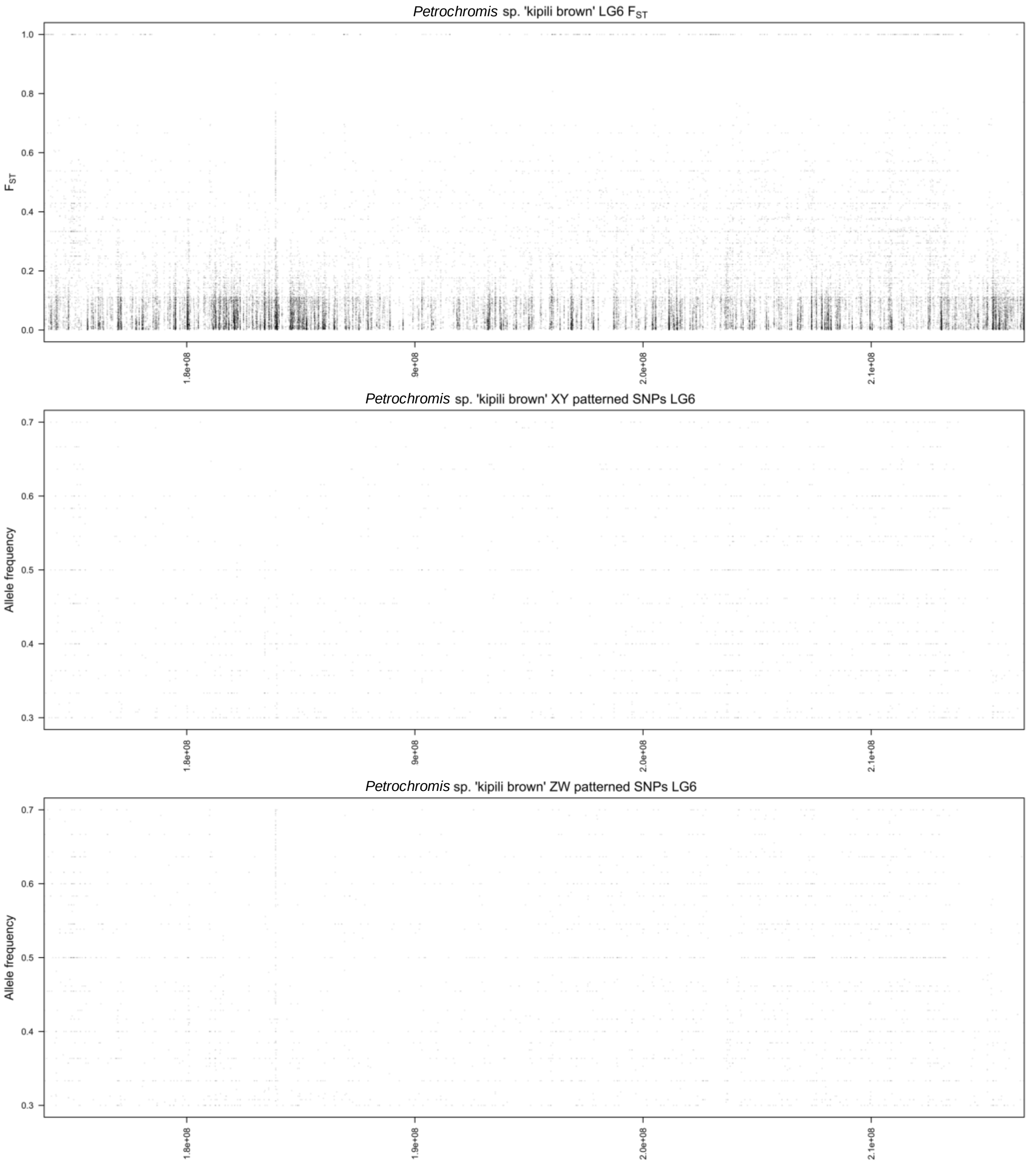

Petrochromis sp. 'kipili brown' LG6 F_{ST}
F_{ST}
1.0
0.8
0.6
0.4
0.2
0.0
1.8e+08
9e+08
2.0e+08
2.1e+08
Petrochromis sp. 'kipili brown' XY patterned SNPs LG6
Allele frequency
0.7
0.6
0.5
0.4
0.3
1.8e+08
9e+08
2.0e+08
2.1e+08
Petrochromis sp. 'kipili brown' ZW patterned SNPs LG6
Allele frequency
0.7
0.6
0.5
0.4
0.3
1.8e+08
1.9e+08
2.0e+08
2.1e+08

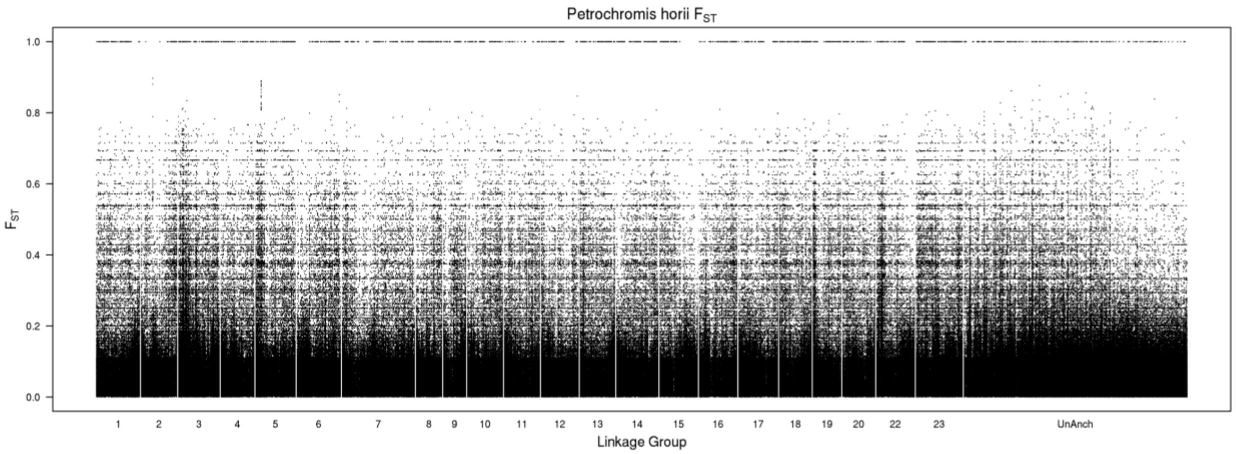

Petrochromis horii F_{ST}
F_{ST}
1.0
0.8
0.6
0.4
0.2
0.0
1 2 3 4 5 6 7 8 9 10 11 12 13 14 15 16 17 18 19 20 22 23 UnAnch
Linkage Group

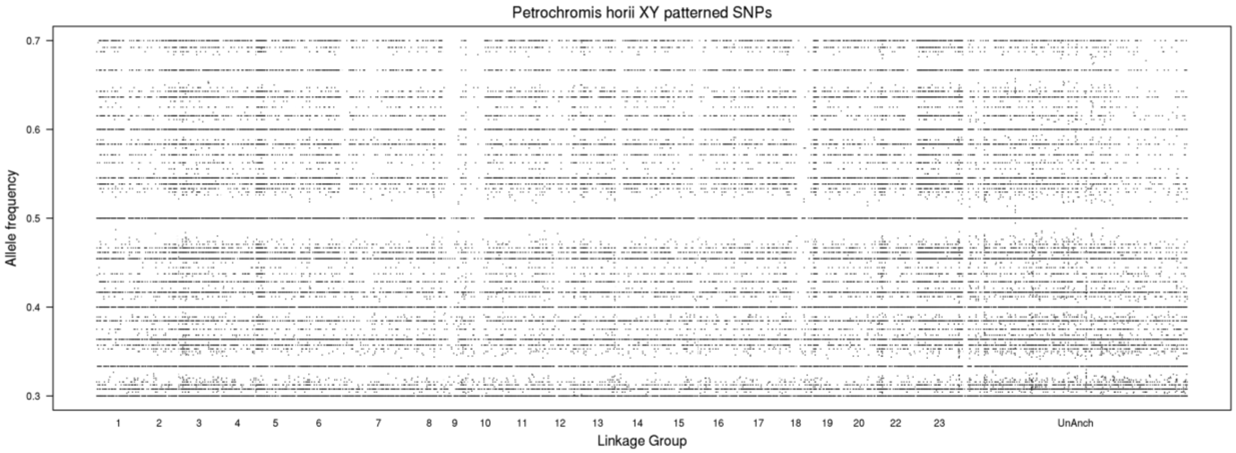

Petrochromis horii XY patterned SNPs
Allele frequency
0.7
0.6
0.5
0.4
0.3
1 2 3 4 5 6 7 8 9 10 11 12 13 14 15 16 17 18 19 20 22 23 UnAnch
Linkage Group

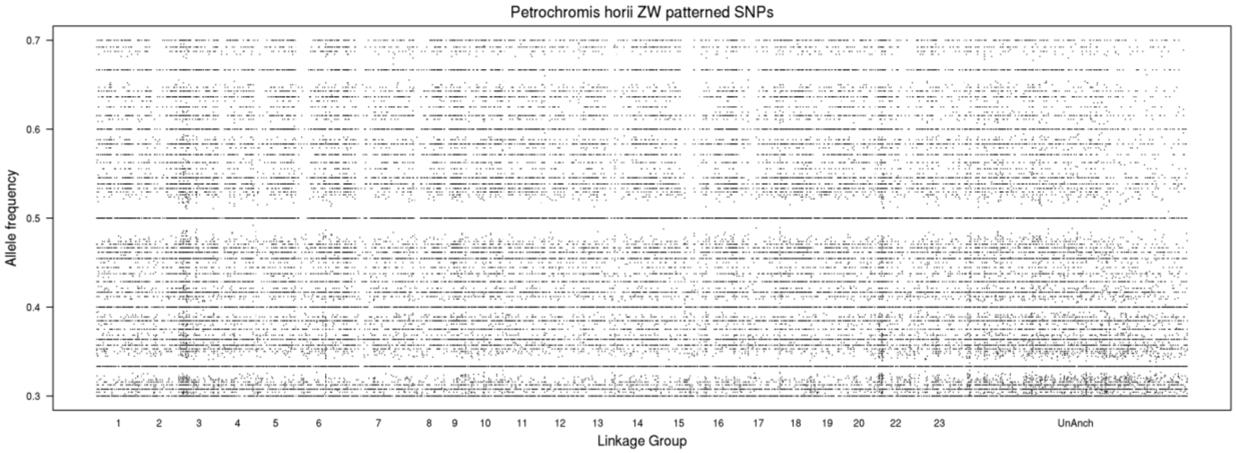

Petrochromis horii ZW patterned SNPs
Allele frequency
0.7
0.6
0.5
0.4
0.3
1 2 3 4 5 6 7 8 9 10 11 12 13 14 15 16 17 18 19 20 22 23 UnAnch
Linkage Group

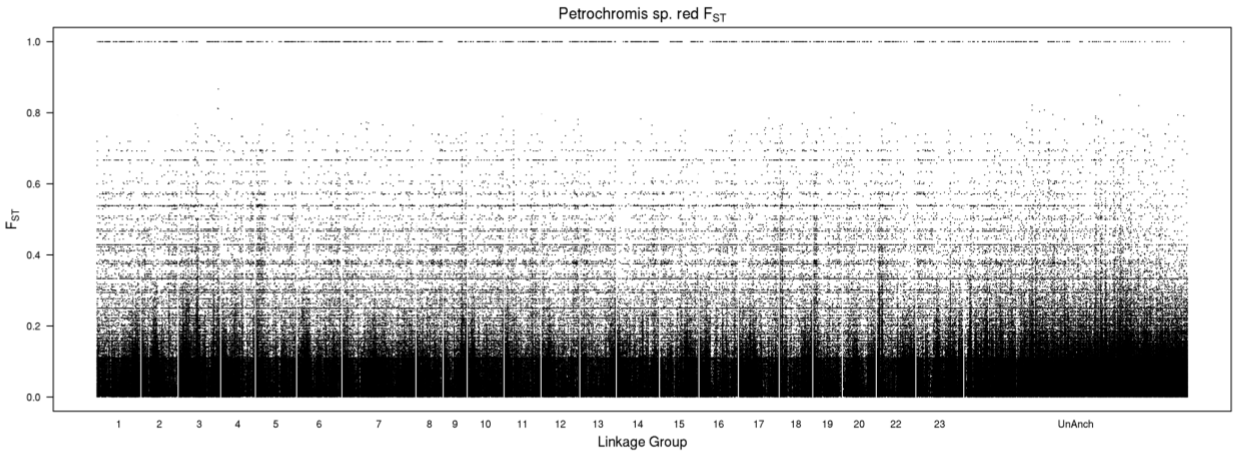
Petrochromis sp. red F_{ST}
F_{ST}
1.0
0.8
0.6
0.4
0.2
0.0
1 2 3 4 5 6 7 8 9 10 11 12 13 14 15 16 17 18 19 20 22 23 UnAnch
Linkage Group

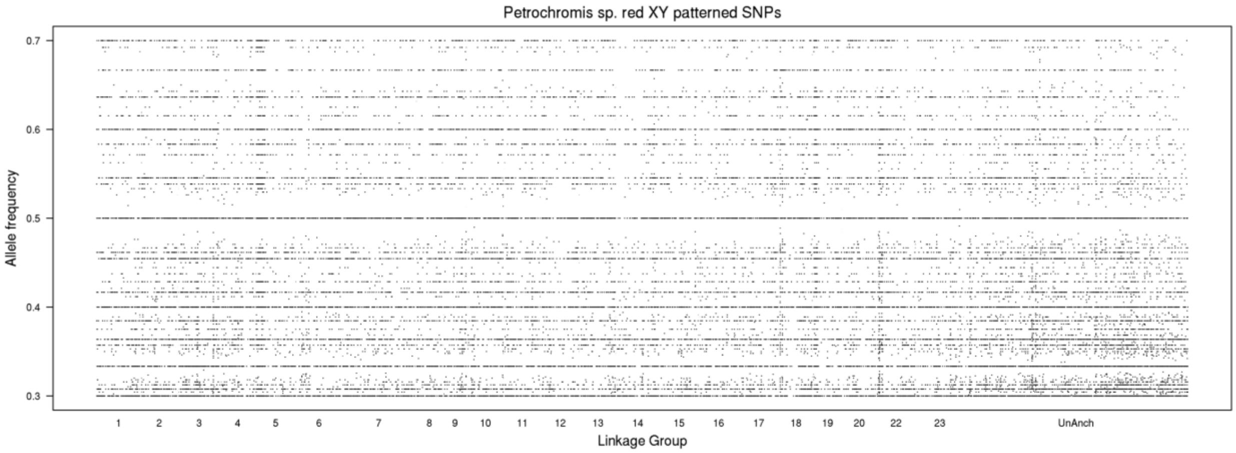
Petrochromis sp. red XY patterned SNPs
Allele frequency
0.7
0.6
0.5
0.4
0.3
1 2 3 4 5 6 7 8 9 10 11 12 13 14 15 16 17 18 19 20 22 23 UnAnch
Linkage Group

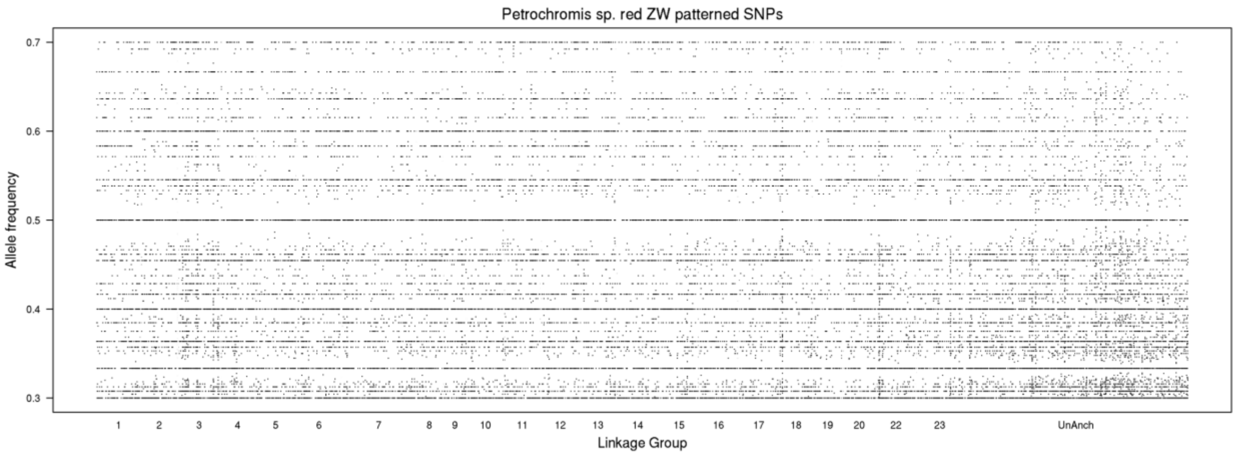
Petrochromis sp. red ZW patterned SNPs
Allele frequency
0.7
0.6
0.5
0.4
0.3
1 2 3 4 5 6 7 8 9 10 11 12 13 14 15 16 17 18 19 20 22 23 UnAnch
Linkage Group

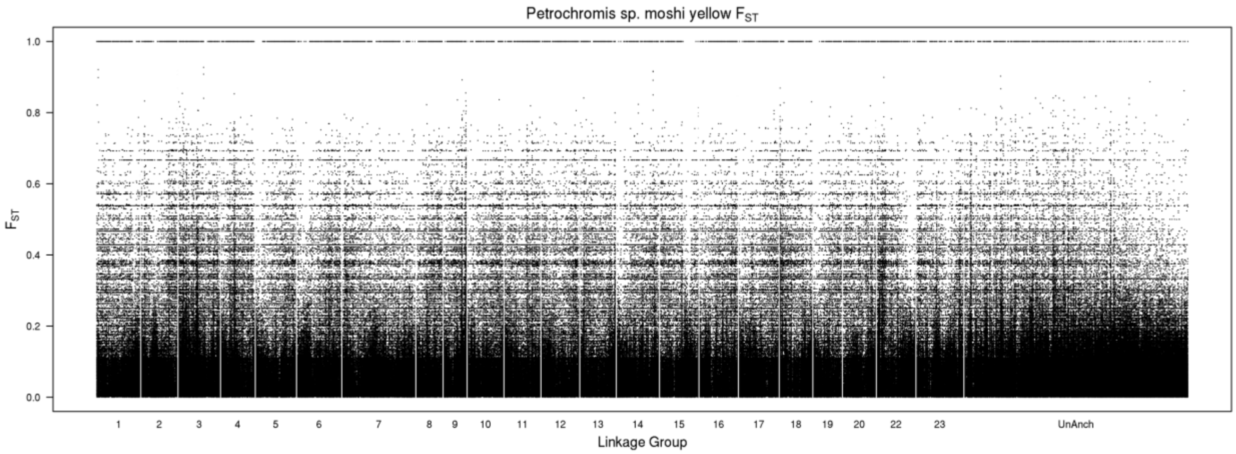
Petrochromis sp. moshi yellow F_{ST}
F_{ST}
0.0 0.2 0.4 0.6 0.8 1.0
1 2 3 4 5 6 7 8 9 10 11 12 13 14 15 16 17 18 19 20 22 23 UnAnch
Linkage Group

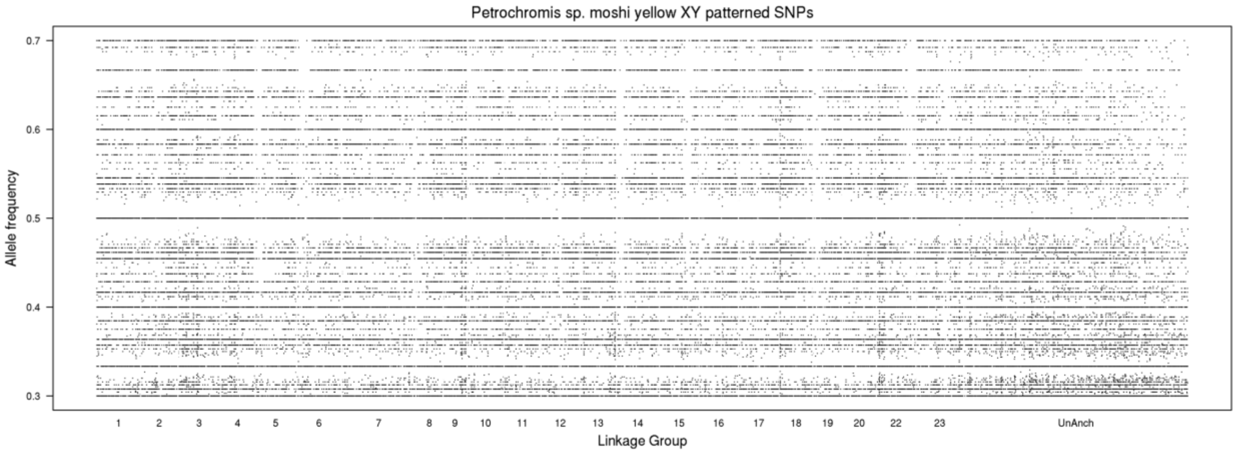
Petrochromis sp. moshi yellow XY patterned SNPs
Allele frequency
0.3 0.4 0.5 0.6 0.7
1 2 3 4 5 6 7 8 9 10 11 12 13 14 15 16 17 18 19 20 22 23 UnAnch
Linkage Group

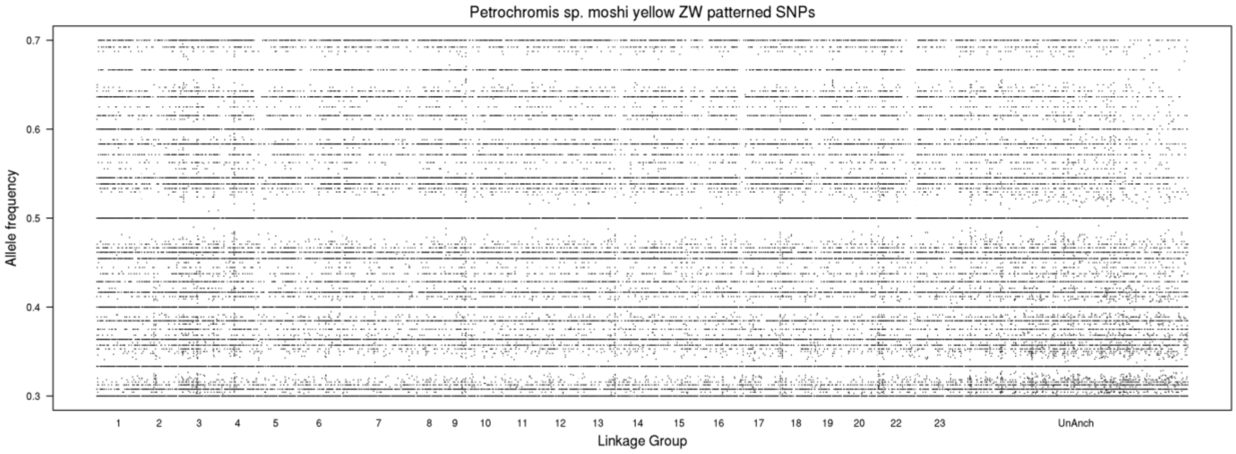
Petrochromis sp. moshi yellow ZW patterned SNPs
Allele frequency
0.3 0.4 0.5 0.6 0.7
1 2 3 4 5 6 7 8 9 10 11 12 13 14 15 16 17 18 19 20 22 23 UnAnch
Linkage Group

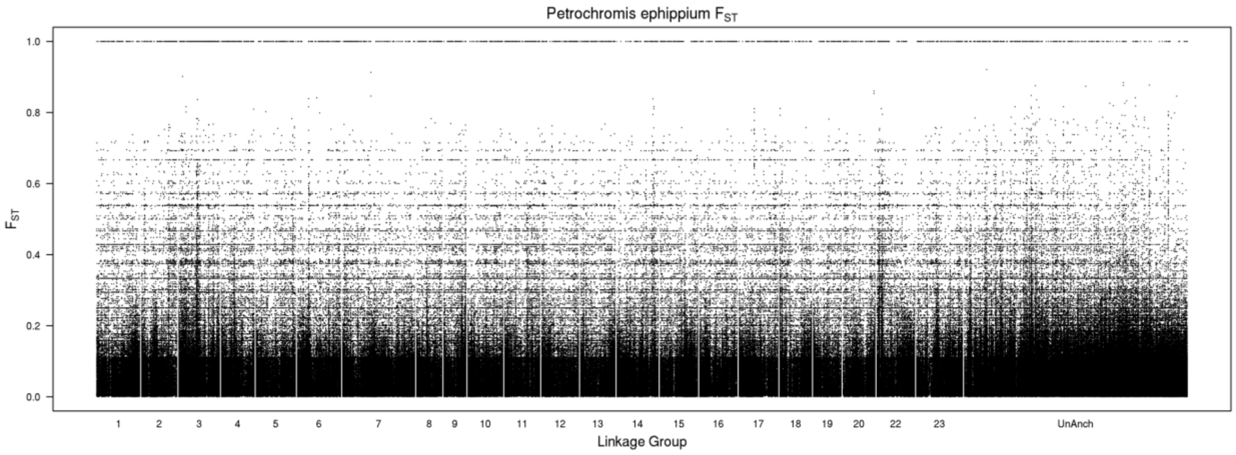
Petrochromis ephippium F_{ST}
F_{ST}
1.0
0.8
0.6
0.4
0.2
0.0
1 2 3 4 5 6 7 8 9 10 11 12 13 14 15 16 17 18 19 20 22 23 UnAnch
Linkage Group

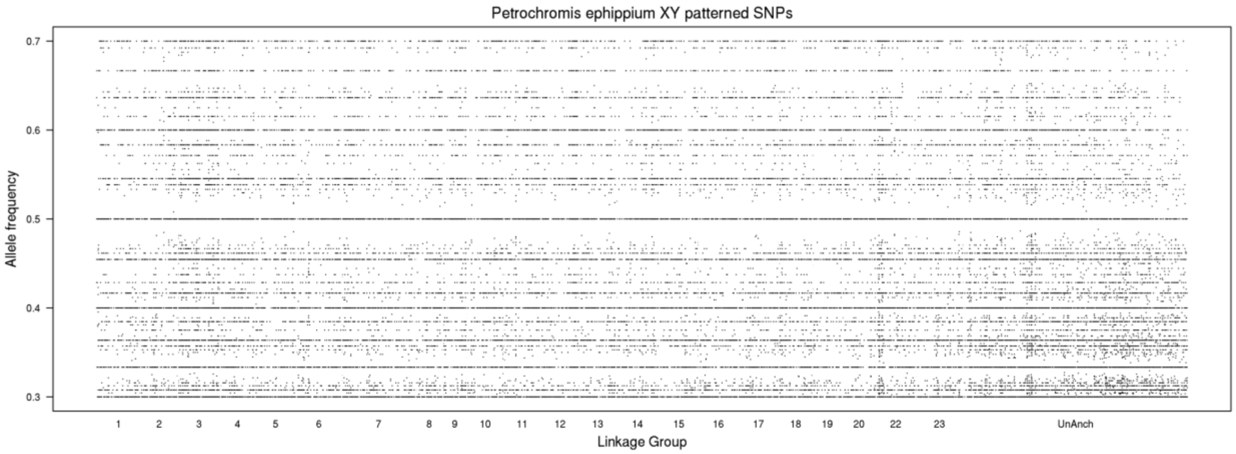
Petrochromis ephippium XY patterned SNPs
Allele frequency
0.7
0.6
0.5
0.4
0.3
1 2 3 4 5 6 7 8 9 10 11 12 13 14 15 16 17 18 19 20 22 23 UnAnch
Linkage Group

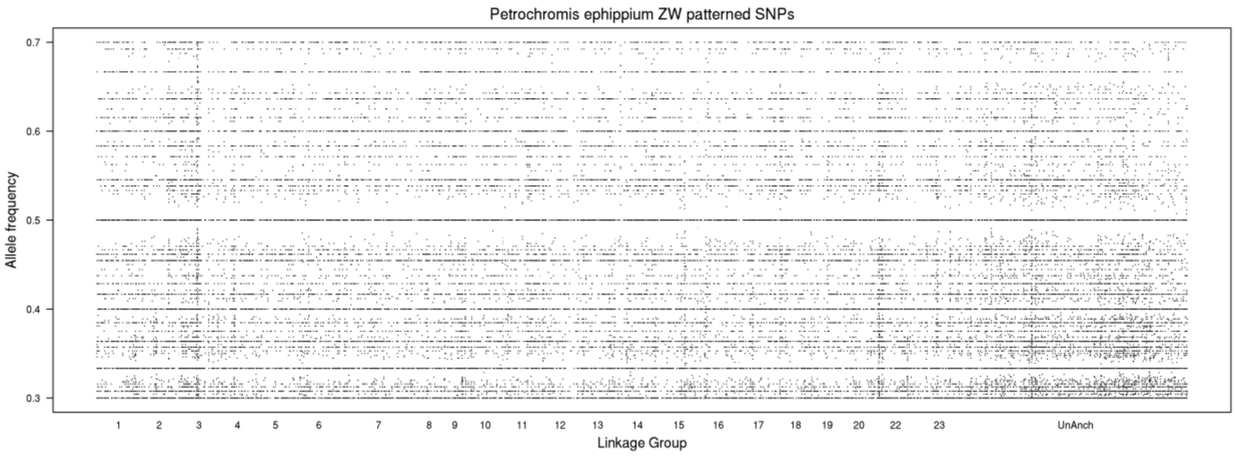
Petrochromis ephippium ZW patterned SNPs
Allele frequency
0.7
0.6
0.5
0.4
0.3
1 2 3 4 5 6 7 8 9 10 11 12 13 14 15 16 17 18 19 20 22 23 UnAnch
Linkage Group

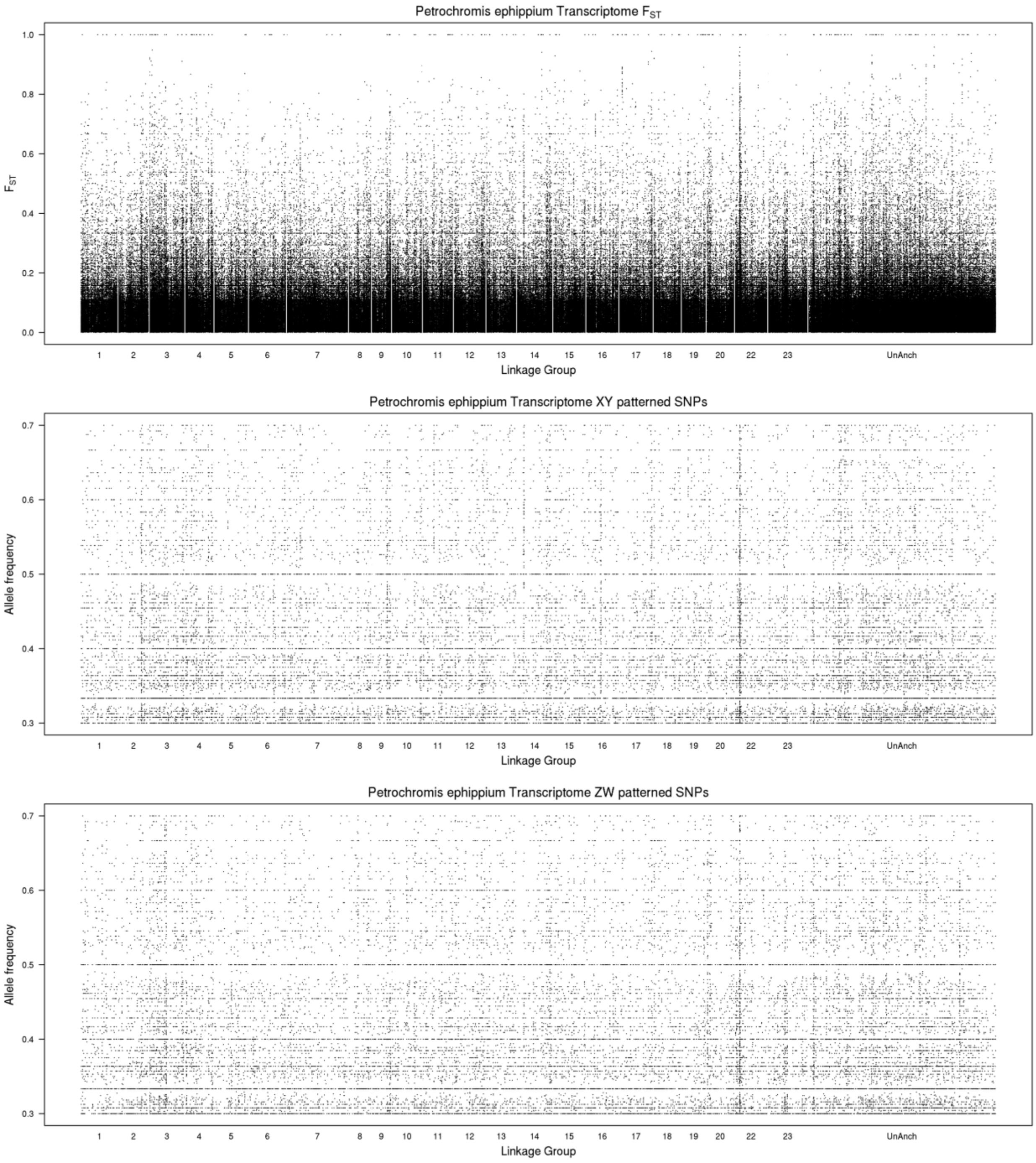
Petrochromis ephippium Transcriptome F_{ST}
F_{ST}
1.0
0.8
0.6
0.4
0.2
0.0
1 2 3 4 5 6 7 8 9 10 11 12 13 14 15 16 17 18 19 20 22 23 UnAnch
Linkage Group
Petrochromis ephippium Transcriptome XY patterned SNPs
Allele frequency
0.7
0.6
0.5
0.4
0.3
1 2 3 4 5 6 7 8 9 10 11 12 13 14 15 16 17 18 19 20 22 23 UnAnch
Linkage Group
Petrochromis ephippium Transcriptome ZW patterned SNPs
Allele frequency
0.7
0.6
0.5
0.4
0.3
1 2 3 4 5 6 7 8 9 10 11 12 13 14 15 16 17 18 19 20 22 23 UnAnch
Linkage Group

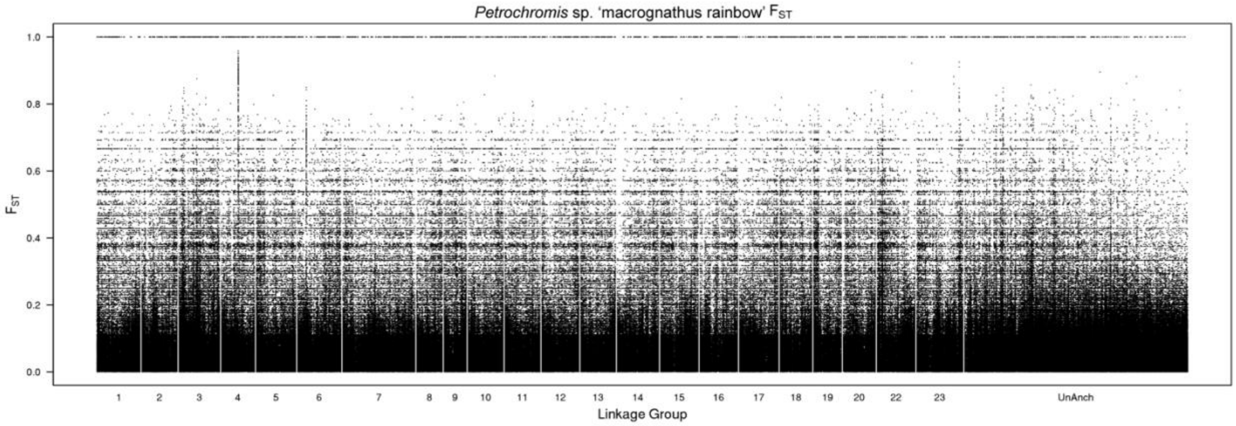
Petrochromis sp. 'macrognathus rainbow' F_ST
F_ST
0.0
0.2
0.4
0.6
0.8
1.0
1 2 3 4 5 6 7 8 9 10 11 12 13 14 15 16 17 18 19 20 22 23 UnAnch
Linkage Group

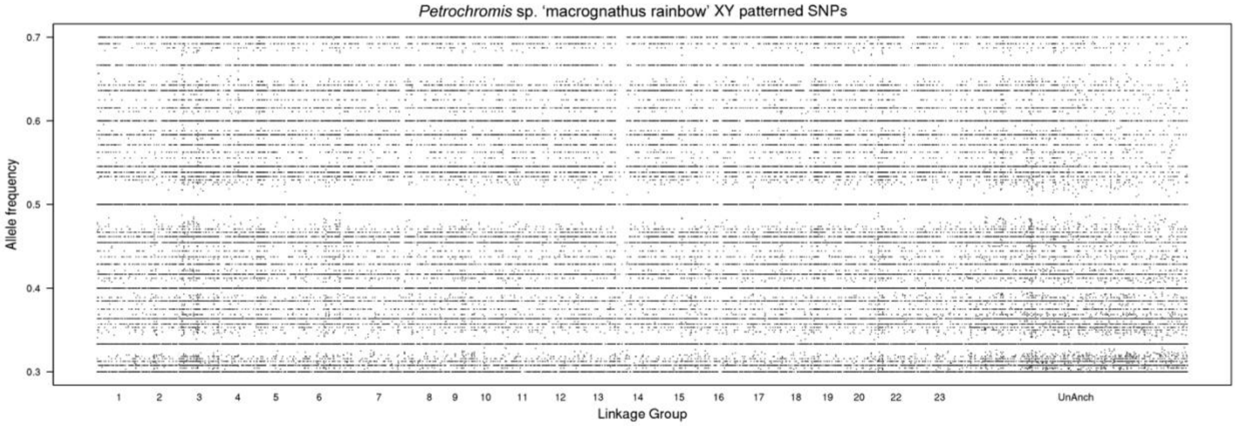
Petrochromis sp. 'macrognathus rainbow' XY patterned SNPs
Allele frequency
0.3
0.4
0.5
0.6
0.7
1 2 3 4 5 6 7 8 9 10 11 12 13 14 15 16 17 18 19 20 22 23 UnAnch
Linkage Group

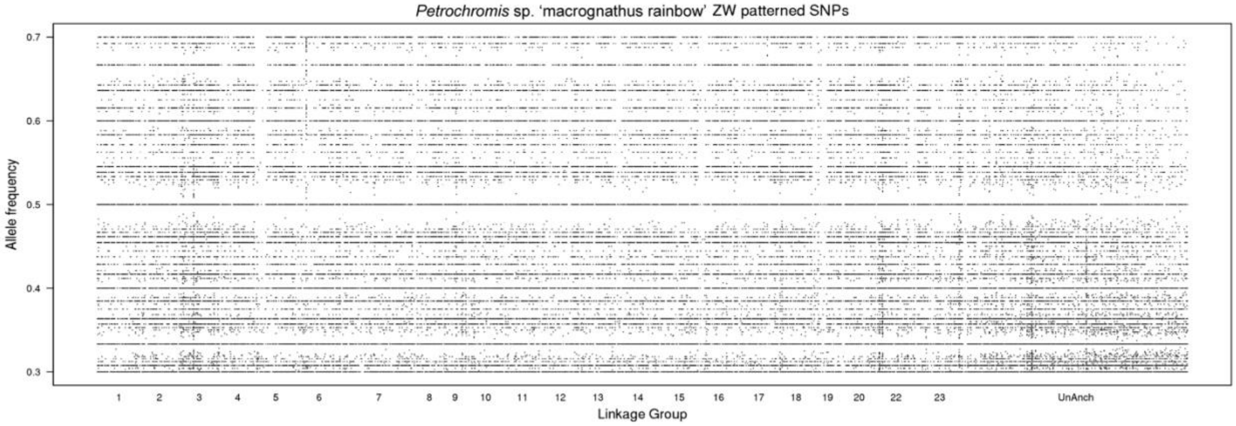
Petrochromis sp. 'macrognathus rainbow' ZW patterned SNPs
Allele frequency
0.3
0.4
0.5
0.6
0.7
1 2 3 4 5 6 7 8 9 10 11 12 13 14 15 16 17 18 19 20 22 23 UnAnch
Linkage Group

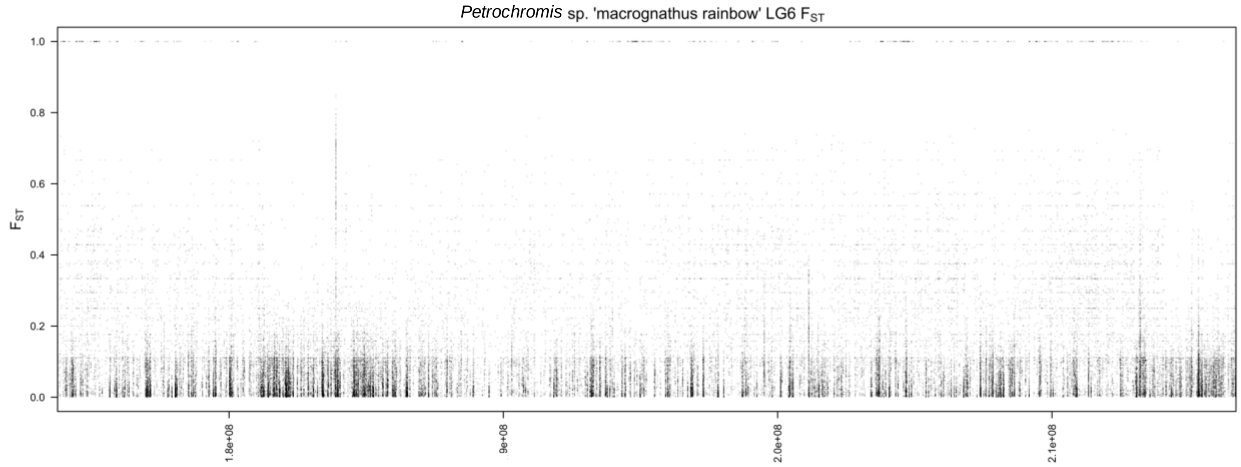
Petrochromis sp. 'macrognathus rainbow' LG6 F_{ST}
F_{ST}
1.0
0.8
0.6
0.4
0.2
0.0
1.8e+08
9e+08
2.0e+08
2.1e+08

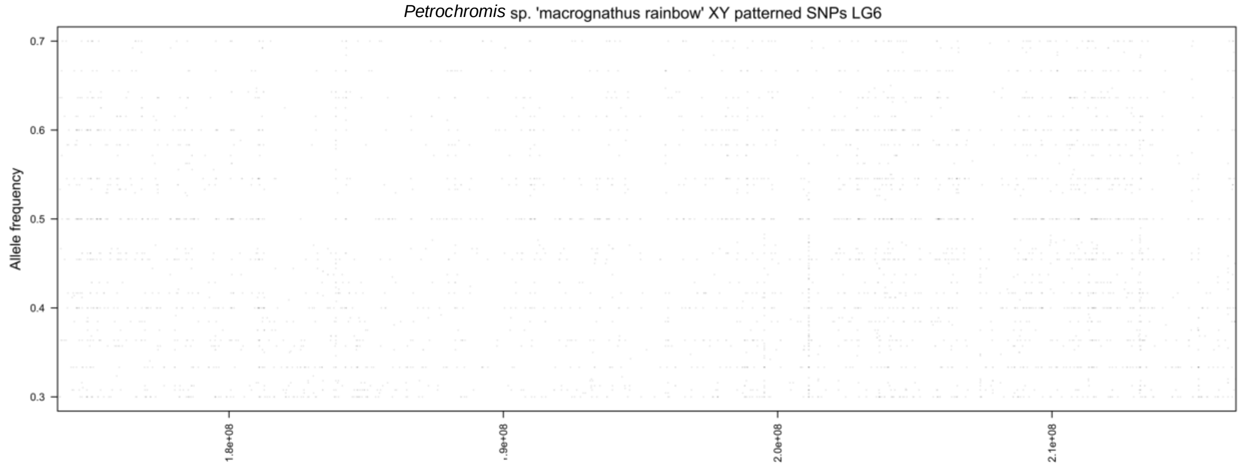
Petrochromis sp. 'macrognathus rainbow' XY patterned SNPs LG6
Allele frequency
0.7
0.6
0.5
0.4
0.3
1.8e+08
.9e+08
2.0e+08
2.1e+08

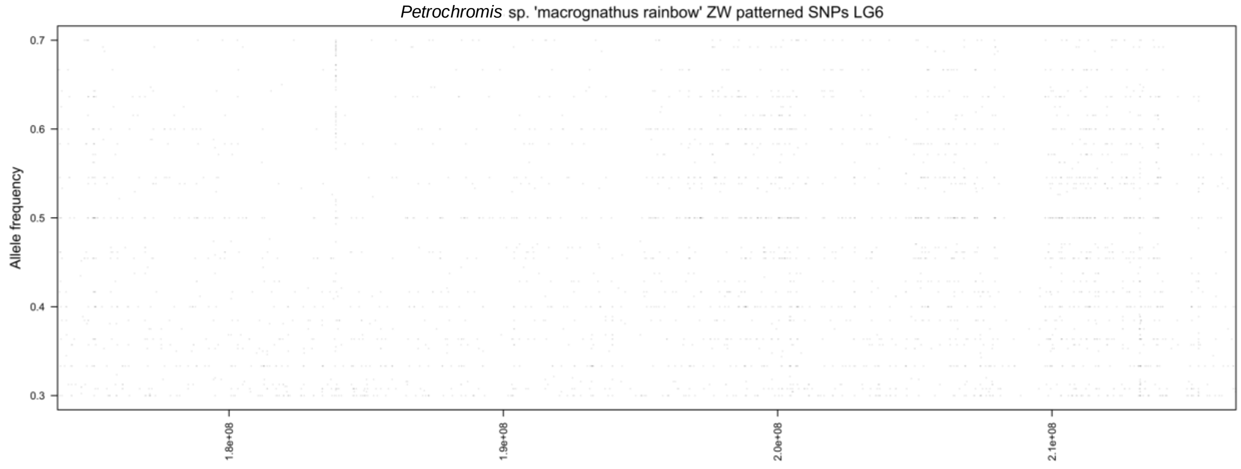
Petrochromis sp. 'macrognathus rainbow' ZW patterned SNPs LG6
Allele frequency
0.7
0.6
0.5
0.4
0.3
1.8e+08
1.9e+08
2.0e+08
2.1e+08

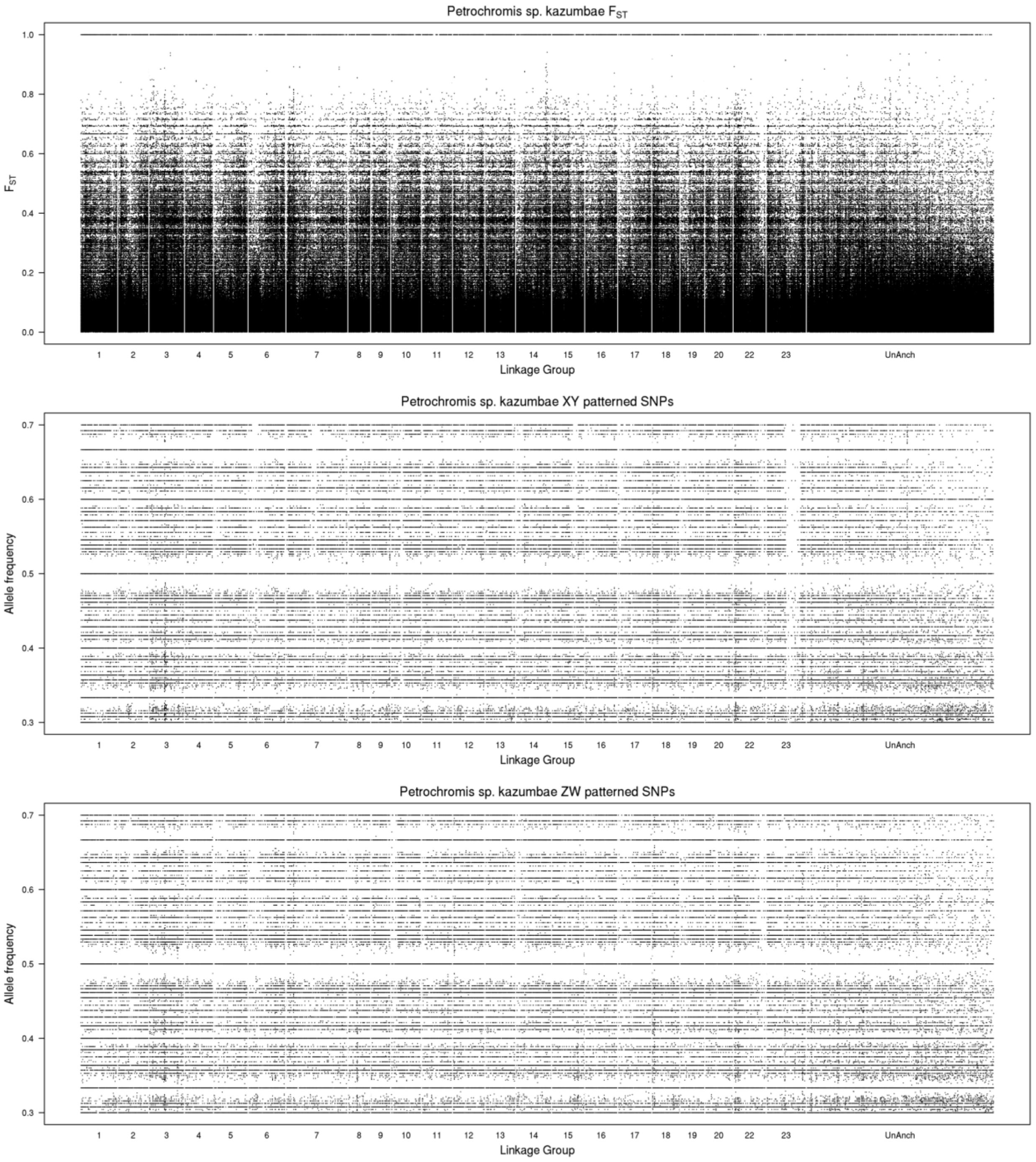
Petrochromis sp. kazumbae F_{ST}
F_{ST}
1.0
0.8
0.6
0.4
0.2
0.0
1 2 3 4 5 6 7 8 9 10 11 12 13 14 15 16 17 18 19 20 22 23 UnAnch
Linkage Group
Petrochromis sp. kazumbae XY patterned SNPs
Allele frequency
0.7
0.6
0.5
0.4
0.3
1 2 3 4 5 6 7 8 9 10 11 12 13 14 15 16 17 18 19 20 22 23 UnAnch
Linkage Group
Petrochromis sp. kazumbae ZW patterned SNPs
Allele frequency
0.7
0.6
0.5
0.4
0.3
1 2 3 4 5 6 7 8 9 10 11 12 13 14 15 16 17 18 19 20 22 23 UnAnch
Linkage Group

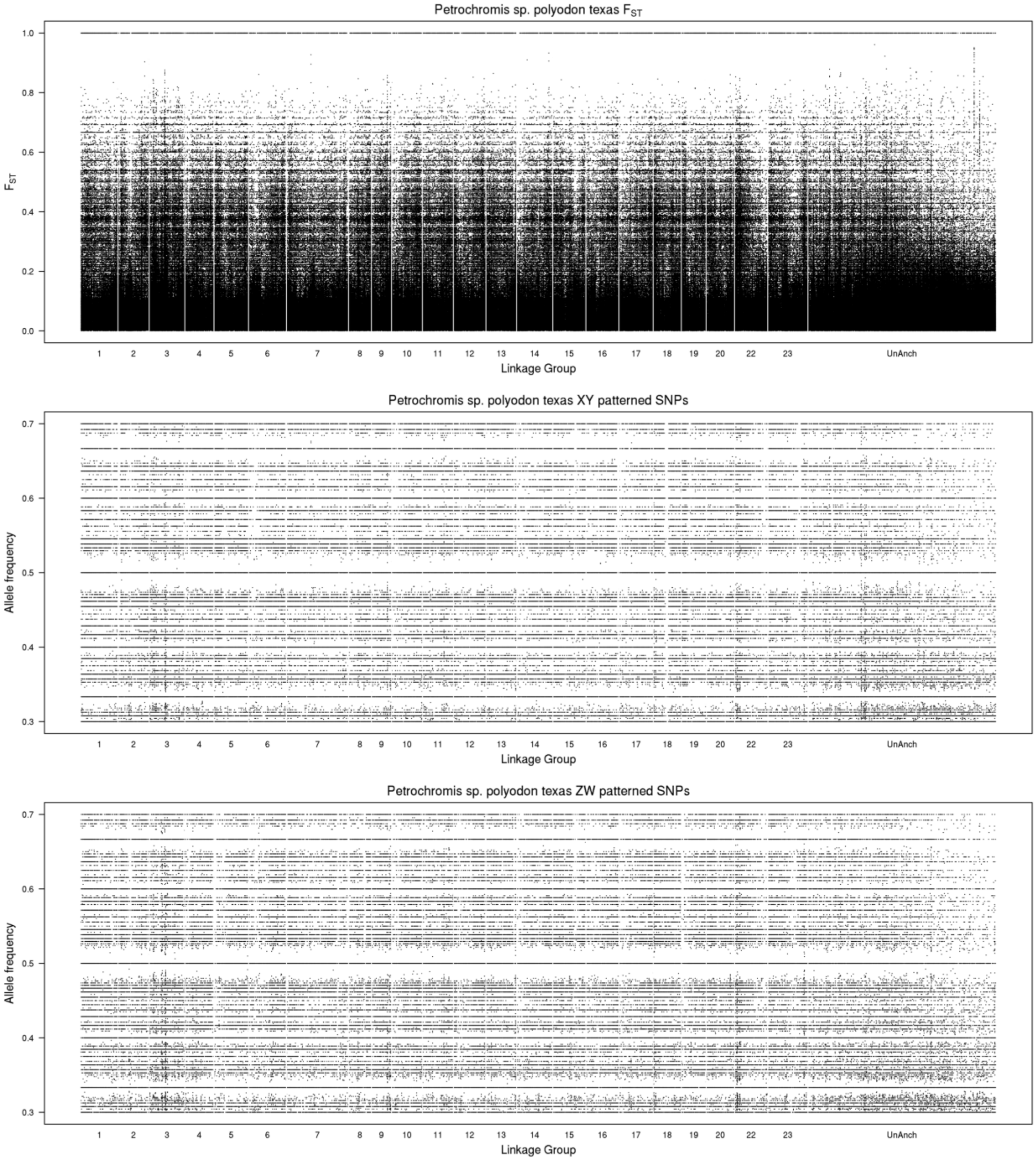

Petrochromis sp. polyodon texas F_{ST}
F_{ST}
Linkage Group
1 2 3 4 5 6 7 8 9 10 11 12 13 14 15 16 17 18 19 20 22 23 UnAnch
Petrochromis sp. polyodon texas XY patterned SNPs
Allele frequency
Linkage Group
Petrochromis sp. polyodon texas ZW patterned SNPs
Allele frequency
Linkage Group

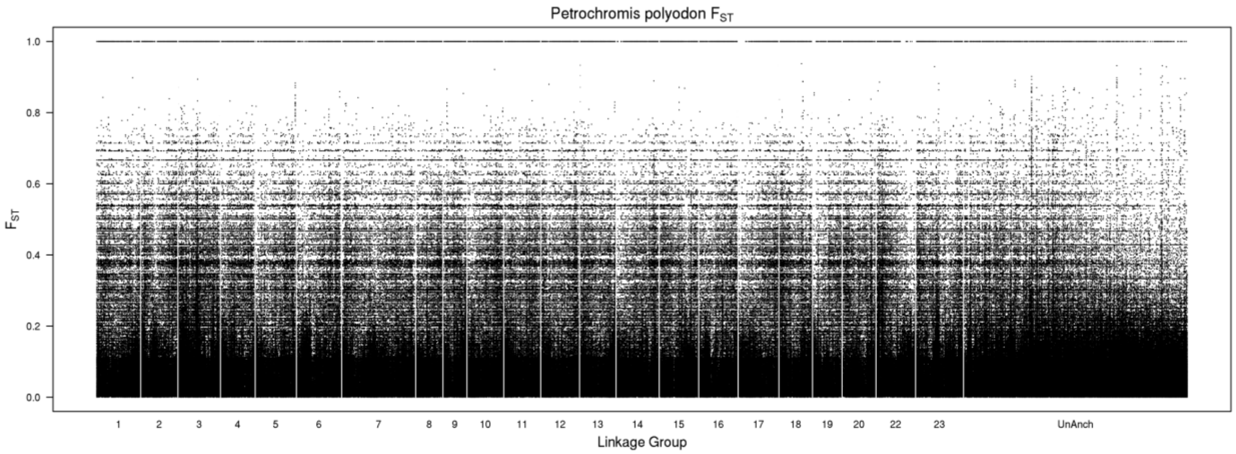
Petrochromis polyodon F_{ST}
F_{ST}
1.0
0.8
0.6
0.4
0.2
0.0
1 2 3 4 5 6 7 8 9 10 11 12 13 14 15 16 17 18 19 20 22 23 UnAnch
Linkage Group

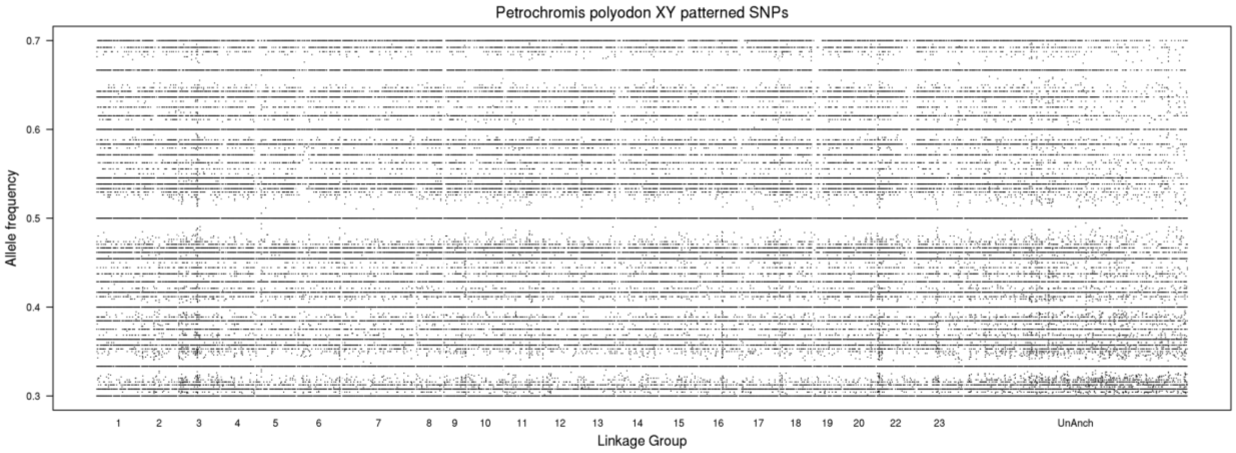
Petrochromis polyodon XY patterned SNPs
Allele frequency
0.7
0.6
0.5
0.4
0.3
1 2 3 4 5 6 7 8 9 10 11 12 13 14 15 16 17 18 19 20 22 23 UnAnch
Linkage Group

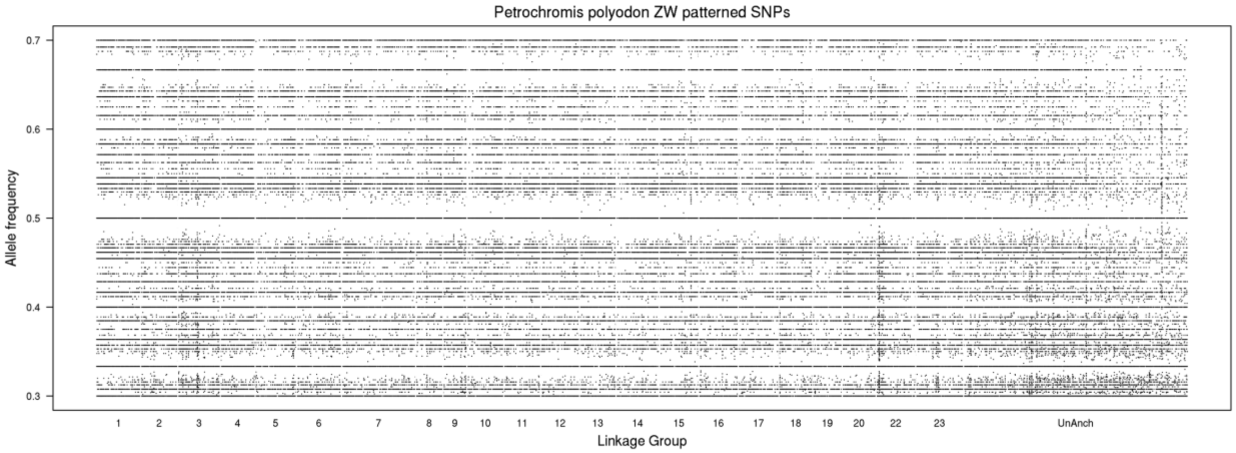
Petrochromis polyodon ZW patterned SNPs
Allele frequency
0.7
0.6
0.5
0.4
0.3
1 2 3 4 5 6 7 8 9 10 11 12 13 14 15 16 17 18 19 20 22 23 UnAnch
Linkage Group

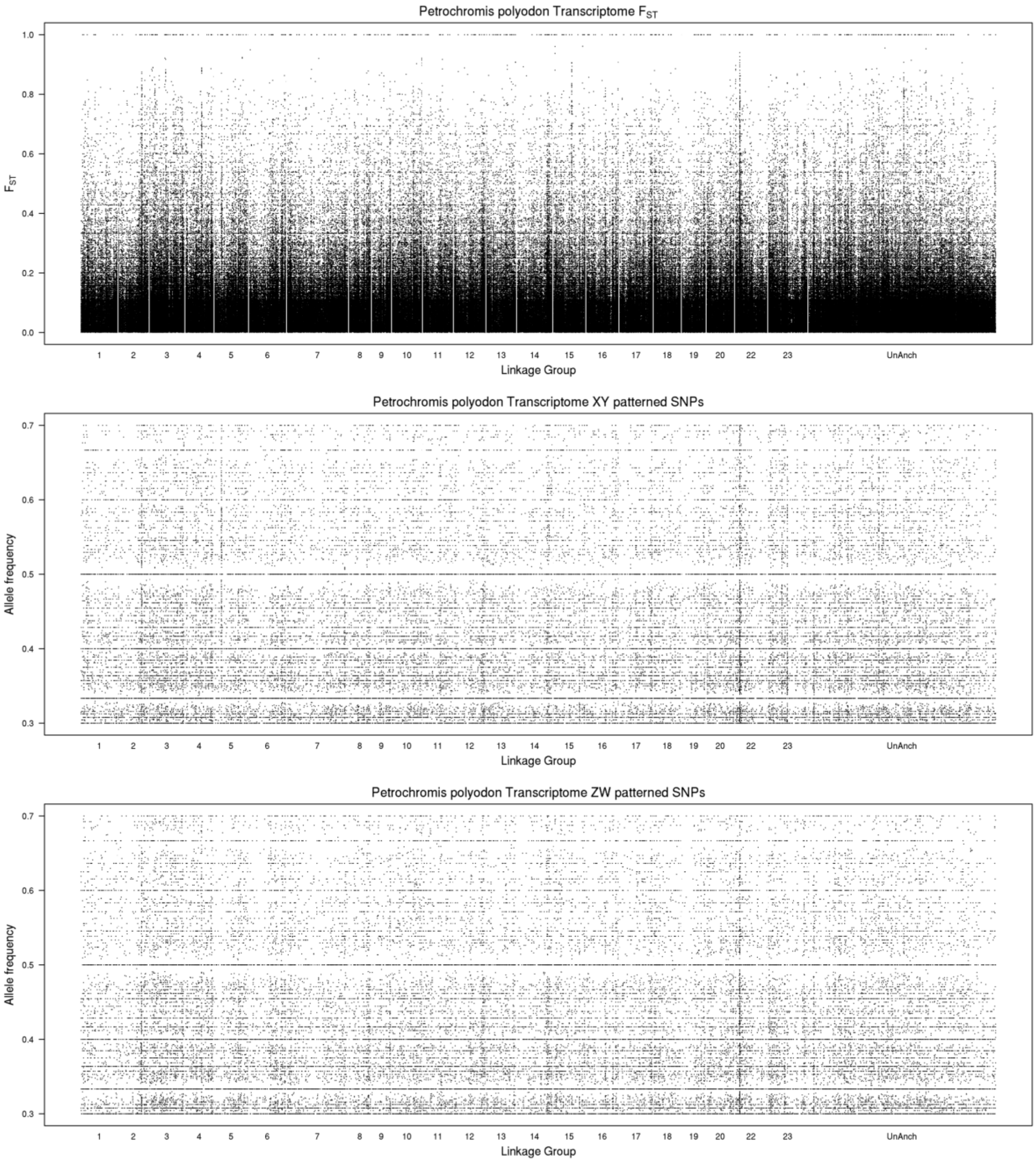

Petrochromis polyodon Transcriptome F_ST
F_ST
1.0
0.8
0.6
0.4
0.2
0.0
1 2 3 4 5 6 7 8 9 10 11 12 13 14 15 16 17 18 19 20 22 23 UnAnch
Linkage Group
Petrochromis polyodon Transcriptome XY patterned SNPs
Allele frequency
0.7
0.6
0.5
0.4
0.3
1 2 3 4 5 6 7 8 9 10 11 12 13 14 15 16 17 18 19 20 22 23 UnAnch
Linkage Group
Petrochromis polyodon Transcriptome ZW patterned SNPs
Allele frequency
0.7
0.6
0.5
0.4
0.3
1 2 3 4 5 6 7 8 9 10 11 12 13 14 15 16 17 18 19 20 22 23 UnAnch
Linkage Group

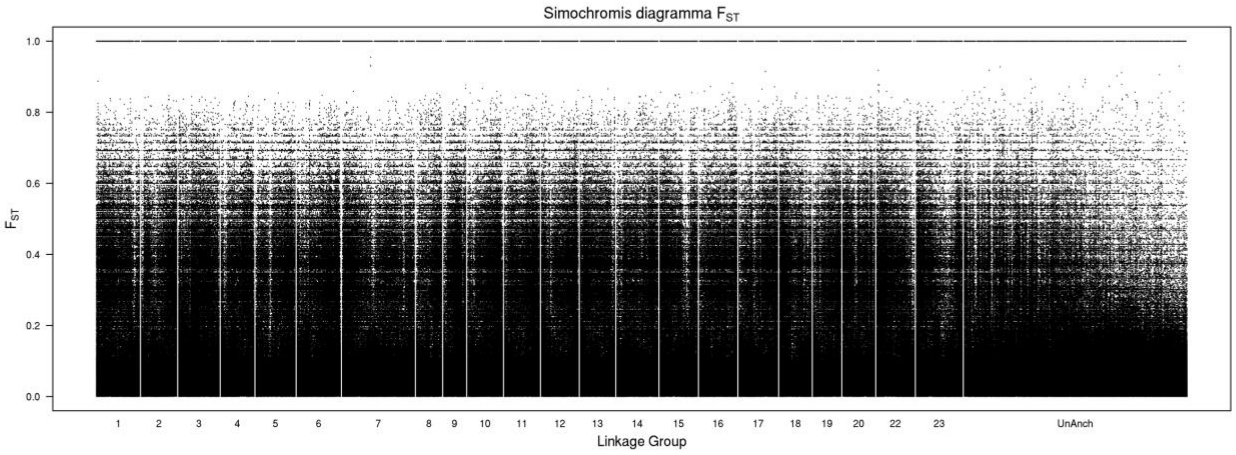
Simochromis diagramma F_{ST}
F_{ST}
0.0
0.2
0.4
0.6
0.8
1.0
1 2 3 4 5 6 7 8 9 10 11 12 13 14 15 16 17 18 19 20 22 23 UnAnch
Linkage Group

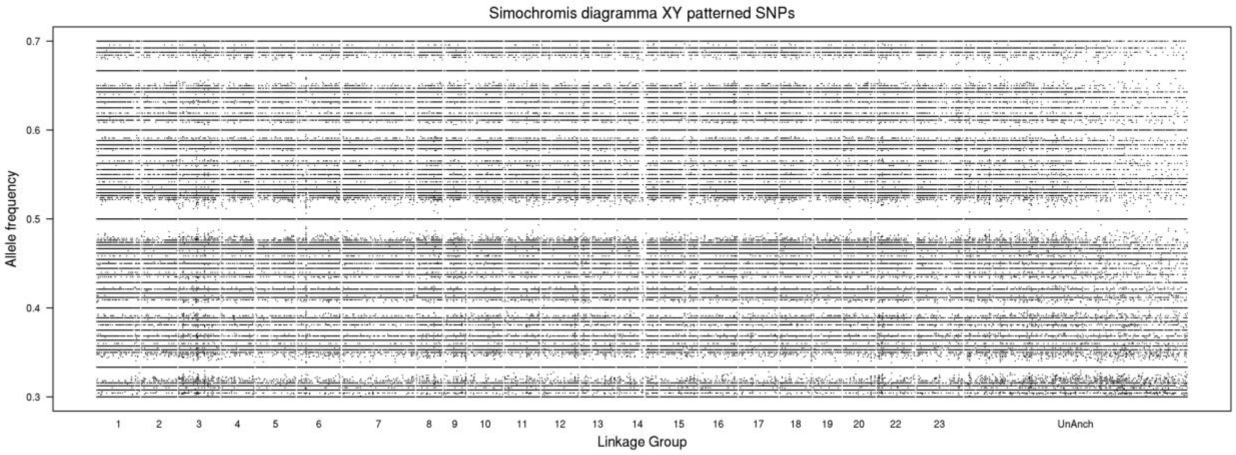
Simochromis diagramma XY patterned SNPs
Allele frequency
0.3
0.4
0.5
0.6
0.7
1 2 3 4 5 6 7 8 9 10 11 12 13 14 15 16 17 18 19 20 22 23 UnAnch
Linkage Group

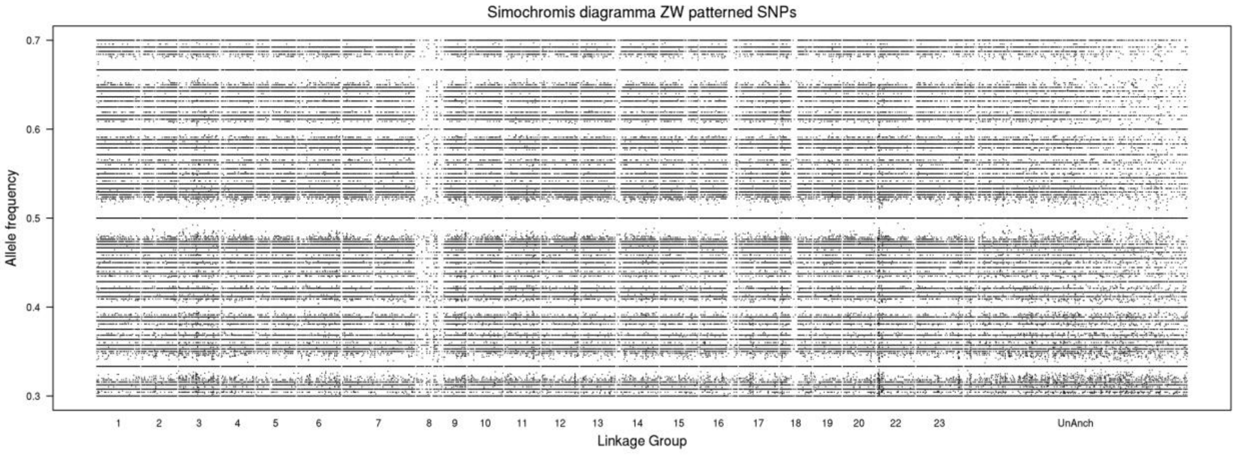
Simochromis diagramma ZW patterned SNPs
Allele frequency
0.3
0.4
0.5
0.6
0.7
1 2 3 4 5 6 7 8 9 10 11 12 13 14 15 16 17 18 19 20 22 23 UnAnch
Linkage Group

Simochromis diagramma Transcriptome F_{ST}

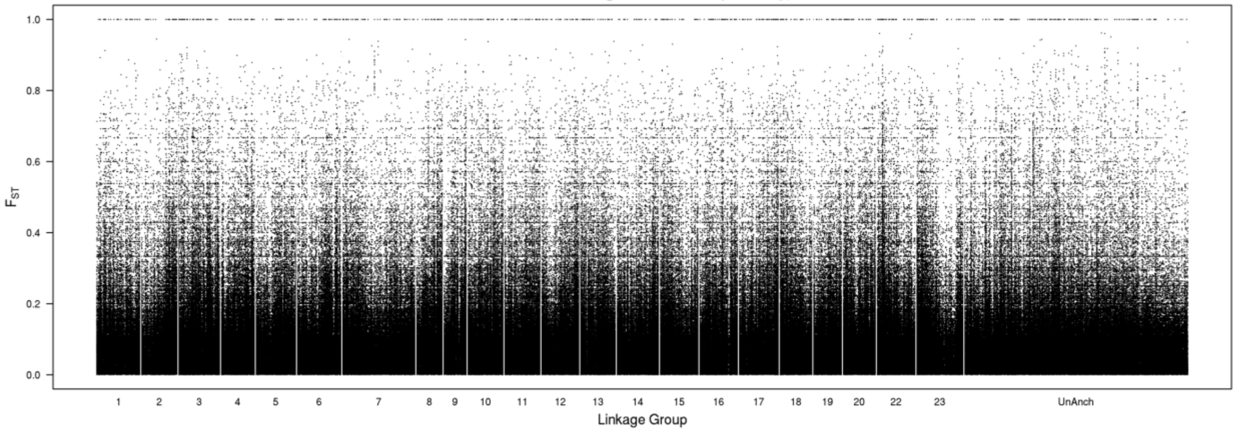

Simochromis diagramma Transcriptome XY patterned SNPs

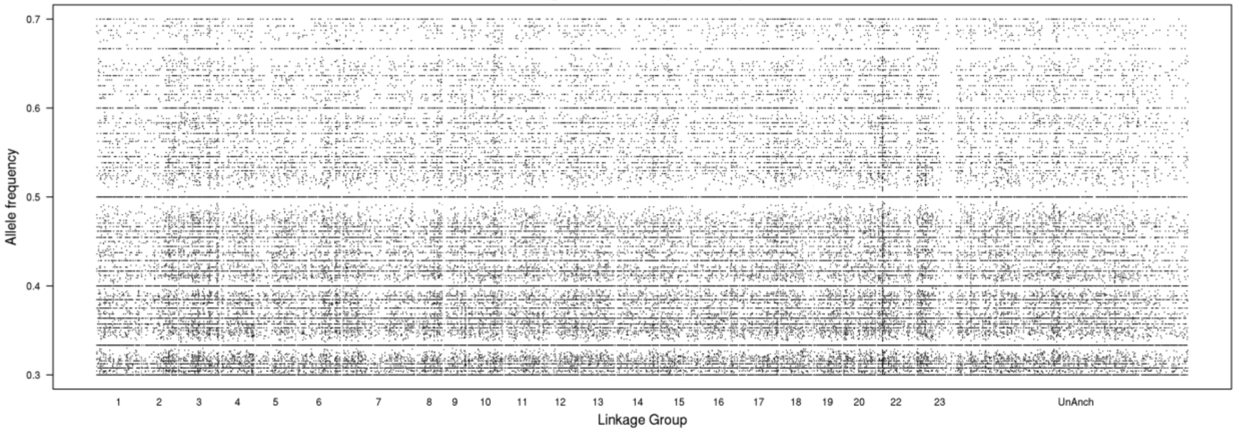

Simochromis diagramma Transcriptome ZW patterned SNPs

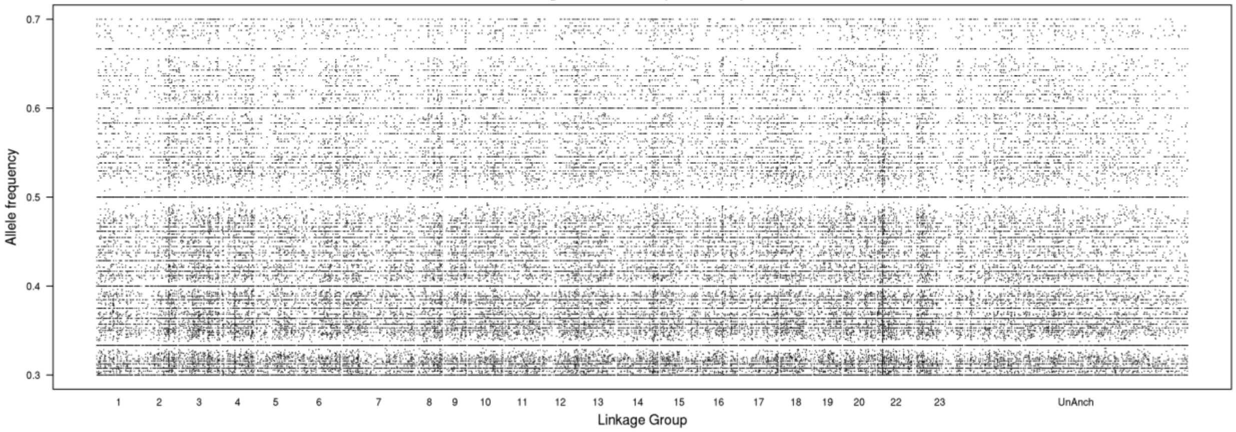

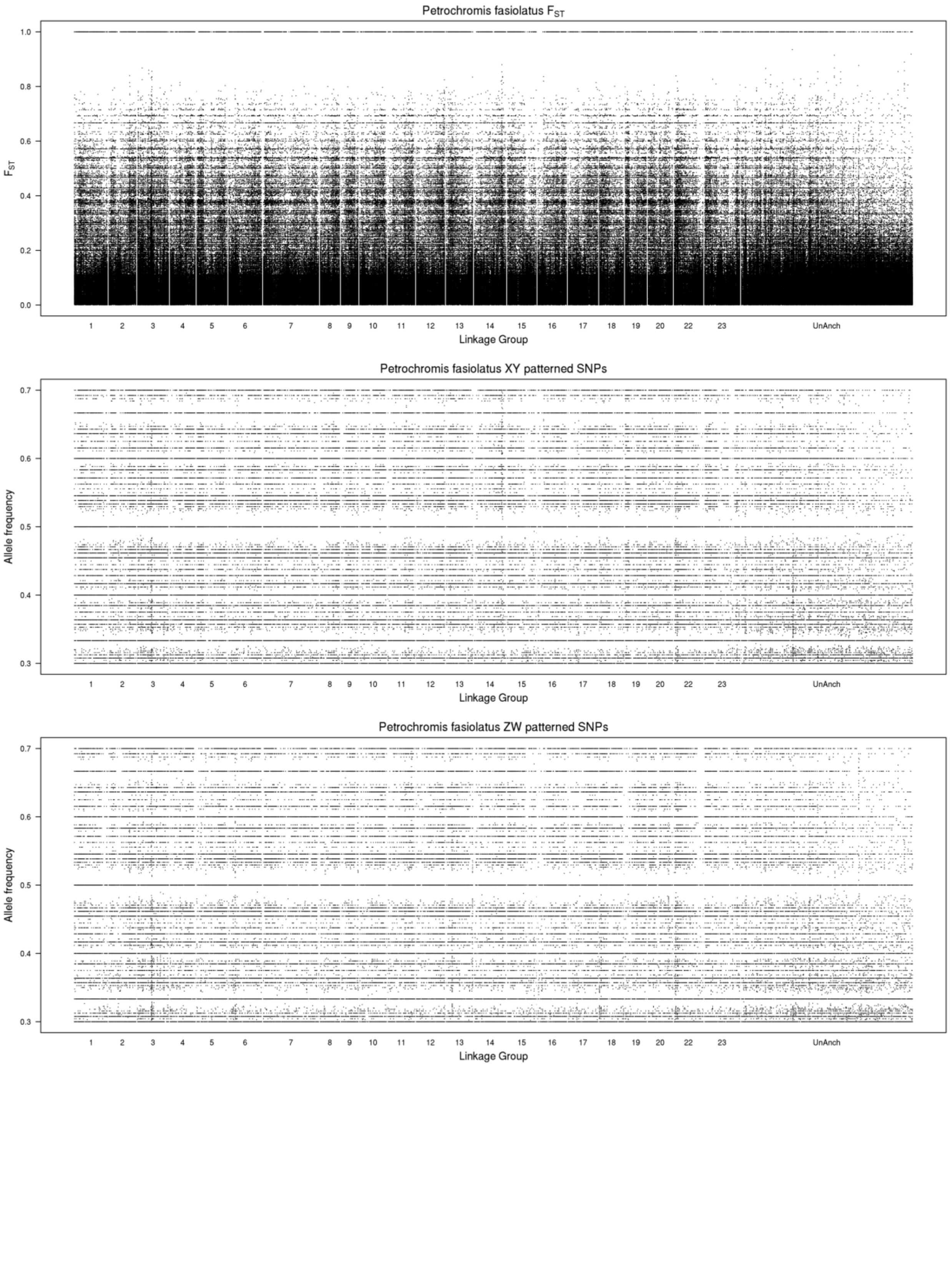
Petrochromis fasiolatus F_ST
1.0
0.8
0.6
0.4
0.2
0.0
F_ST
1 2 3 4 5 6 7 8 9 10 11 12 13 14 15 16 17 18 19 20 22 23 UnAnch
Linkage Group
Petrochromis fasiolatus XY patterned SNPs
0.7
0.6
0.5
0.4
0.3
Allele frequency
1 2 3 4 5 6 7 8 9 10 11 12 13 14 15 16 17 18 19 20 22 23 UnAnch
Linkage Group
Petrochromis fasiolatus ZW patterned SNPs
0.7
0.6
0.5
0.4
0.3
Allele frequency
1 2 3 4 5 6 7 8 9 10 11 12 13 14 15 16 17 18 19 20 22 23 UnAnch
Linkage Group

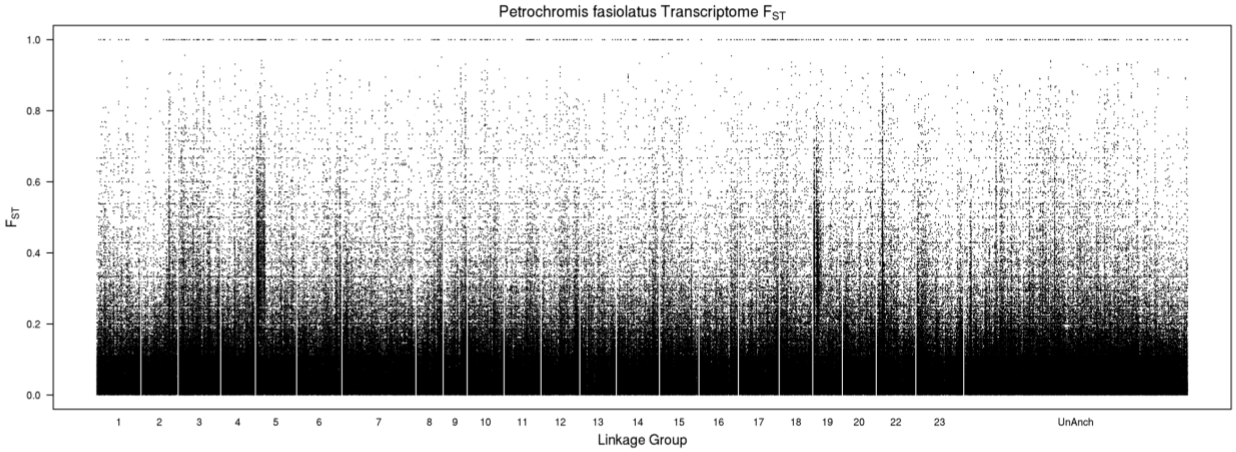
Petrochromis fasiolatus Transcriptome F_{ST}
F_{ST}
0.0
0.2
0.4
0.6
0.8
1.0
1 2 3 4 5 6 7 8 9 10 11 12 13 14 15 16 17 18 19 20 22 23 UnAnch
Linkage Group

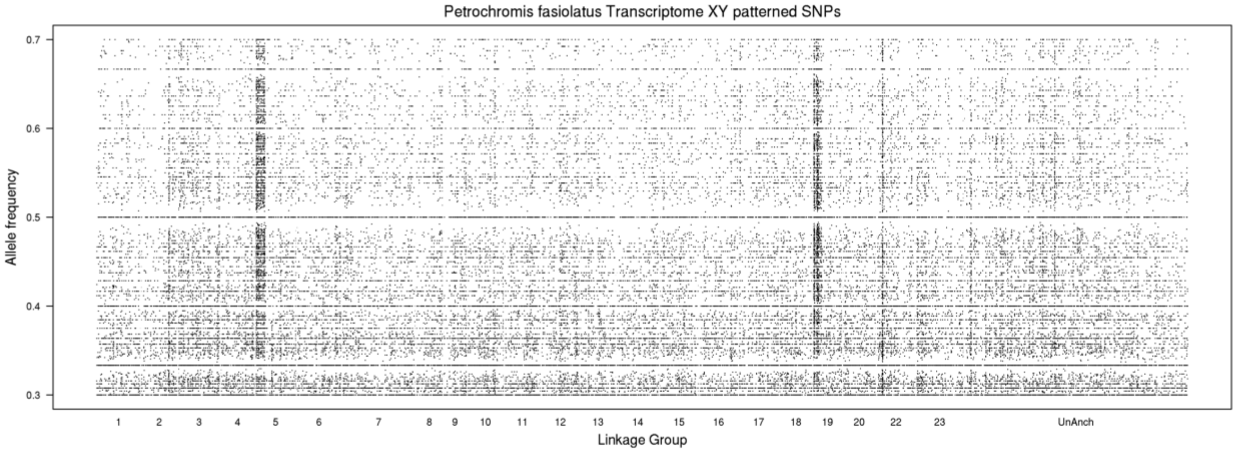
Petrochromis fasiolatus Transcriptome XY patterned SNPs
Allele frequency
0.3
0.4
0.5
0.6
0.7
1 2 3 4 5 6 7 8 9 10 11 12 13 14 15 16 17 18 19 20 22 23 UnAnch
Linkage Group

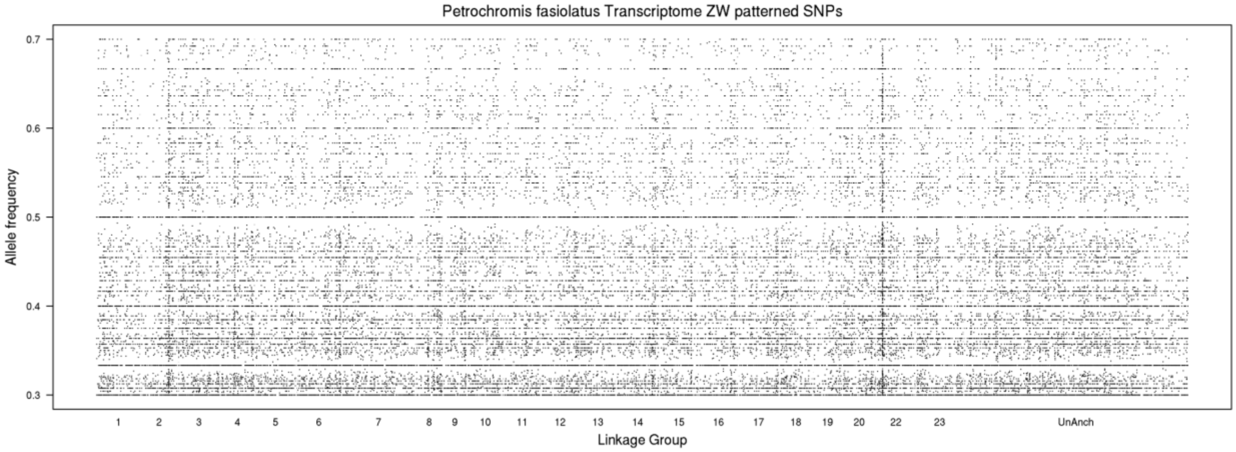
Petrochromis fasiolatus Transcriptome ZW patterned SNPs
Allele frequency
0.3
0.4
0.5
0.6
0.7
1 2 3 4 5 6 7 8 9 10 11 12 13 14 15 16 17 18 19 20 22 23 UnAnch
Linkage Group

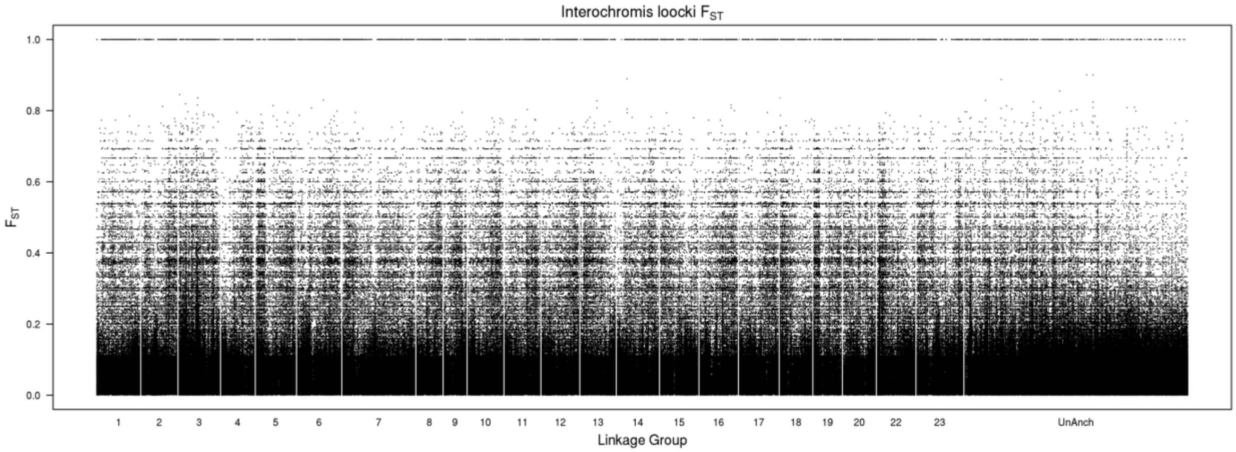
Interochromis loocki F_{ST}
F_{ST}
1.0
0.8
0.6
0.4
0.2
0.0
1 2 3 4 5 6 7 8 9 10 11 12 13 14 15 16 17 18 19 20 22 23 UnAnch
Linkage Group

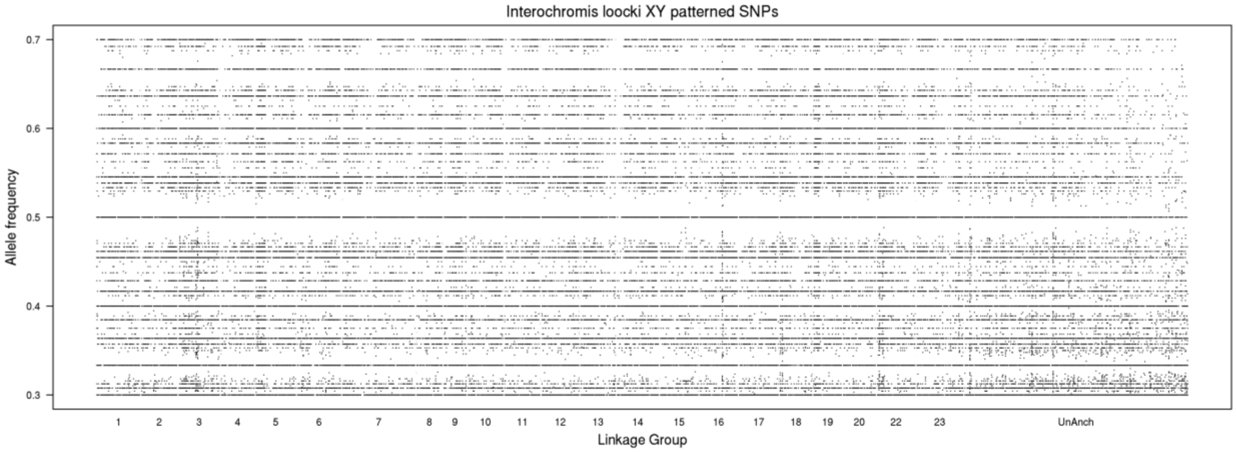
Interochromis loocki XY patterned SNPs
Allele frequency
0.7
0.6
0.5
0.4
0.3
1 2 3 4 5 6 7 8 9 10 11 12 13 14 15 16 17 18 19 20 22 23 UnAnch
Linkage Group

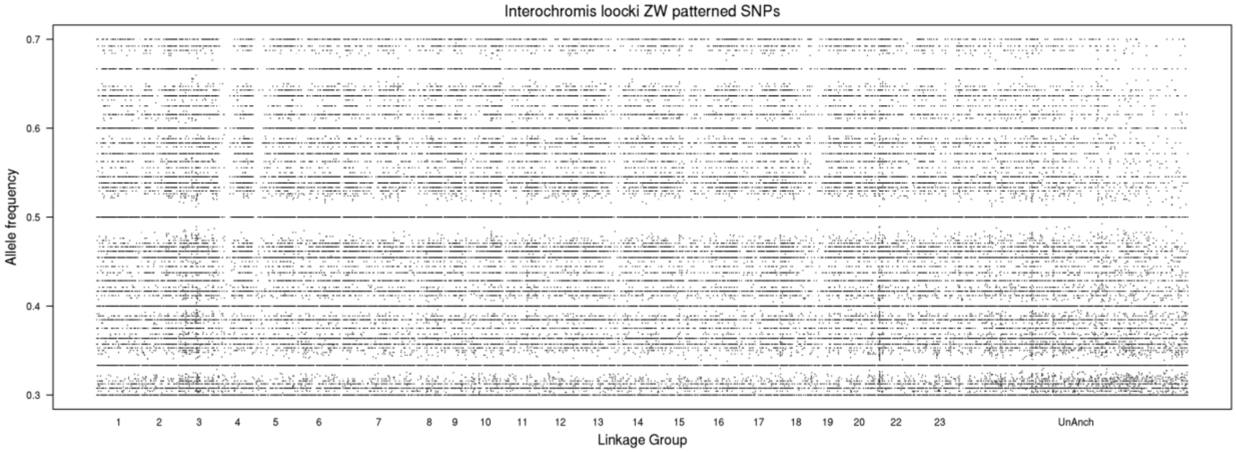
Interochromis loocki ZW patterned SNPs
Allele frequency
0.7
0.6
0.5
0.4
0.3
1 2 3 4 5 6 7 8 9 10 11 12 13 14 15 16 17 18 19 20 22 23 UnAnch
Linkage Group

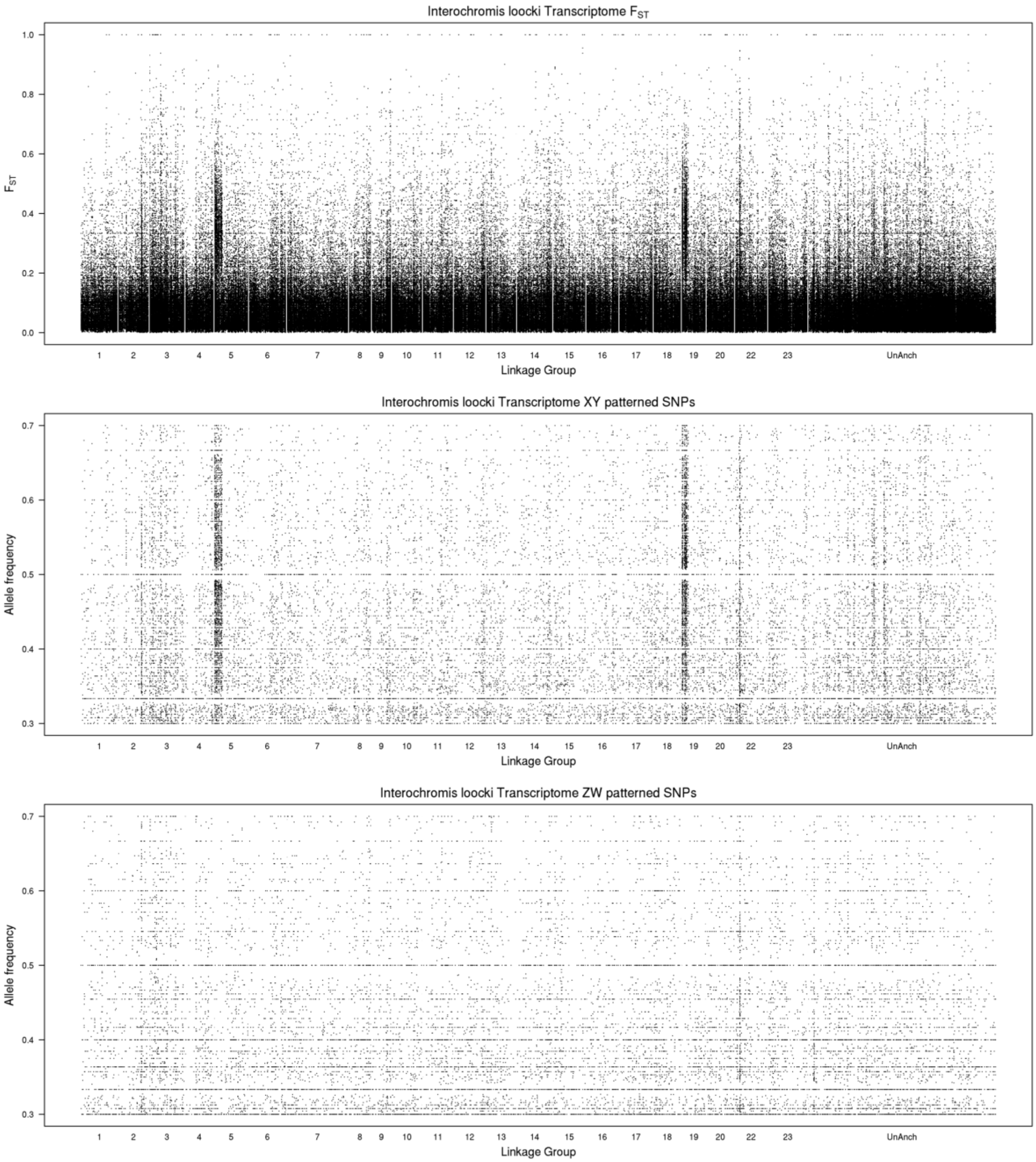

Interochromis loocki Transcriptome F_{ST}
F_{ST}
1.0
0.8
0.6
0.4
0.2
0.0
1 2 3 4 5 6 7 8 9 10 11 12 13 14 15 16 17 18 19 20 22 23 UnAnch
Linkage Group
Interochromis loocki Transcriptome XY patterned SNPs
Allele frequency
0.7
0.6
0.5
0.4
0.3
1 2 3 4 5 6 7 8 9 10 11 12 13 14 15 16 17 18 19 20 22 23 UnAnch
Linkage Group
Interochromis loocki Transcriptome ZW patterned SNPs
Allele frequency
0.7
0.6
0.5
0.4
0.3
1 2 3 4 5 6 7 8 9 10 11 12 13 14 15 16 17 18 19 20 22 23 UnAnch
Linkage Group

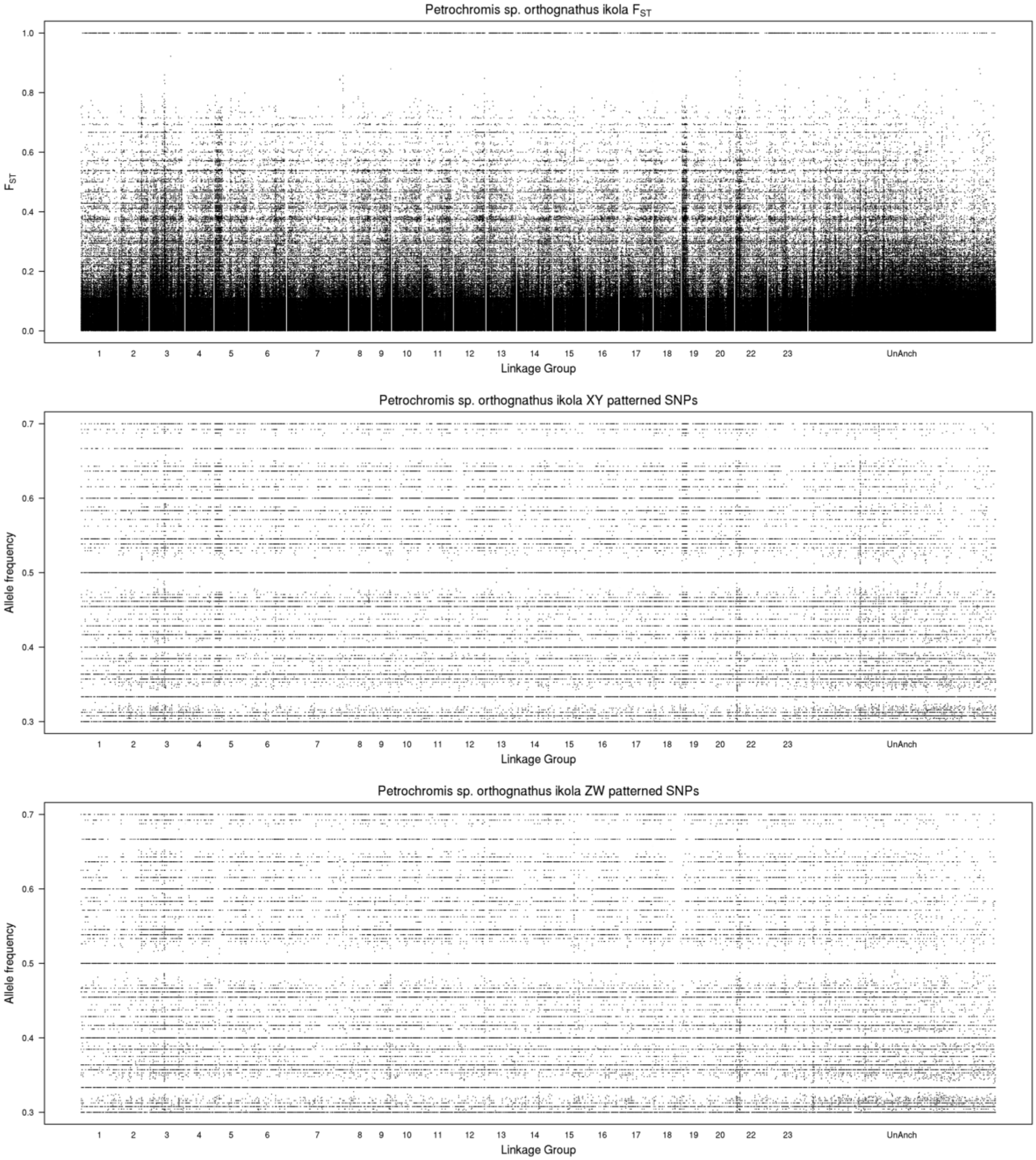

Petrochromis sp. orthognathus ikola F_{ST}
F_{ST}
1.0
0.8
0.6
0.4
0.2
0.0
1
2
3
4
5
6
7
8
9
10
11
12
13
14
15
16
17
18
19
20
22
23
UnAnch
Linkage Group
Petrochromis sp. orthognathus ikola XY patterned SNPs
Allele frequency
0.7
0.6
0.5
0.4
0.3
Linkage Group
Petrochromis sp. orthognathus ikola ZW patterned SNPs
Allele frequency
Linkage Group

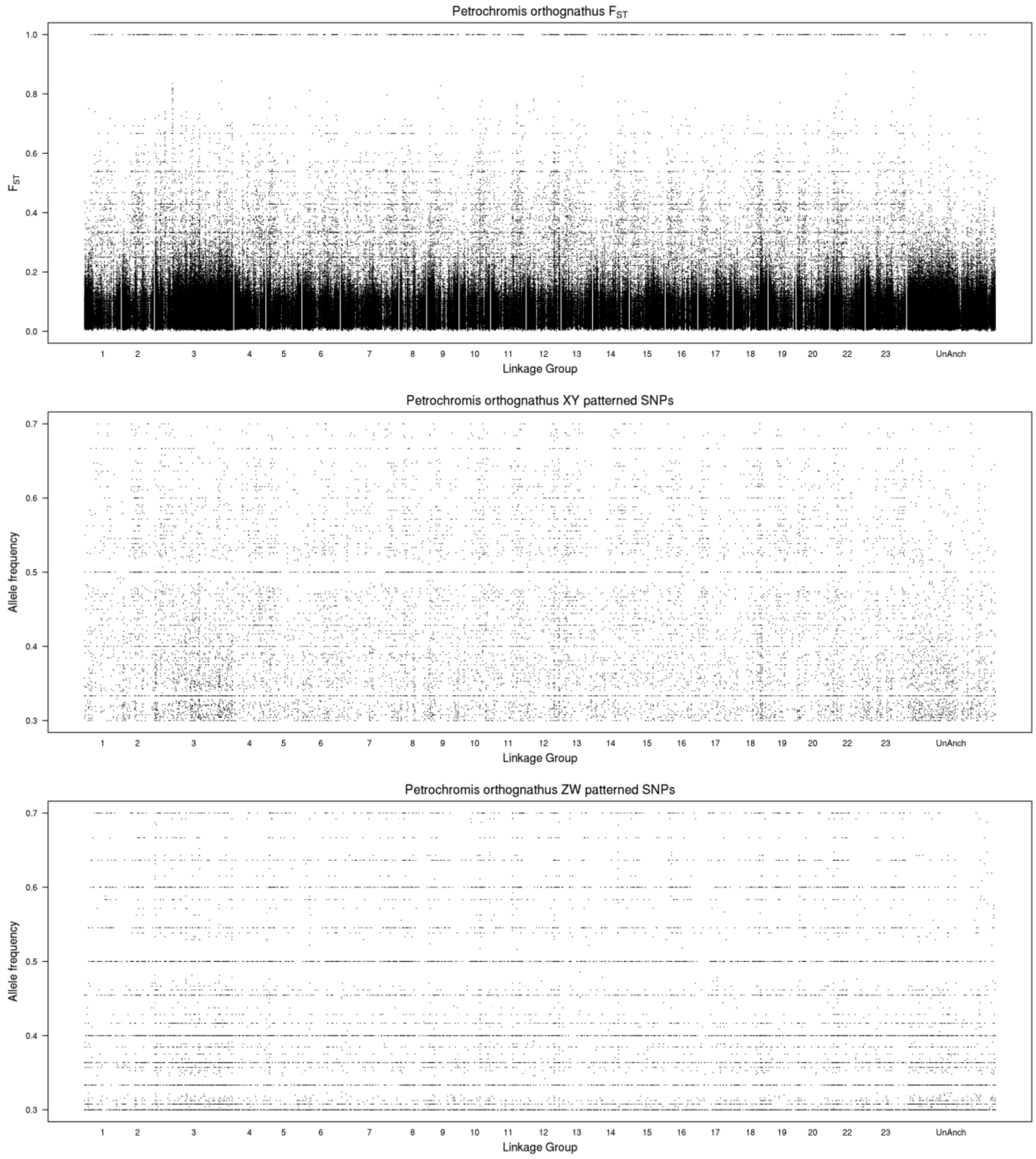

Petrochromis orthognathus F_{ST}
F_{ST}
1.0
0.8
0.6
0.4
0.2
0.0
1 2 3 4 5 6 7 8 9 10 11 12 13 14 15 16 17 18 19 20 22 23 UnAnch
Linkage Group
Petrochromis orthognathus XY patterned SNPs
Allele frequency
0.7
0.6
0.5
0.4
0.3
1 2 3 4 5 6 7 8 9 10 11 12 13 14 15 16 17 18 19 20 22 23 UnAnch
Linkage Group
Petrochromis orthognathus ZW patterned SNPs
Allele frequency
0.7
0.6
0.5
0.4
0.3
1 2 3 4 5 6 7 8 9 10 11 12 13 14 15 16 17 18 19 20 22 23 UnAnch
Linkage Group

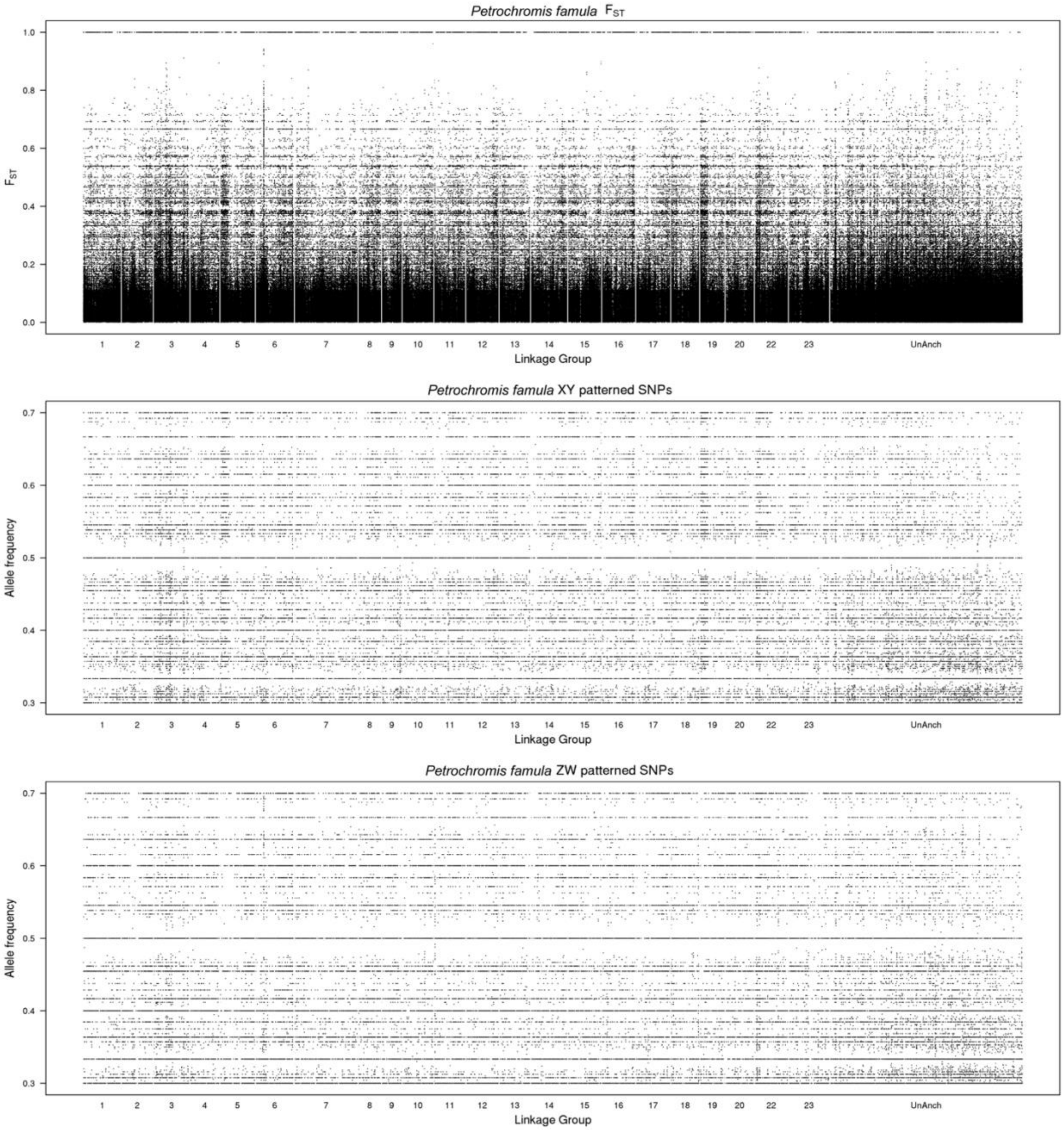

Petrochromis famula F_{ST}
F_{ST}
Linkage Group
1 2 3 4 5 6 7 8 9 10 11 12 13 14 15 16 17 18 19 20 22 23 UnAnch
Petrochromis famula XY patterned SNPs
Allele frequency
Linkage Group
Petrochromis famula ZW patterned SNPs
Allele frequency
Linkage Group

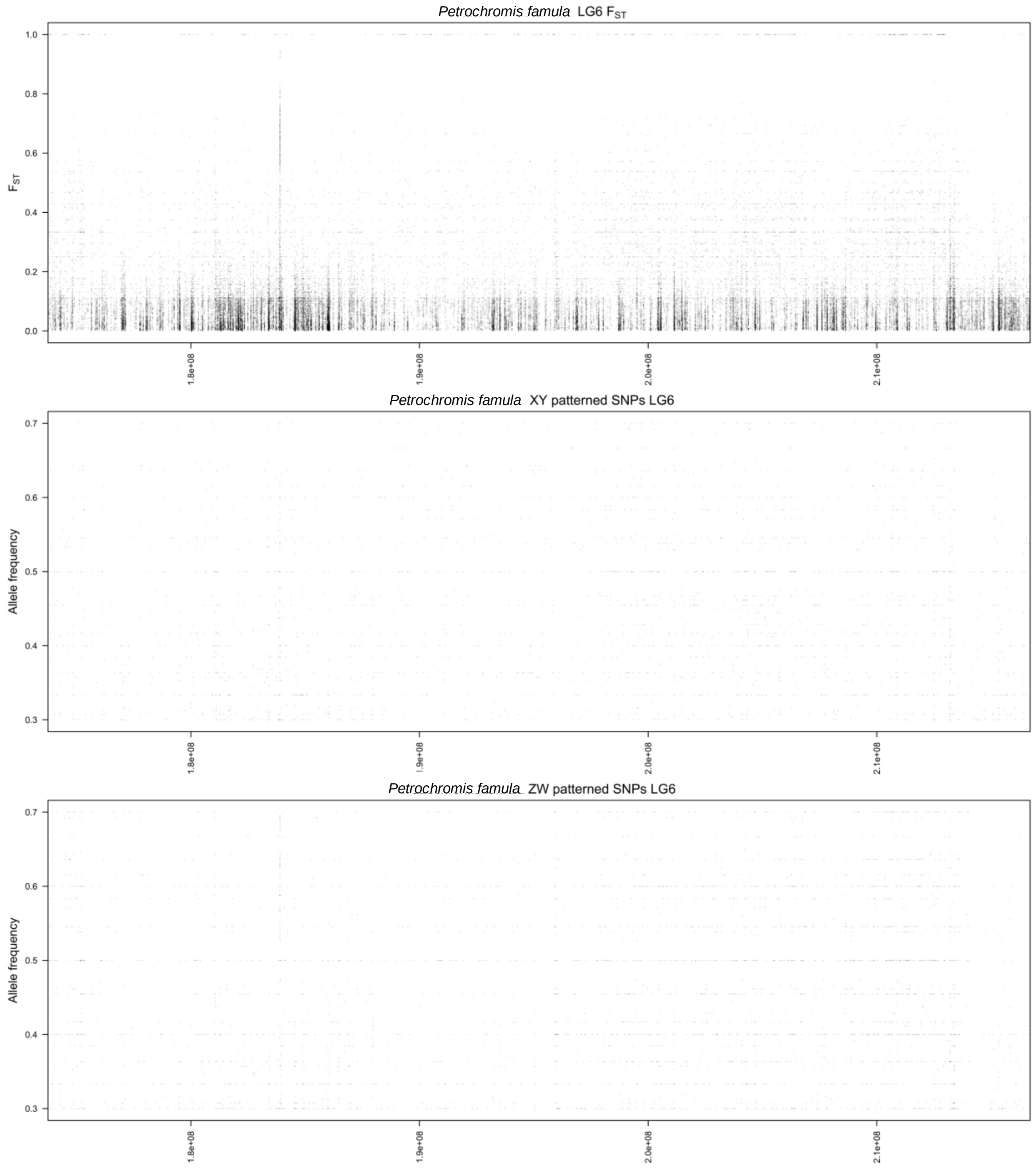
Petrochromis famula LG6 F_{ST}
F_{ST}
1.0
0.8
0.6
0.4
0.2
0.0
1.8e+08
1.9e+08
2.0e+08
2.1e+08
Petrochromis famula XY patterned SNPs LG6
Allele frequency
0.7
0.6
0.5
0.4
0.3
1.8e+08
1.9e+08
2.0e+08
2.1e+08
Petrochromis famula ZW patterned SNPs LG6
Allele frequency
0.7
0.6
0.5
0.4
0.3
1.8e+08
1.9e+08
2.0e+08
2.1e+08

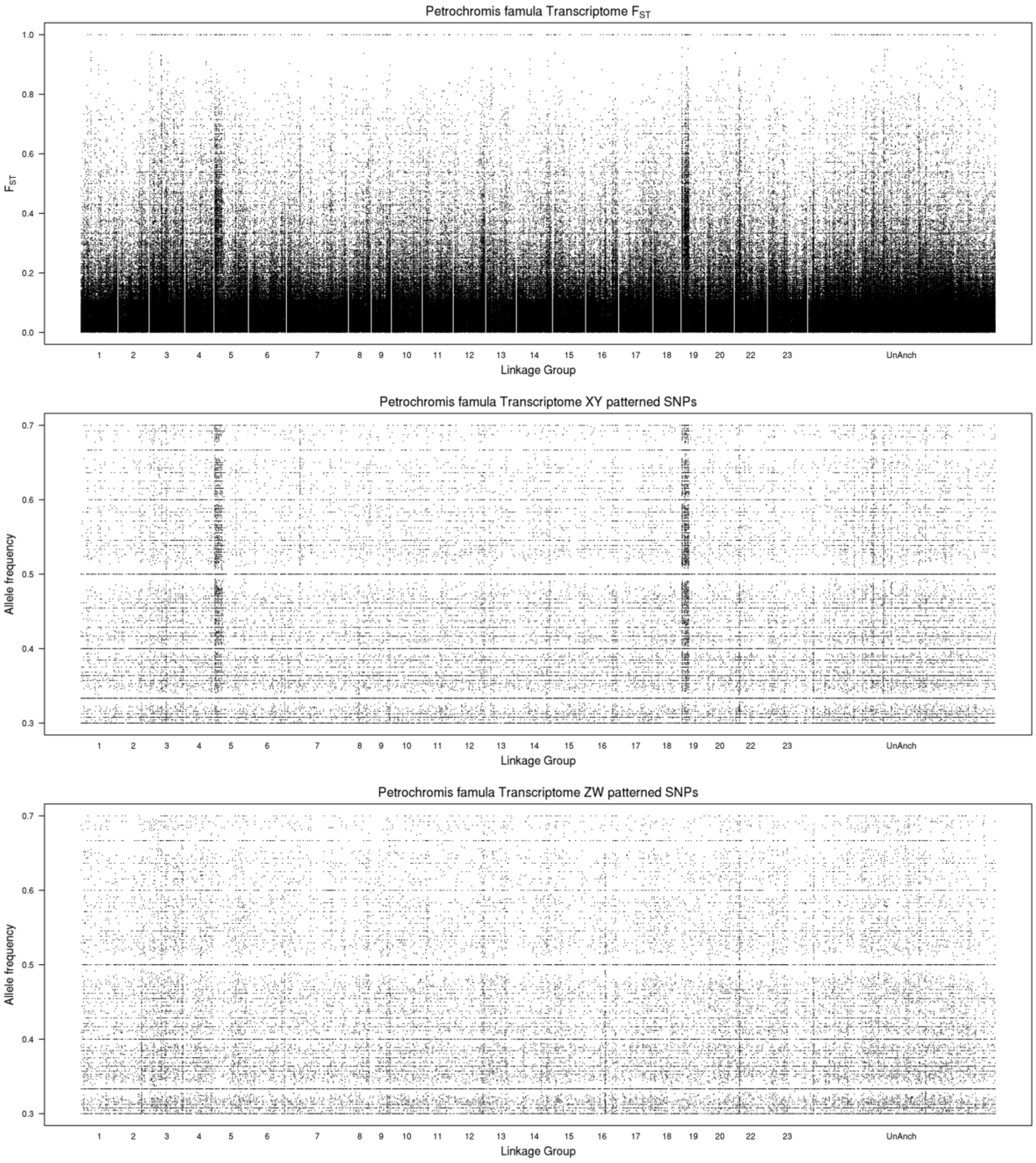

Petrochromis famula Transcriptome F_{ST}
F_{ST}
1.0
0.8
0.6
0.4
0.2
0.0
1 2 3 4 5 6 7 8 9 10 11 12 13 14 15 16 17 18 19 20 22 23 UnAnch
Linkage Group
Petrochromis famula Transcriptome XY patterned SNPs
Allele frequency
0.7
0.6
0.5
0.4
0.3
1 2 3 4 5 6 7 8 9 10 11 12 13 14 15 16 17 18 19 20 22 23 UnAnch
Linkage Group
Petrochromis famula Transcriptome ZW patterned SNPs
Allele frequency
0.7
0.6
0.5
0.4
0.3
1 2 3 4 5 6 7 8 9 10 11 12 13 14 15 16 17 18 19 20 22 23 UnAnch
Linkage Group

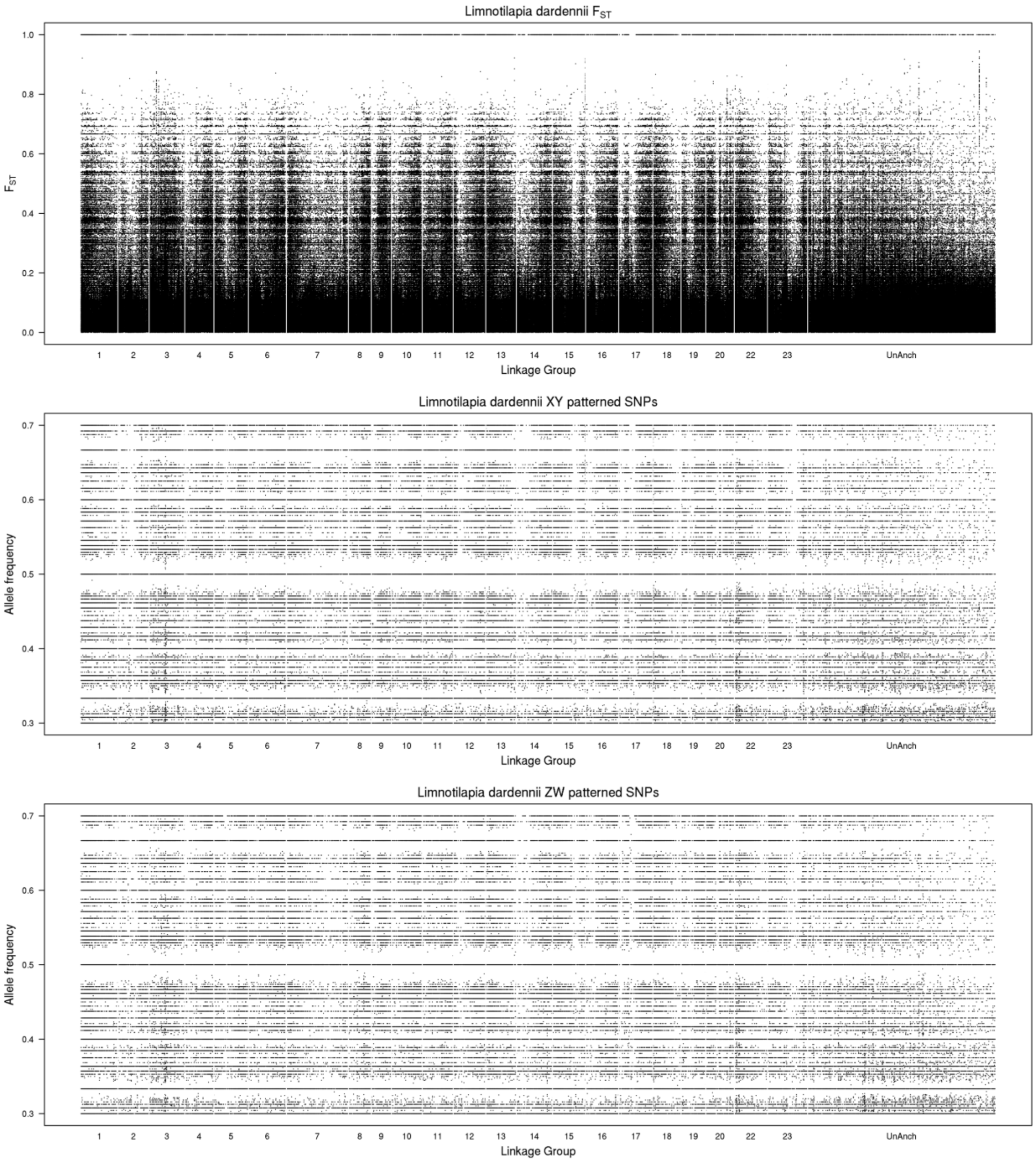

Limnotilapia dardennii F_{ST}
F_{ST}
1.0
0.8
0.6
0.4
0.2
0.0
1 2 3 4 5 6 7 8 9 10 11 12 13 14 15 16 17 18 19 20 22 23 UnAnch
Linkage Group
Limnotilapia dardennii XY patterned SNPs
Allele frequency
0.7
0.6
0.5
0.4
0.3
1 2 3 4 5 6 7 8 9 10 11 12 13 14 15 16 17 18 19 20 22 23 UnAnch
Linkage Group
Limnotilapia dardennii ZW patterned SNPs
Allele frequency
0.7
0.6
0.5
0.4
0.3
1 2 3 4 5 6 7 8 9 10 11 12 13 14 15 16 17 18 19 20 22 23 UnAnch
Linkage Group

Pseudosimochromis curvifrons F_{ST}

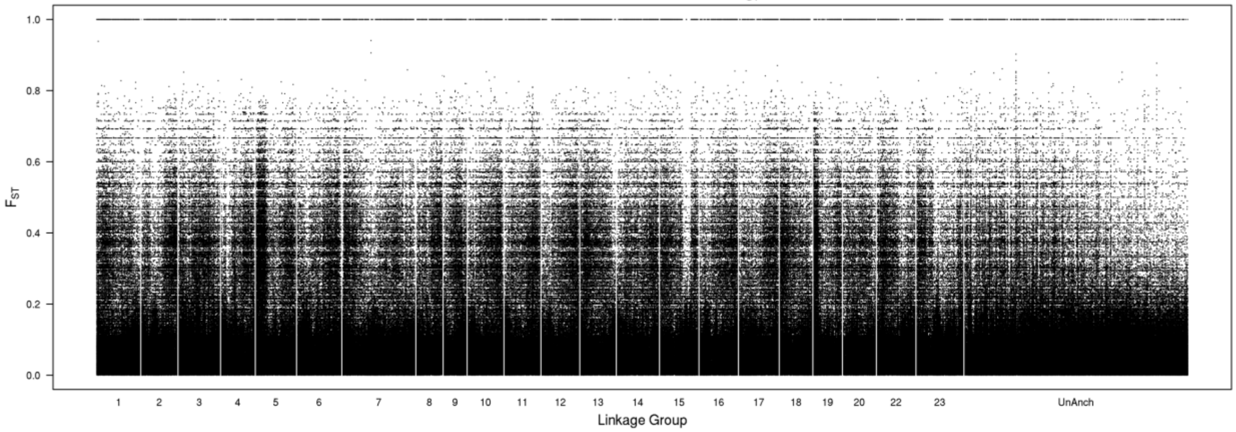

Pseudosimochromis curvifrons South XY patterned SNPs

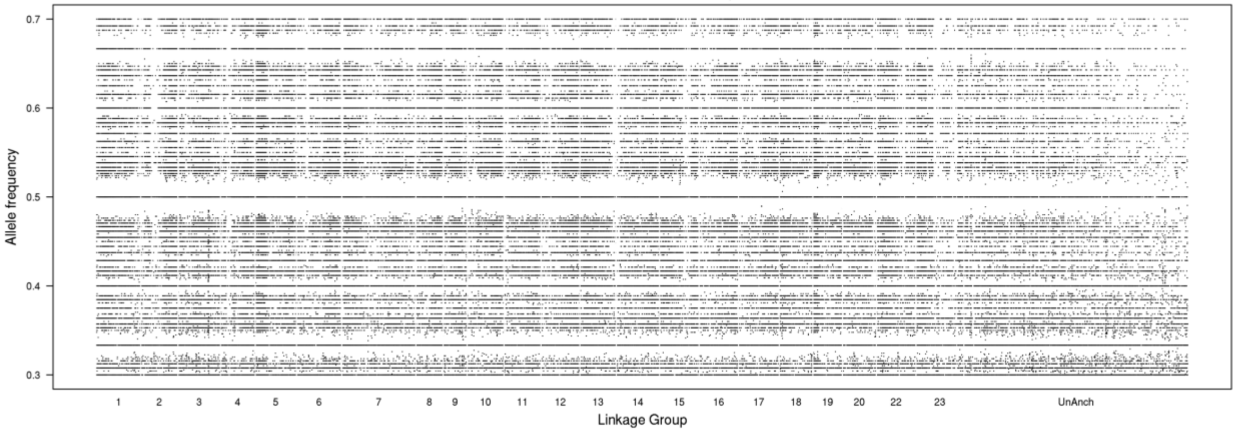

Pseudosimochromis curvifrons ZW patterned SNPs

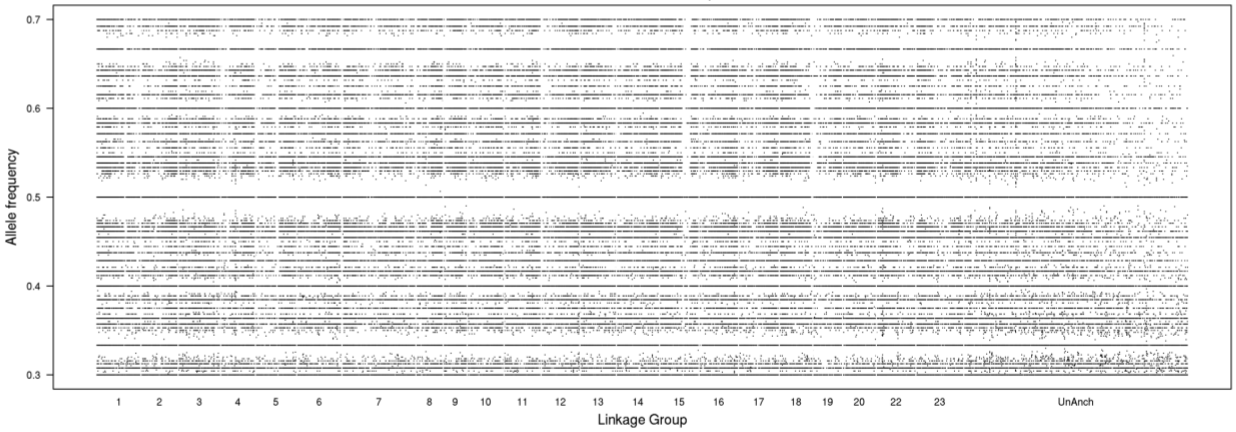

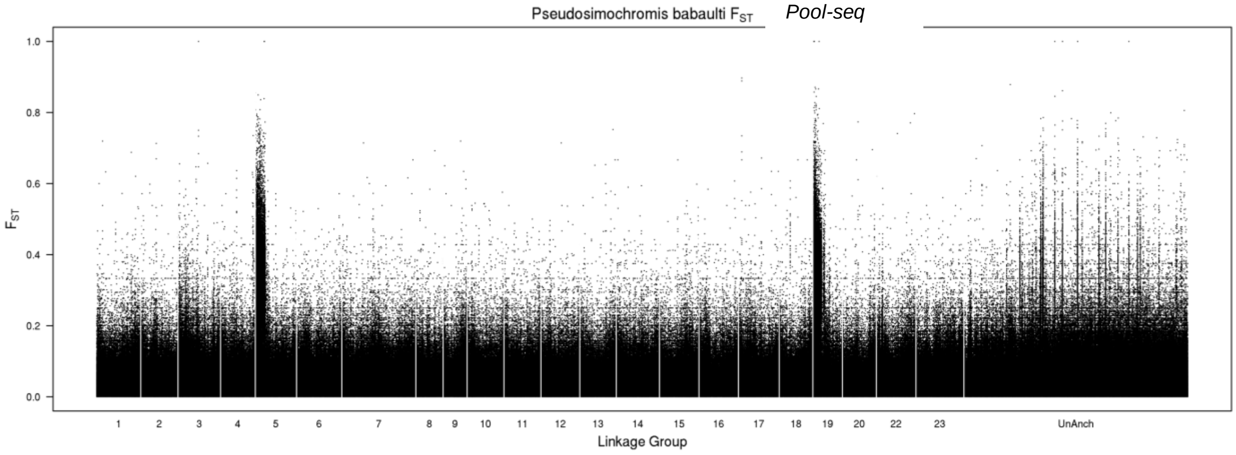

Pseudosimochromis babaulti F_{ST}
Pool-seq
F_{ST}
1.0
0.8
0.6
0.4
0.2
0.0
1 2 3 4 5 6 7 8 9 10 11 12 13 14 15 16 17 18 19 20 22 23 UnAnch
Linkage Group

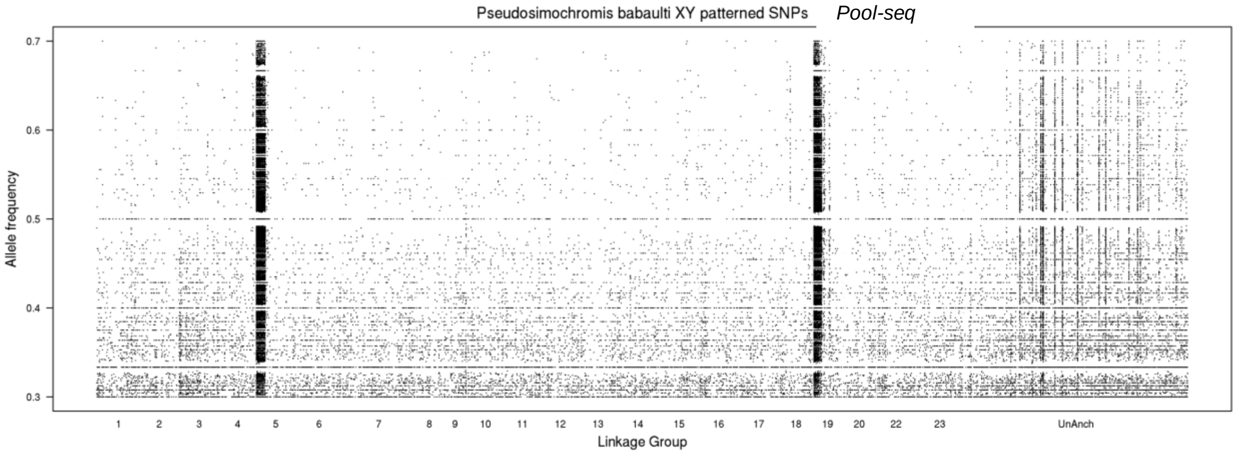

Pseudosimochromis babaulti XY patterned SNPs
Pool-seq
Allele frequency
0.7
0.6
0.5
0.4
0.3
1 2 3 4 5 6 7 8 9 10 11 12 13 14 15 16 17 18 19 20 22 23 UnAnch
Linkage Group

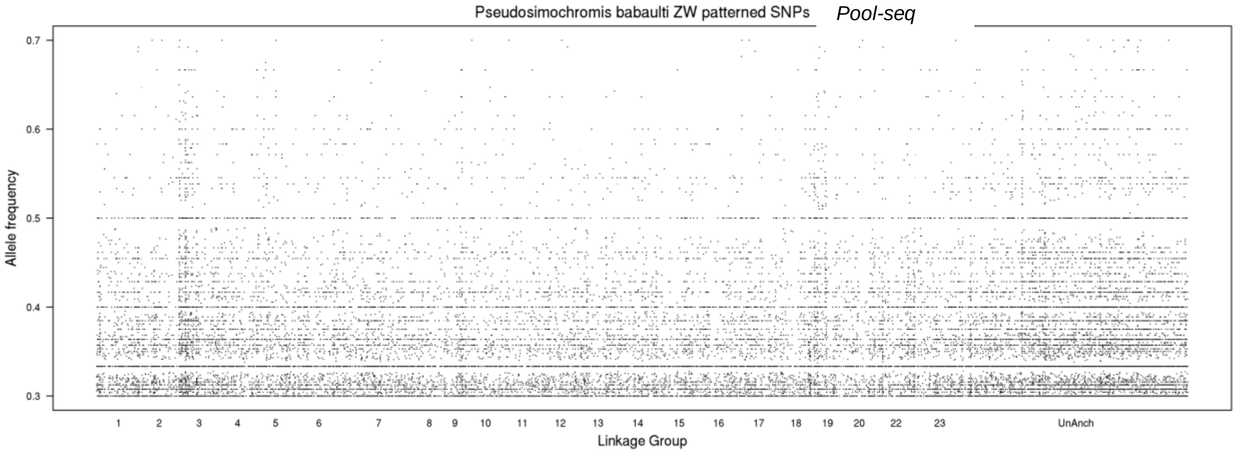

Pseudosimochromis babaulti ZW patterned SNPs
Pool-seq
Allele frequency
0.7
0.6
0.5
0.4
0.3
1 2 3 4 5 6 7 8 9 10 11 12 13 14 15 16 17 18 19 20 22 23 UnAnch
Linkage Group

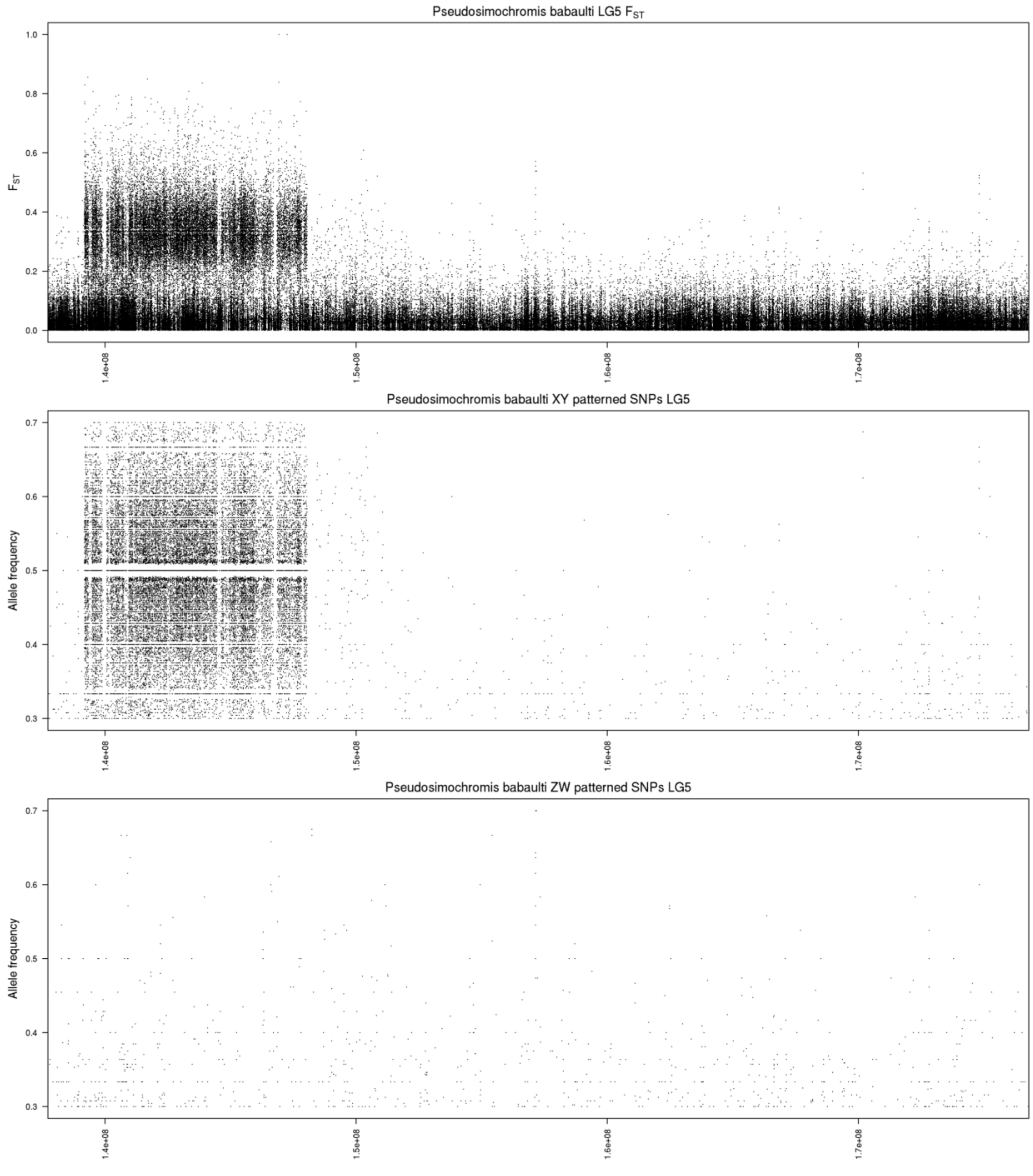

Pseudosimochromis babaulti LG5 F_{ST}
F_{ST}
1.0
0.8
0.6
0.4
0.2
0.0
1.4e+08
1.5e+08
1.6e+08
1.7e+08
Pseudosimochromis babaulti XY patterned SNPs LG5
Allele frequency
0.7
0.6
0.5
0.4
0.3
1.4e+08
1.5e+08
1.6e+08
1.7e+08
Pseudosimochromis babaulti ZW patterned SNPs LG5
Allele frequency
0.7
0.6
0.5
0.4
0.3
1.4e+08
1.5e+08
1.6e+08
1.7e+08

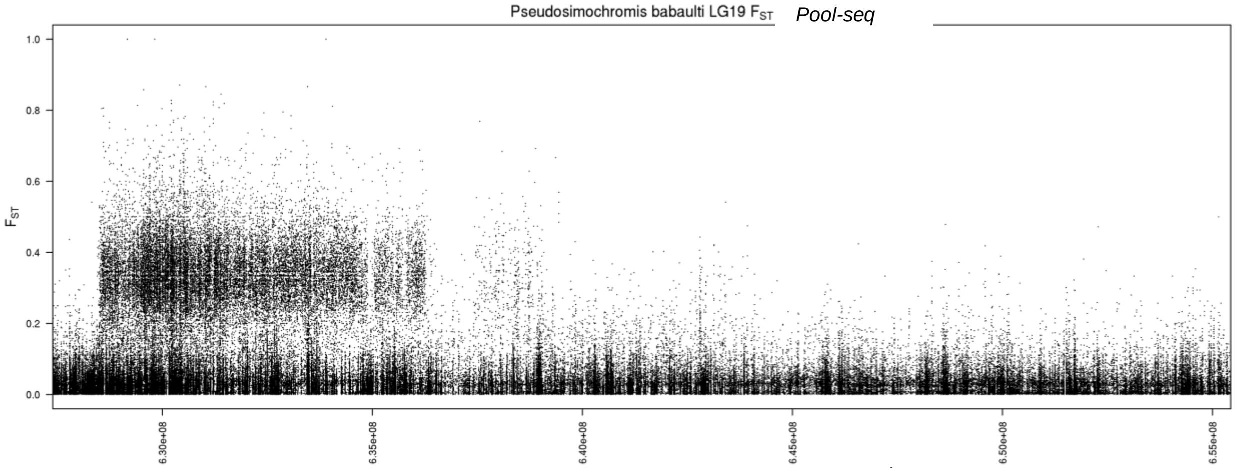
Pseudosimochromis babaulti LG19 F_{ST}
Pool-seq
F_{ST}
1.0
0.8
0.6
0.4
0.2
0.0
6.30e+08
6.35e+08
6.40e+08
6.45e+08
6.50e+08
6.55e+08

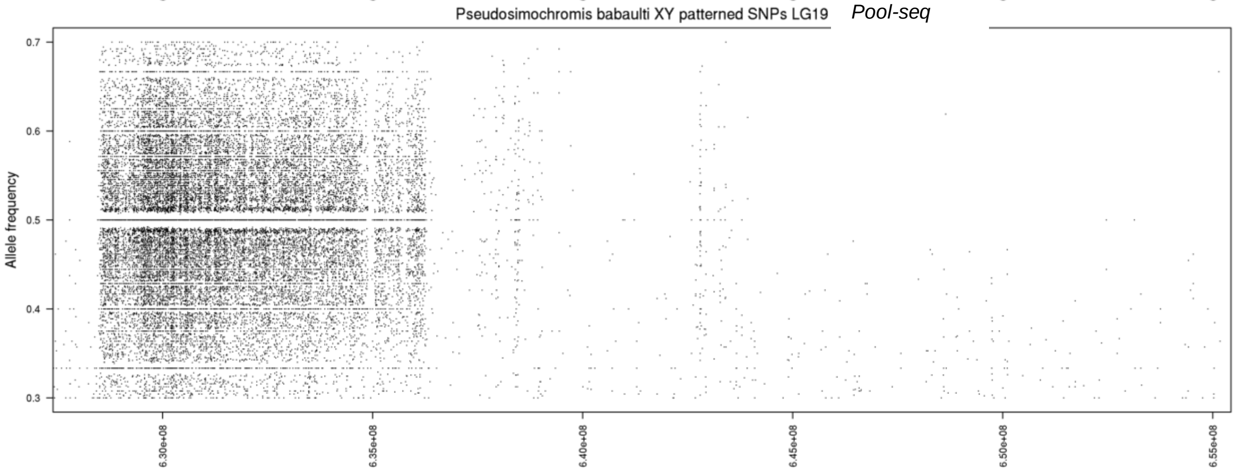
Pseudosimochromis babaulti XY patterned SNPs LG19
Pool-seq
Allele frequency
0.7
0.6
0.5
0.4
0.3
6.30e+08
6.35e+08
6.40e+08
6.45e+08
6.50e+08
6.55e+08

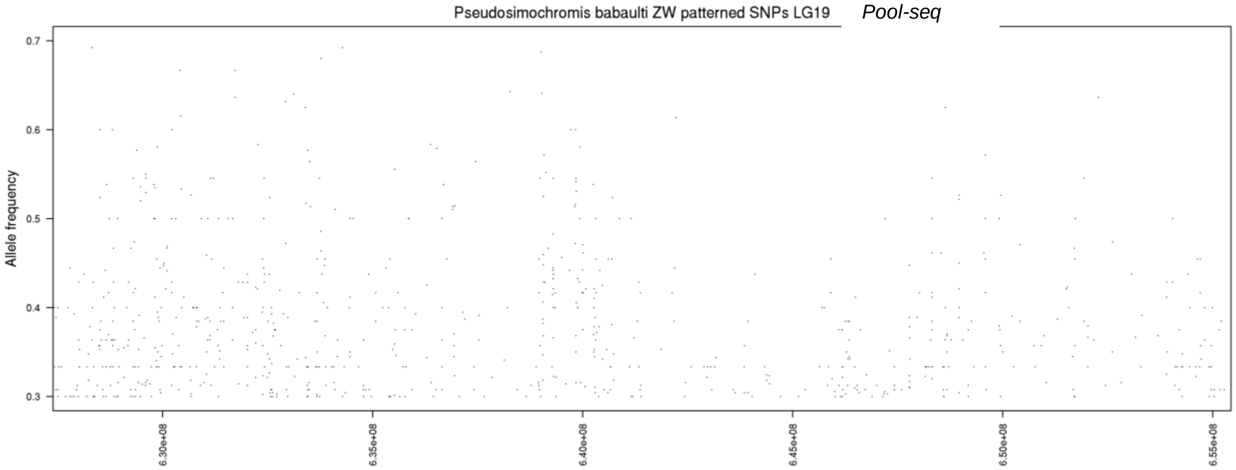
Pseudosimochromis babaulti ZW patterned SNPs LG19
Pool-seq
Allele frequency
0.7
0.6
0.5
0.4
0.3
6.30e+08
6.35e+08
6.40e+08
6.45e+08
6.50e+08
6.55e+08

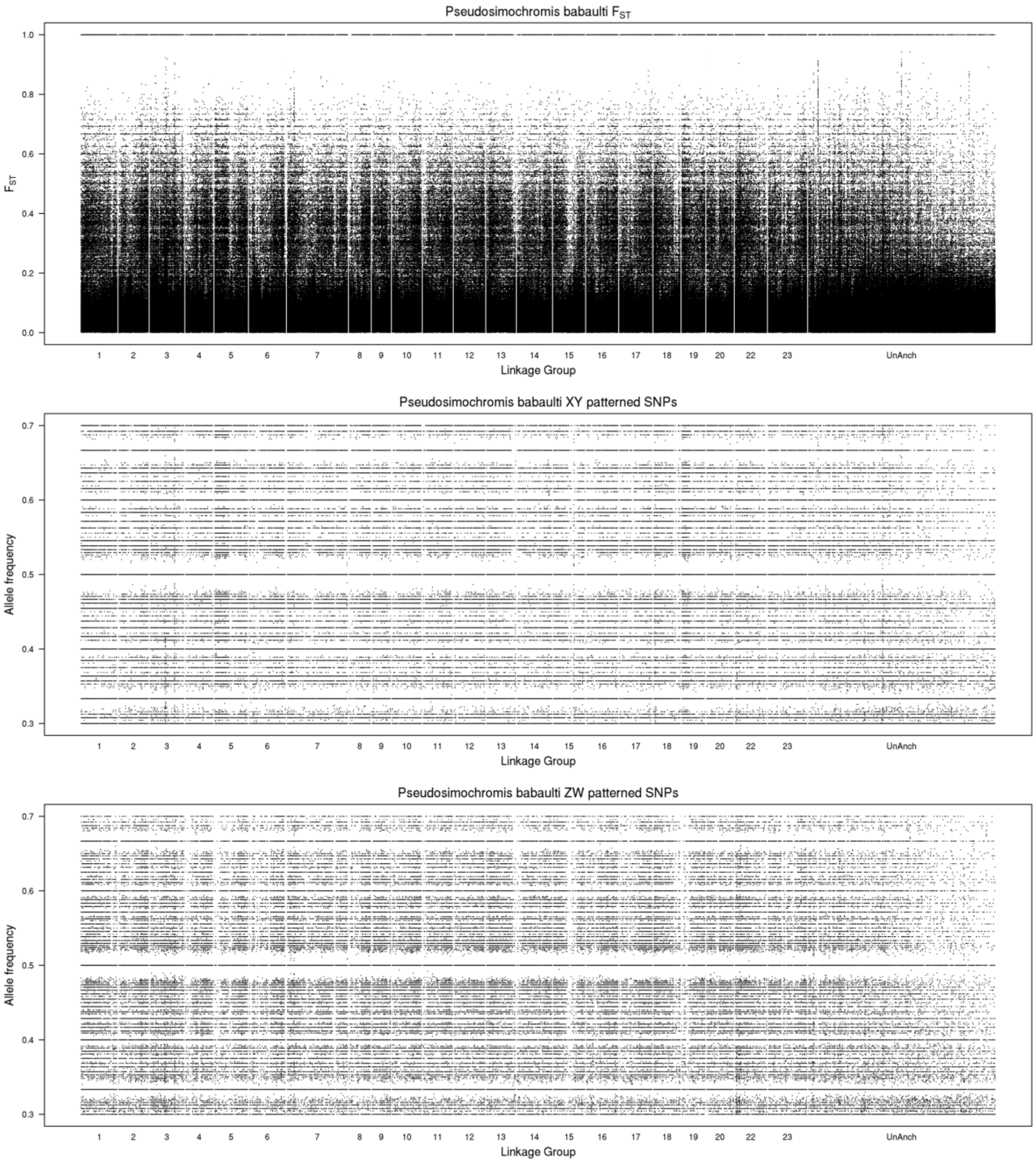

Pseudosimochromis babaulti F_{ST}
F_{ST}
Linkage Group
1 2 3 4 5 6 7 8 9 10 11 12 13 14 15 16 17 18 19 20 22 23 UnAnch
Pseudosimochromis babaulti XY patterned SNPs
Allele frequency
Linkage Group
Pseudosimochromis babaulti ZW patterned SNPs
Allele frequency
Linkage Group

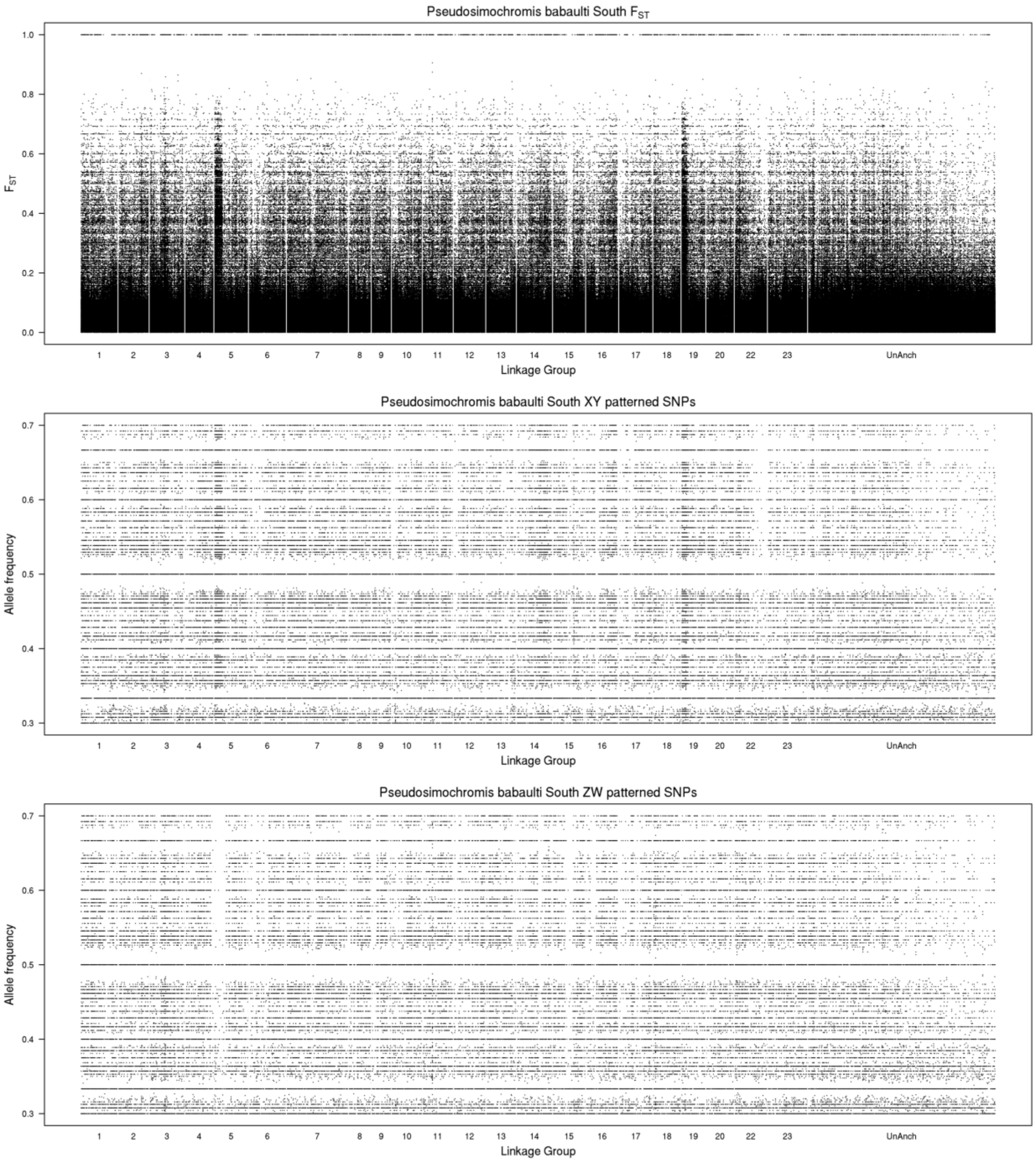

Pseudosimochromis babaulti South F_{ST}
F_{ST}
1.0
0.8
0.6
0.4
0.2
0.0
1 2 3 4 5 6 7 8 9 10 11 12 13 14 15 16 17 18 19 20 22 23 UnAnch
Linkage Group
Pseudosimochromis babaulti South XY patterned SNPs
Allele frequency
0.7
0.6
0.5
0.4
0.3
1 2 3 4 5 6 7 8 9 10 11 12 13 14 15 16 17 18 19 20 22 23 UnAnch
Linkage Group
Pseudosimochromis babaulti South ZW patterned SNPs
Allele frequency
0.7
0.6
0.5
0.4
0.3
1 2 3 4 5 6 7 8 9 10 11 12 13 14 15 16 17 18 19 20 22 23 UnAnch
Linkage Group

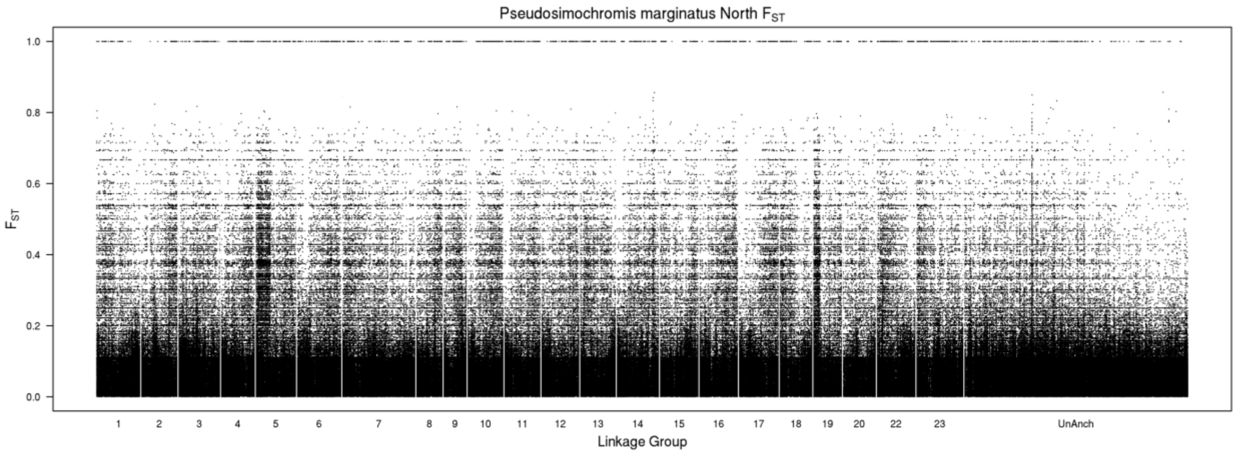
Pseudosimochromis marginatus North F_{ST}
F_{ST}
1.0
0.8
0.6
0.4
0.2
0.0
1 2 3 4 5 6 7 8 9 10 11 12 13 14 15 16 17 18 19 20 22 23 UnAnch
Linkage Group

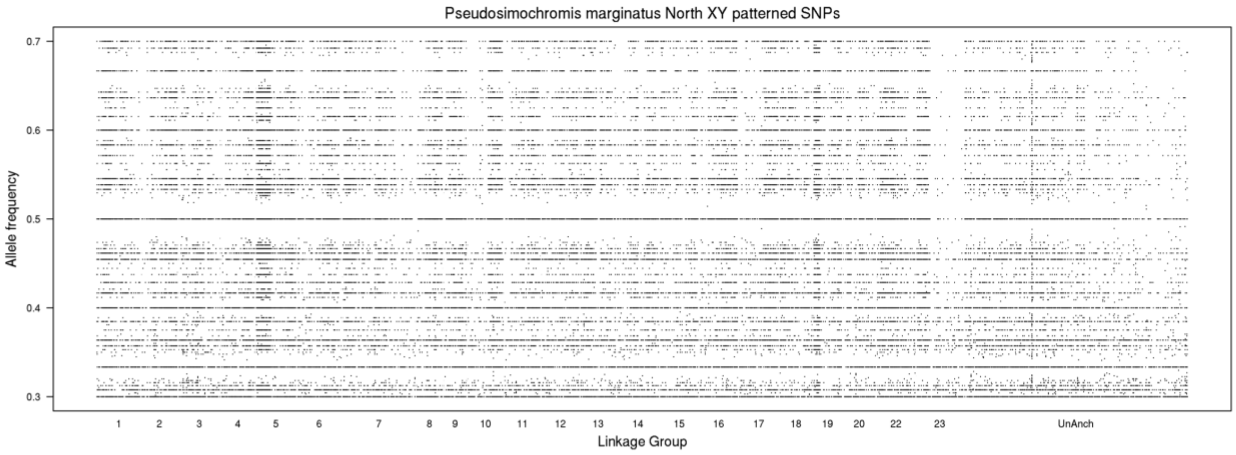
Pseudosimochromis marginatus North XY patterned SNPs
Allele frequency
0.7
0.6
0.5
0.4
0.3
1 2 3 4 5 6 7 8 9 10 11 12 13 14 15 16 17 18 19 20 22 23 UnAnch
Linkage Group

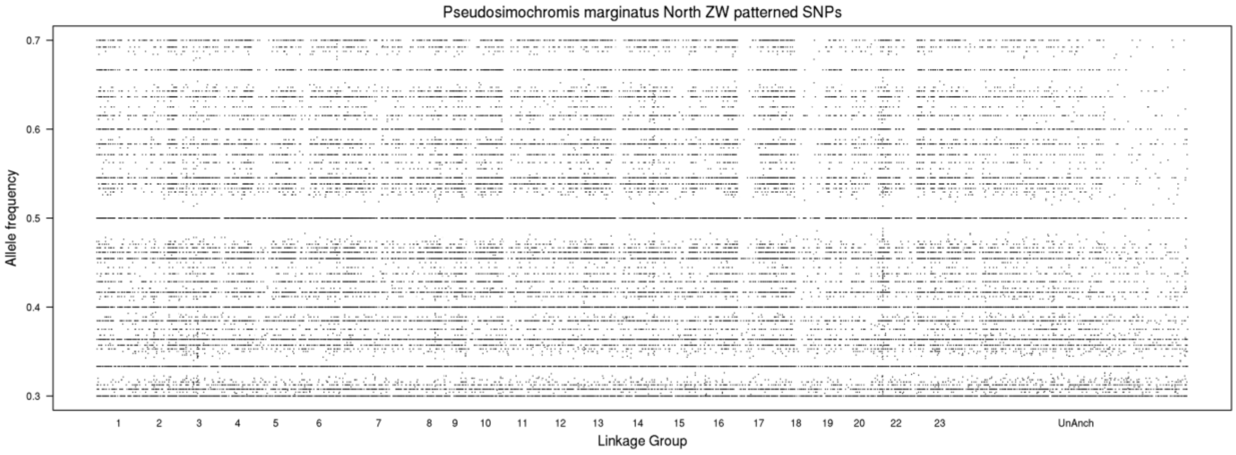
Pseudosimochromis marginatus North ZW patterned SNPs
Allele frequency
0.7
0.6
0.5
0.4
0.3
1 2 3 4 5 6 7 8 9 10 11 12 13 14 15 16 17 18 19 20 22 23 UnAnch
Linkage Group

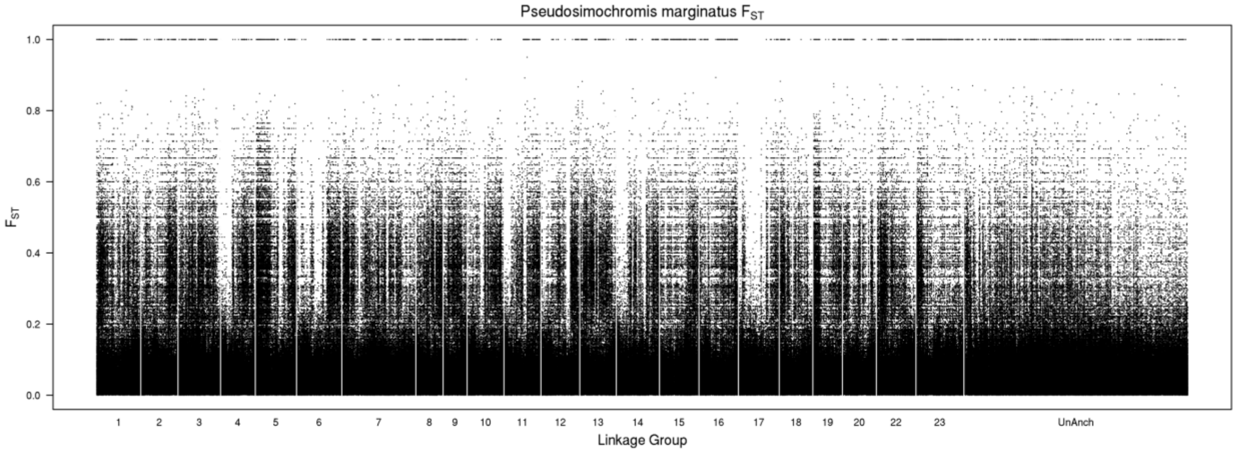
Pseudosimochromis marginatus F_{ST}
F_{ST}
0.0
0.2
0.4
0.6
0.8
1.0
1 2 3 4 5 6 7 8 9 10 11 12 13 14 15 16 17 18 19 20 22 23 UnAnch
Linkage Group

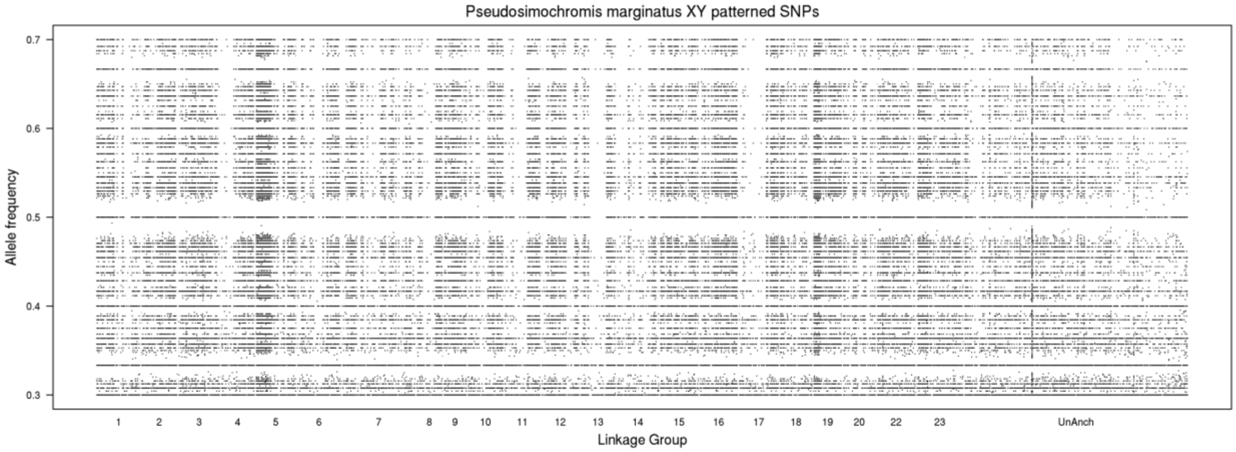
Pseudosimochromis marginatus XY patterned SNPs
Allele frequency
0.3
0.4
0.5
0.6
0.7
1 2 3 4 5 6 7 8 9 10 11 12 13 14 15 16 17 18 19 20 22 23 UnAnch
Linkage Group

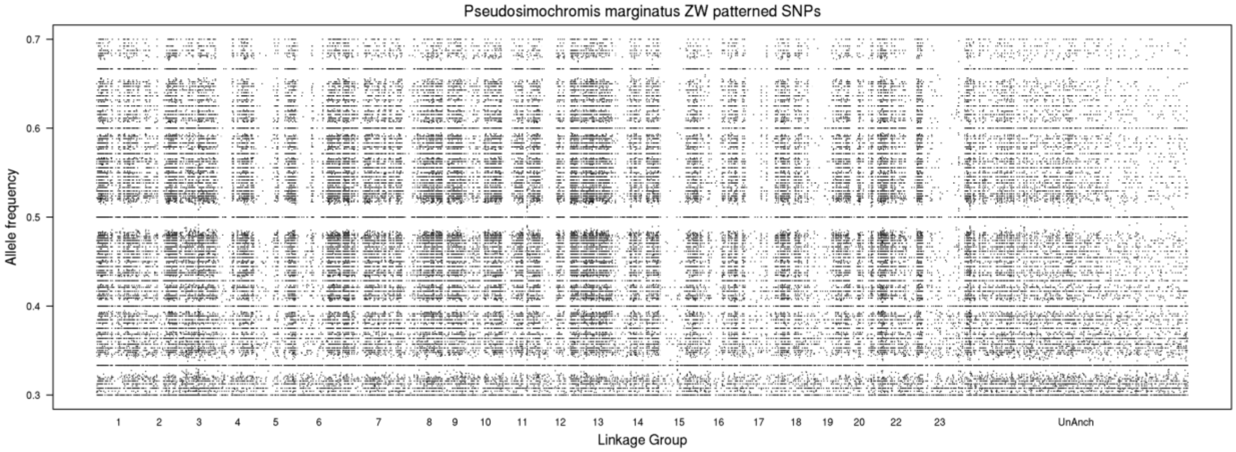
Pseudosimochromis marginatus ZW patterned SNPs
Allele frequency
0.3
0.4
0.5
0.6
0.7
1 2 3 4 5 6 7 8 9 10 11 12 13 14 15 16 17 18 19 20 22 23 UnAnch
Linkage Group
